# Hydroarylation of enamides enabled by HFIP *via* a hexafluoroisopropyl ether as iminium reservoir†

**DOI:** 10.1039/d2sc02012b

**Published:** 2022-06-27

**Authors:** Nicolas Zeidan, Sergiu Bicic, Robert J. Mayer, David Lebœuf, Joseph Moran

**Affiliations:** Institut de Science et d’Ingénierie Supramoléculaires (ISIS), CNRS UMR 7006, Université de Strasbourg 8 allée Gaspard Monge 67000 Strasbourg France dleboeuf@unistra.fr moran@unistra.fr

## Abstract

Here we describe that HFIP greatly expands the scope with respect to both reaction partners of the Brønsted acid-catalyzed hydroarylation of enamides. The reaction is fast and practical and can be performed on the gram scale. A hexafluoroisopropyl ether intermediate was isolated from the reaction mixture and was shown to convert to the product when resubmitted to the reaction conditions. Extensive kinetic studies and computations reveal that the hexafluoroisopropyl ether is formed rapidly and serves as a slow-release reservoir for the key cationic intermediate, preventing the oligomerization of the substrate under the reaction conditions. Given the relatively low electrophilicity of the cationic intermediates in the present study, it seems likely that HFIP also actively participates in other reactions involving more electrophilic carbocations.

## Abstract 2

Hexafluoroisopropanol reversibly traps and releases iminium ions to greatly expand the scope of enamide hydroarylation, a mechanism that is likely common to other reactions featuring carbocations in that solvent.

## Graphical abstract

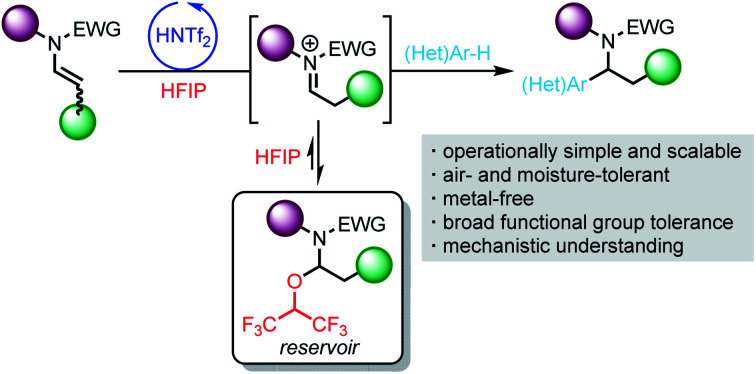

## Introduction

**Scheme 1:**
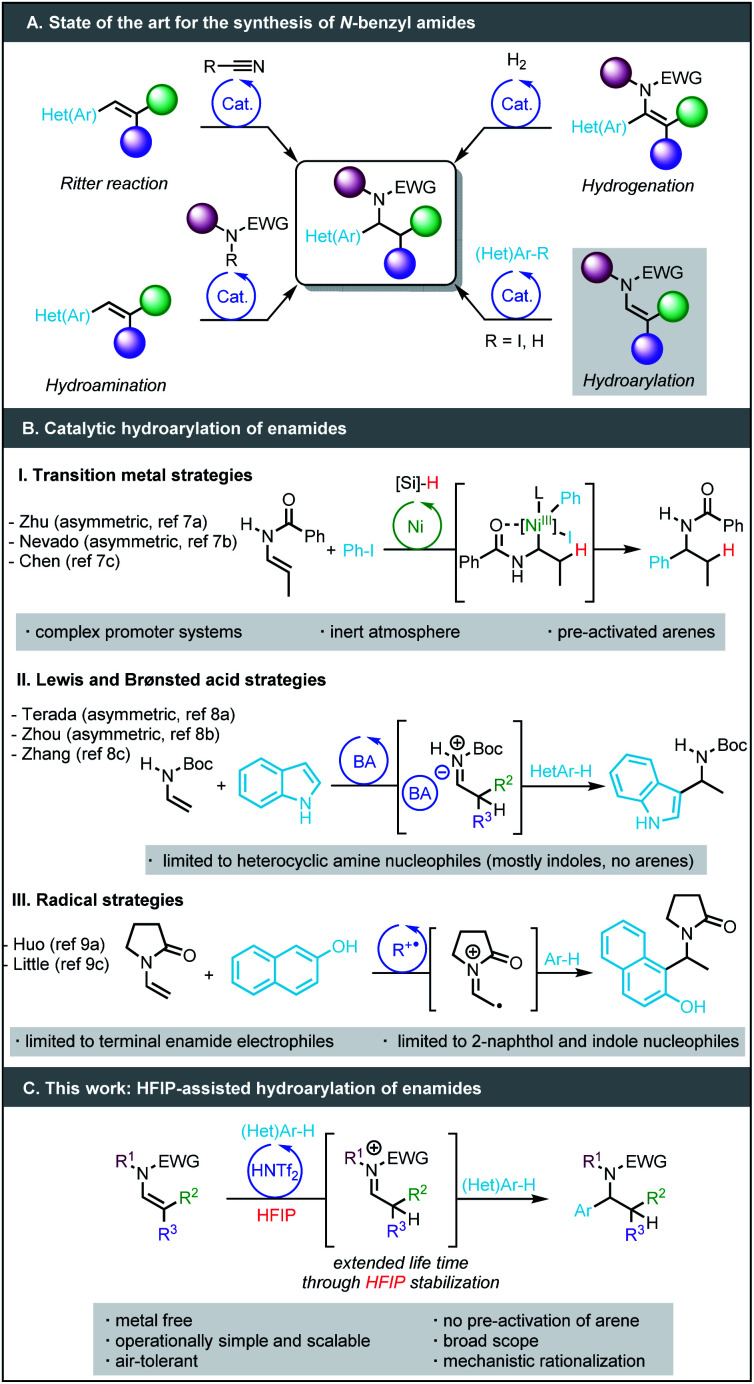
Strategies toward the synthesis of *N*-benzyl amides.

*N*-Benzyl amides, and more generally *N*-benzyl amines, are present in numerous biologically active compounds, natural products, pharmaceuticals, and agrochemicals.^1^ The development of new, simple, yet synthetically useful methods for their preparation is highly sought. Among the methods of choice to access *N*-benzyl amides are the intermolecular hydroamidation of styrenes^2^ and the Ritter reaction,^3^ the scope of both of which are limited (Scheme 1A). The hydroamidation is generally not compatible with a large variety of *N*-protecting groups; the Ritter reaction can only form secondary amides. As an alternative, several protocols involving the hydrogenation of versatile enamides^4^ bearing an aryl group in the alpha position have been developed;^5^ however, the preparation of the requisite starting materials may require multi-step synthesis.

More recently, the groups of Zhu and Nevado pioneered nickel-catalysed enantioselective reductive hydroarylation^6^ of enamides with iodoarenes (Scheme 1B).^7^ These reactions assemble more complex *N*-benzyl amides from simpler enamide precursors, but require sophisticated ligands, a large excess of silane as a hydride source, inert conditions (*i.e.* oxygen/water-free) and pre-activated arenes. Only three examples of redox neutral hydroarylation of enamides to *N*-benzyl amides have been reported. The groups of Terada, Zhou and Zhang showed that Brønsted acids (chiral phosphoric acid) or Lewis acids (FeCl_3_) promote the coupling of enamides with strongly electron-rich, dual H-bond donor/acceptor (hetero)arenes, such as indoles and 2-naphthols *via* the formation of a postulated acyl iminium intermediate.^8^ The limitation of enamide hydroarylation to these specific nucleophiles is likely due to the relative instability of the iminium intermediates. Radical processes are fraught with similar limitations.^9^

Our group has ongoing interest in using hexafluoroisopropanol (HFIP)^10^ as a solvent to overcome limitations in Brønsted or Lewis acid-catalysed transformations.^11,12^ HFIP can augment the lifetime of cationic intermediates due to its high polarity, low nucleophilicity, and its capacity to stabilize transient intermediates by forming strong H-bond networks. We anticipated that these properties could increase the stability of the iminium intermediate and expand the scope of the hydroarylation reaction.^13^ Herein we disclose a method based on the use of HFIP that enables the hydroarylation of enamides by widely available (hetero)arenes (Scheme 1C), turning this redox-neutral transformation into a complementary alternative to reductive transition metal-catalysed methods employing iodoarenes. Among the advantages of this approach are (i) an operationally simple and demonstrably scalable metal-free protocol, (ii) the use of (hetero)arenes that are not pre-functionalised, and (iii) a broad functional group tolerance. Perhaps most importantly, (iv) a detailed mechanistic investigation featuring kinetic studies and DFT computations demonstrates that reversibly formed HFIP ether intermediates are crucial to the success of this transformation, providing new insights into HFIP-mediated processes in general.

## Results and discussion

### Optimisation studies

We began our studies by investigating the reactivity of *N*-vinylformamide 1a (NVF) with mesitylene 2a in the presence of triflic acid (TfOH, 10 mol%) at 60 °C (Table 1). After 4 h, the target product 3a was obtained in 92% yield (entry 1). In contrast, the use of common organic solvents such as MeCN, DCM, and MeNO_2_ led only to the oligomerization of the enamide (entries 2–4). In the case of iPrOH, while the reaction did not yield 3a, hemiaminal 4 was isolated in 72% yield (entry 5). Trifluoroethanol (TFE) also proved to be a suitable solvent for the reaction, affording 3a in 90% yield (entry 6). Then, the influence of the reaction parameters on the reaction outcome was examined. Lowering the concentration, the catalyst loading, or the amount of mesitylene led to a decrease in efficiency (entries 7–9). Of note, the reaction could be conducted at ambient temperature, albeit at a slower rate (entry 10). The use of bistrifluoromethylsulfonimide (HNTf_2_) delivers 3a in a nearly quantitative yield (entry 11), and it was subsequently chosen for exploring the scope of the transformation.

**Table 1:** Optimisation of reaction conditions for the formation of *N*-benzyl amide 3a

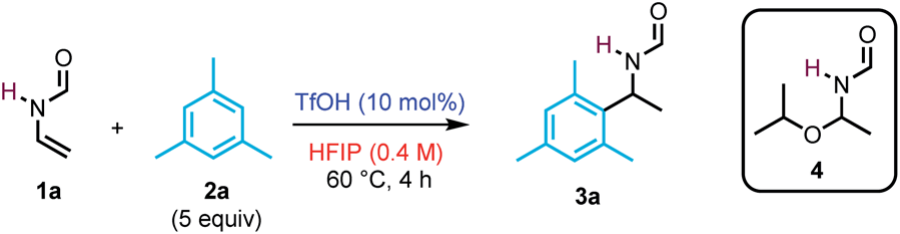		
Entry	Variation from standard conditions^a^	Yield 3a (%)
1	None	92
2	MeCN instead of HFIP	—
3	DCM instead of HFIP	—
4	MeNO_2_ instead of HFIP	—
5	iPrOH instead of HFIP	–(72)^c^
6	TFE instead of HFIP	90
7	0.2 M instead of 0.4 M	83
8	5 mol% TfOH instead of 10 mol%	74
9	2 equiv. 2a instead of 5 equiv.	77
10^b^	22 °C instead of 60 °C	75
11	HNTf_2_ instead of TfOH	99
12	Without HNTf_2_	n.r.

a Reactions performed in a sealed tube.

b 18 h reaction time.

c Yield of product 4.

### Scope and limitations

**Scheme 2:**
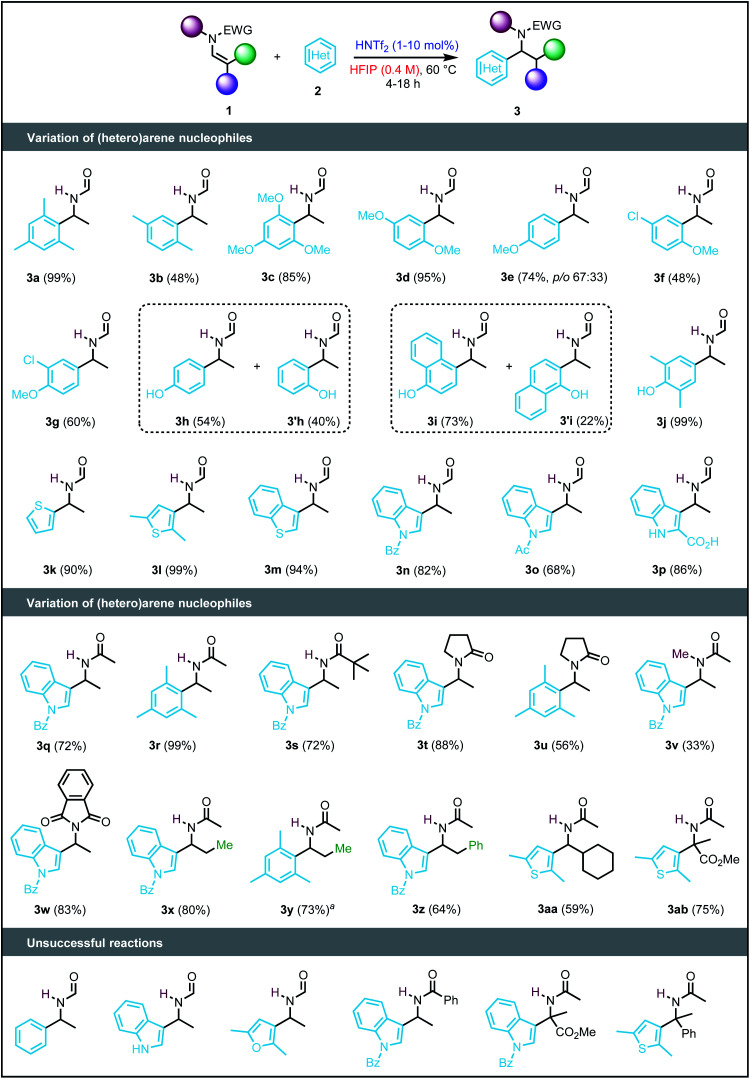
Scope and limitations of the reaction. ^*a*^TfOH used as a catalyst.

Having established the optimal reaction conditions, we next explored the scope of (hetero)arenes using 1a as an electrophile (Scheme 2). The reaction tolerated most of the (hetero)arenes tested, affording the products 3 in a range from 48% to quantitative yields (3a–3p). However, the arenes must be sufficiently nucleophilic to enable the reaction, as no product was observed with benzene. Importantly, arenes incorporating halide functionalities, such as 3- and 4-chloroanisole, were tolerant to our reaction conditions to afford 3f and 3g, in 48% and 60% yield, respectively. In the case of phenol and 1-naphthol, a mixture of regioisomers was obtained that could be separated by flash column chromatography to provide the corresponding products (3h/3h′ and 3i/3i′) in synthetically useful yields. In turn, using an *ortho*-disubstituted phenol led to the target product 3j in a nearly quantitative yield. With respect to heteroarenes, thiophene derivatives (3k–3m) were obtained in high yields (83–99%). The robustness of this catalytic system was further demonstrated by a gram-scale (15 mmol) synthesis of compound 3n starting from 2,5-dimethylthiophene (2.74 g, 99% yield). Furans and free (NH)-indoles underwent direct oligomerisation, likely due to their protonation/decomposition. However, by adding an electron-withdrawing group at nitrogen or the C-2 position, indoles became stable under the reaction conditions, delivering products 3n–3p in yields from 52 to 86%. Of note, in the case of heterocyclic nucleophiles, the catalyst loading was decreased from 10 mol% to 1 mol% to minimise decomposition of the product or decay pathways of the starting materials. A nitrogen heterocycle – *N*-benzoylindole – and an arene – mesitylene – were then chosen to study the scope of enamides. In general, the reaction was compatible with a broad range of enamides to afford the corresponding products (3q–3ab) in yields ranging from 33% to 99%. The reaction was also tolerant to the use of a bulkier substituent on the nitrogen (3s), albeit at a slower rate (24 h *vs.* 6 h). Attempts to increase the reaction temperature to 80 °C proved to be detrimental to the reactivity as, in most cases, it led to the decomposition of the substrates. On the other hand, *N*-vinylbenzamide only gave traces of the target product, yielding several byproducts resulting from intramolecular and dimerisation reactions. Cyclic enamides such as *N*-vinylpyrrolidin-2-one were tolerated as well to provide products 3t and 3u in 88% and 56% yields, respectively. The reaction was also achieved in 83% yield with 2-vinylisoindoline-1,3-dione. The reaction was not limited to terminal enamides but could be also extended to di- and tri-substituted enamides (3y–3aa, 59–73% yields). Finally, methyl 2-acetamidoacrylate could be employed as electrophile to yield α-methyl-α-3-thienylglycine 3ab in 75% yield. Here, the functional group used is critical, as replacing the ester by a phenyl group led to decomposition of the substrate.

To illustrate the utility of the compounds, the deprotection of formamide 3l was carried out to furnish free amine 5 in 74% yield (eqn (1)). Our methodology also offers a straightforward access to densely functionalised isocyanides such as 6 (eqn (2)),^14^ which are particularly useful as reaction partners in cycloadditions, in multi-component reactions,^15^ or as bioactive molecules.^16^
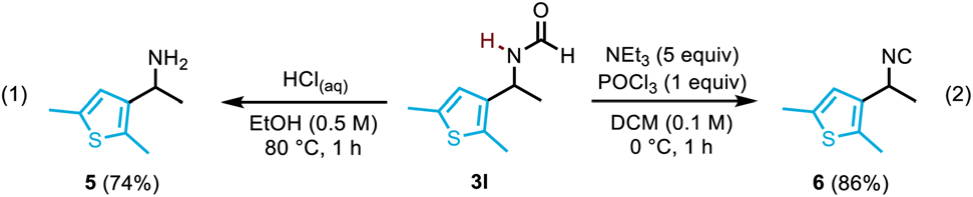


### Mechanistic studies

**Fig. 1:**
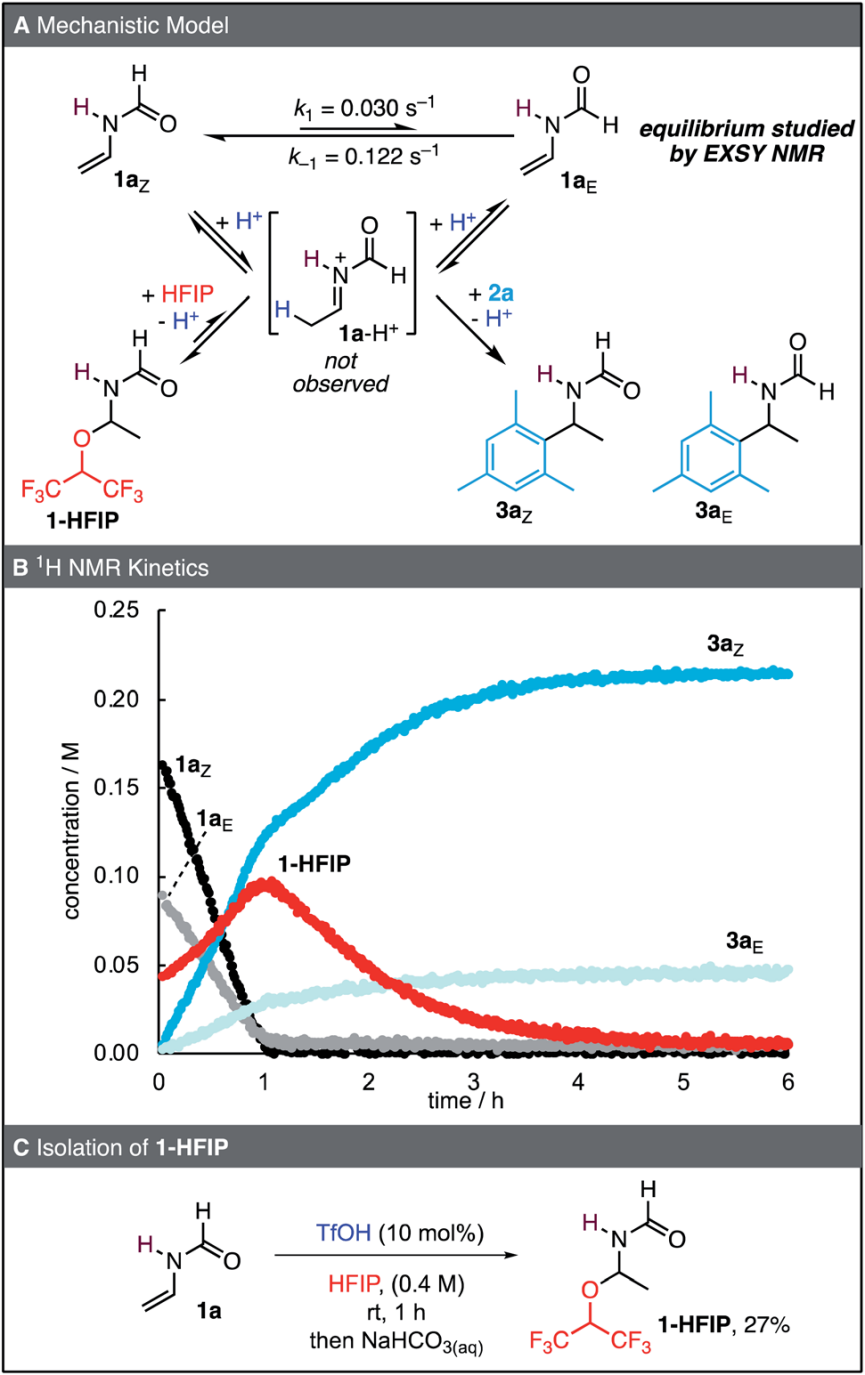
(A) Proposed reaction mechanism and isomerisation rates of 1a from 2D-EXSY NMR at 23 °C. (B) ^1^H NMR kinetics of a reaction of 1a (0.4 M) and 2a (1.6 M) in the presence of TfOH (0.04 M) at 23 °C in HFIP containing 8.3 vol% C_6_D_6_. Concentrations were determined by using tetrachloroethane as an internal standard. (C) Isolation of 1-HFIP.

To obtain further insights into the reaction mechanism, we conducted a series of NMR experiments. On the NMR timescale, 1a exists as a mixture of *E*- and *Z*-isomers, which interconvert *via* a barrier of 81.7 kJ mol^−1^ as determined by a 2D-EXSY experiment (Fig. 1A, ESI†).^17^ This rotational barrier is of similar magnitude to other formamides in DMSO.^18^ When 0.1 equiv. of TfOH was added to a solution of 1a in HFIP followed by 4 equiv. of mesitylene (2a), the resonances of a newly formed hemiaminal 1-HFIP were immediately detected (<1 min, Fig. 1B). Interestingly, such species have only been sporadically reported in the literature.^19^ Within the first hour of the reaction, 1a was completely consumed and both 1-HFIP and the two rotamers 3a_*E*_ and 3a_*Z*_ were formed. During this initial phase of the reaction, the kinetics of all species were found to follow 0^th^-order behaviour. After full consumption of 1a, the concentration of 1-HFIP reached its maximum. Subsequently, the kinetics changed and disappearance of the resonances of 1-HFIP was accompanied by an increase of those of 3a_*E*_/3a_*Z*_. In contrast to the first, linear phase of the reaction, the disappearance of 1-HFIP as well as the formation of both rotamers of 3a could be fitted to mono-exponential decrease and increase functions, respectively, indicating first-order kinetics. To verify the identity of the hemiaminal 1-HFIP, 1a was next subjected to the reaction conditions on a preparative scale. After aqueous workup, 1-HFIP was isolated in 27% yield and was characterized by 2D NMR spectroscopy (Fig. 1C).

**Fig. 2:**
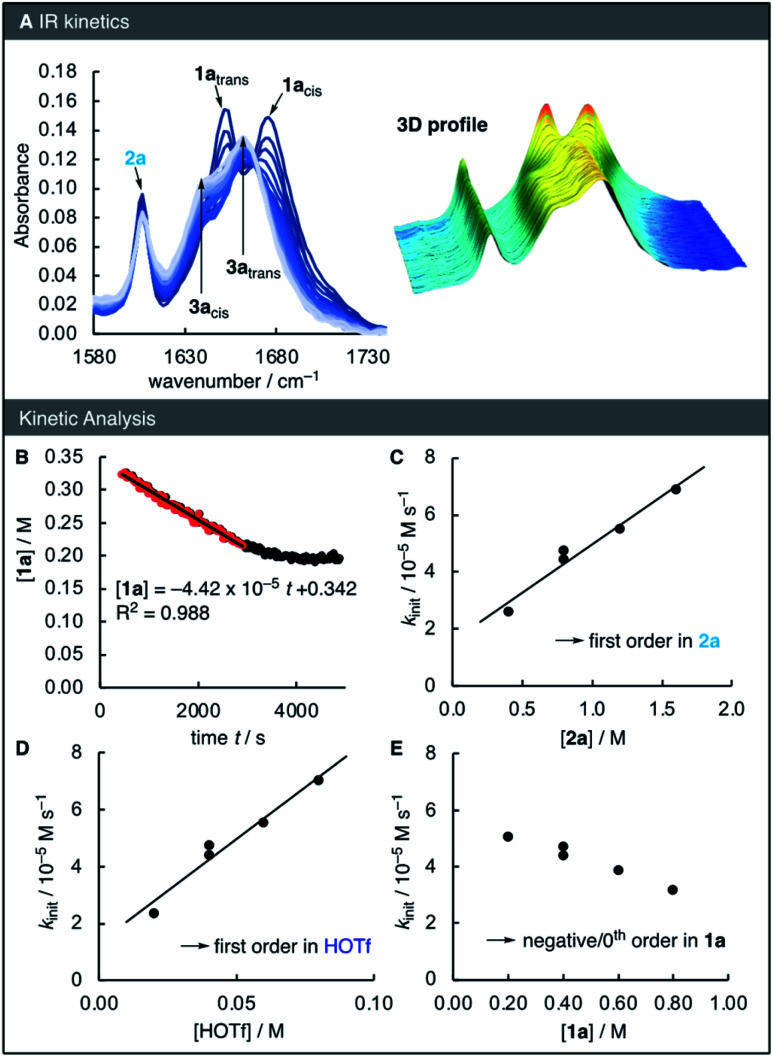
(A) *In situ* IR spectra of the reaction of 1a (0.4 M) and 2a (0.8 M) in the presence of TfOH (0.04 M) at 23 °C in HFIP and (B) time-dependent concentrations of 1a. The red data points were used to for the linear regression to determine the initial rate *k*_init_. (C) Correlations of *k*_init_*vs.* [2a], (D) of *k*_init_*vs.* [HOTf] and (E) of *k*_init_*vs.* [1a] to derive reaction orders.

To elucidate the reaction orders of all species, the reaction was next analysed by *in situ* IR spectroscopy under similar conditions as those of the NMR kinetics (Fig. 2A). The linear relationship of the IR absorbance at 1652 cm^−1^ and the concentration of 1a was used to determine the time-dependent concentration of 1a during the kinetic runs. Analogous to the NMR kinetics (Fig. 1B), the initial phase of the IR kinetics was characterized by a 0^th^ order decay of the concentration of 1a, from which the initial rate *k*_init_ of the reaction was determined by means of linear regression (Fig. 2B). Next, the kinetics of the reaction of 1a with 2a catalysed by HOTf were studied at different concentrations of all three reaction partners. Correlations of the initial rates with the reactant concentrations were then used to derive the reaction orders. The linear correlation of *k*_init_ with both [2a] and [HOTf] suggests that the reaction is first-order in these two species (Fig. 2C and D). However, with increasing concentration of 1a the initial rate decreases, implying a negative reaction order in 1a (Fig. 2E).

**Fig. 3:**
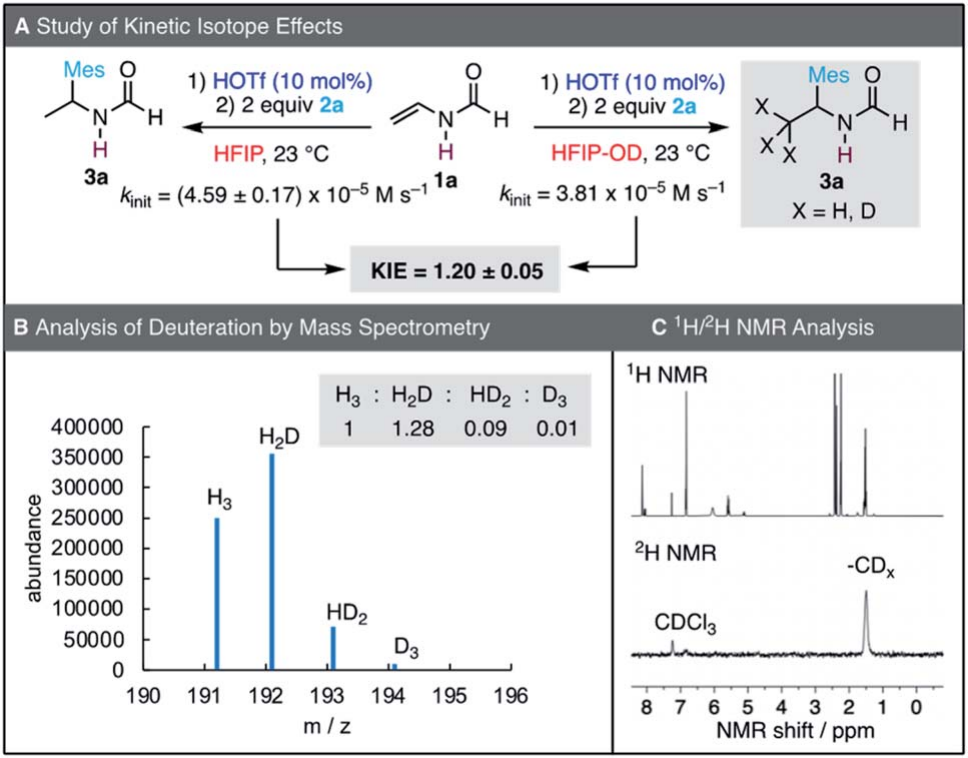
(A) Initial rates *k*_init_ from IR kinetics of the reaction of 1a (0.4 M) and 2a (0.8 M) in the presence of TfOH (0.04 M) at 23 °C in deuterated and non-deuterated HFIP. (B) Analysis of the reaction product 3a obtained in (A) in deuterated HFIP by mass spectrometry and (C) by ^1^H and ^2^H NMR.

When the reaction was performed in deuterated HFIP, a slightly reduced initial rate *k*_init_ was observed, from which a solvent KIE of 1.20 ± 0.05 was derived (Fig. 3A). As the O–D bond is in rapid exchange with the relatively small amounts of HOTf in the reaction, the active acid involved in the protonation of 1a is almost exclusively deuterated. The absence of a large primary isotope effect suggests that protonation of 1a to an iminium intermediate is not rate-determining. Analysis of the reaction product by mass spectrometry and NMR spectroscopy indicated significant deuteration of 3a. ^2^H NMR spectroscopy (Fig. 3C) was next used to verify the site of deuteration, and deuterium incorporation was found to occur almost exclusively at the newly formed CH_3_ group, in line with the mechanism shown in Fig. 1A. Notably, analysis by mass spectrometry showed that multiple isotopologues of 3a were formed, including di- and tri-deuterated 3a (Fig. 3B). Accordingly, the initial protonation of 1a to give the iminium intermediate 1a-H^+^ can be considered reversible and relatively fast with respect to the subsequent nucleophilic addition of either HFIP (to yield 1-HFIP) or 2a (to yield 3a).

While we were unable to directly observe the iminium intermediate 1a-H^+^, the results of the kinetics and deuteration experiments provide compelling evidence for the mechanism of the reaction: after a fast pre-equilibrium to yield an iminium ion, slow subsequent nucleophilic addition occurs. Such pre-equilibrium conditions followed by a slower subsequent reaction are typical conditions under which (pseudo)-0^th^ order kinetics have previously been observed,^20^ in line with our experiments. While the mechanism in Fig. 1A explains the apparent first order in both acid and nucleophile, one would expect a 0^th^ order dependency in 1a. The small negative reaction order indicated by Fig. 2E might therefore be caused by small amounts of basic impurities in 1a which quench the acid catalyst, thereby masking a potential 0^th^ order in 1a.

### DFT computations

Lastly, DFT computations at the SMD(HFIP)/MN15/def2-TZVP level of theory were performed to investigate the nature of the rate-determining step, namely the addition of either HFIP or mesitylene 2a to the iminium intermediate.^21^ The iminium intermediate 1a-H^+^ was chosen as a starting point for the computations since the energetics for the protonation of 1a are not meaningfully computable. This choice was made because the true nature of the effective acid in our reaction (protonated HFIP, TfOH, H_3_O^+^ depending on the content of H_2_O, *etc.*) is difficult to pinpoint. Additionally, a correct description of proton transfer steps is heavily dependent on the solvation model used and is not well-described by implicit solvation.

**Fig. 4:**
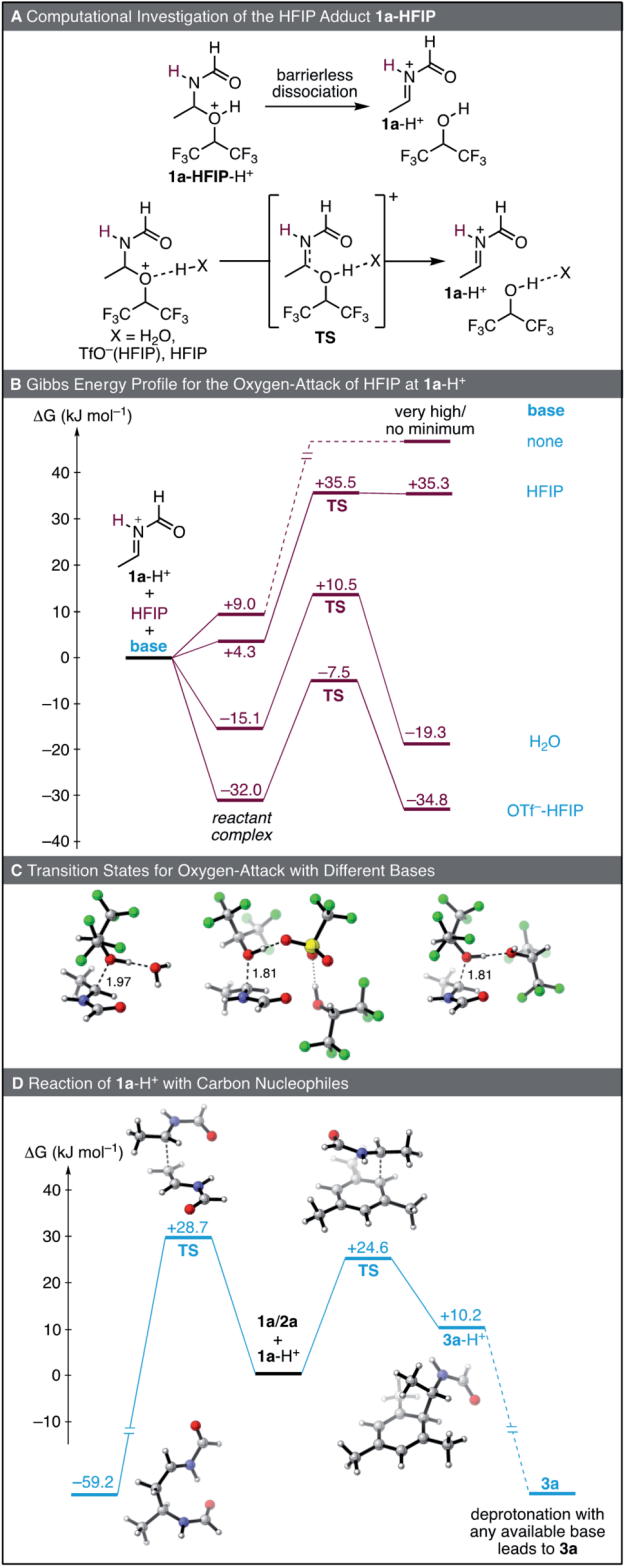
Computational investigations of oxygen- and carbon-attack of 1a-H^+^ with HFIP and 1a/2a at the SMD(HFIP)/MN15/def2-TZVP level of theory. All depicted 3D structures correspond to the lowest-energy conformers for each point.

We next investigated the formation of the HFIP adduct 1a-HFIP. When trying to optimize the structure of 1a-HFIP-H^+^, which is the adduct of 1a-H^+^ with HFIP, dissociation into the reactants occurred indicating a high endergonicity of this species (Fig. 4A). Consequently, no transition state could be localized for the direct formation of 1-HFIP-H^+^. The reason for this observation is likely due to the insufficient description of the solvent by the implicit solvation model. Accordingly, the inclusion of an explicitly coordinated base, *e.g.* an additional molecule of HFIP, TfO^−^ or H_2_O, stabilized both the transition state and the adduct 1-HFIP-H^+^ by allowing a proton-transfer, thereby enabling their computational investigation (Fig. 4B and C).

Depending on the basicity of the base explicitly added, both the activation barrier for the oxygen-attack as well as the Gibbs free energy of the reaction change (Fig. 4B and C). Additionally, in the case of H_2_O and TfO^−^, energetically favourable reactant complexes were observed, which, in the case of TfO^−^, corresponds to an ion pair. The highest barrier for the oxygen-attack of HFIP at the iminium ion 1a-H^+^ was obtained when HFIP itself acted as a base for the proton-transfer. Significantly lower barriers were observed with molecules of H_2_O or TfO^−^ (solvated by an explicit molecule of HFIP) acting as base.

Experimentally, formation of the adduct 1a-HFIP was found to be reversible. However, this could not be computationally studied in a straightforward way due to the significant amount of HOTf present in the reaction (10 mol%): While the reactions of the ion pair of 1a-H^+^ with TfO^−^/HFIP or with H_2_O as base are only slightly exergonic (−2.8 and −4.2 kJ mol^−1^, respectively) and thus reversible, the computed energy value for the reaction of 1a + HFIP to yield 1a-HFIP is significantly more negative (−47.4 kJ mol^−1^). Accordingly, the experimental reality lies somewhere between those two values.

Next, we investigated the barrier for the reaction of 1a-H^+^ with both mesitylene (2a) and 1a, the latter reaction being the starting point for the undesired polymerization of 1a in HFIP (Fig. 4D). In agreement with our experimental findings indicating little polymerization, 1a-H^+^ reacts *via* a 4.1 kJ mol^−1^ lower barrier with mesitylene (2a) than with 1a. Fast proton transfer of the resulting Wheland-complex to any available base (*e.g.* HFIP, TfO^−^, 1a, H_2_O, *etc.*) yields the final reaction product 3a, thereby restoring the catalytic cycle. The computed activation barrier for the reaction of 1a-H^+^ with 2a (+24.6 mol^−1^) is in a similar range as that for oxygen-attack of HFIP (+24.5 kJ mol^−1^ with TfO^−^/HFIP or +25.6 kJ mol^−1^ with H_2_O), thus being in line with the experimental observation of both reactions happening concurrently at the beginning of the kinetics (Fig. 1B).

**Fig. 5:**
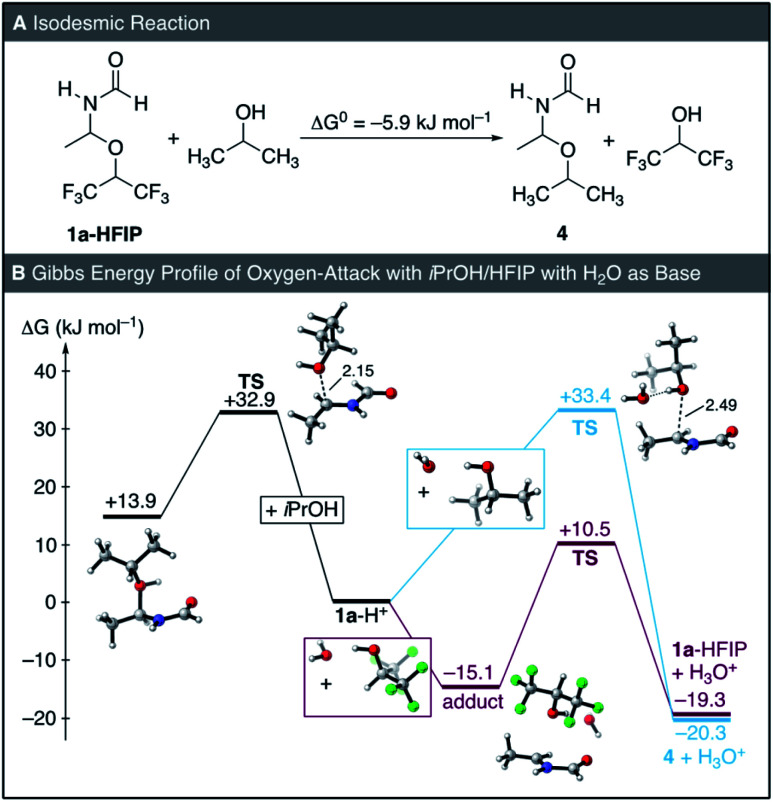
(A) Isodesmic reaction at MN15/def2-TZVP level of theory in gas phase. (B) Gibbs energy profile for the reaction of 1a-H^+^ with iPrOH and HFIP at the SMD(HFIP or iPrOH)/MN15/def2-TZVP level of theory. All depicted 3D structures correspond to the lowest-energy conformers for each point.

Finally, we computationally compared the solvent HFIP to its non-fluorinated parent iPrOH. In iPrOH, only the formation of the ether 4 was observed experimentally, indicating that formation of 4 is either more exergonic than that of 1a-HFIP in HFIP or that the nucleofugality of the iPrOH is higher than that of HFIP, thus, the product is kinetically stabilized. As discussed above, the highly acidic nature of our reaction medium makes comparisons of the overall energetics of the reaction challenging. Additionally, comparisons of the energetics in different solvents would require an accurate computational description of the solvent, which is not necessarily the case. However, HFIP is not parametrized within most computational software packages and has to be manually defined (see the ESI†). Thus, we compared the relative energetics of 4 and 1a-HFIP by means of an isodesmic reaction computed in the gas-phase (Fig. 5A). This analysis indicated that the stability of 4 is only slightly higher than that of 1a-HFIP by 5.9 kJ mol^−1^.

In contrast to the reaction with HFIP (Fig. 4A), a transition state for the direct addition of iPrOH at 1a-H^+^ could be located and the protonated ether 4-H^+^ is stable toward dissociation (Fig. 5B, left). This observation is in line with the higher Brønsted basicity of iPrOH compared to HFIP. To be able to directly compare the oxygen-attack of iPrOH to that of HFIP in solution, we additionally investigated the reaction of 1a-H^+^ with iPrOH coordinated to an explicit molecule of water as a model base (Fig. 5B, right; *cf.*Fig. 4B for HFIP). Inclusion of the additional water molecule did not alter the activation barrier for addition of iPrOH but resulted in the reaction being thermodynamically more favourable. Compared to the oxygen-attack of HFIP, the most striking difference in the pathway with iPrOH is the different barrier for the reverse reaction. While the barrier for the reverse reaction of 1a-HFIP is computed at 29.8 kJ mol^−1^, the value is 53.7 kJ mol^−1^ for 4. While our computations are at best qualitative, they suggest that one of the main differences in the reactivities of iPrOH and HFIP is the higher nucleofugality of the latter, which renders solvent-trapping reversible.

In contrast to many reports that only hypothesized about the precise nature by which HFIP promotes reactions in Lewis and Brønsted acid catalysis,^10*e*^ our mechanistic studies reveal how HFIP enables the addition of arenes to the key iminium intermediate formed by reversible protonation of the enamide precursor. Authors typically claimed that HFIP stabilizes cationic intermediates due to its low nucleophilicity, relatively high dielectric constant, and H-bond network. However, these factors are not of relevance in our study as HFIP does not stabilize the iminium intermediates, but rather reacts with them reversibly to generate HFIP ethers, thereby precluding the oligomerization of the substrates observed in other solvents. As the electrophilicities of the iminium ions formed in this study are expected to be lower than many of the cationic intermediates previously investigated in HFIP,^10*e*^ mechanisms involving reversible cation trapping are likely of much more general importance in reactions performed in HFIP than previously appreciated.

## Conclusions

In conclusion, we have developed a simple protocol for the hydroarylation of enamides which provides access to a diverse array of synthetically useful *N*-benzyl amides. The present method is another addition to the family of HFIP-enabled reactions. A wide scope of (hetero)arenes was displayed and a broad range of functional groups on the enamide was tolerated. All the reactions reported herein proceed *via* the use of readily available precursors without requiring any pre-activation step and produce no stoichiometric waste. Mechanistic studies show that, after initial protonation of the enamide, an iminium intermediate is formed which reacts with both HFIP and the (hetero)arene nucleophile to give both the product and the ether adduct. Subsequent heterolysis of the latter leads to full conversion to the target products in a slower subsequent step. This demonstrates that the central role of HFIP is to trap the highly reactive iminium intermediate to generate a reservoir species for the hydroarylation, thus preventing undesired side reactions. A similar reservoir effect is likely operative in many other reactions involving carbocationic intermediates in HFIP.

## Data availability

All experimental procedures, characterisation data, mechanistic investigations and NMR spectra for all new compounds can be found in the ESI.†

## Author contributions

Conceptualization, D. L.; Methodology, D. L. and J. M.; Investigation, N. Z., S. B. and R. J. M.; Writing – Original Draft, N. Z., S. B. and R. J. M.; Writing – Review & Editing, D. L. and J. M.; Funding Acquisition, J. M.; Supervision, D. L. and J. M.

## Conflicts of interest

There are no conflicts to declare.

## Supplementary Material

SC-013-D2SC02012B-s001
