# Supplementary material for: Hydroarylation of enamides enabled by HFIP via a hexafluoroisopropyl ether as iminium reservoir
Source: Chem Sci. 2022 Jun 27;13(28):8436–43. doi: 10.1039/d2sc02012b (PMC9297520; doi:10.1039/d2sc02012b)
Supplement: SC-013-D2SC02012B-s001 [file SC-013-D2SC02012B-s001.pdf]

Electronic Supplementary Material (ESI) for Chemical Science.
This journal is © The Royal Society of Chemistry 2022

Hydroarylation of Enamides Enabled by Hexafluoroisopropanol via a Hemiaminal as Iminium Reservoir

Nicolas Zeidan,[1] Sergiu Bicic,[1] Robert J. Mayer,[1] David Lebœuf* and Joseph Moran*

[1]Contributed equally

Institut de Science et d'Ingénierie Supramoléculaires (ISIS), CNRS UMR 7006, Université de Strasbourg, 8 allée Gaspard Monge, 67000 Strasbourg, France.

dleboeuf@unistra.fr

moran@unistra.fr

Table of Contents

I. GENERAL REMARKS **2**

II. HYDROARYLATION OF ENAMIDES **3**

II.1 General Procedure for the Hydroarylation of Enamides by (Hetero)arenes 3
II.2 Characterization Data of *N*-Benzyl Amides (Scheme 2) 3
II.3 Characterization Data of Derivatized Compounds (Equations 1 and 2) 14
II.4 Characterization Data of Hemiaminal 1-HFIP (Figure 1C) 15

III. NMR SPECTRA **16**

IV. MECHANISTIC STUDIES **50**

IV.1 Quantification of the Rotational Barrier of 1a (Figure 1A) 50
IV.2 NMR Kinetics (Figure 1B) 52
IV.3 IR Kinetics (Figure 2) 54
IV.4 Computations (Figure 4) 62
IV.5 Geometries in HFIP 69
IV.5 Geometries in Gas Phase 112
IV.6 Geometries in *i*PrOH 120

V. REFERENCES **129**

I. General Remarks

Materials: All commercial materials were purchased from Sigma-Aldrich, TCI and FluoroChem, and were used as received, without further purification. Triflic acid (TfOH) *ReagentPlus*®, ≥99% (CAS: 1493-13-6) and Trifluoromethanesulfonimide ($HNTf_2$), ≥95% (CAS: 82113-65-3) were purchased from Sigma Aldrich, and HFIP (CAS: 920-66-1) from FluoroChem. The other starting starting materials were prepared according to known protocols.

Reactions wert monitored by thin layer chromatography (TLC) performed on aluminum plates coated with silica gel F_{254} with 0.2 mm thickness. Chromatograms were visualized by fluorescence quenching with UV light at 254 nm and/or by staining using *p*-anisaldehyde. Flash column chromatography (FC) was performed using silica gel 60 (230-400 mesh, Merck and co.). Yields refer to chromatographically and spectroscopically pure compounds.

^{1}H NMR, ^{13}C NMR, and ^{19}F NMR spectra were recorded using a Bruker UltraShield 400, 500 or 700 at 300K. ^{1}H NMR chemical shifts are reported in ppm using residual solvent peak as reference ($CDCl_3$: δ = 7.26 ppm or DMSO-d_6: δ = 2.50 ppm). Data for ^{1}H NMR are presented as follows: chemical shift δ (ppm), multiplicity (s = singlet, d = doublet, t = triplet, m = multiplet, br = broad), coupling constant *J* (Hz) and integration. ^{13}C NMR spectra were recorded at 100, 125 or 150 MHz using broadband proton decoupling and chemical shifts are reported in ppm using residual solvent peaks as reference ($CDCl_3$: δ = 77.16 ppm or DMSO-d_6: δ = 39.52 ppm). Multiplicity was defined by recorded a ^{13}C NMR spectra using the attached proton test (APT). ^{19}F NMR spectra were recorded at 376.5 or 471 MHz at ambient temperature. High-resolution mass spectrometry (HRMS) analysis was performed on instruments GCT 1er Waters (EI and IC), MicroTOF-Q Bruker (ESI) and a GC Thermo Scientific Trace 1300 GC unit coupled to an APPI MasCom source mounted on a Thermo Scientific Exactive Plus EMR mass unit (Orbitrap FT-HRMS analyzer).

II. Hydroarylation of Enamides

II.1 General Procedure for the Hydroarylation of Enamides by (Hetero)arenes

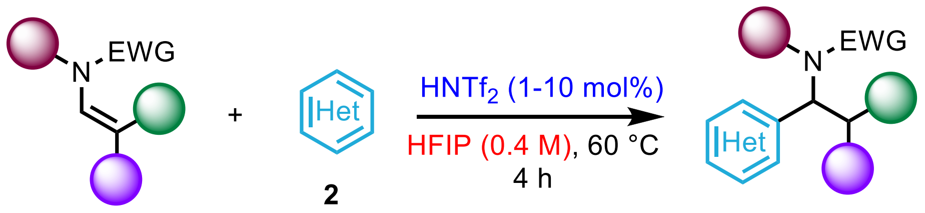

1 2 3

Enamide (0.40 mmol, 1.0 equiv.) and (hetero)arene (5.0 equiv.) were charged (in air) in a 10 mL screw-cap vial equipped with a Teflon-coated magnetic stir. A stock solution of trifluoromethanesulfonimide (1 or 10 mol%) in HFIP (1 mL, 0.4 M) was added, and the glass tube was sealed. The reaction mixture was stirred at 60 °C for the indicated time (4-18 h). Upon completion, the reaction mixture was quenched with a saturated solution of $NaHCO_3$, filtered through a short plug of silica, and concentrated under reduced pressure. The crude reaction mixture was purified by FC over silica gel to furnish the target products **3**.

II.2 Characterization Data of *N*-Benzyl Amides (Scheme 2)

If the ratio between rotamers is superior to 3:1, only the characterization of the major rotamer is described. However, the shifts and multiplicity of the minor rotamer is shown, when possible, in the NMR spectra for reference.

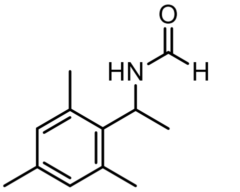

Chemical Formula: $C_{12}H_{17}NO$
Exact Mass: 191.1310

***N*-(1-mesitylethyl)formamide (3a) –** General procedure **II.1** was followed, using 10 mol% catalyst loading. The product was purified by flash column chromatography over silica gel using pentane–EtOAc (20 to 80%) as the mobile phase and was isolated as a white solid (76.5 mg, 0.400 mmol, >99%, major:minor 81:19, mp = 91–93 °C). **^{1}H NMR** (500 MHz, $CDCl_3$) δ 8.13 (t, *J* = 1.3 Hz, 1H), 6.83 (s, 2H), 6.11 (s, 1H), 5.58 (p, *J* = 7.3 Hz, 1H), 2.42 (s, 6H), 2.24 (s, 3H), 1.52 (d, *J* = 7.2 Hz, 3H). **^{13}C NMR** (126 MHz, $CDCl_3$) δ 160.3, 136.6, 135.5, 135.3, 130.3, 44.4, 20.9, 20.7, 19.9. **HRMS** (ESI) *m/z* calcd. for $C_{12}H_{18}NO$ [M+H] 192.1383, found 192.1391.

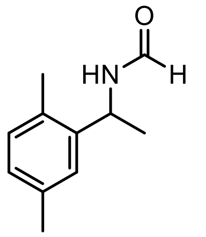

Chemical Formula: $C_{11}H_{15}NO$
Exact Mass: 177.1154

***N*-(1-(2,5-dimethylphenyl)ethyl)formamide (3b)** – General procedure **II.1** was followed, using 10 mol% catalyst loading. The product was purified by flash column chromatography over silica gel using pentane–EtOAc (20 to 80%) as the mobile phase and was isolated as a white solid (34.0 mg, 0.192 mmol, 48%, major:minor 84:16, mp = 82–85 °C). **^{1}H NMR** (500 MHz, $CDCl_3$) δ 8.14 (s, 1H), 7.13–6.97 (m, 3H), 5.68 (s, 1H), 5.37 (p, *J* = 7.1 Hz, 1H), 2.33 (s, 3H), 2.32 (s, 3H), 1.50 (d, *J* = 6.8 Hz, 3H). **^{13}C NMR** (126 MHz, $CDCl_3$) δ 160.1, 140.3, 136.0, 132.8, 130.9, 128.4, 125.5, 44.3, 21.3, 21.3, 18.8. **HRMS** (ESI) *m/z* calcd. for $C_{11}H_{16}NO$ $[M+H]^+$ 178.1227, found 178.1233.

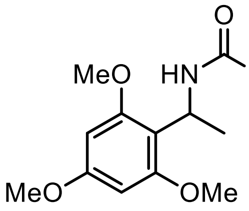

Chemical Formula: $C_{12}H_{17}NO_4$
Exact Mass: 239.1158

***N*-(1-(2,4,6-trimethoxyphenyl)ethyl)formamide (3c)** – General procedure **II.1** was followed, using 10 mol% catalyst loading. The product was purified by flash column chromatography over silica gel using pentane–EtOAc (20 to 80%) as the mobile phase and was isolated as a white solid (81.4 mg, 0.340 mmol, 85%, major:minor 67:33, mp = 94–97 °C). *Major rotamer:* **^{1}H NMR** (500 MHz, $CDCl_3$) δ 8.09 (dd, *J* = 1.8, 0.9 Hz, 1H), 6.97 (d, *J* = 9.6 Hz, 1H), 6.13 (s, 2H), 5.91–5.81 (m, 1H), 3.84 (s, 6H), 3.80 (s, 3H), 1.37 (d, *J* = 6.9 Hz, 3H). **^{13}C NMR** (126 MHz, $CDCl_3$) δ 160.4, 160.1, 158.5, 111.2, 91.1, 55.9, 55.5, 39.0, 21.2. *Minor rotamer:* **^{1}H NMR** (500 MHz, $CDCl_3$) δ 8.14 (d, *J* = 12.2 Hz, 1H), 6.82 (t, *J* = 11.3 Hz, 1H), 6.12 (s, 2H), 5.20 (dq, *J* = 10.4, 6.9 Hz, 1H), 3.82 (s, 6H), 3.80 (s, 3H), 1.44 (d, *J* = 7.0 Hz, 3H). **^{13}C NMR** (126 MHz, $CDCl_3$) δ 163.9, 160.6, 157.9, 111.2, 90.9, 55.8, 55.5, 43.6, 22.5. **HRMS** (ESI) *m/z* calcd. for $C_{12}H_{18}NO_4$ $[M+H]^+$ 240.1231, found 240.1238.

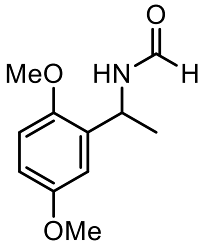

Chemical Formula: $C_{11}H_{15}NO_3$
Exact Mass: 209.1052

***N*-(1-(2,5-dimethoxyphenyl)ethyl)formamide (3d)** – General procedure **II.1** was followed, using 10 mol% catalyst loading. The product was purified by flash column chromatography over silica gel using pentane–EtOAc (20 to 80%) as the mobile phase and was isolated as a white solid (79.5 mg, 0.380 mmol, 95%, major:minor 76:24, mp = 81–84 °C). **^{1}H NMR** (500 MHz, $CDCl_3$) δ 8.13 (t, *J* = 1.2 Hz, 1H), 6.85–6.73 (m, 3H), 6.61 (d, *J* = 8.5 Hz, 1H), 5.29 (dq, *J* = 8.9, 6.9 Hz, 1H), 3.84 (s, 3H), 3.75 (s, 3H), 1.46 (d, *J* = 6.9 Hz, 3H). **^{13}C NMR** (126 MHz, $CDCl_3$) δ 160.2, 153.8, 151.2, 131.4, 114.6, 112.7, 112.1, 55.9, 55.8, 46.4, 21.6. **HRMS** (ESI) *m/z* calcd. for $C_{11}H_{16}NO_3$ $[M+H]^+$ 210.1125, found 210.1119.

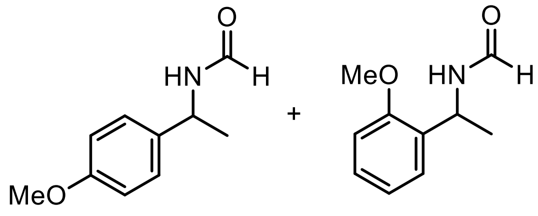

Chemical Formula: $C_{10}H_{13}NO_2$
Exact Mass: 179.0946

***N*-(1-(4-methoxyphenyl)ethyl)formamide (3e) + *N*-(1-(2-methoxyphenyl)ethyl) formamide (3'e) –** General procedure **II.1** was followed, using 10 mol% catalyst loading. The product was purified by flash column chromatography over silica gel using pentane–EtOAc (20 to 80%) as the mobile phase and was isolated as a colorless oil (53.0 mg, 0.296 mmol, 74%, *o/p* 63:37).

3e (major:minor 78:22): ^{1}**H NMR** (500 MHz, $CDCl_3$) δ 8.09 (s, 1H), 7.24 (d, *J* = 8.8 Hz, 2H), 6.86 (d, *J* = 8.8 Hz, 2H), 6.25 (d, *J* = 7.7 Hz, 1H), 5.13 (p, *J* = 7.1 Hz, 1H), 3.78 (s, 3H), 1.47 (d, *J* = 6.9, 3H). ^{13}**C NMR** (126 MHz, $CDCl_3$) δ 160.7, 159.0, 134.9, 127.5, 114.2, 55.5, 47.2, 21.8.

3'e (major:minor 70:30): ^{1}**H NMR** (500 MHz, $CDCl_3$) δ 8.14 (s, 1H), 7.22 (d, J = 5 Hz, 2H), 6.96-6.89 (m, 2H), 6.54 (br s, 1H), 5.38-5.32 (m, 1H), 3.89 (s, 3H) 1.49 (d, J = 5 Hz, 3H). ^{13}**C NMR** (126 MHz, $CDCl_3$) δ 160.5, 157.1, 128.8, 128.1, 127.2, 121.1, 111.2, 55.5, 46.2, 21.6.

HRMS (ESI) *m/z* calcd. for $C_{10}H_{14}NO_2$ $[M+H]^+$ 180.1019, found 180.1015.

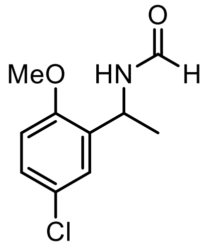

Chemical Formula: $C_{10}H_{12}ClNO_2$
Exact Mass: 213.0557

***N*-(1-(5-chloro-2-methoxyphenyl)ethyl)formamide (3f) –** General procedure **II.1** was followed, using 10 mol% catalyst loading. The product was purified by flash column chromatography over silica gel using pentane–EtOAc (20 to 80%) as the mobile phase and was isolated as a white solid (41.1 mg, 0.192 mmol, 48%, major:minor 78:22, mp = 91–95 °C). ^{1}**H NMR** (400 MHz, $CDCl_3$) δ 8.14 (s, 1H), 7.26–7.13 (m, 2H), 6.81 (d, *J* = 8.2 Hz, 1H), 6.35 (s, 1H), 5.38–5.26 (m, 1H), 3.86 (s, 3H), 1.45 (d, *J* = 6.9 Hz, 3H). ^{13}**C NMR** (101 MHz, $CDCl_3$) δ 160.2, 155.7, 132.3, 128.3, 127.9, 125.9, 112.4, 55.8, 45.5, 21.4. **HRMS** (ESI) *m/z* calcd. for $C_{10}H_{13}ClNO_2$ $[M+H]^+$ 214.0629, found 214.0625.

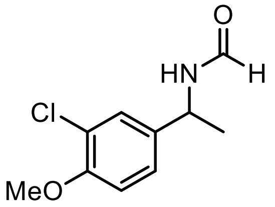

Chemical Formula: $C_{10}H_{12}ClNO_2$
Exact Mass: 213.0557

***N*-(1-(3-chloro-4-methoxyphenyl)ethyl)formamide (3g)** – General procedure **II.1** was followed, using 10 mol% catalyst loading. The product was purified by flash column chromatography over silica gel using pentane–EtOAc (20 to 80%) as the mobile phase and was isolated as a colorless oil (51.3 mg, 0.24 mmol, major:minor 85:15, 60%). ^{1}**H NMR** (400 MHz, $CDCl_3$) δ 8.15 (s, 1H), 7.32 (d, *J* = 2.3 Hz, 1H), 7.18 (dd, *J* = 8.5, 2.3 Hz, 1H), 6.88 (d, *J* = 8.5 Hz, 1H), 5.85 (s, 1H), 5.14 (p, *J* = 7.1 Hz, 1H), 3.88 (s, 3H), 1.49 (d, *J* = 6.9 Hz, 3H). ^{13}**C NMR** (101 MHz, $CDCl_3$) δ

160.4, 154.5, 135.9, 128.1, 125.9, 122.7, 112.3, 56.4, 46.8, 21.7. **HRMS** (ESI) *m/z* calcd. for $C_{10}H_{13}NO_2Cl$ $[M+H]^+$ 214.0629, found 214.0626.

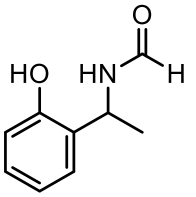

Chemical Formula: $C_9H_{11}NO_2$
Exact Mass: 165.0790

***N*-(1-(2-hydroxyphenyl)ethyl)formamide (3'h)** – General procedure **II.1** was followed, using 10 mol% catalyst loading. The product was purified by flash column chromatography over silica gel using pentane–EtOAc (20 to 80%) as the mobile phase and was the first of two regioisomers isolated as a colorless oil (26.4 mg, 0.160 mmol, major:minor 88:12, 40%). **^{1}H NMR** (400 MHz, DMSO-d6) δ 9.50 (s, 1H), 8.40 (d, *J* = 8.5 Hz, 1H), 8.04–7.90 (m, 1H), 7.17 (dd, *J* = 7.6, 1.7 Hz, 1H), 7.04 (td, *J* = 7.7, 1.7 Hz, 1H), 6.82–6.72 (m, 2H), 5.22 (p, *J* = 7.1 Hz, 1H), 1.29 (d, *J* = 6.9 Hz, 3H). **^{13}C NMR** (101 MHz, DMSO-d6) δ 160.0, 154.0, 130.2, 127.6, 126.0, 118.9, 115.2, 41.8, 21.4. **HRMS** (ESI) *m/z* calcd. for $C_9H_{12}NO_2$ $[M+H]^+$ 166.0863, found 166.0859.

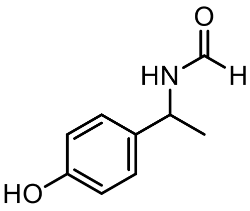

Chemical Formula: $C_9H_{11}NO_2$
Exact Mass: 165.0790

***N*-(1-(4-hydroxyphenyl)ethyl)formamide (3h)** – General procedure **II.1** was followed, using 10 mol% catalyst loading. The product was purified by flash column chromatography over silica gel using pentane–EtOAc (20 to 80%) as the mobile phase and was the second of two regioisomers isolated as an amorphous solid (35.7 mg, 0.216 mmol, major:minor 88:12, 54%). **^{1}H NMR** (400 MHz, DMSO-d6) δ 9.27 (s, 1H), 8.37 (d, *J* = 8.3 Hz, 1H), 7.98 (dd, *J* = 1.9, 1.0 Hz, 1H), 7.10 (d, *J* = 8.5 Hz, 2H), 6.70 (d, *J* = 8.5 Hz, 2H), 4.89 (p, *J* = 7.2 Hz, 1H), 1.31 (d, *J* = 7.0 Hz, 3H). **^{13}C NMR** (101 MHz, DMSO-d6) δ 159.9, 156.2, 134.3, 127.1, 115.0, 45.9, 22.4. **HRMS** (ESI) *m/z* calcd. for $C_9H_{11}NO_2Na$ $[M+Na]^+$ 188.0682, found 188.0677.

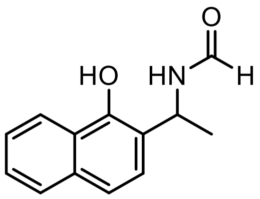

Chemical Formula: $C_{13}H_{13}NO_2$
Exact Mass: 215.0946

***N*-(1-(1-hydroxynaphthalen-2-yl)ethyl)formamide (3'i) –** General procedure **II.1** was followed, using 10 mol% catalyst loading. The product was purified by flash column chromatography over silica gel using pentane–EtOAc (20 to 80%) as the mobile phase and was the first of two regioisomers isolated as a yellow oil (19.1 mg, 0.089 mmol, major:minor 95:5, 22%). **^{1}H NMR** (500 MHz, DMSO-d6) δ 9.71 (d, *J* = 1.8 Hz, 1H), 8.83 (d, *J* = 8.0 Hz, 1H), 8.31–8.24 (m, 1H), 8.08 (t, *J* = 1.5 Hz, 1H), 7.89–7.82 (m, 1H), 7.58–7.41 (m, 4H), 5.66–5.44 (m, 1H), 1.48 (d, *J* = 6.8 Hz, 3H). **^{13}C NMR** (126 MHz, DMSO-d6) δ 161.4, 149.2, 133.8, 127.9, 126.2, 125.9, 125.4, 125.2, 124.5, 122.6, 120.0, 41.9, 21.4. **HRMS** (ESI) *m/z* calcd. for $C_{13}H_{13}NO_2Na$ $[M+Na]^+$ 238.0838, found 238.0833.

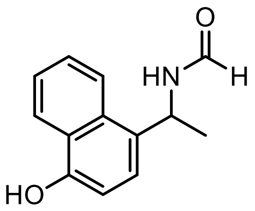

Chemical Formula: $C_{13}H_{13}NO_2$
Exact Mass: 215.0946

***N*-(1-(4-hydroxynaphthalen-1-yl)ethyl)formamide (3i) –** General procedure **II.1** was followed, using 10 mol% catalyst loading. The product was purified by flash column chromatography over silica gel using pentane–EtOAc (20 to 80%) as the mobile phase and was the second of two regioisomers isolated as an off-white solid (62.8 mg, 0.292 mmol, 73%, major:minor 91:9, mp = 174–176 °C). **^{1}H NMR** (500 MHz, DMSO-d6) δ 10.06 (s, 1H), 8.54–8.41 (m, 1H), 8.19 (dd, *J* = 8.3, 1.5 Hz, 1H), 8.01 (d, *J* = 1.4 Hz, 1H), 7.98 (d, *J* = 8.5 Hz, 1H), 7.52 (ddd, *J* = 8.5, 6.8, 1.5 Hz, 1H), 7.45 (ddd, *J* = 8.1, 6.8, 1.2 Hz, 1H), 7.34 (d, *J* = 7.9 Hz, 1H), 6.85 (d, *J* = 7.9 Hz, 1H), 5.67 (p, *J* = 7.0 Hz, 1H), 1.49 (d, *J* = 6.9 Hz, 3H). **^{13}C NMR** (126 MHz, DMSO-d6) δ 160.2, 153.1, 132.1, 129.7, 126.8, 125.3, 124.7, 123.7, 123.4, 123.1, 107.6, 42.6, 21.8. **HRMS** (ESI) *m/z* calcd. for $C_{13}H_{13}NO_2Na$ $[M+Na]^+$ 238.0838, found 238.0834.

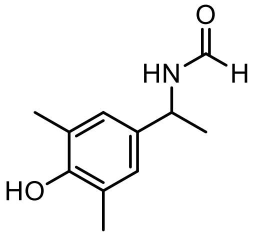

Chemical Formula: $C_{11}H_{15}NO_2$
Exact Mass: 193.1103

***N*-(1-(4-hydroxy-3,5-dimethylphenyl)ethyl)formamide (3j) –** General procedure **II.1** was followed, using 1 mol% catalyst loading. The product was purified by flash column chromatography over silica gel using pentane–EtOAc (20 to 80%) as the mobile phase and was isolated as a white solid (77.3 mg, 0.400 mmol, >99%, major:minor 54:46, mp = 146–148 °C). **^{1}H NMR** (400 MHz, $CDCl_3$) *Major Rotamer:* δ 8.09 (d, *J* = 2.1 Hz, 1H), 7.57–7.44 (m, 1H), 7.36 (s, 1H), 6.55 (s, 1H), 6.51 (d, *J* = 1.6 Hz, 1H), 5.60 (dq, *J* = 9.7, 7.0 Hz, 1H), 2.35 (s, 3H), 2.20 (s, 3H), 1.49 (d, *J* = 6.9 Hz, 3H). *Minor Rotamer:* δ 9.69 (s, 1H), 8.18 (d, *J* = 12.6 Hz, 1H), 8.03 (t, *J* = 11.2 Hz, 1H), 6.60 (d, *J* = 1.7 Hz, 1H), 6.52 (s, 1H), 4.86 (dq, *J* = 9.9, 6.9 Hz, 1H), 2.29 (s, 3H), 2.20 (s, 3H), 1.56 (d, *J* = 6.9 Hz, 3H). **^{13}C NMR** (101 MHz, $CDCl_3$) δ 164.7, 160.7, 154.7, 154.5, 138.5, 138.0, 136.4, 134.7, 123.9, 123.8, 123.1, 123.0, 115.2, 114.9, 49.0, 42.8, 22.3, 21.0 (2C), 20.4, 20.0, 19.8. **HRMS** (ESI) *m/z* calcd. for $C_{11}H_{16}NO_2$ $[M+H]^+$ 194.1176, found 194.1172.

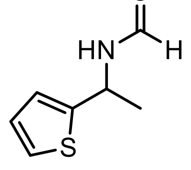

Chemical Formula: C_7H_9NOS
Exact Mass: 155.0405

***N*-(1-(thiophen-2-yl)ethyl)formamide (3k) –** General procedure **II.1** was followed, using 1 mol% catalyst loading. The product was purified by flash column chromatography over silica gel using pentane–EtOAc (20 to 80%) as the mobile phase and was isolated as a colorless oil (55.9 mg, 0.360 mmol, major:minor 85:15, 90%). **^{1}H NMR** (400 MHz, $CDCl_3$) δ 8.11 (s, 1H), 7.19 (dd, *J* = 5.0, 1.3 Hz,

1H), 6.99–6.91 (m, 2H), 6.27 (s, 1H), 5.53–5.37 (m, 1H), 1.58 (d, *J* = 6.9 Hz, 3H). **^{13}C NMR** (101 MHz, $CDCl_3$) δ 160.2, 146.4, 126.9, 124.5, 124.2, 43.3, 22.2. **HRMS** (ESI) *m/z* calcd. for $C_7H_{10}NOS$ [M+H]$^+$ 156.0478, found 156.0474.

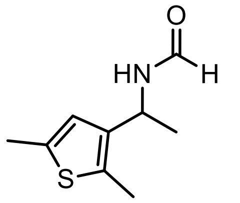

Chemical Formula: $C_9H_{13}NOS$
Exact Mass: 183.0718

***N*-(1-(2,5-dimethylthiophen-3-yl)ethyl)formamide (3l) –** General procedure **II.1** was followed, using 1 mol% catalyst loading. The product was purified by flash column chromatography over silica gel using pentane–EtOAc (20 to 80%) as the mobile phase and was isolated as a colorless oil (73.3 mg, 0.400 mmol, >99%, major:minor 85:15, mp = 51–54 °C). **^{1}H NMR** (400 MHz, $CDCl_3$) δ 8.09 (s, 1H), 6.57 (d, *J* = 1.4 Hz, 1H), 5.67 (s, 1H), 5.18 (p, *J* = 7.1 Hz, 1H), 2.43–2.37 (m, 3H), 2.36 (s, 3H), 1.45 (d, *J* = 6.8 Hz, 3H). **^{13}C NMR** (101 MHz, $CDCl_3$) δ 160.0, 137.8, 136.6, 133.0, 123.1, 41.9, 21.8, 15.3, 12.9. **HRMS** (ESI) *m/z* calcd. for $C_9H_{14}NOS$ [M+H]$^+$ 184.0791, found 184.0787.

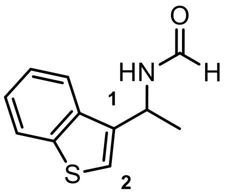

Chemical Formula: $C_{11}H_{11}NOS$
Exact Mass: 205.0561

***N*-(1-(benzo[*b*]thiophen-3-yl)ethyl)acetamide (3m) –** General procedure **II.1** was followed, using 1 mol% catalyst loading. The product was purified by flash column chromatography over silica gel using pentane–EtOAc (20 to 80%) as the mobile phase and was isolated as a colorless oil (77.2 mg, 0.376 mmol, 94%, ratio *1/2* 92:8, major:minor 87:13). **^{1}H NMR** (400 MHz, $CDCl_3$) δ 8.19 (s, 1H), 7.88–7.82 (m, 2H), 7.42–7.36 (m, 2H), 7.35 (s, 1H), 5.77–5.61 (m, 2H), 1.69 (d, *J* = 6.5 Hz, 3H). **^{13}C NMR** (101 MHz, $CDCl_3$) δ 160.2, 140.6, 137.5, 137.1, 124.8, 124.5, 122.9, 122.5, 122.2, 41.6, 20.3. **HRMS** (ESI) *m/z* calcd. for $C_{11}H_{12}NOS$ [M+H]$^+$ 206.0634, found 206.0629.

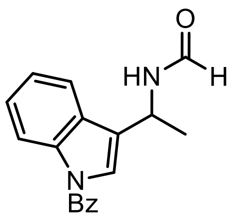

Chemical Formula: $C_{18}H_{16}N_2O_2$
Exact Mass: 292.1212

***N*-(1-(1-benzoyl-1*H*-indol-3-yl)ethyl)formamide (3n) –** General procedure **II.1** was followed, using 1 mol% catalyst loading. The product was purified by flash column chromatography over silica gel using pentane–EtOAc (20 to 80%) as the mobile phase and was isolated as a white solid (95.9 mg, 0.328 mmol, 82%, major:minor 87:13, mp = 122–125 °C). **^{1}H NMR** (400 MHz, $CDCl_3$) δ 8.33–8.28 (m, 1H), 8.21 (s, 1H), 7.75–7.69 (m, 2H), 7.67–7.60 (m, 2H), 7.55 (tt, *J* = 6.5, 1.3 Hz, 2H), 7.40 (ddd, *J* = 8.3, 7.3, 1.4 Hz, 1H), 7.33 (td, *J* = 7.5, 1.2 Hz, 1H), 7.24 (d, *J* = 1.1 Hz, 1H), 5.70 (d, *J* = 9.1 Hz, 1H), 5.59–5.48 (m, 1H), 1.60 (d, *J* = 6.8 Hz, 3H). **^{13}C NMR** (101

MHz, $CDCl_3$) δ 168.5, 160.2, 136.7, 134.4, 132.1, 129.1, 129.1, 129.0, 128.8, 125.6, 124.1, 123.4, 119.5, 116.6, 40.1, 20.2. **HRMS** (ESI) *m/z* calcd. for $C_{18}H_{16}N_2O_2Na$ $[M+Na]^+$ 315.1104, found 315.1096.

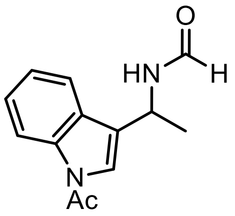

Chemical Formula: $C_{13}H_{14}N_2O_2$
Exact Mass: 230.1055

***N*-(1-(1-acetyl-1*H*-indol-3-yl)ethyl)formamide(3o) –** General procedure **II.1** was followed, using 1 mol% catalyst loading. The product was purified by flash column chromatography over silica gel using pentane–EtOAc (20 to 80%) as the mobile phase and was isolated as a white solid (62.6 mg, 0.272 mmol, 68%, major:minor 87:13, mp = 139–142 °C). **^{1}H NMR** (400 MHz, $CDCl_3$) δ 8.40 (d, *J* = 8.4 Hz, 1H), 8.23 (s, 1H), 7.61 (dd, *J* = 7.8, 1.2 Hz, 1H), 7.43–7.27 (m, 3H), 5.76 (s, 1H), 5.60–5.48 (m, 1H), 2.60 (s, 3H), 1.67 (d, *J* = 6.8 Hz, 3H). **^{13}C NMR** (101 MHz, $CDCl_3$) δ 168.6, 160.4, 136.3, 128.8, 125.9, 124.0, 124.0, 122.1, 119.6, 116.9, 40.2, 24.2, 20.3. **HRMS** (ESI) *m/z* calcd. for $C_{13}H_{15}N_2O_2$ $[M+H]^+$ 231.1128, found 231.1123.

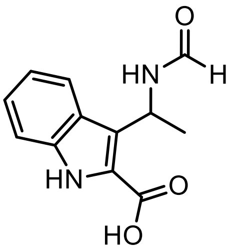

Chemical Formula: $C_{12}H_{12}N_2O_3$
Exact Mass: 232.0848

3-(1-formamidoethyl)-1*H*-indole-2-carboxylic acid (3p) – General procedure **II.1** was followed, using 1 mol% catalyst loading. The product was purified by flash column chromatography over silica gel using pentane–EtOAc (49 to 99%, with a constant 1% AcOH) as the mobile phase and was isolated as a white solid (79.9 mg, 0.344 mmol, 86%, major:minor 88:12, mp = 88–90 °C). **^{1}H NMR** (400 MHz, DMSO-d6) δ 13.18 (s, 1H), 11.51 (s, 1H), 8.69–8.52 (m, 1H), 7.99 (t, *J* = 1.4 Hz, 1H), 7.92 (d, *J* = 8.2 Hz, 1H), 7.44–7.39 (m, 1H), 7.23 (ddd, *J* = 8.1, 6.9, 1.1 Hz, 1H), 7.04 (ddd, *J* = 8.1, 6.9, 1.1 Hz, 1H), 6.06–5.86 (m, 1H), 1.48 (d, *J* = 7.0 Hz, 3H). **^{13}C NMR** (101 MHz, DMSO-d6) δ 163.2, 160.2, 136.1, 124.9, 124.3, 124.1, 123.3, 121.3, 119.3, 112.6, 21.8. *Note:* Benzylic carbon present below DMSO peak, confirmed by **HSQC** (101 MHz, 400 MHz, DMSO) δ [40.31, 5.93]. **HRMS** (ESI) *m/z* calcd. for $C_{12}H_{13}N_2O_3$ $[M+H]^+$ 233.0921, found 233.0917.

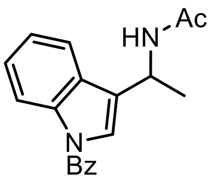

Chemical Formula: $C_{19}H_{18}N_2O_2$
Exact Mass: 306.1368

***N*-(1-(1-benzoyl-1*H*-indol-3-yl)ethyl)acetamide (3q) –** General procedure **II.1** was followed, using 1 mol% catalyst loading and allowing the reaction run for 18 h. The product was purified by flash column chromatography over silica gel using pentane–EtOAc (20 to 80%) as the mobile phase and was isolated as a white solid (88.2 mg, 0.288 mmol, 72%, major:minor > 95:5, mp = 161–164 °C). **^{1}H NMR** (400 MHz, $CDCl_3$) δ 8.30 (dt, *J* = 8.2, 0.9 Hz, 1H), 7.75–7.68 (m, 2H), 7.67–7.59 (m, 2H), 7.58–7.50 (m, 2H), 7.39 (ddd, *J* = 8.3, 7.7, 1.4 Hz, 1H), 7.33 (td, *J* = 7.3, 1.1 Hz, 1H), 7.22 (d, *J* =

1.0 Hz, 1H), 5.63 (d, *J* = 8.5 Hz, 1H), 5.44 (tt, *J* = 7.5, 6.3 Hz, 1H), 1.98 (s, 3H), 1.56 (d, *J* = 6.7 Hz, 3H). **^{13}C NMR** (101 MHz, $CDCl_3$) δ 169.3, 168.6, 136.8, 134.6, 132.2, 129.4, 129.2, 128.9, 125.6, 124.2, 124.1, 124.1, 119.8, 116.7, 41.4, 23.6, 20.3. **HRMS** (ESI) *m/z* calcd. for $C_{19}H_{19}N_2O_2$ $[M+H]^+$ 307.1441, found 307.1433.

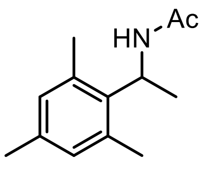

Chemical Formula: $C_{13}H_{19}NO$
Exact Mass: 205.1467

***N*-(1-mesitylethyl)acetamide (3r) –** General procedure **II.1** was followed, using 10 mol% catalyst loading. The product was purified by flash column chromatography over silica gel using pentane–EtOAc (20 to 80%) as the mobile phase and was isolated as a white solid (82.1 mg, 0.400 mmol, >99%, major:minor > 95:5, mp = 91–93 °C). ^{1}H NMR (500 MHz, $CDCl_3$) δ 6.82 (s, 2H), 6.13–5.67 (m, 1H), 5.50 (p, *J* = 7.3 Hz, 1H), 2.42 (s, 6H), 2.23 (s, 3H), 1.96 (s, 3H), 1.49 (d, *J* = 7.2 Hz, 3H). ^{13}C NMR (126 MHz, $CDCl_3$) δ 169.0, 136.5, 136.1, 135.4, 130.3, 45.6, 23.4, 20.9, 20.7, 19.9. **HRMS** (ESI) *m/z* calcd. for $C_{13}H_{20}NO$ $[M+H]^+$ 206.1539, found 206.1534.

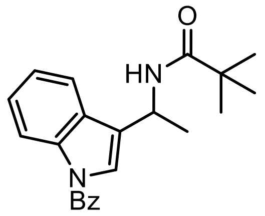

Chemical Formula: $C_{22}H_{24}N_2O_2$
Exact Mass: 348.1838

***N*-(1-(1-benzoyl-1*H*-indol-3-yl)ethyl)pivalamide (3s) –** General procedure **II.1** was followed, using 1 mol% catalyst loading and allowing the reaction run for 18 h. The product was purified by flash column chromatography over silica gel using pentane–EtOAc (20 to 80%) as the mobile phase and was isolated as a white solid (100.3 mg, 0.288 mmol, 72%, major:minor > 95:5, mp = 157–159 °C). **^{1}H NMR** (400 MHz, $CDCl_3$) δ 8.32 (dd, *J* = 8.1, 1.1 Hz, 1H), 7.78–7.70 (m, 2H), 7.67–7.49 (m, 4H), 7.39 (ddd, *J* = 8.4, 7.2, 1.3 Hz, 1H), 7.35–7.28 (m, 1H), 7.21 (d, *J* = 1.2 Hz, 1H), 5.72 (d, *J* = 8.2 Hz, 1H), 5.42 (tt, *J* = 6.9, 5.8 Hz, 1H), 1.54 (d, *J* = 6.8 Hz, 3H), 1.18 (s, 9H). **^{13}C NMR** (101 MHz, $CDCl_3$) δ 177.6, 168.6, 136.9, 134.7, 132.2, 129.5, 129.2, 128.8, 125.5, 124.5, 124.2, 124.0, 119.8, 116.7, 41.3, 38.9, 27.7, 20.2. **HRMS** (ESI) *m/z* calcd. for $C_{22}H_{25}N_2O_2$ $[M+H]^+$ 349.1911, found 349.1903.

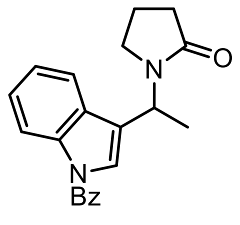

Chemical Formula: $C_{21}H_{20}N_2O_2$
Exact Mass: 332.1525

1-(1-(1-benzoyl-1*H*-indol-3-yl)ethyl)pyrrolidin-2-one (3t) – General procedure **II.1** was followed, using 1 mol% catalyst loading. The product was purified by flash column chromatography over silica gel using pentane–EtOAc (20 to 80%) as the mobile phase and was isolated as a white solid (117.0 mg, 0.352 mmol, 88%, major:minor > 95:5, mp = 131–133 °C). **^{1}H**

NMR (400 MHz, $CDCl_3$) δ 8.26 (d, *J* = 8.2 Hz, 1H), 7.77–7.70 (m, 2H), 7.68–7.59 (m, 2H), 7.59–7.51 (m, 2H), 7.37 (ddd, *J* = 8.4, 7.2, 1.3 Hz, 1H), 7.30 (td, *J* = 7.6, 1.1 Hz, 1H), 7.23 (d, *J* = 1.3 Hz, 1H), 5.71 (qd, *J* = 7.0, 1.3 Hz, 1H), 3.27 (ddd, *J* = 9.5, 8.3, 5.9 Hz, 1H), 2.89 (ddd, *J* = 9.6, 8.6, 5.4 Hz, 1H), 2.51–2.30 (m, 2H), 2.01–1.74 (m, 2H), 1.51 (d, *J* = 6.9 Hz, 3H). **^{13}C NMR** (101 MHz, $CDCl_3$) δ 174.5, 168.6, 136.7, 134.6, 132.2, 129.8, 129.3, 128.9, 125.6, 124.9, 124.4, 122.3, 119.9, 116.4, 42.3, 42.2, 31.6, 17.9, 16.4. **HRMS** (ESI) *m/z* calcd. for $C_{21}H_{21}N_2O_2$ [M+H]$^+$ 333.1598, found 333.1588.

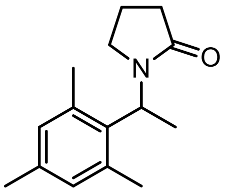

Chemical Formula: $C_{15}H_{21}NO$
Exact Mass: 231.1623

1-(1-mesitylethyl)pyrrolidin-2-one (3u) – General procedure **II.1** was followed, using 10 mol% catalyst loading. The product was purified by flash column chromatography over silica gel using pentane–EtOAc (20 to 80%) as the mobile phase and was isolated as a white solid (51.8 mg, 0.224 mmol, 56%, major:minor > 95:5, mp = 131–133 °C). **^{1}H NMR** (500 MHz, $CDCl_3$) δ 6.82 (s, 2H), 5.40 (q, *J* = 7.4 Hz, 1H), 3.42 (dt, *J* = 9.4, 7.6 Hz, 1H), 3.19 (ddd, *J* = 9.5, 8.5, 4.2 Hz, 1H), 2.44–2.30 (m, 8H), 2.24 (s, 3H), 2.06–1.83 (m, 2H), 1.60 (d, *J* = 7.4 Hz, 3H). **^{13}C NMR** (126 MHz, $CDCl_3$) δ 174.8, 136.9, 136.7, 134.3, 130.5, 49.6, 45.3, 31.7, 21.4, 20.8, 18.3, 17.9. **HRMS** (ESI) *m/z* calcd. for $C_{15}H_{21}NONa$ [M+Na]$^+$ 254.1515, found 254.1509.

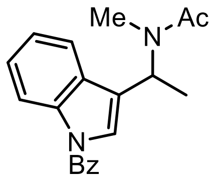

Chemical Formula: $C_{20}H_{20}N_2O_2$
Exact Mass: 320.1525

***N*-(1-(1-benzoyl-1*H*-indol-3-yl)ethyl)-*N*-methylacetamide (3v) –** General procedure **II.1** was followed, using 1 mol% catalyst loading. The product was purified by flash column chromatography over silica gel using pentane–EtOAc (20 to 80%) as the mobile phase and was isolated as a colorless oil (42.3 mg, 0.132 mmol, major:minor 83:17, 33%).**^{1}H NMR** (400 MHz, $CDCl_3$) δ 8.25 (d, *J* = 8.2 Hz, 1H), 7.78–7.70 (m, 2H), 7.67–7.58 (m, 1H), 7.59–7.50 (m, 3H), 7.36 (ddd, *J* = 8.4, 7.2, 1.3 Hz, 1H), 7.32–7.23 (m, 1H), 7.23 (d, *J* = 1.4 Hz, 1H), 6.25 (qd, *J* = 6.9, 1.4 Hz, 1H), 2.64 (s, 3H), 2.14 (s, 3H), 1.43 (d, *J* = 6.9 Hz, 3H). **^{13}C NMR** (101 MHz, $CDCl_3$) δ 170.6, 168.5, 136.6, 134.6, 132.2, 129.8, 129.2, 128.8, 125.5, 125.3, 124.3, 122.8, 119.9, 116.3, 44.0, 29.9, 22.5, 16.0. **HRMS** (ESI) *m/z* calcd. for $C_{20}H_{21}N_2O_2$ [M+H]$^+$ 321.1598, found 321.1590.

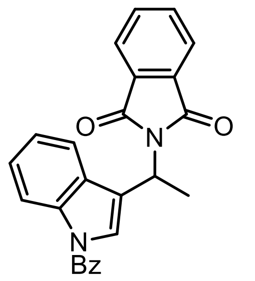

Chemical Formula: $C_{25}H_{18}N_2O_3$
Exact Mass: 394.1317

2-(1-(1-benzoyl-1H-indol-3-yl)ethyl)isoindoline-1,3-dione (3w) – General procedure **II.1** was followed, using 1 mol% catalyst loading. The product was purified by flash column chromatography over silica gel using pentane–EtOAc (20 to 80%) as the mobile phase and was isolated as a white solid (131.0 mg, 0.332 mmol, 83%, mp = 111–115 °C). **^{1}H NMR** (400 MHz, $CDCl_3$) δ 8.35 (d, *J* = 8.2 Hz, 1H), 7.84–7.76 (m, 4H), 7.72–7.55 (m, 7H), 7.35 (ddd, *J* = 8.4, 7.2, 1.3 Hz, 1H), 7.27 (td, *J* = 7.5, 1.1 Hz, 1H), 5.83 (qd, *J* = 7.2, 1.1 Hz, 1H), 1.90 (d, *J* = 7.2 Hz, 3H). **^{13}C NMR** (101 MHz, $CDCl_3$) δ 168.7, 168.1, 136.3, 134.5, 134.1, 132.2, 132.0, 129.6 (2C), 128.8, 126.8, 125.3, 124.2, 123.4, 120.7, 119.1, 116.6, 41.5, 17.5. **HRMS** (ESI) *m/z* calcd. for $C_{25}H_{19}N_2O_3$ $[M+H]^+$ 395.1390, found 395.1382.

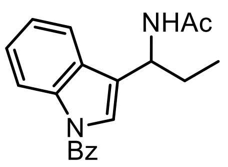

Chemical Formula: $C_{20}H_{20}N_2O_2$
Exact Mass: 320.1525

***N*-(1-(1-benzoyl-1*H*-indol-3-yl)propyl)acetamide (3x) –** General procedure **II.1** was followed, using 1 mol% catalyst loading and allowing the reaction run for 18 h. The product was purified by flash column chromatography over silica gel using pentane–EtOAc (20 to 80%) as the mobile phase and was isolated as a white solid (102.5 mg, 0.32 mmol, 80%, major:minor > 95:5, mp = 168–170 °C). **^{1}H NMR** (400 MHz, $CDCl_3$) δ 8.32 (dt, *J* = 8.2, 1.0 Hz, 1H), 7.75–7.69 (m, 2H), 7.69–7.58 (m, 2H), 7.58–7.50 (m, 2H), 7.39 (ddd, *J* = 8.4, 7.3, 1.3 Hz, 1H), 7.35–7.28 (m, 1H), 7.21 (d, *J* = 0.9 Hz, 1H), 5.54 (d, *J* = 8.7 Hz, 1H), 5.33–5.13 (m, 1H), 2.06–1.81 (m, 5H), 0.99 (t, **J** = 7.4 Hz, 3H). **^{13}C NMR** (101 MHz, $CDCl_3$) δ 169.5, 168.6, 136.8, 134.6, 132.2, 129.5, 129.3, 128.9, 125.5, 124.4, 124.2, 123.0, 119.7, 116.7, 47.3, 27.6, 23.6, 11.0. **HRMS** (ESI) *m/z* calcd. for $C_{20}H_{21}N_2O_2$ $[M+H]^+$ 321.1598, found 321.1590.

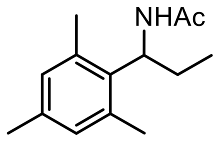

Chemical Formula: $C_{14}H_{21}NO$
Exact Mass: 219.1623

***N*-(1-mesitylpropyl)acetamide (3y) –** General procedure **II.1** was followed, using 10 mol% catalyst loading of TfOH. The product was purified by flash column chromatography over silica gel using pentane–EtOAc (20 to 80%) as the mobile phase and was isolated as a pale yellow oil (102.5 mg, 0.32 mmol, 73%, major:minor > 95:5). **^{1}H NMR** (250 MHz, $CDCl_3$) δ 6.82 (s, 2H), 5.93 (d, *J* = 8.0 Hz, 1H), 5.31 (q, *J* = 8.0 Hz, 1H), 2.41 (s, 6H), 2.23 (s, 3H), 1.96 (s, 3H), 1.93–1.68 (m, 2H), 0.89 (t, *J* = 7.4 Hz, 3H).**^{13}C NMR** (101 MHz, $CDCl_3$) δ 169.4, 136.5, 136.0, 135.1, 130.4, 51.6, 27.4, 23.4, 21.2, 20.8, 11.4. **HRMS** (ESI) *m/z* calcd. for $C_{20}H_{21}N_2O_2$ $[M+H]^+$ 220.1696, found 220.1691.

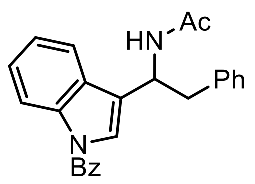

Chemical Formula: $C_{25}H_{22}N_2O_2$
Exact Mass: 382.1681

***N*-(1-(1-benzoyl-1*H*-indol-3-yl)-2-phenylethyl)acetamide (3z) –** General procedure **II.1** was followed, using 1 mol% catalyst loading and allowing the reaction run for 18 h. The product was purified by flash column chromatography over silica gel using pentane–EtOAc (20 to 80%) as the mobile phase and was isolated as a white solid (97.9 mg, 0.256 mmol, 64%, major:minor > 95:5, mp = 162–164 °C). ^{1}**H NMR** (500 MHz, $CDCl_3$) δ 8.36 (d, *J* = 8.3 Hz, 1H), 7.68 (dt, *J* = 7.5, 1.0 Hz, 1H), 7.62–7.58 (m, 1H), 7.57–7.54 (m, 2H), 7.52–7.45 (m, 2H), 7.41 (ddd, *J* = 8.3, 7.2, 1.3 Hz, 1H), 7.35 (td, *J* = 7.5, 1.1 Hz, 1H), 7.32–7.17 (m, 3H), 7.12–7.07 (m, 2H), 7.03 (d, *J* = 0.9 Hz, 1H), 5.71 (d, *J* = 8.5 Hz, 1H), 5.65–5.55 (m, 1H), 3.23 (qd, *J* = 13.8, 7.0 Hz, 2H), 1.94 (s, 3H). ^{13}**C NMR** (126 MHz, $CDCl_3$) δ 169.4, 168.5, 137.5, 136.7, 134.4, 132.2, 129.4 (2C), 129.2, 128.7, 128.6, 126.8, 125.6, 125.1, 124.2, 121.7, 119.6, 116.8, 47.1, 40.4, 23.5. **HRMS** (ESI) *m/z* calcd. for $C_{25}H_{22}N_2O_2Na$ $[M+Na]^+$ 405.1573, found 405.1566.

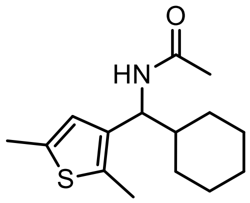

Chemical Formula: $C_{15}H_{23}NOS$
Exact Mass: 265.1500

***N*-((1-benzoyl-1*H*-indol-3-yl)(cyclohexyl)methyl)acetamide (3aa) –** General procedure **II.1** was followed (0.2 mmol scale), using 1 mol% catalyst loading and allowing the reaction run for 18 h. The product was purified by flash column chromatography over silica gel using pentane–EtOAc (20 to 80%) as the mobile phase and was isolated as a white solid (31.3 mg, 0.118 mmol, 59%, major:minor > 95:5, mp = 105–108 °C). ^{1}**H NMR** (500 MHz, $CDCl_3$) δ 6.43 (d, *J* = 1.3 Hz, 1H), 5.70 (d, *J* = 9.2 Hz, 1H), 4.76 (t, *J* = 9.3 Hz, 1H), 2.38 (s, 3H), 2.36 (s, 3H), 1.94 (s, 3H), 1.92–1.83 (m, 1H), 1.79–1.72 (m, 1H), 1.67–1.61 (m, 2H), 1.58–1.40 (m, 2H), 1.25–0.98 (m, 4H), 0.91–0.80 (m, 1H). ^{13}**C NMR** (126 MHz, $CDCl_3$) δ 169.1, 137.0, 136.3, 133.2, 123.3, 52.0, 43.4, 30.1, 29.9, 26.4, 26.2 (2C), 23.6, 15.4, 13.2. **HRMS** (ESI) *m/z* calcd. for $C_{15}H_{24}NOS$ $[M+H]^+$ 266.1573, found 266.1583.

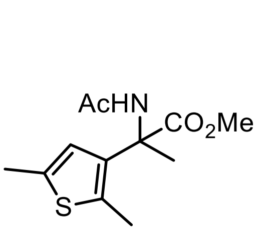

Chemical Formula: $C_{12}H_{17}NO_3S$
Exact Mass: 255.0929

methyl 2-acetamido-2-(2,5-dimethylthiophen-3-yl)propanoate (3ab) – General procedure **II.1** was followed, using 1 mol% catalyst loading and allowing the reaction run for 18 h. The product was purified by flash column chromatography over silica gel using pentane–EtOAc (20 to 80%) as the mobile phase and was isolated as a white solid (76.9 mg, 0.301 mmol, 75%, major:minor > 95:5, mp = 135–138 °C).^{1}**H NMR** (400 MHz, $CDCl_3$) δ 6.72 (s, 1H), 6.69–6.65 (m, 1H), 3.75 (s, 3H), 2.37 (s, 3H), 2.28 (s, 3H), 2.00 (s, 3H), 1.98 (s, 3H). ^{13}**C NMR** (101 MHz, $CDCl_3$) δ 173.9, 168.6, 134.9, 134.9, 132.7, 126.2,

59.8, 53.4, 23.9, 23.8, 15.3, 14.1. **HRMS** (ESI) *m/z* calcd. for $C_{12}H_{18}NO_3S$ $[M+H]^+$ 256.1002, found 256.0996.

II.3 Characterization Data of Derivatized Compounds (Equations 1 and 2)

1-(2,5-dimethylthiophen-3-yl)ethan-1-amine (5)

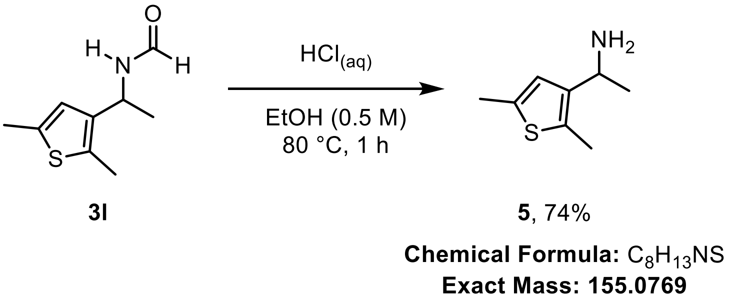

To a solution of **1a** (71.1 mg, 1 mmol, 1 equiv) in EtOH (1 M) was added HCl (1 mL, 37%). The reaction mixture was stirred at 80 °C for 1 h. It was then cooled to room temperature, diluted with water, and washed with Et_2O (2 x 5 mL). The aqueous solution was basified with solid NaOH and extracted with Et_2O (3 x 10 mL). The organic layer was washed with brine, dried over K_2CO_3, filtered, and concentrated under reduced pressure to give **5** as a yellow oil (115 mg, 0.74 mmol, 74%).

^{1}H NMR (500 MHz, $CDCl_3$) δ 6.64 (d, *J* = 1.3 Hz, 1H), 4.11 (q, *J* = 6.6 Hz, 1H), 2.38 (s, 3H), 2.31 (s, 3H), 1.53 (brs, 2H), 1.30 (d, *J* = 6.6 Hz, 3H). **^{13}C NMR** (126 MHz, $CDCl_3$) δ 143.3, 135.8, 129.8, 123.3, 44.9, 24.6, 15.2, 12.6. **HRMS** (ESI) *m/z* calcd. for $C_8H_{14}NS$ $[M+H]^+$ 156.0841, found 156.0842.

3-(1-isocyanoethyl)-2,5-dimethylthiophene (6)

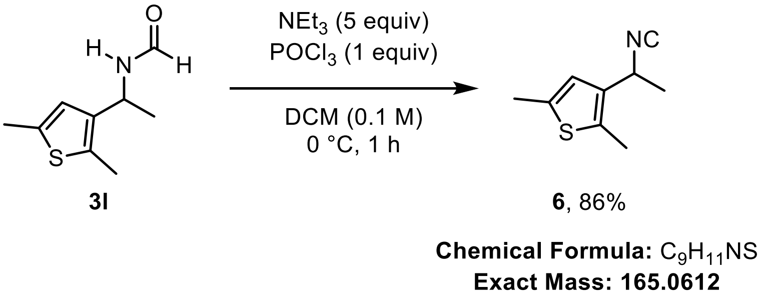

To a solution of **3l** (71.1 mg, 1 mmol, 1 equiv) and NEt_3 (697 µl, 5 mmol, 5 equiv) in DCM (0.1 M) was slowly added $POCl_3$ (93 µl, 1 mmol, 1 equiv). The reaction mixture was then shaken for 5 minutes and filtered through a silica plug eluted by DCM. The solvent was removed by rotary evaporator to give **6** as a yellow oil (133.5 mg, 0.86 mmol, 86%).

^{1}H NMR (500 MHz, $CDCl_3$) δ 6.69 (d, *J* = 1.3 Hz, 1H), 4.75 (dtt, *J* = 6.9, 3.9, 1.9 Hz, 1H), 2.40 (s, 3H), 2.33 (s, 3H), 1.62 (dt, *J* = 6.9, 2.2 Hz, 3H). **^{13}C NMR** (126 MHz, $CDCl_3$) δ 154.8 (m), 136.9, 133.9, 132.5 (m), 123.44, 47.7 (m), 23.6, 15.2, 12.6.

II.4 Characterization Data of Hemiaminal 1-HFIP (Figure 1C)

***N*-(1-((1,1,1,3,3,3-hexafluoropropan-2-yl)oxy)ethyl)formamide (1-HFIP)**

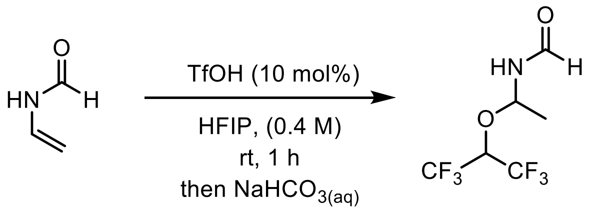

1a

1-HFIP, 27%

Chemical Formula: $C_6H_7F_6NO_2$

Exact Mass: 239,0381

To a solution of **1a** (71.1 mg, 1 mmol, 1 equiv) in HFIP (2.5 mL, 0.4 M) was added TfOH (8.8 µL, 0.1 mmol, 10 mol%). The reaction mixture was stirred at room temperature for 1 h. Then, it was quenched by a saturated solution of sodium bicarbonate (0.5 mL) and stirred for 5 minutes (note: it was crucial to quench the reaction with a saturated solution of a carbonate base, otherwise total decomposition was observed). After filtration over silica, eluting with EtOAc, the solvent was removed by rotary evaporator The product was purified by flash column chromatography over silica gel using pentane–EtOAc (20 to 80%) as the mobile phase and was isolated as a colorless oil (64.6 mg, 0.27 mmol, 27%), which decomposed quickly upon drying.

^{1}H NMR (500 MHz, Benzene-d6) δ 7.39 (s, 1H), 5.37 (dq, *J* = 9.5, 6.0 Hz, 1H), 5.04 (hept, *J* = 6.3 Hz, 1H), 4.55 (s, 1H), 0.68 (d, *J* = 6.0 Hz, 3H). **^{13}C NMR** (126 MHz, Benzene-d6) δ 162.1, 122.6 (m), 121.9 (m), 78.1, 74.2 (p, *J* = 32.5 Hz), 20.1. **^{19}F NMR** (471 MHz, Benzene-d6) δ -74.10 (m), -74.4 (m). **HRMS** (ESI) *m/z* calcd. for $C_6H_6NO_2F_6$ $[M+H]^-$ 238.0308, found 238.0305.

III. NMR Spectra

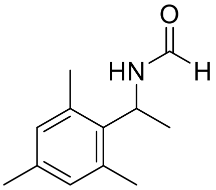

3a ($CDCl_3$)

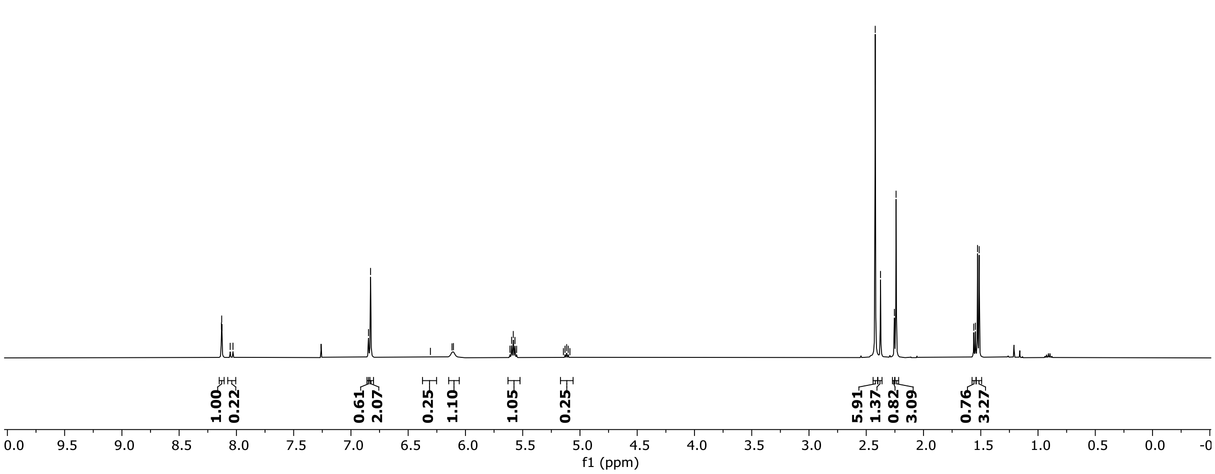

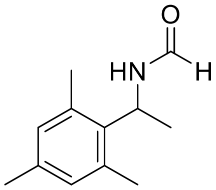

3a ($CDCl_3$)

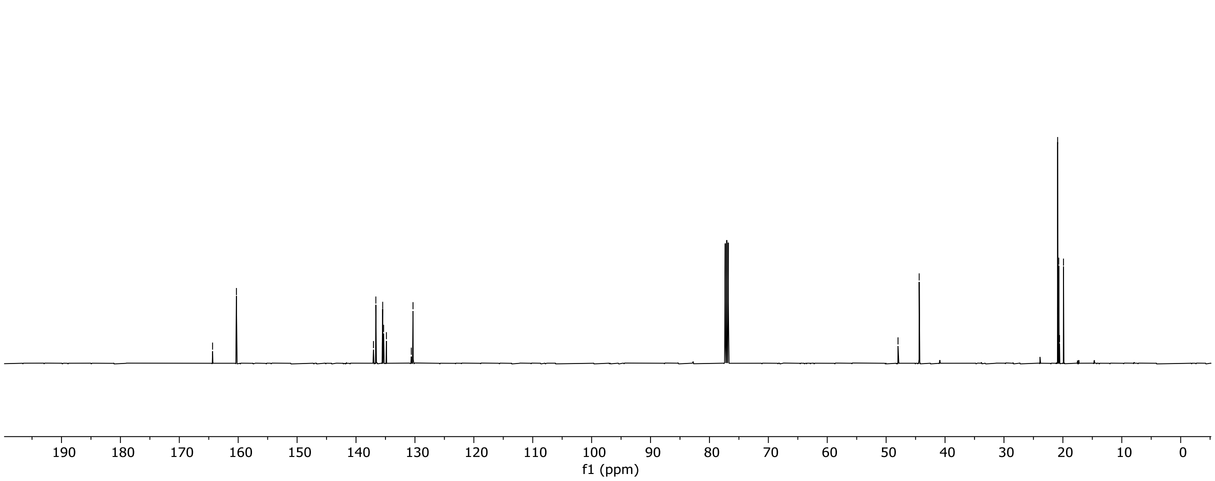

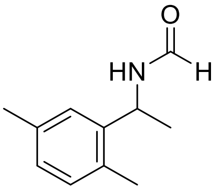

3b ($CDCl_3$)

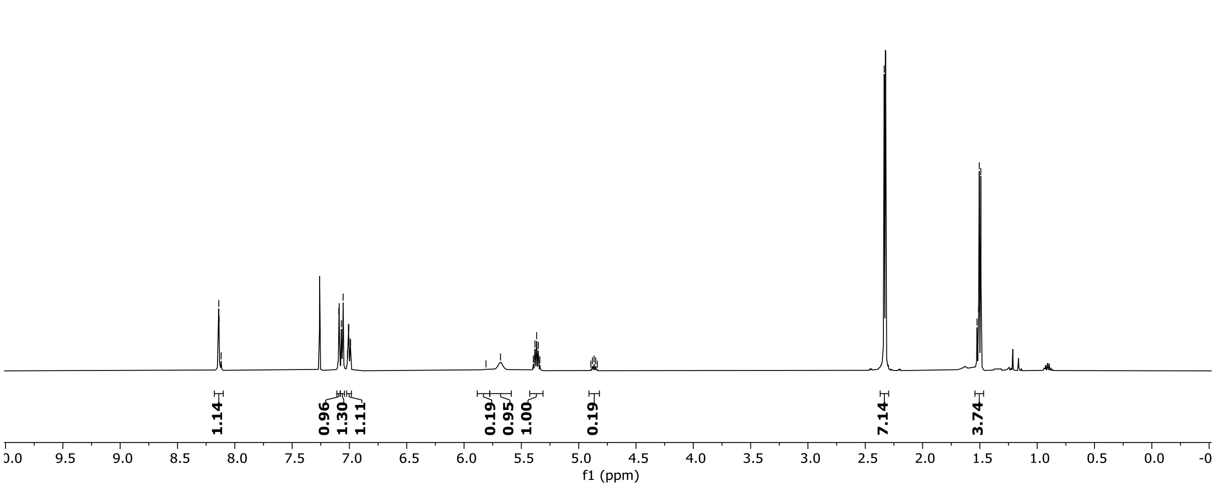

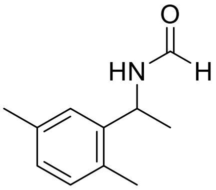

3b ($CDCl_3$)

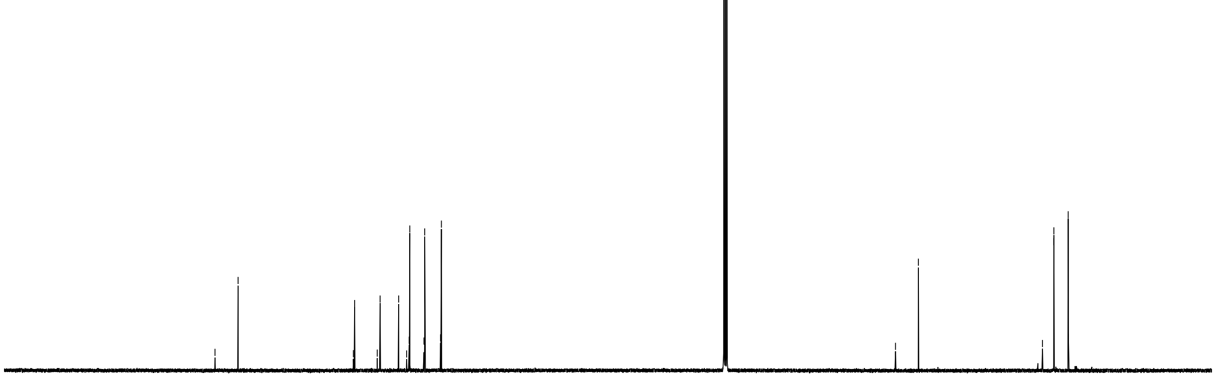

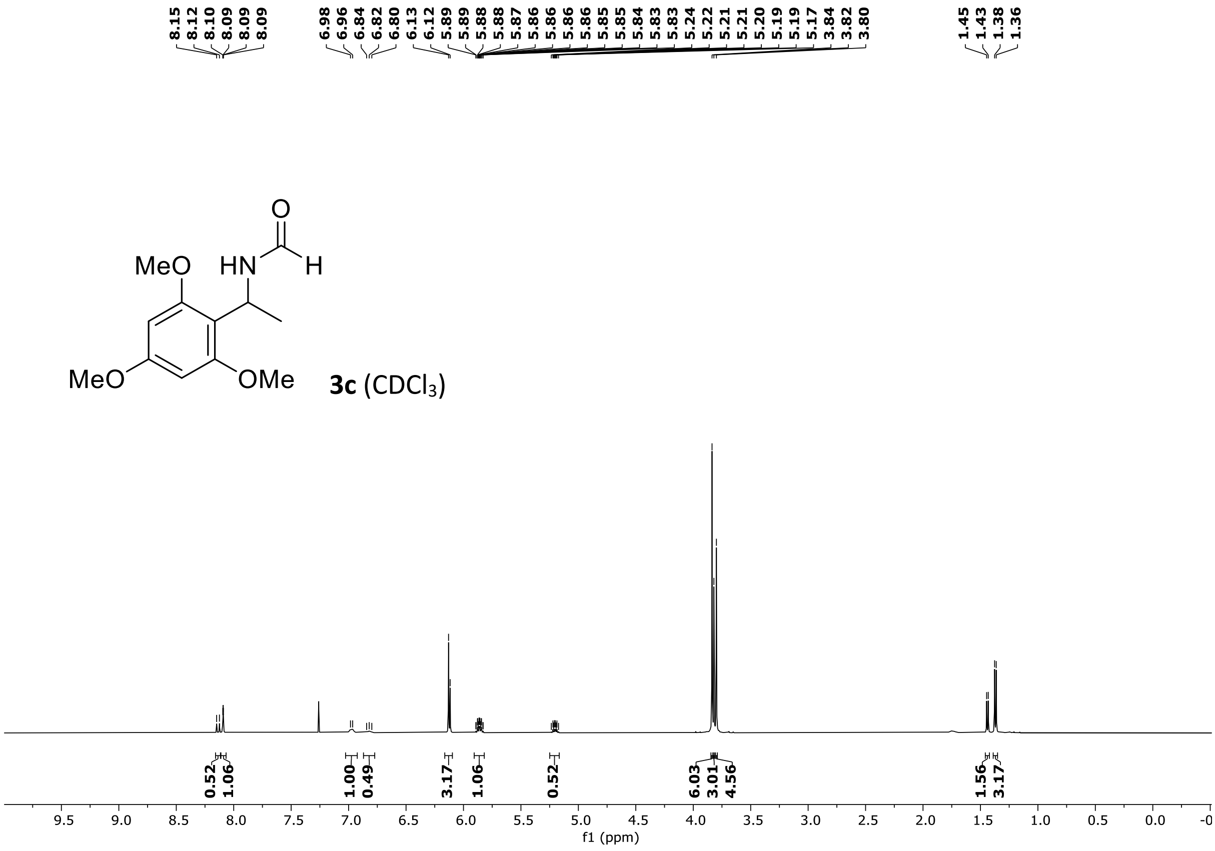
8.15 8.12 8.10 8.09 8.09 8.09 6.98 6.96 6.84 6.82 6.80 6.13 6.12 5.89 5.89 5.88 5.88 5.87 5.86 5.86 5.86 5.85 5.85 5.84 5.83 5.83 5.24 5.22 5.21 5.21 5.20 5.19 5.19 5.17 3.84 3.82 3.80 1.45 1.43 1.38 1.36
MeO
HN
O
H
MeO
OMe
3c (CDCl3)
0.52 1.06 1.00 0.49 3.17 1.06 0.52 6.03 3.01 4.56 1.56 3.17
9.5 9.0 8.5 8.0 7.5 7.0 6.5 6.0 5.5 5.0 4.5 4.0 3.5 3.0 2.5 2.0 1.5 1.0 0.5 0.0 -0
f1 (ppm)

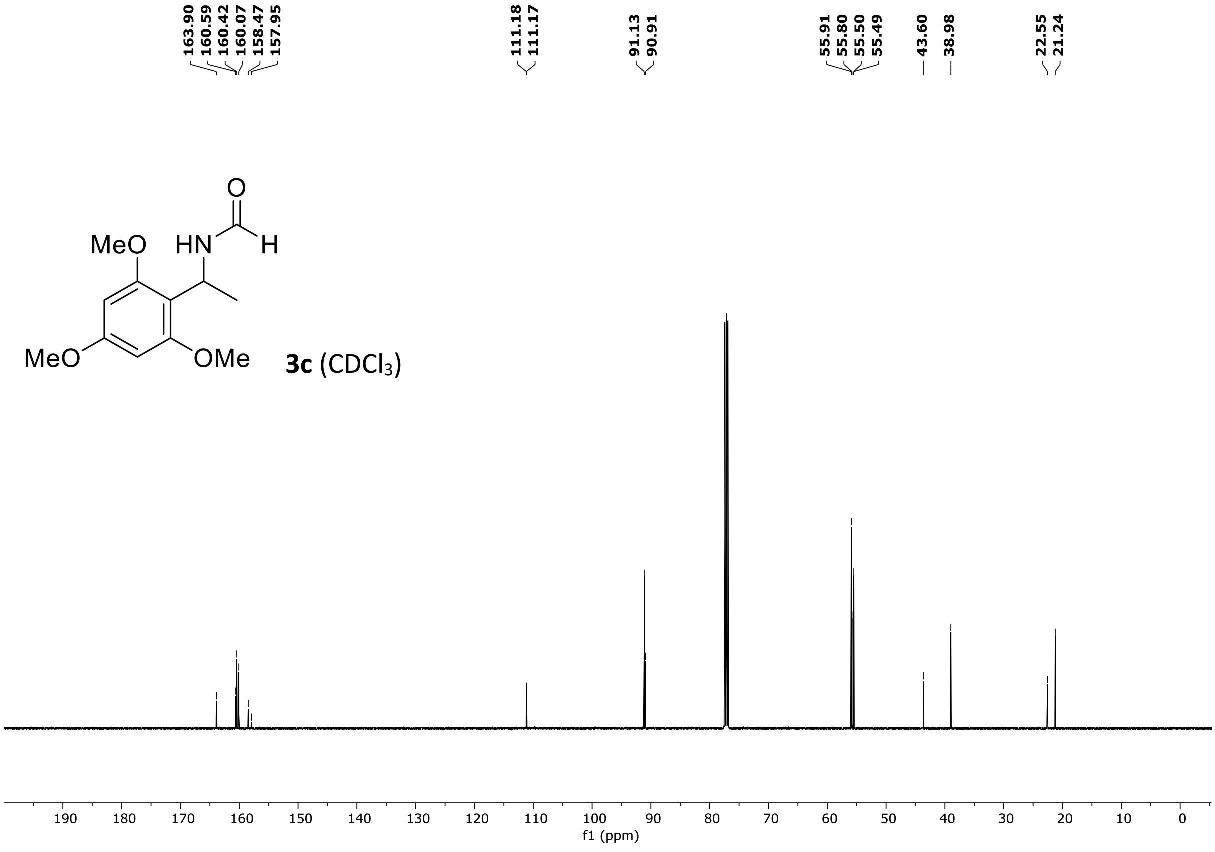
163.90 160.59 160.42 160.07 158.47 157.95 111.18 111.17 91.13 90.91 55.91 55.80 55.50 55.49 43.60 38.98 22.55 21.24
MeO
HN
O
H
MeO
OMe
3c (CDCl3)
190 180 170 160 150 140 130 120 110 100 90 80 70 60 50 40 30 20 10 0
f1 (ppm)

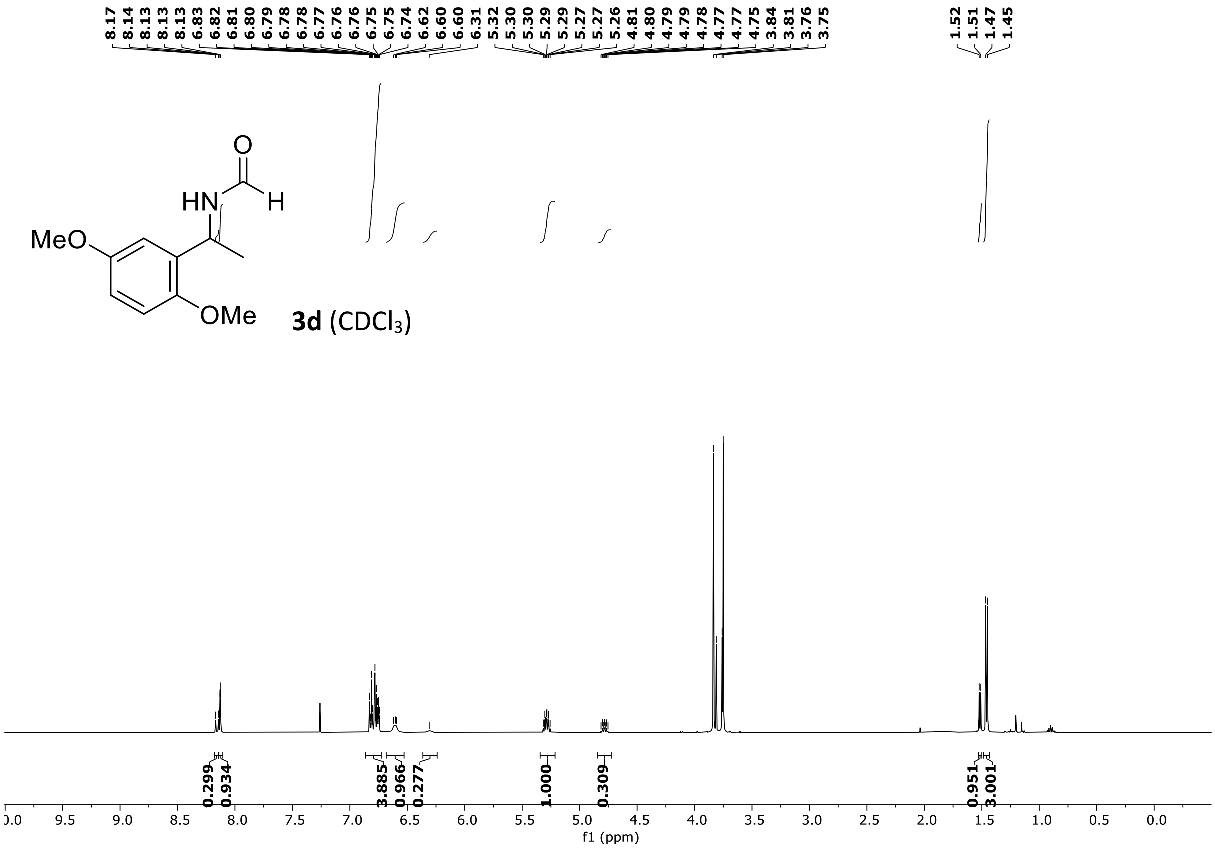

3d ($CDCl_3$)

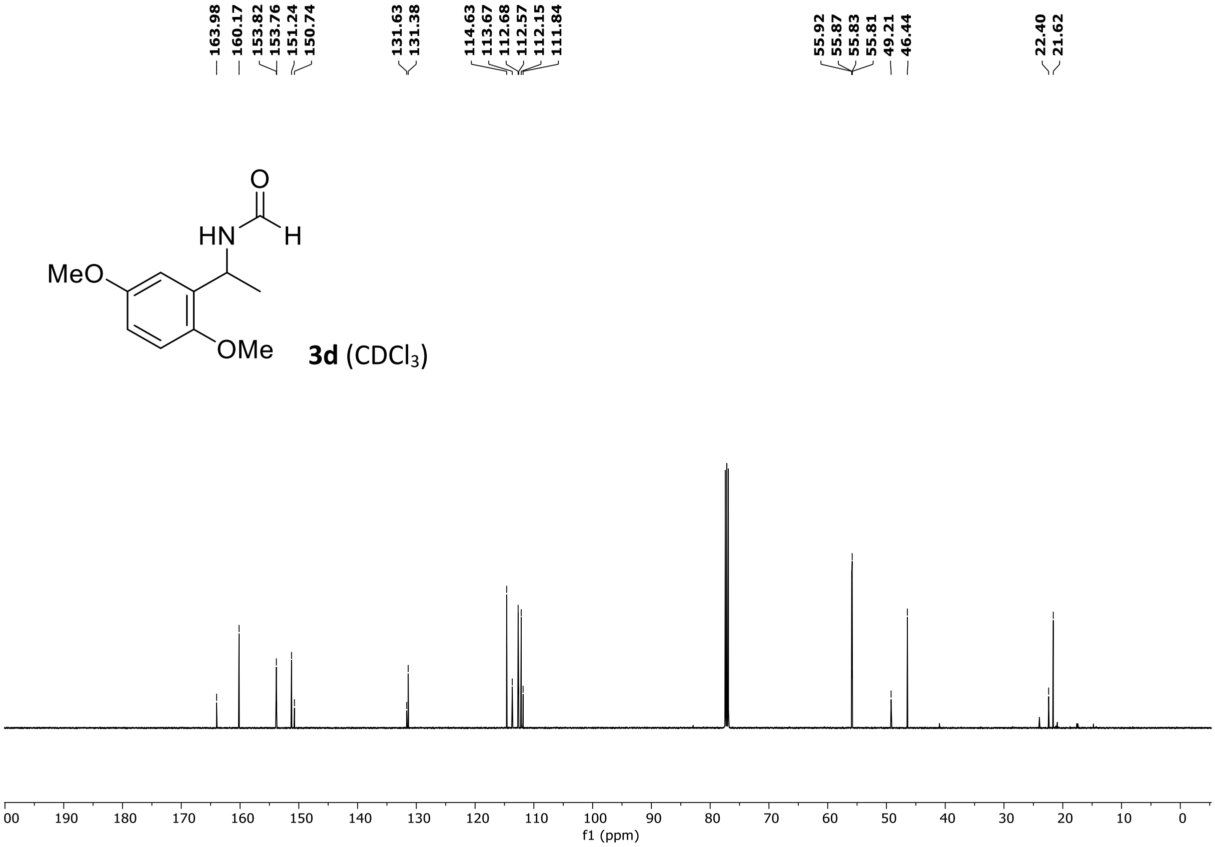

3d ($CDCl_3$)

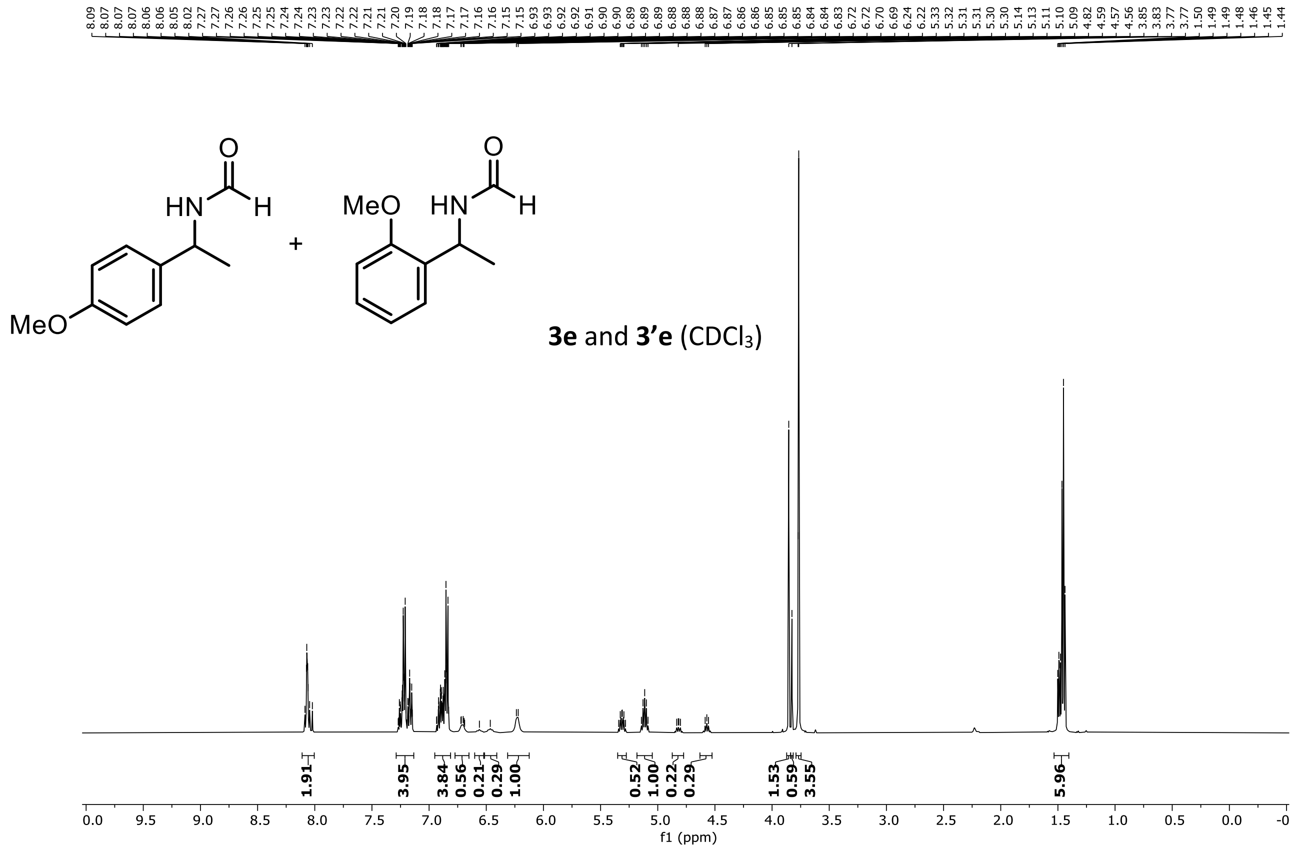
3e and 3'e (CDCl3)

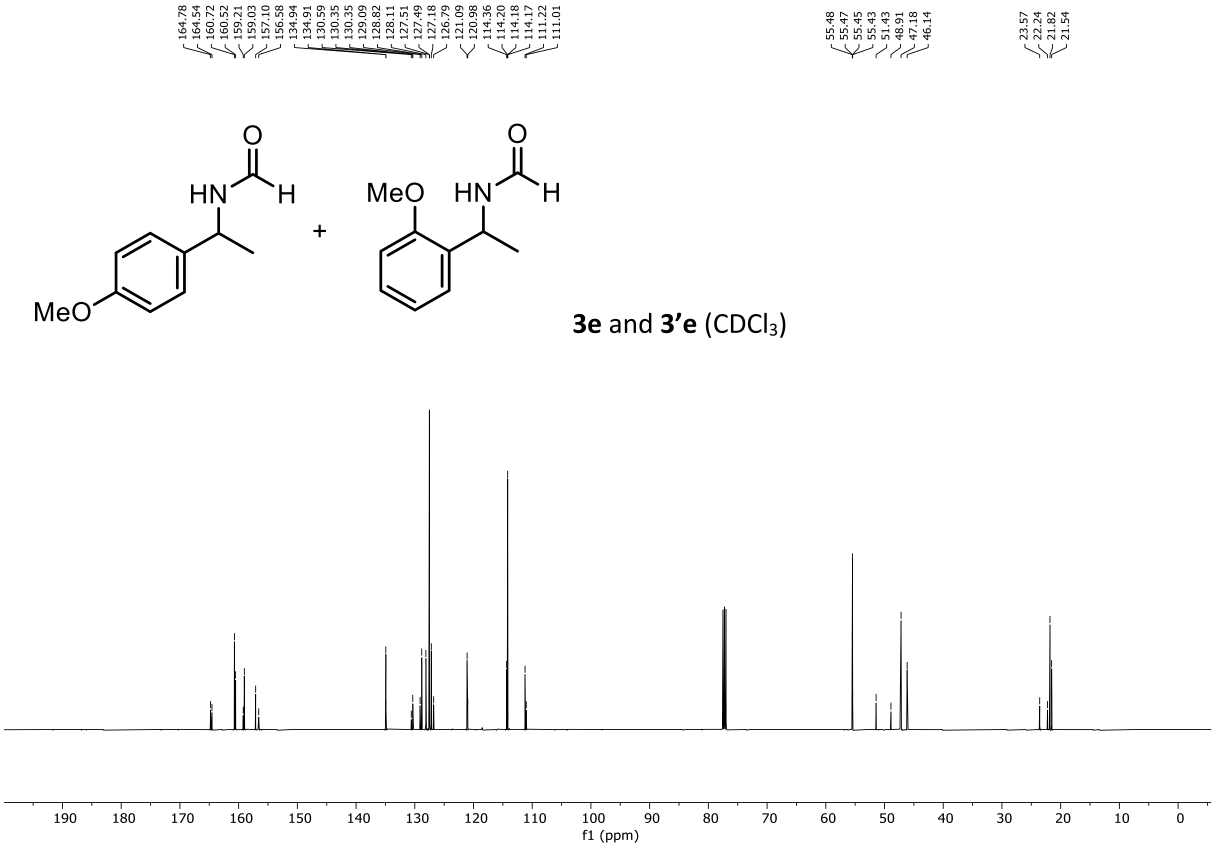
3e and 3'e (CDCl3)

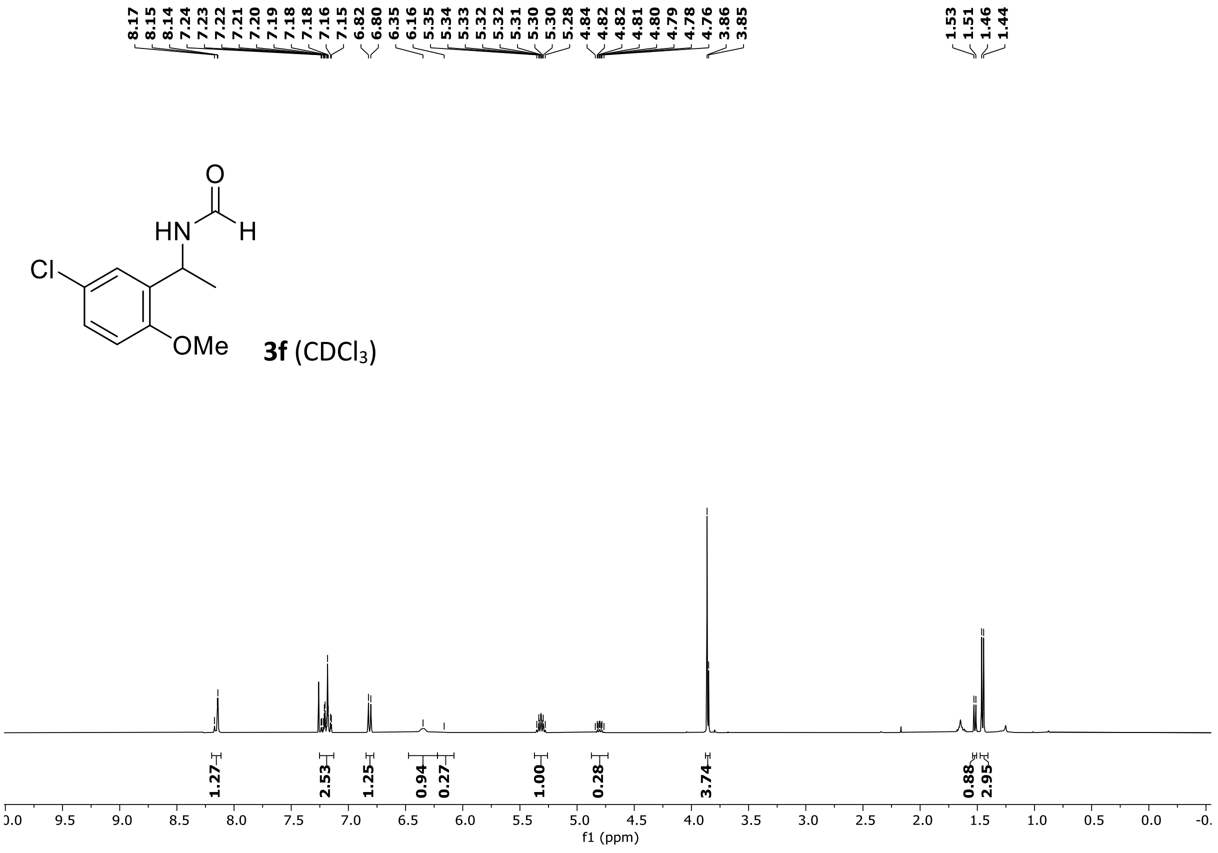
8.17 8.15 8.14 7.24 7.23 7.22 7.21 7.20 7.19 7.18 7.16 7.15 6.82 6.80 6.35 6.16 5.35 5.34 5.33 5.32 5.32 5.31 5.30 5.30 5.28 4.84 4.82 4.82 4.81 4.80 4.79 4.78 4.76 3.86 3.85
1.53 1.51 1.46 1.44
O
HN
H
Cl
OMe
3f (CDCl3)
1.27 2.53 1.25 0.94 0.27 1.00 0.28 3.74 0.88 2.95
f1 (ppm)

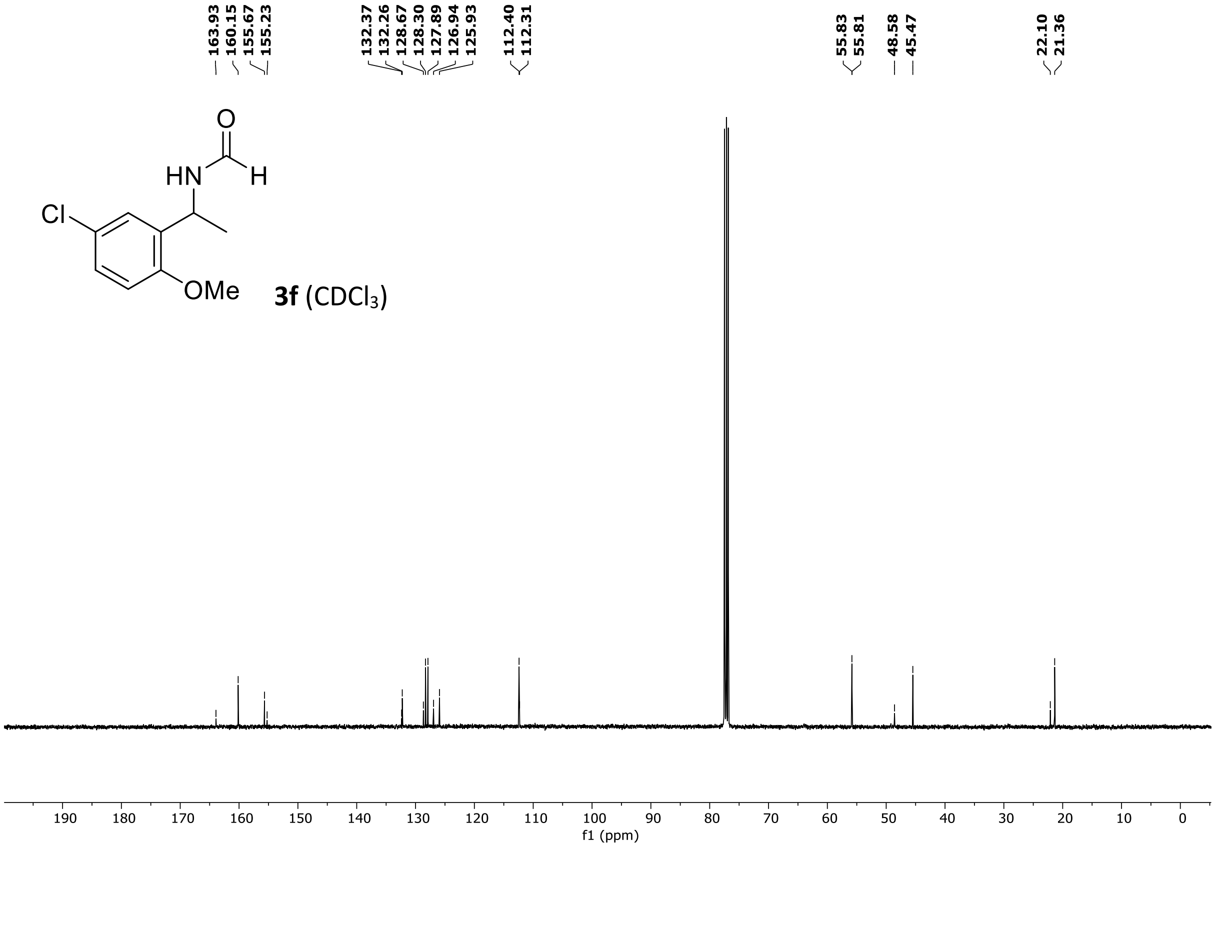
163.93 160.15 155.67 155.23
132.37 132.26 128.67 128.30 127.89 126.94 125.93
112.40 112.31
55.83 55.81 48.58 45.47
22.10 21.36
O
HN
H
Cl
OMe
3f (CDCl3)
190 180 170 160 150 140 130 120 110 100 90 80 70 60 50 40 30 20 10 0
f1 (ppm)

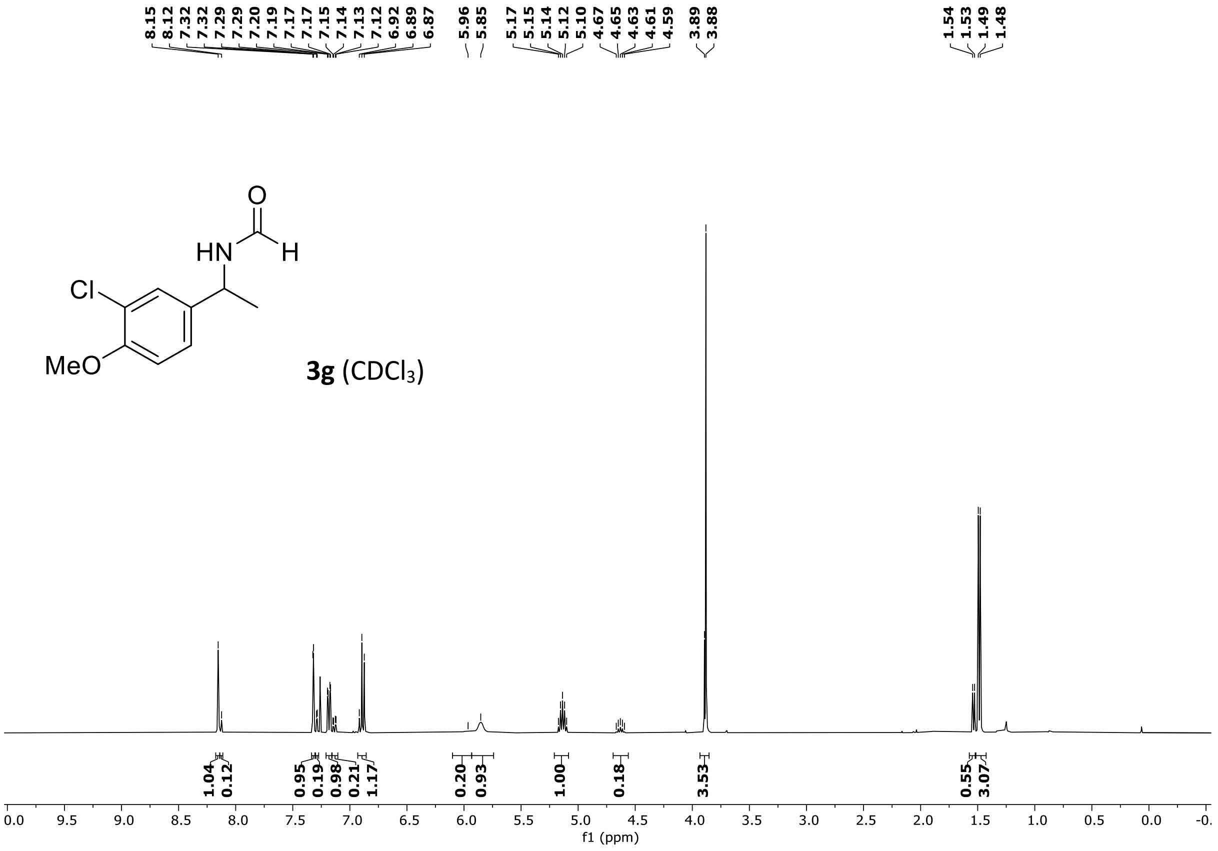
8.15 8.12 7.32 7.32 7.29 7.29 7.20 7.19 7.17 7.17 7.15 7.14 7.13 7.12 6.92 6.89 6.87 5.96 5.85 5.17 5.15 5.14 5.12 5.10 4.67 4.65 4.63 4.61 4.59 3.89 3.88 1.54 1.53 1.49 1.48
O
HN
H
Cl
MeO
3g (CDCl3)
1.04 0.12 0.95 0.19 0.98 0.21 1.17 0.20 0.93 1.00 0.18 3.53 0.55 3.07
f1 (ppm)

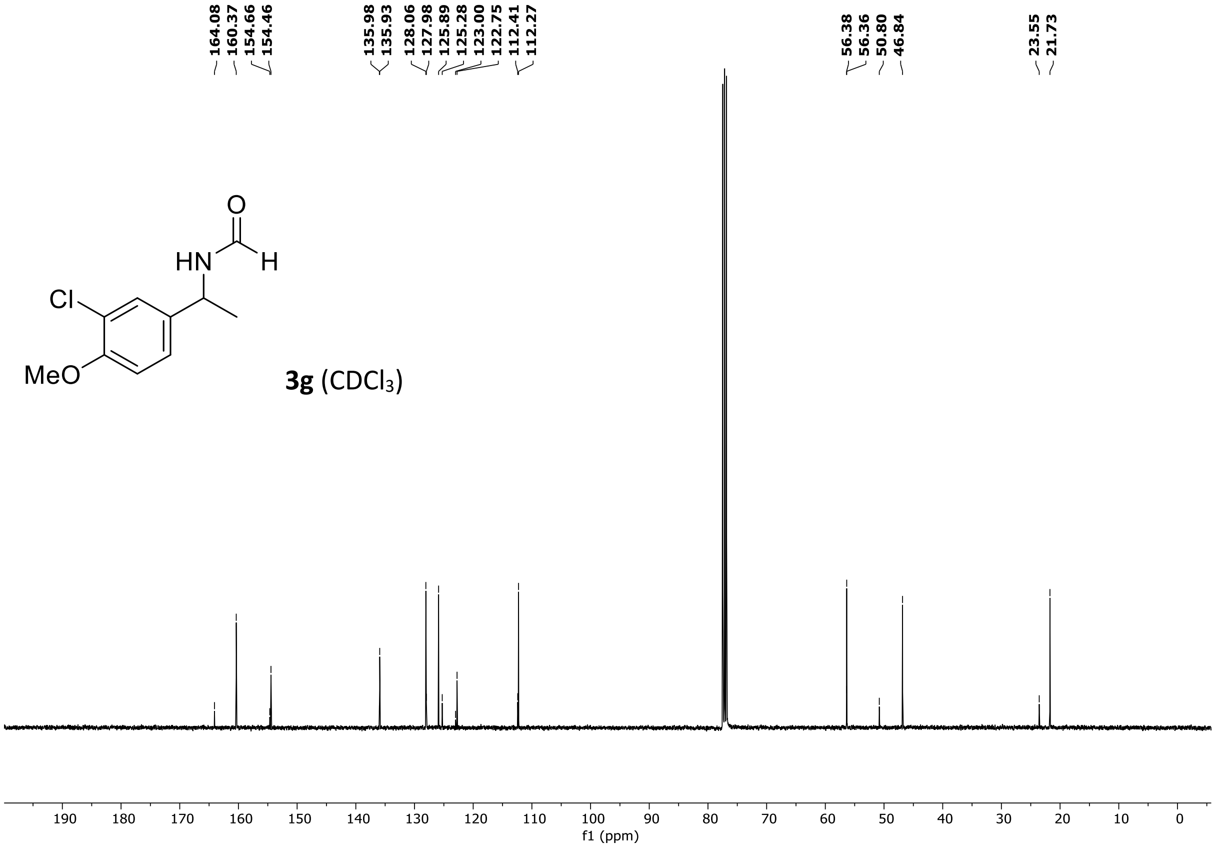
164.08 160.37 154.66 154.46 135.98 135.93 128.06 127.98 125.89 125.28 123.00 122.75 112.41 112.27 56.38 56.36 50.80 46.84 23.55 21.73
O
HN
H
Cl
MeO
3g (CDCl3)
f1 (ppm)

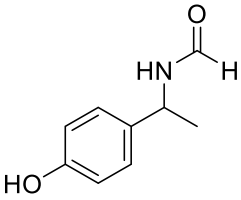

3h (DMSO-d6)

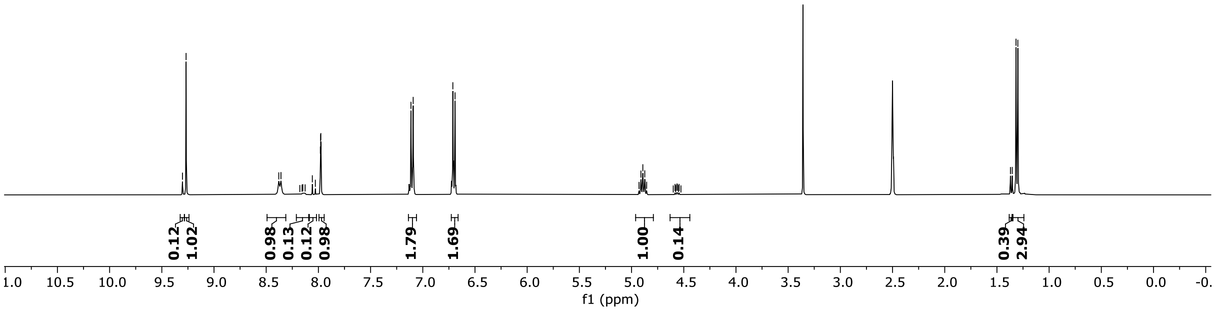

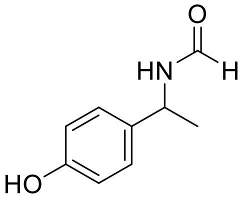

3h (DMSO-d6)

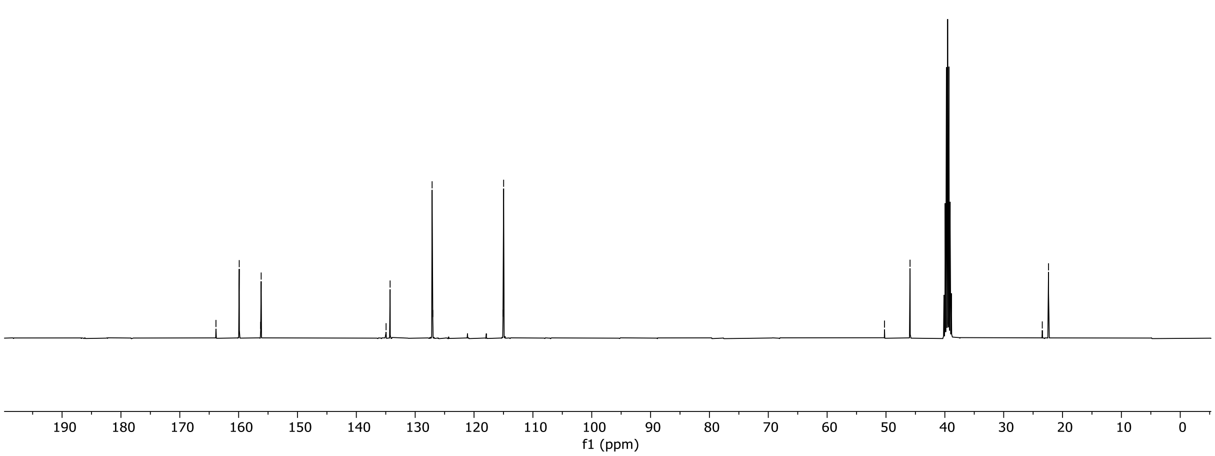

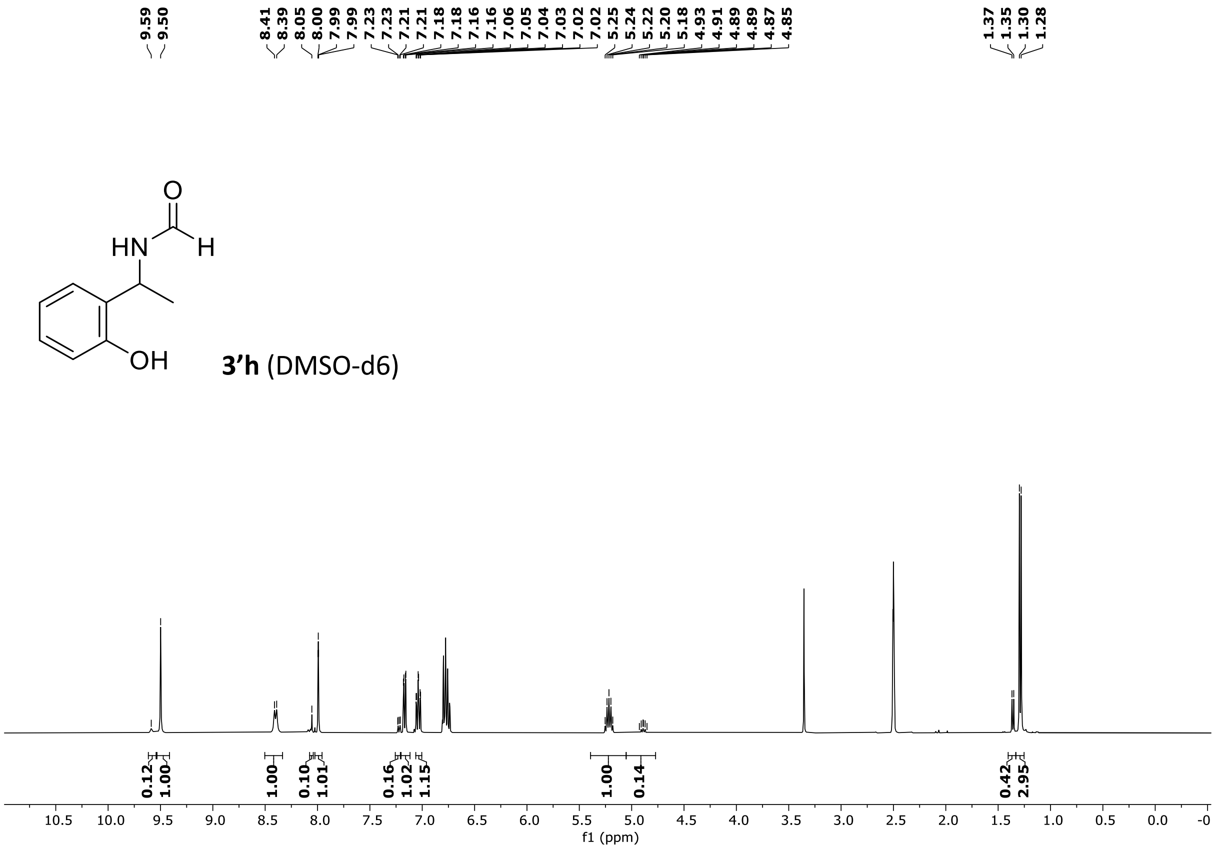
9.59
9.50
8.41
8.39
8.05
8.00
7.99
7.99
7.23
7.23
7.21
7.21
7.18
7.18
7.16
7.16
7.06
7.05
7.04
7.03
7.02
7.02
5.25
5.24
5.22
5.20
5.18
4.93
4.91
4.89
4.87
4.85
1.37
1.35
1.30
1.28
O
HN
H
OH
3’h (DMSO-d6)
0.12
1.00
1.00
0.10
1.01
0.16
1.02
1.15
1.00
0.14
0.42
2.95
f1 (ppm)

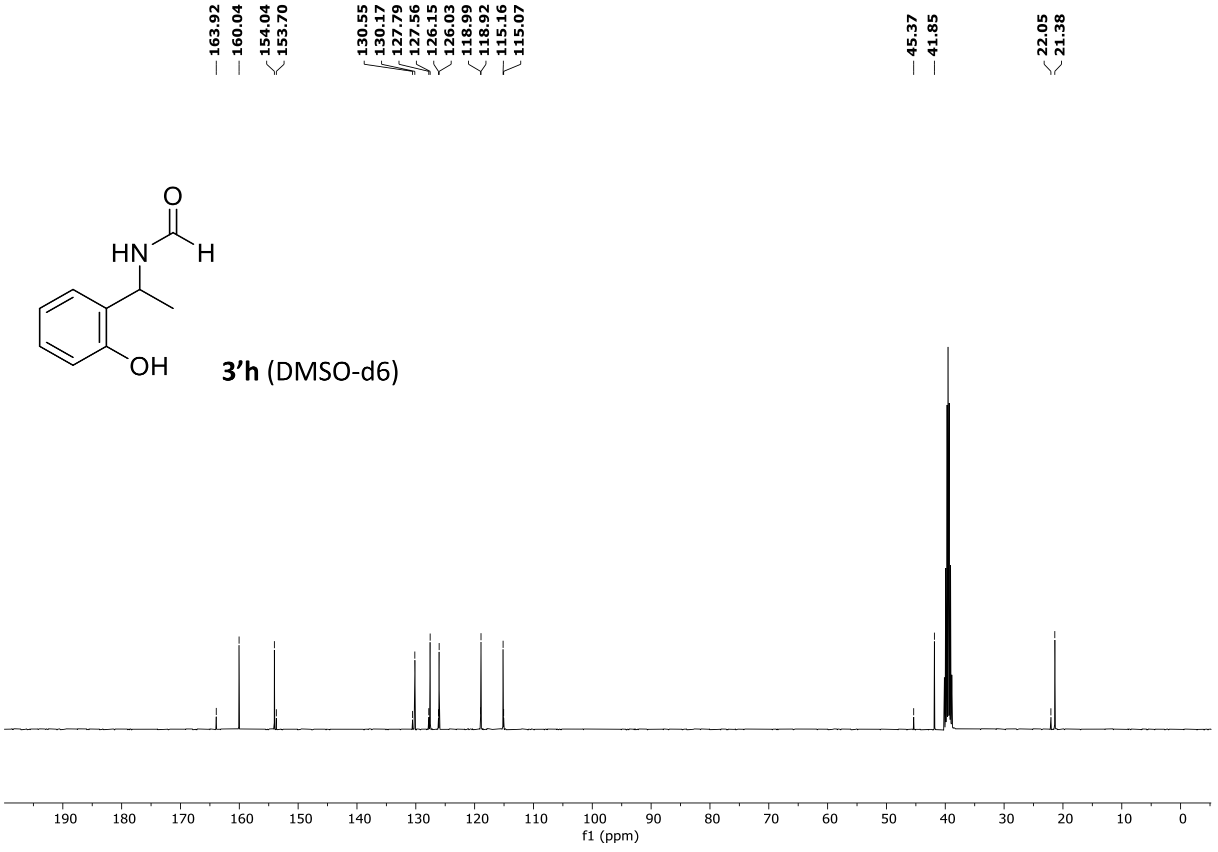
163.92
160.04
154.04
153.70
130.55
130.17
127.79
127.56
126.15
126.03
118.99
118.92
115.16
115.07
45.37
41.85
22.05
21.38
O
HN
H
OH
3’h (DMSO-d6)
f1 (ppm)

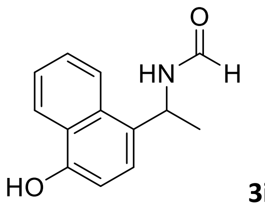

3i (DMSO-d6)

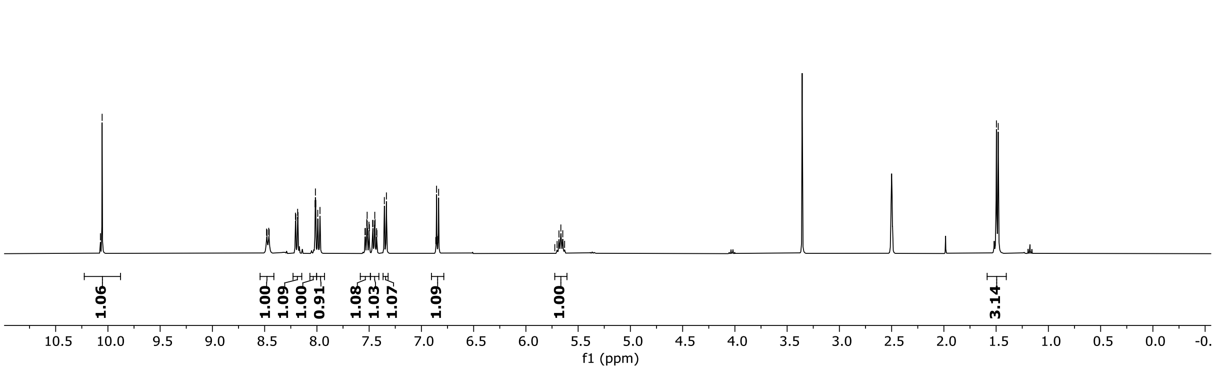

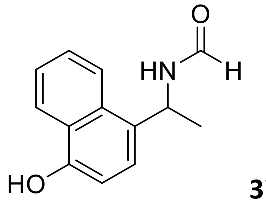

3i (DMSO-d6)

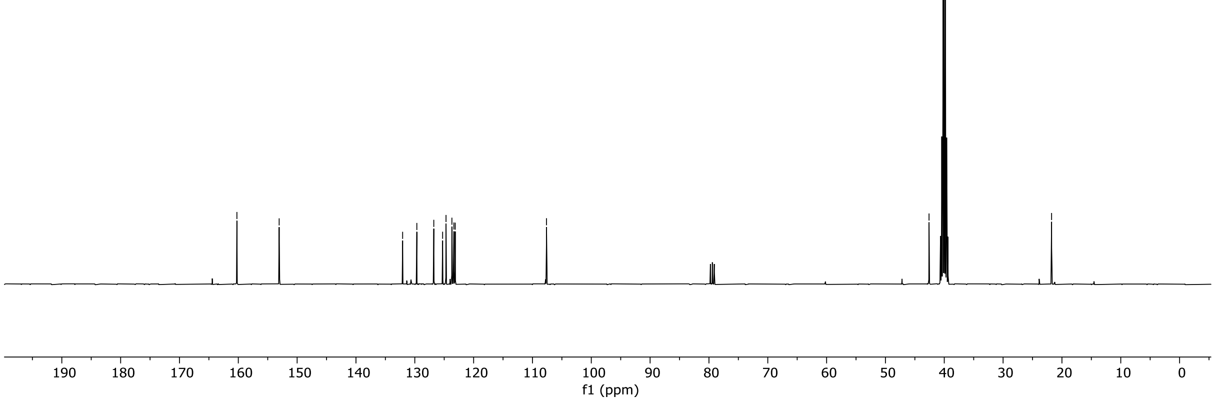

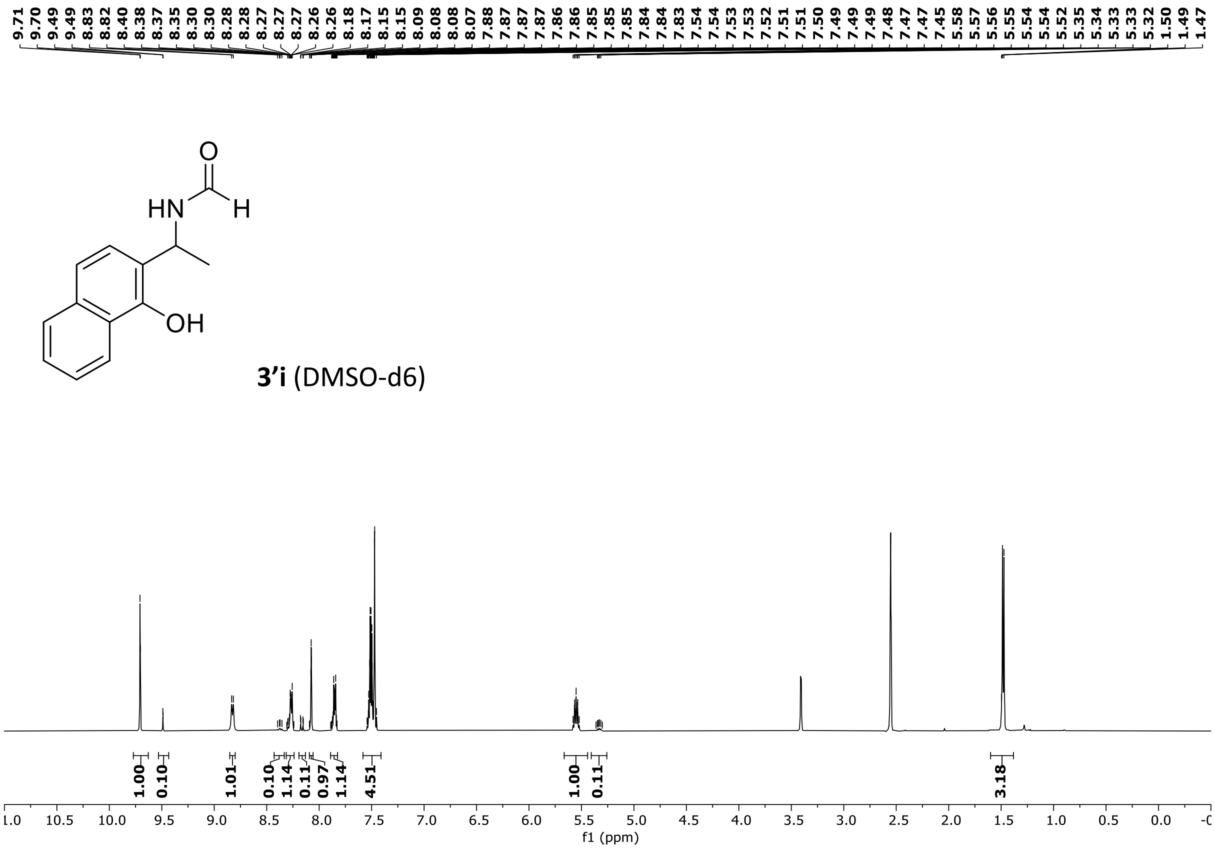

3'i (DMSO-d6)

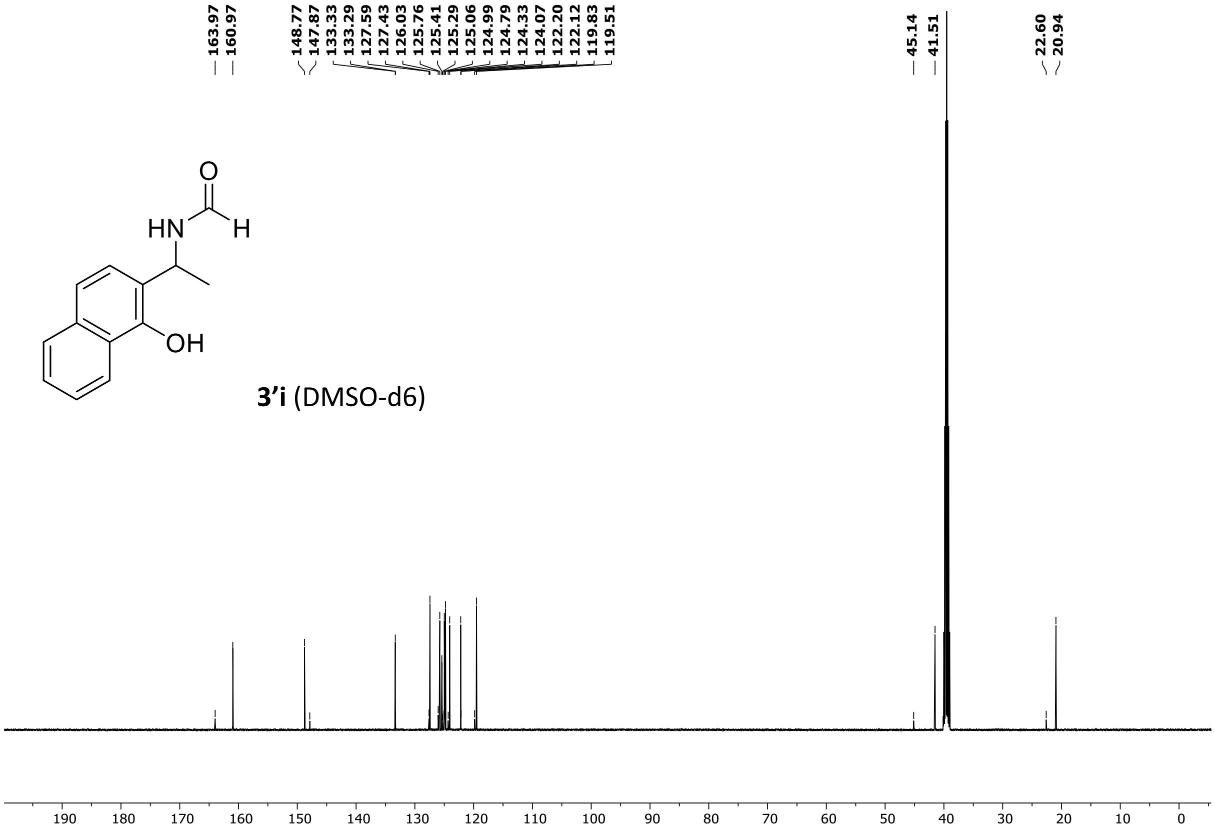

3'i (DMSO-d6)

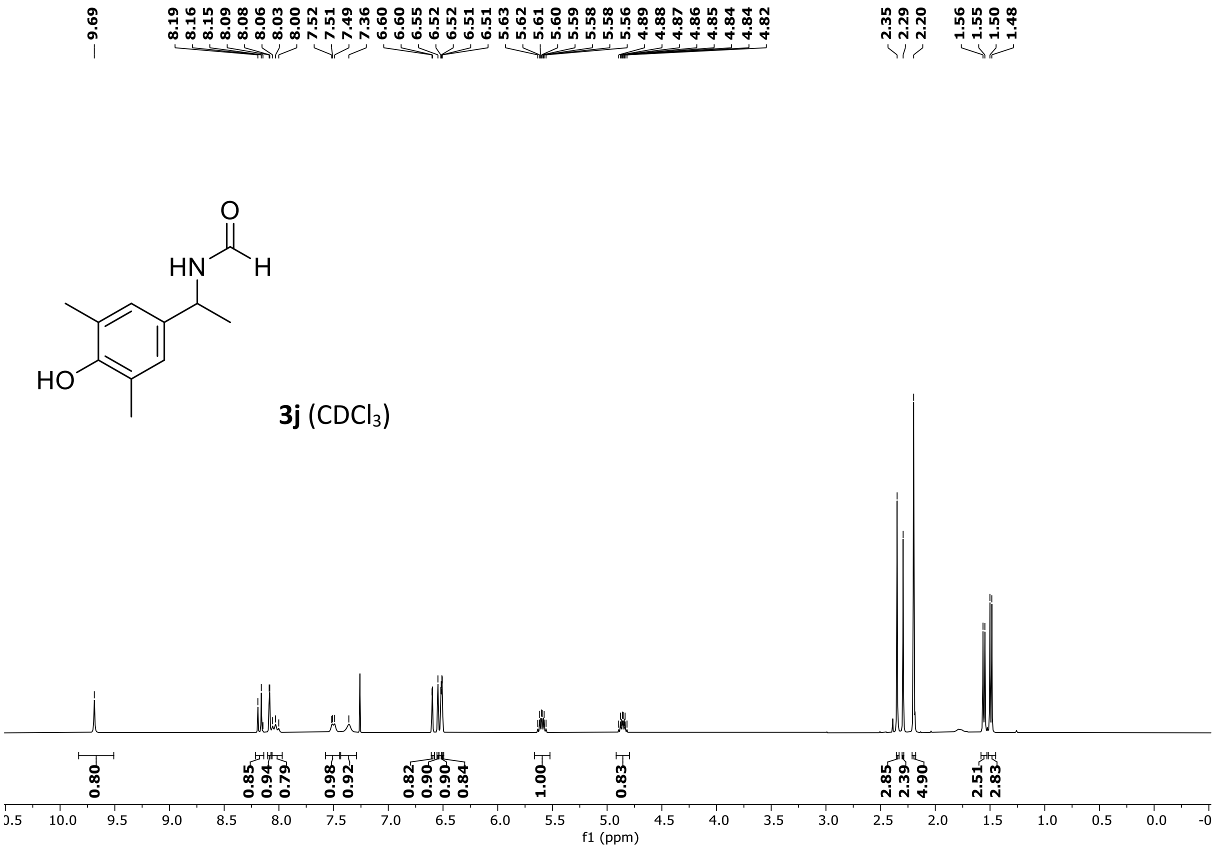
9.69
8.19 8.16 8.15 8.09 8.08 8.06 8.03 8.00 7.52 7.51 7.49 7.36 6.60 6.55 6.52 6.52 6.51 6.51 5.63 5.62 5.61 5.60 5.59 5.58 5.58 5.56 4.89 4.88 4.87 4.86 4.85 4.84 4.84 4.82
2.35 2.29 2.20
1.56 1.55 1.50 1.48
O
HN
H
HO
3j (CDCl3)
0.80
0.85 0.94 0.79
0.98 0.92
0.82 0.90 0.90 0.84
1.00
0.83
2.85 2.39 4.90
2.51 2.83
f1 (ppm)

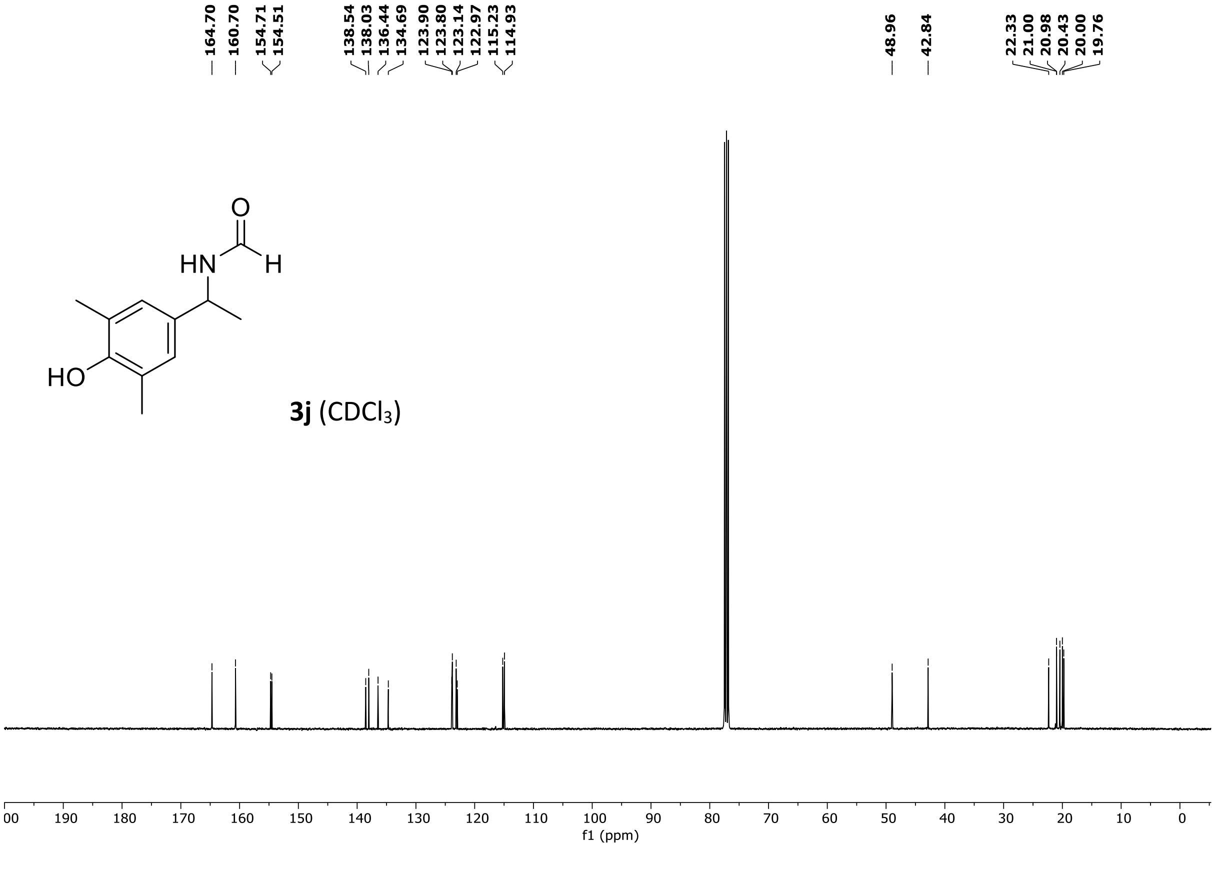
164.70
160.70
154.71 154.51
138.54 138.03 136.44 134.69 123.90 123.80 123.14 122.97 115.23 114.93
48.96
42.84
22.33 21.00 20.98 20.43 20.00 19.76
O
HN
H
HO
3j (CDCl3)
f1 (ppm)

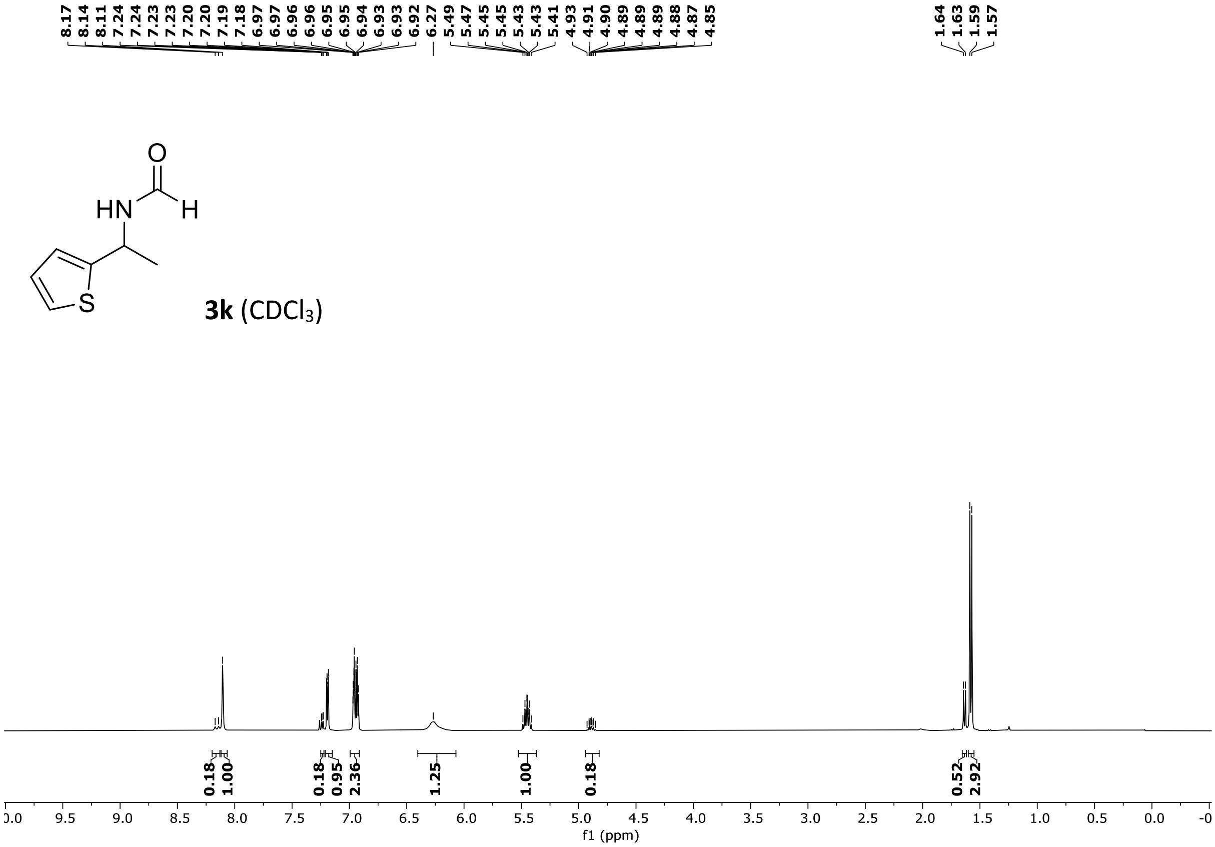

3k ($CDCl_3$)

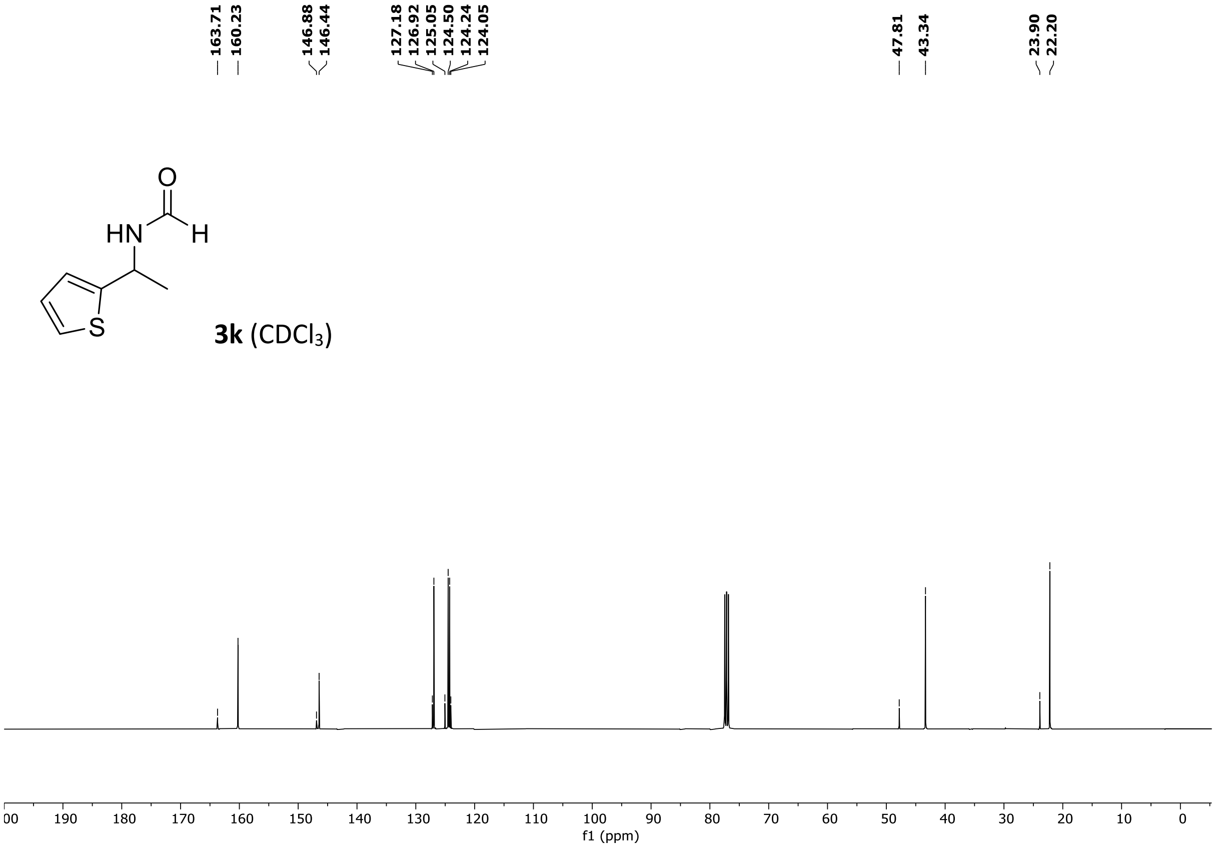

3k ($CDCl_3$)

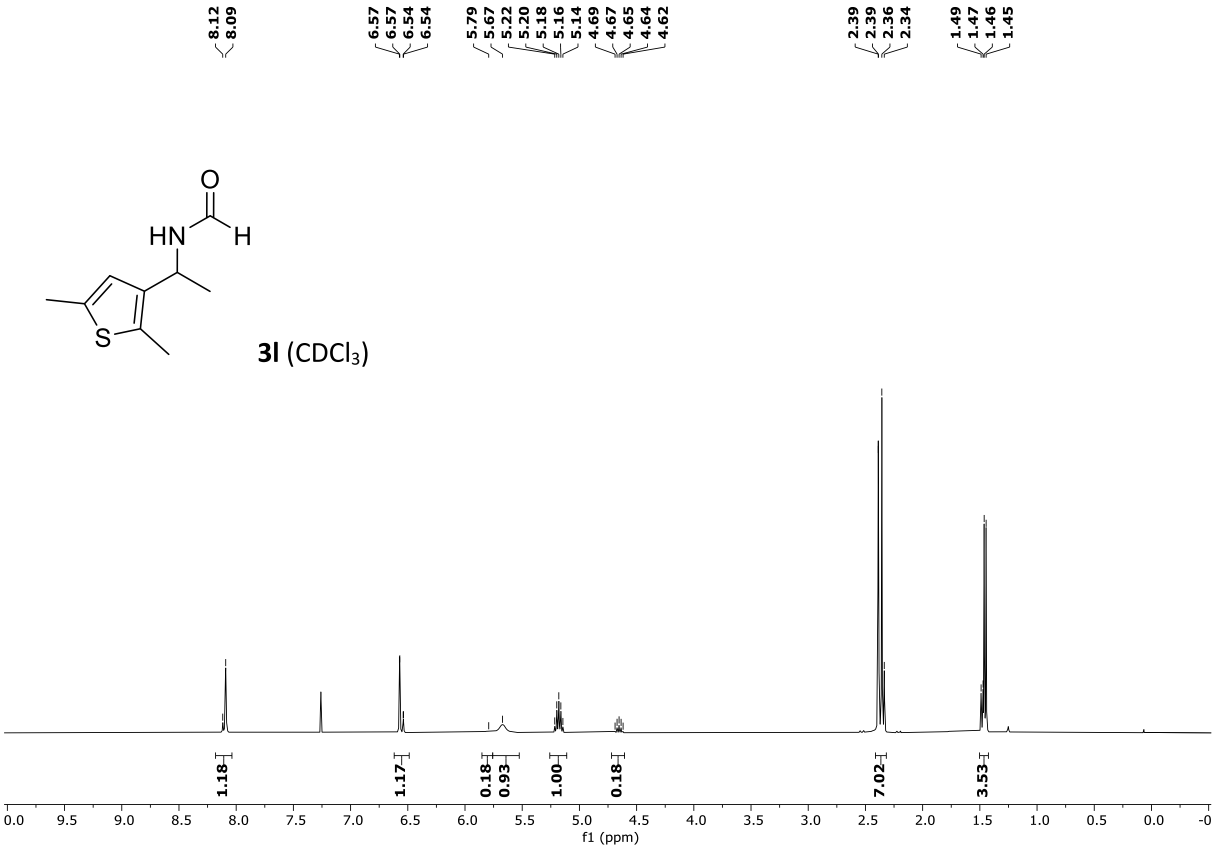
8.12
8.09
6.57
6.57
6.54
6.54
5.79
5.67
5.22
5.20
5.18
5.16
5.14
4.69
4.67
4.65
4.64
4.62
2.39
2.39
2.36
2.34
1.49
1.47
1.46
1.45
O
HN
H
S
3l (CDCl3)
1.18
1.17
0.18
0.93
1.00
0.18
7.02
3.53
f1 (ppm)

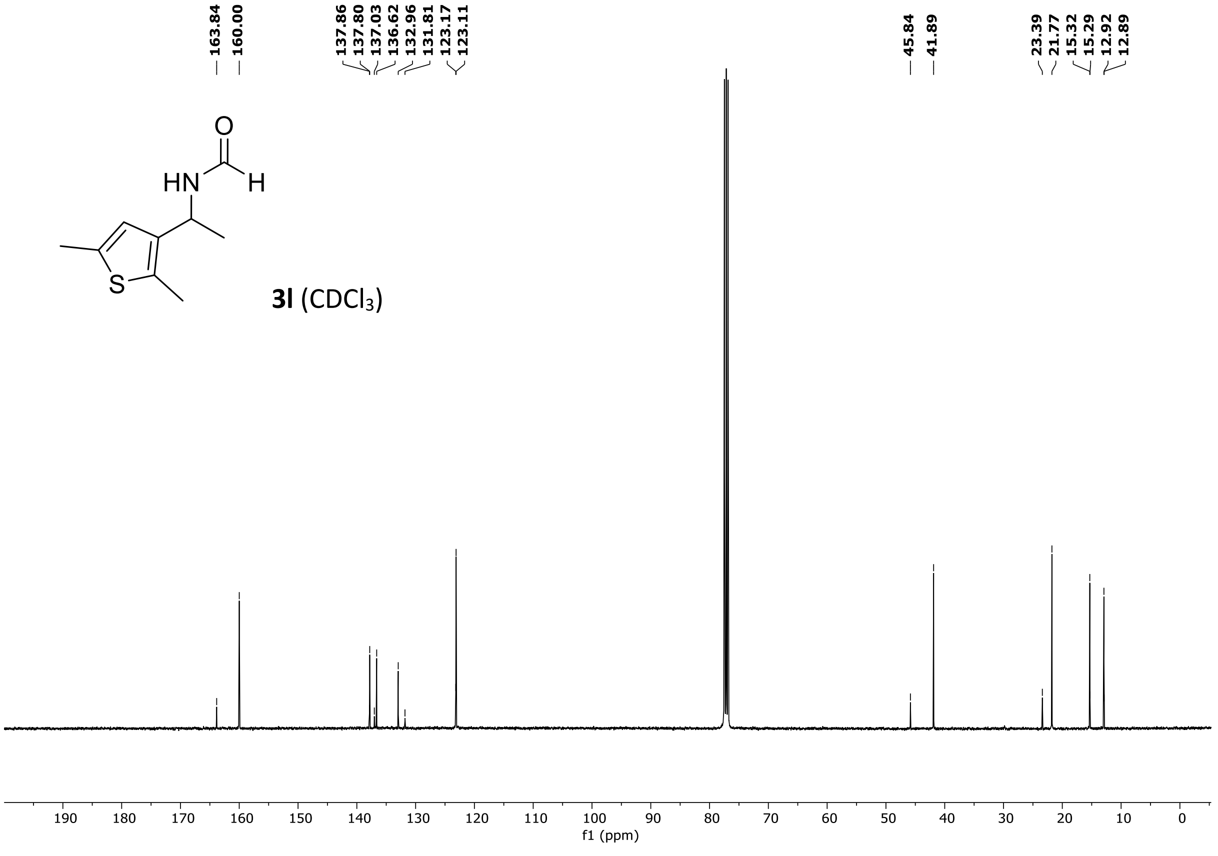
163.84
160.00
137.86
137.80
137.03
136.62
132.96
131.81
123.17
123.11
45.84
41.89
23.39
21.77
15.32
15.29
12.92
12.89
O
HN
H
S
3l (CDCl3)
f1 (ppm)

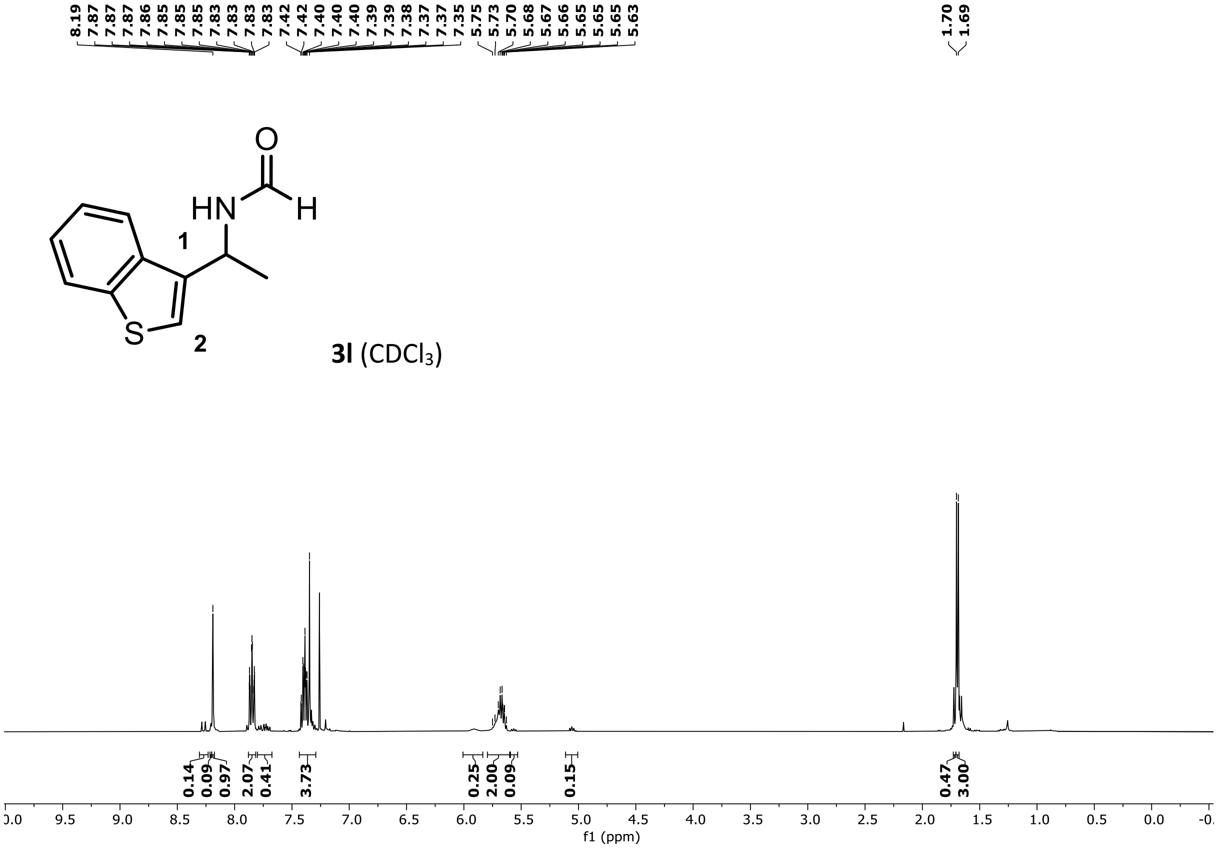
8.19 7.87 7.87 7.86 7.85 7.85 7.83 7.83 7.83 7.83 7.42 7.40 7.40 7.39 7.39 7.38 7.37 7.37 7.35 5.75 5.73 5.70 5.68 5.67 5.66 5.65 5.65 5.63
1.70 1.69
O
HN
H
1
S
2
3l (CDCl$_3$)
0.14 0.09 0.97 2.07 0.41 3.73 0.25 2.00 0.09 0.15 0.47 3.00
f1 (ppm)

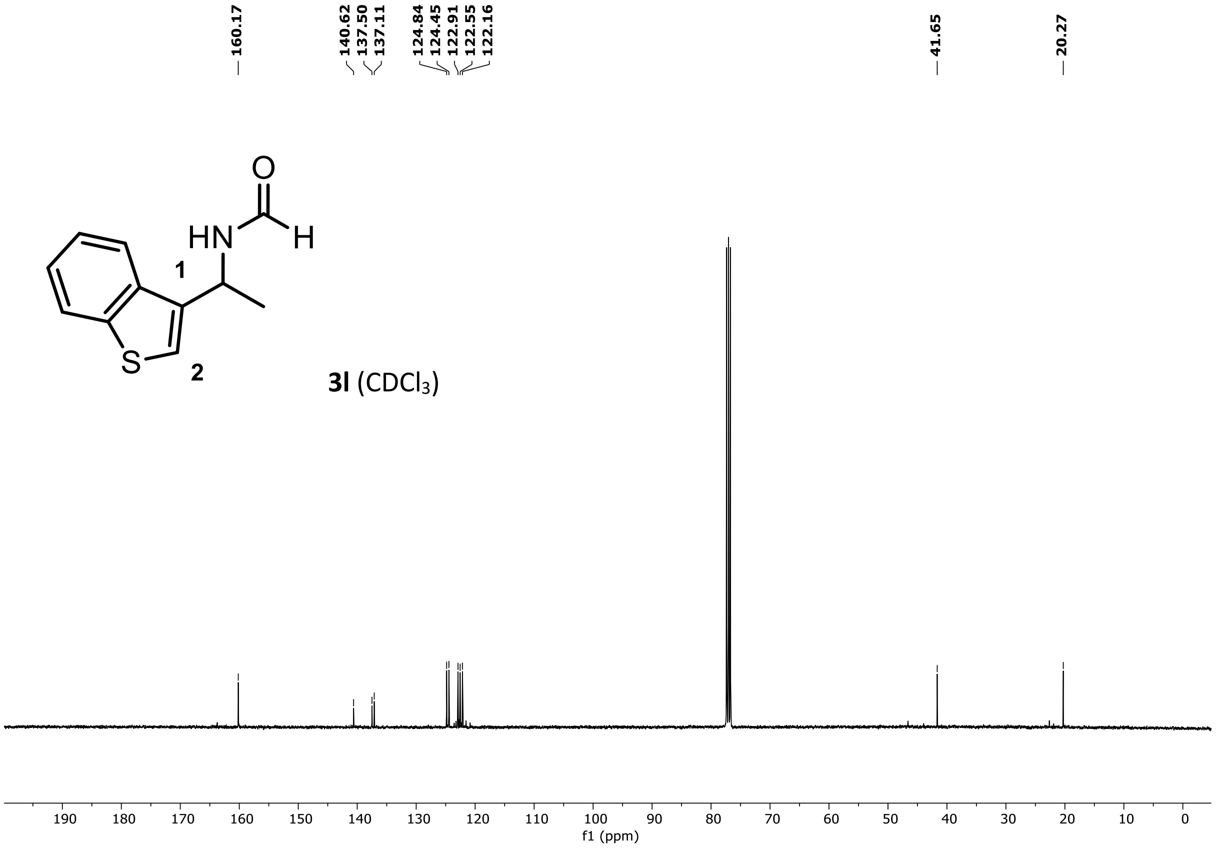
160.17
140.62 137.50 137.11
124.84 124.45 122.91 122.55 122.16
41.65
20.27
O
HN
H
1
S
2
3l (CDCl$_3$)
f1 (ppm)

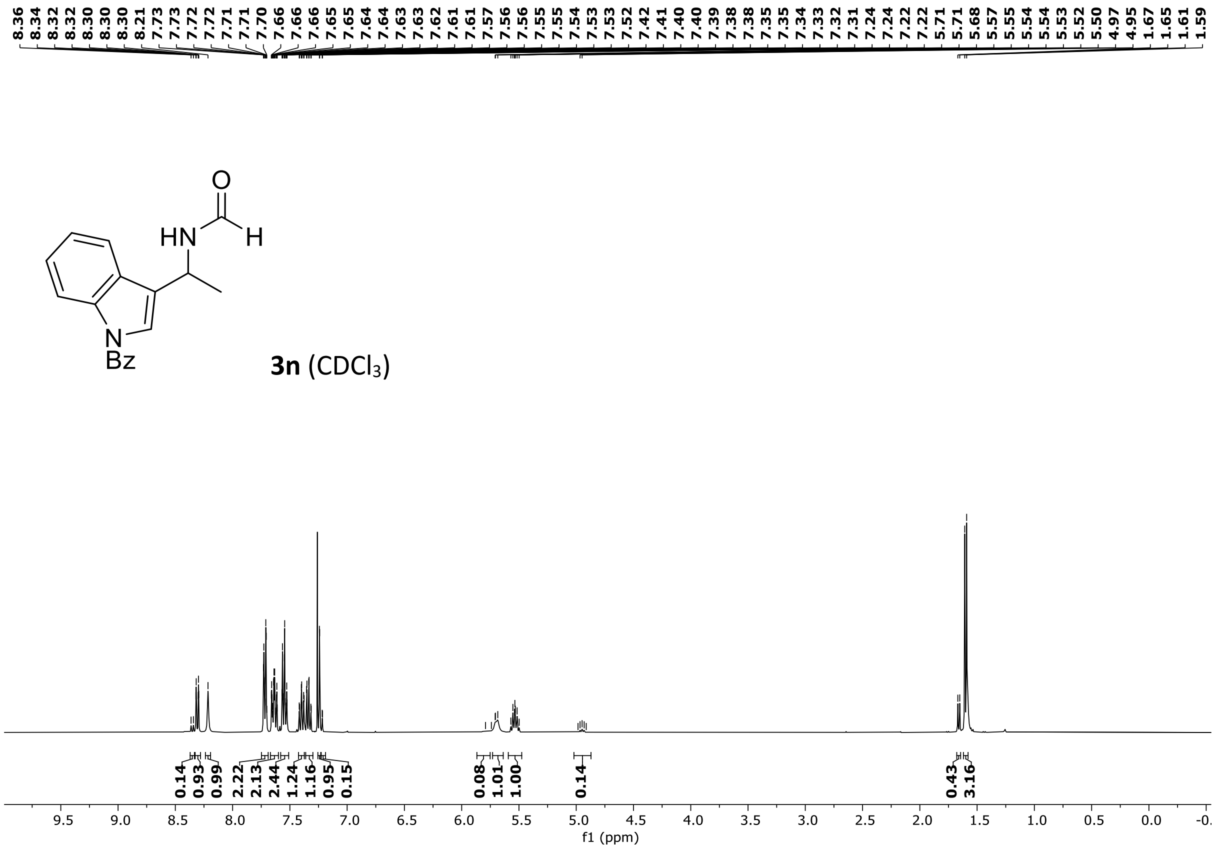
8.36 8.34 8.32 8.32 8.30 8.30 8.21 7.73 7.73 7.72 7.72 7.71 7.71 7.70 7.66 7.66 7.66 7.65 7.64 7.63 7.63 7.62 7.61 7.61 7.57 7.56 7.56 7.55 7.55 7.54 7.53 7.53 7.52 7.42 7.41 7.40 7.40 7.39 7.38 7.38 7.35 7.35 7.34 7.33 7.32 7.31 7.24 7.24 7.24 7.22 7.22 5.71 5.68 5.57 5.55 5.54 5.54 5.53 5.52 5.50 4.97 4.95 1.67 1.65 1.61 1.59
O
HN
H
N
Bz
3n (CDCl3)
0.14 0.93 0.99 2.22 2.13 2.44 1.24 1.16 0.95 0.15 0.08 1.01 1.00 0.14 0.43 3.16
9.5 9.0 8.5 8.0 7.5 7.0 6.5 6.0 5.5 5.0 4.5 4.0 3.5 3.0 2.5 2.0 1.5 1.0 0.5 0.0
f1 (ppm)

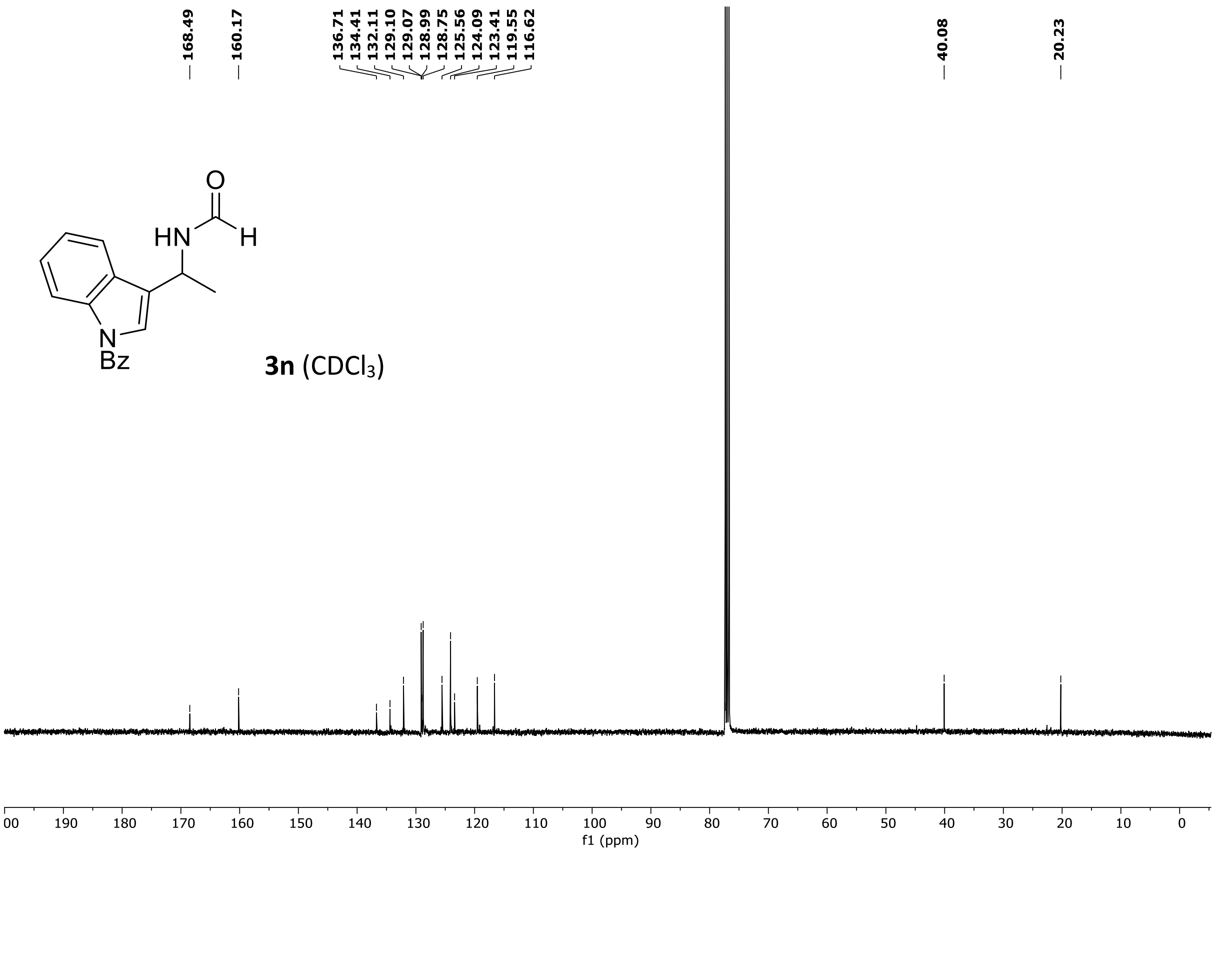
168.49 160.17 136.71 134.41 132.11 129.10 129.07 128.99 128.75 125.56 124.09 123.41 119.55 116.62 40.08 20.23
O
HN
H
N
Bz
3n (CDCl3)
190 180 170 160 150 140 130 120 110 100 90 80 70 60 50 40 30 20 10 0
f1 (ppm)

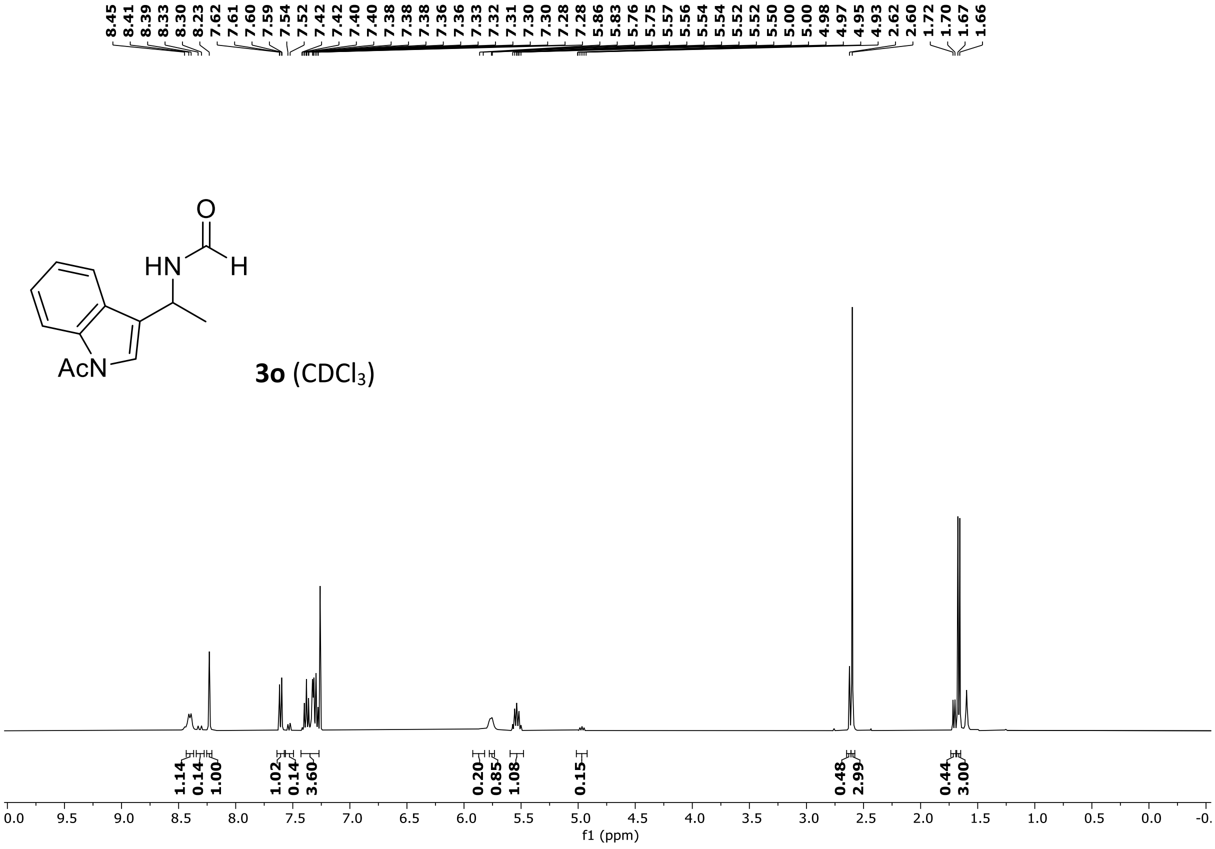
8.45 8.41 8.39 8.33 8.30 8.23 7.62 7.61 7.60 7.59 7.54 7.52 7.42 7.42 7.40 7.40 7.38 7.38 7.36 7.36 7.33 7.32 7.31 7.30 7.30 7.28 7.28 5.86 5.83 5.76 5.75 5.57 5.56 5.54 5.54 5.52 5.52 5.50 5.00 5.00 4.98 4.97 4.95 4.93 2.62 2.60 1.72 1.70 1.67 1.66
HN
O
H
AcN
3o (CDCl3)
1.14 0.14 1.00 1.02 0.14 3.60 0.20 0.85 1.08 0.15 0.48 2.99 0.44 3.00
f1 (ppm)

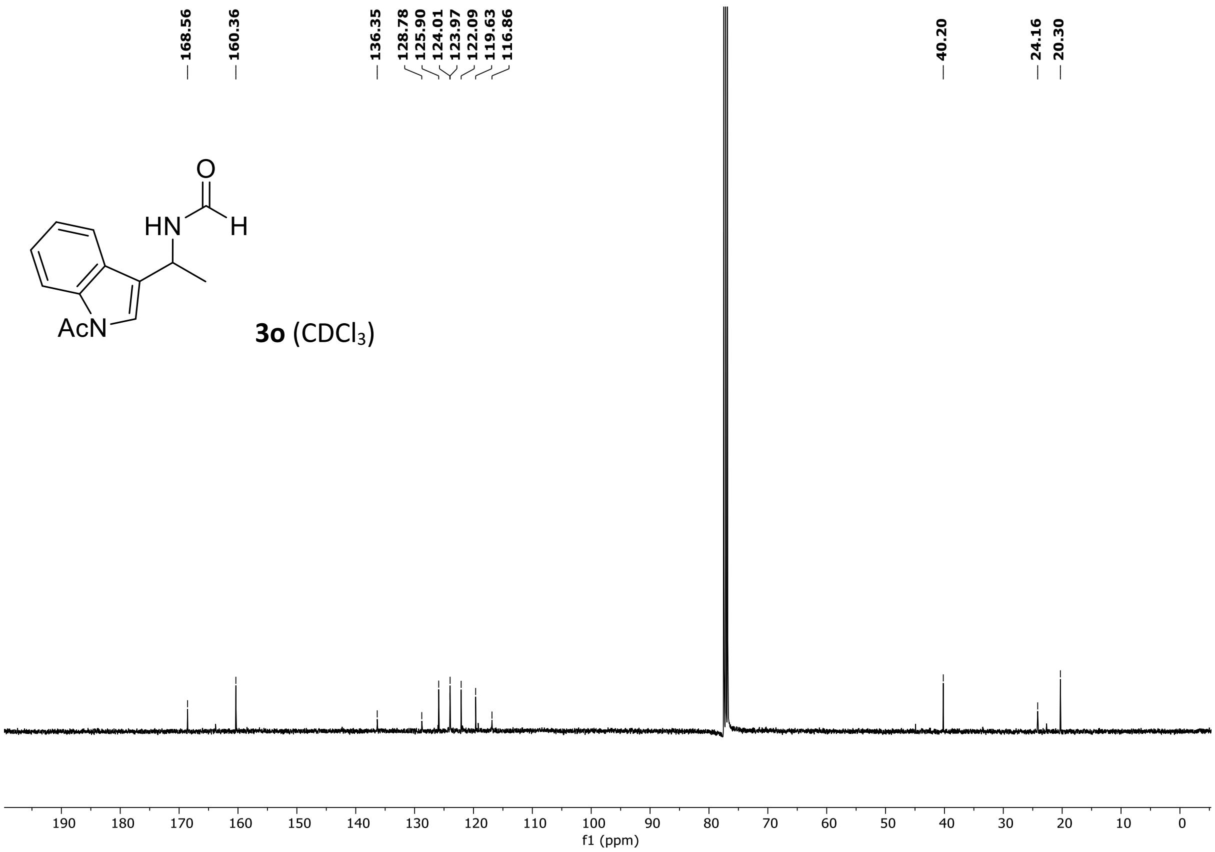
168.56 160.36 136.35 128.78 125.90 124.01 123.97 122.09 119.63 116.86 40.20 24.16 20.30
HN
O
H
AcN
3o (CDCl3)
f1 (ppm)

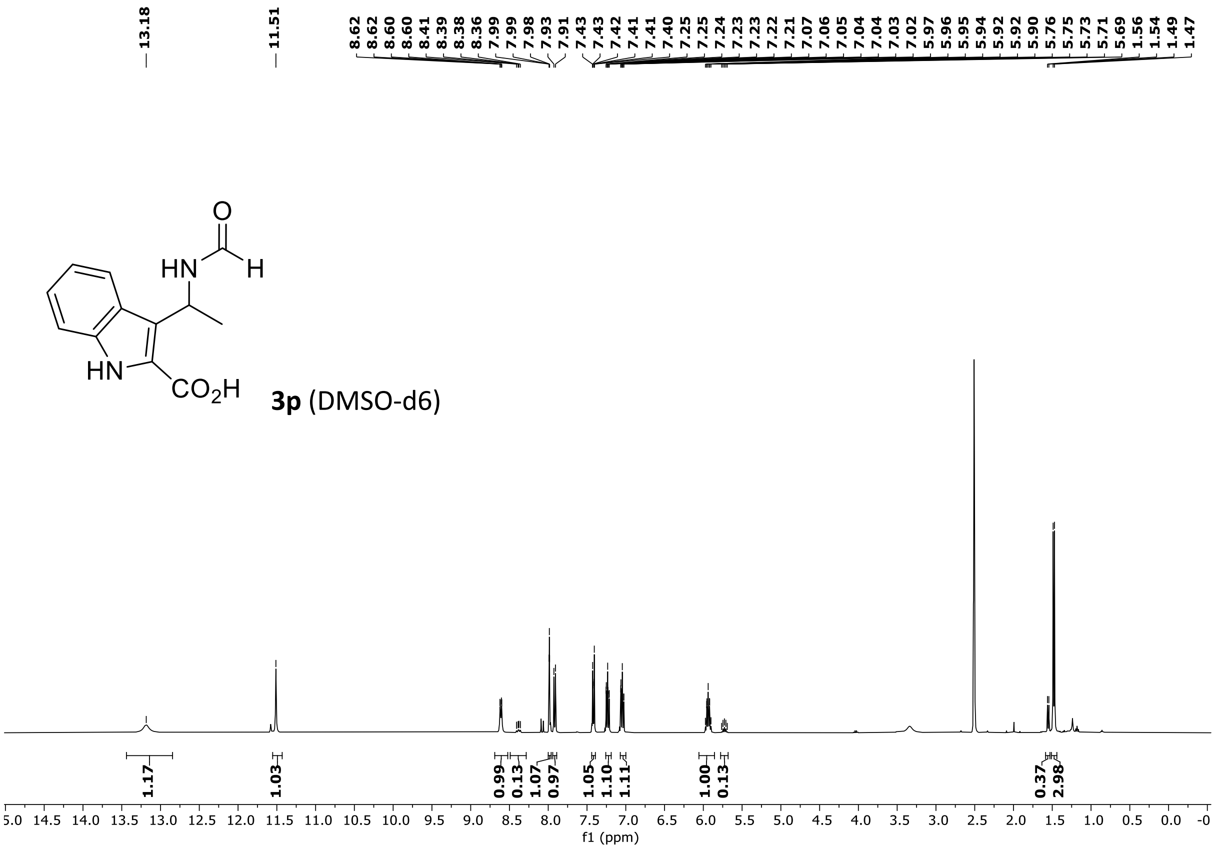
13.18
11.51
8.62 8.62 8.60 8.60 8.41 8.39 8.38 8.36 7.99 7.99 7.98 7.93 7.91 7.43 7.43 7.42 7.41 7.41 7.40 7.25 7.25 7.24 7.23 7.23 7.22 7.21 7.07 7.06 7.05 7.04 7.04 7.03 7.02 5.97 5.96 5.95 5.94 5.92 5.92 5.90 5.76 5.75 5.73 5.71 5.69 1.56 1.54 1.49 1.47
3p (DMSO-d6)
1.17
1.03
0.99 0.13 1.07 0.97
1.05 1.10 1.11
1.00 0.13
0.37 2.98
f1 (ppm)

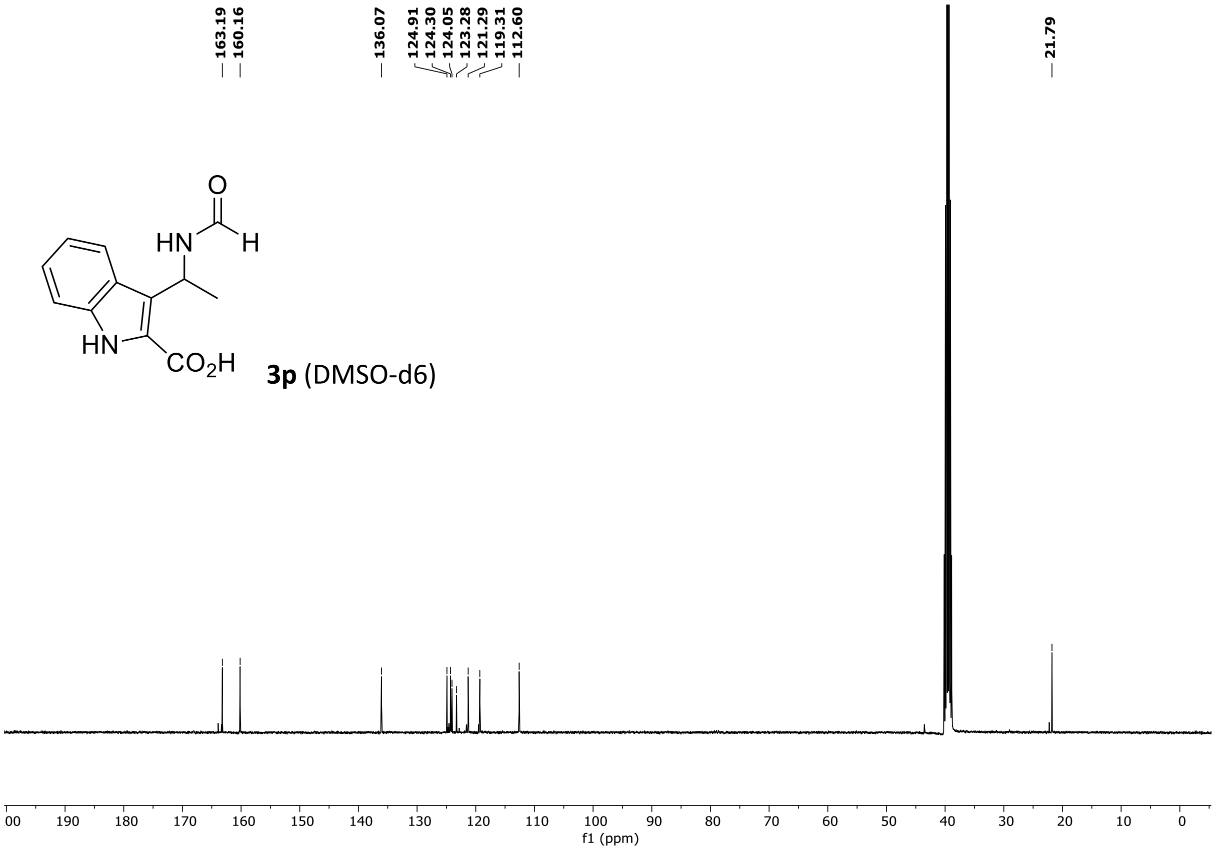
163.19
160.16
136.07
124.91 124.30 124.05 123.28 121.29 119.31 112.60
21.79
3p (DMSO-d6)
f1 (ppm)

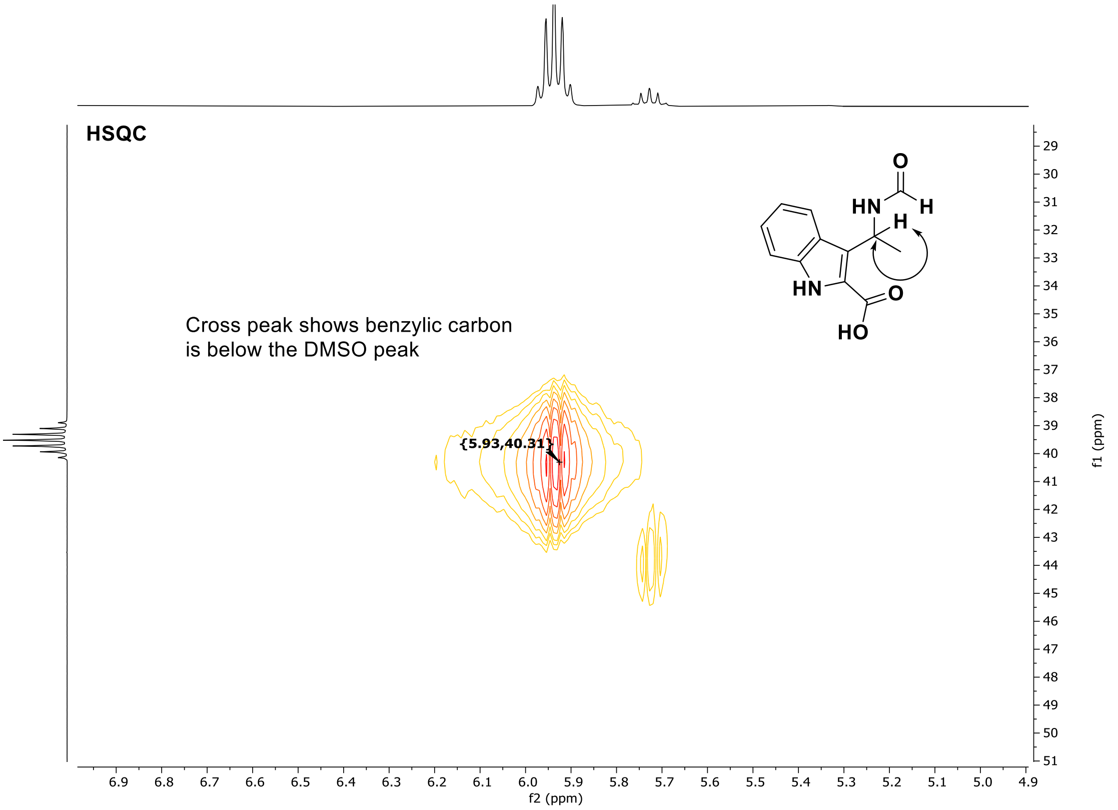
HSQC
Cross peak shows benzylic carbon
is below the DMSO peak
{5.93,40.31}
f2 (ppm)
f1 (ppm)

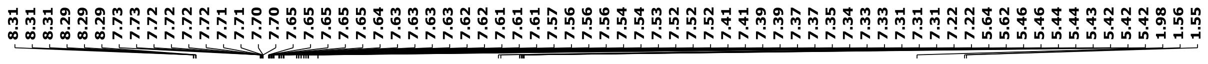
8.31 8.31 8.31 8.29 8.29 8.29 7.73 7.73 7.72 7.72 7.72 7.72 7.71 7.71 7.70 7.70 7.65 7.65 7.65 7.65 7.64 7.63 7.63 7.63 7.62 7.62 7.61 7.61 7.61 7.57 7.56 7.56 7.54 7.54 7.53 7.52 7.52 7.52 7.41 7.41 7.39 7.39 7.37 7.37 7.35 7.34 7.33 7.33 7.31 7.31 7.31 7.22 7.22 5.64 5.62 5.46 5.46 5.44 5.43 5.42 5.42 5.42 1.98 1.56 1.55

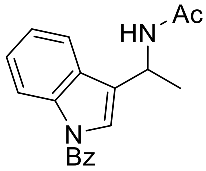
HN
Ac
N
Bz

3q (CDCl$_3$)

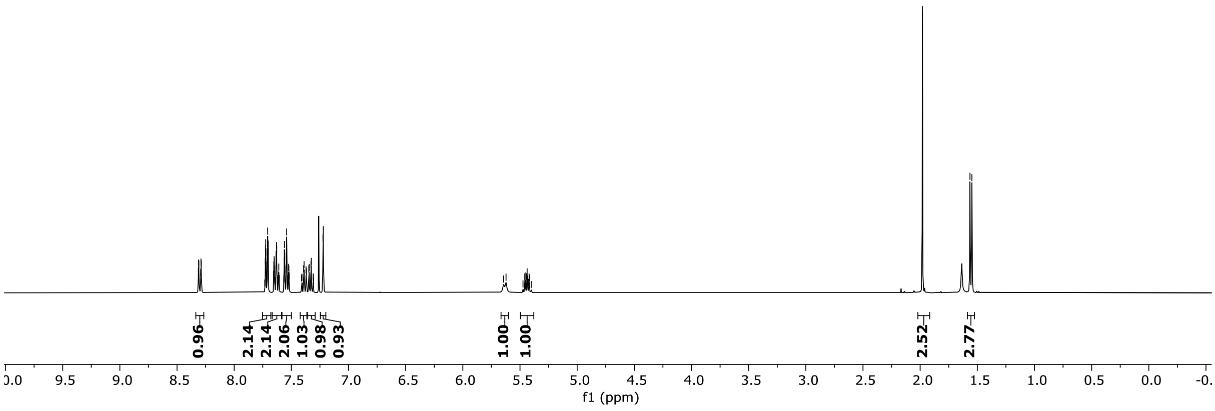
0.96
2.14
2.14
2.06
1.03
0.98
0.93
1.00
1.00
2.52
2.77
f1 (ppm)

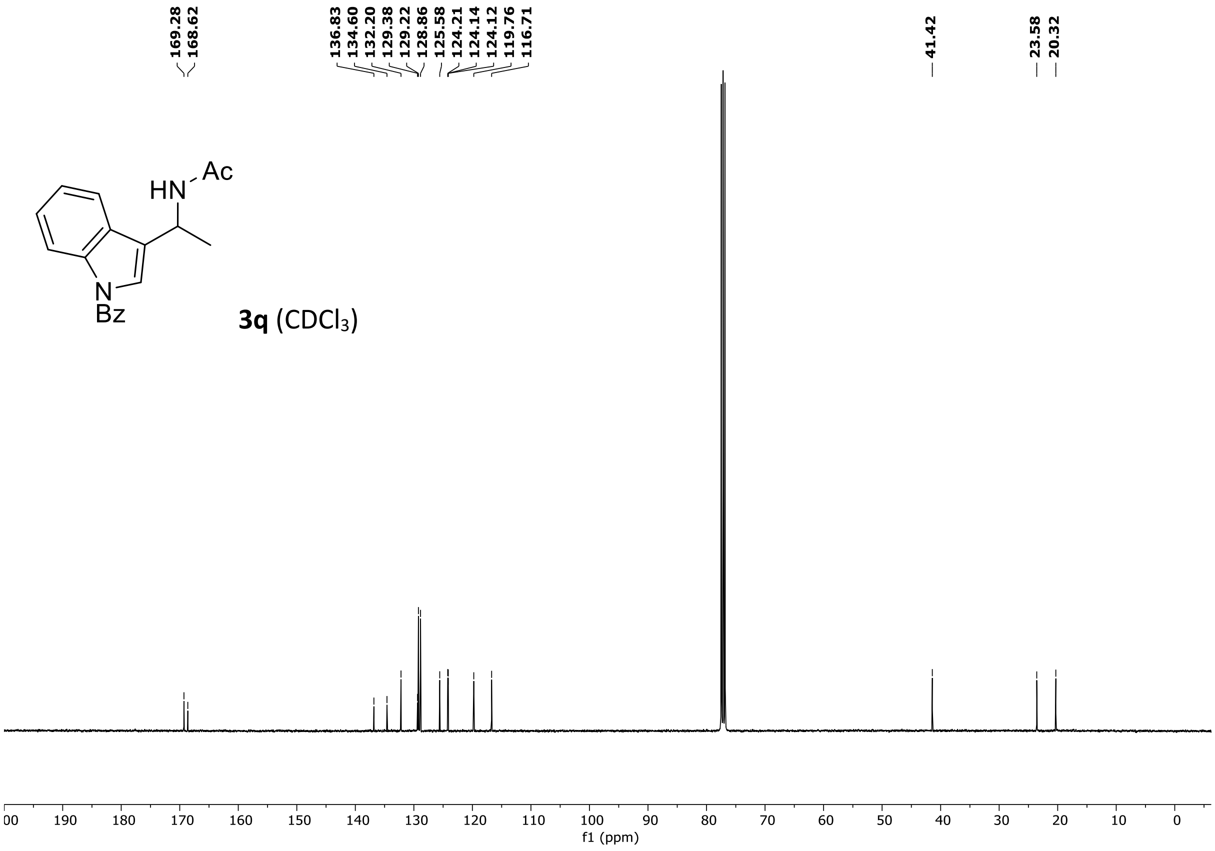

3q ($CDCl_3$)

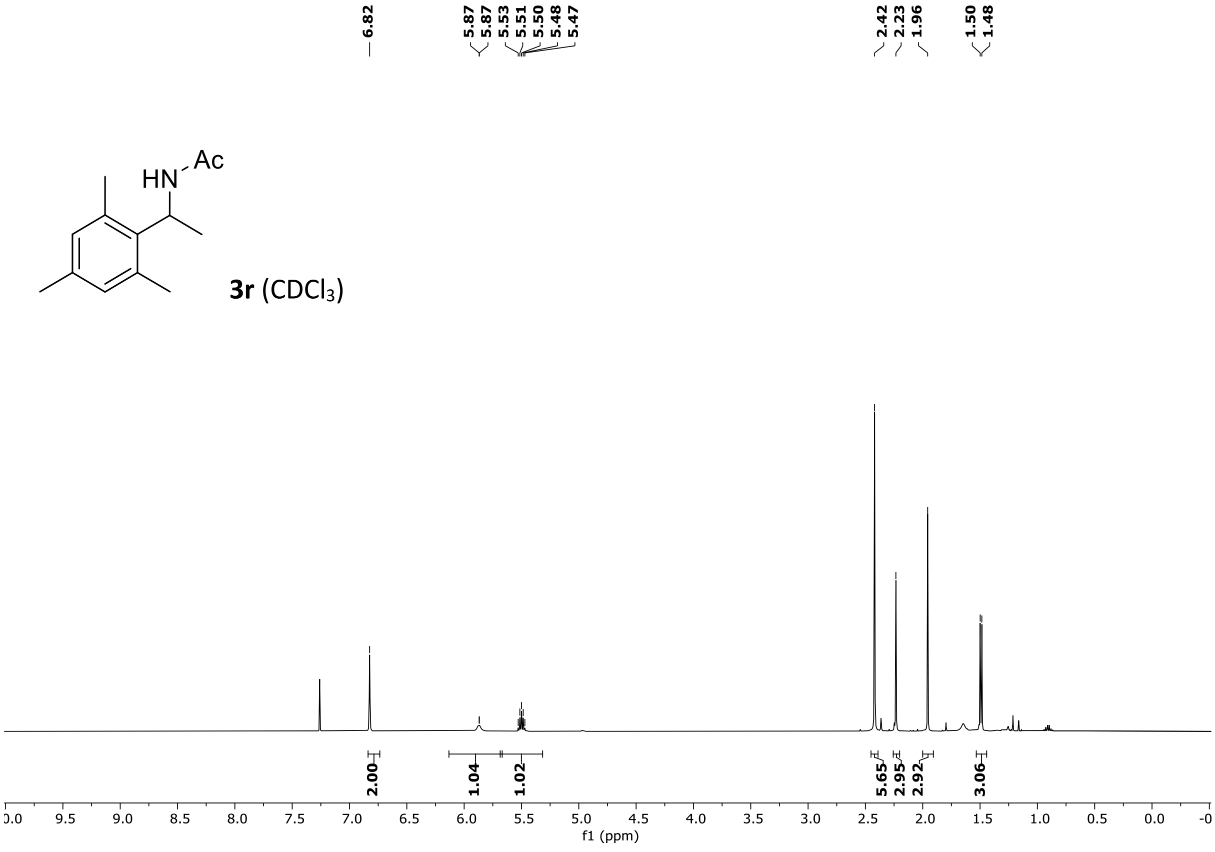

3r ($CDCl_3$)

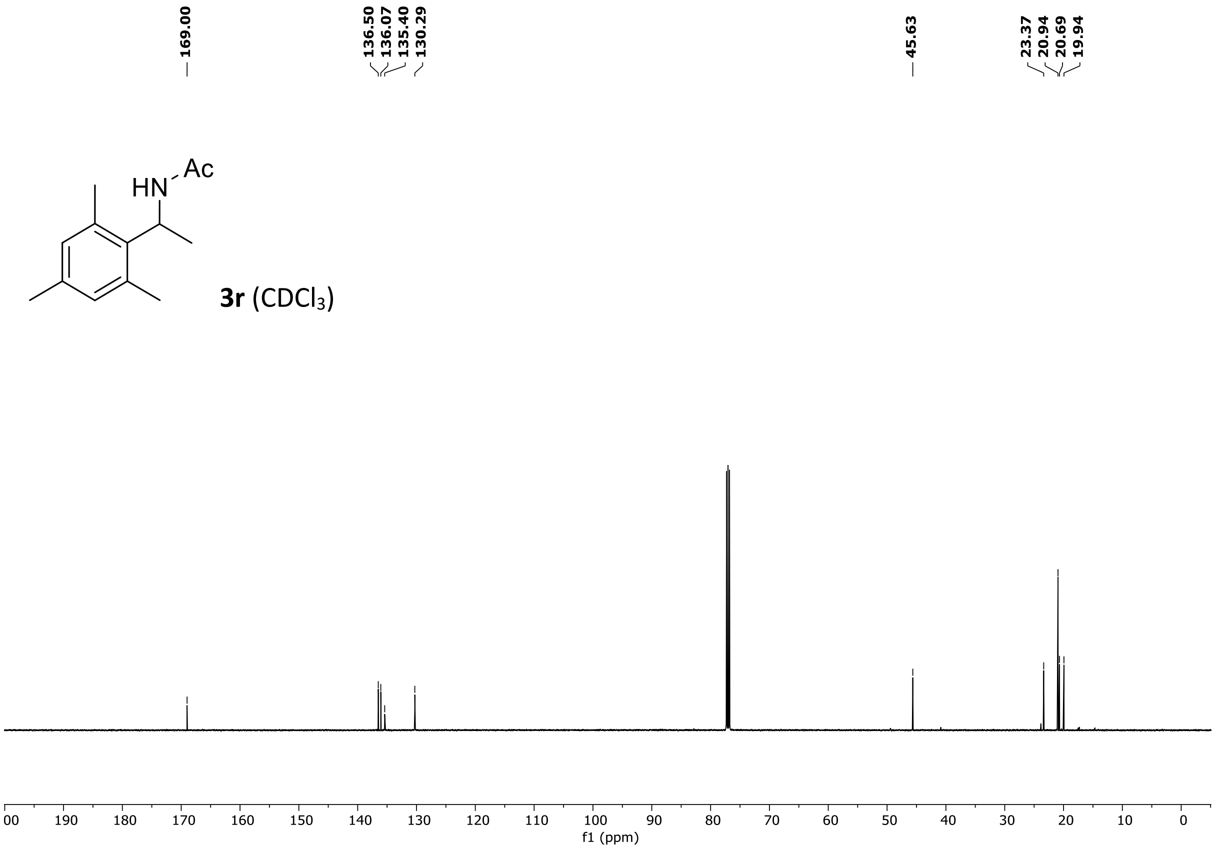
169.00
136.50
136.07
135.40
130.29
45.63
23.37
20.94
20.69
19.94
HN
Ac
3r (CDCl3)
f1 (ppm)

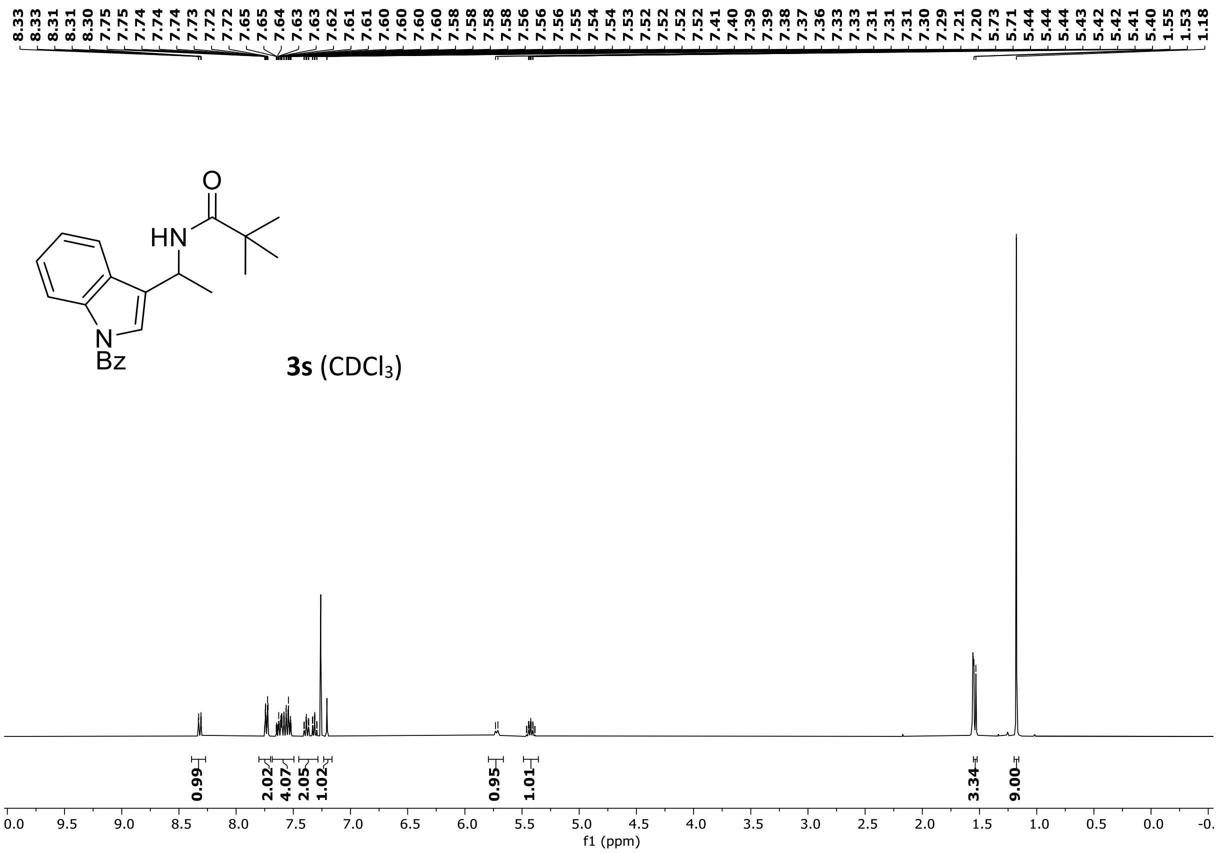
8.33 8.33 8.31 8.31 8.30 7.75 7.75 7.74 7.74 7.74 7.73 7.72 7.72 7.65 7.65 7.64 7.63 7.63 7.62 7.61 7.61 7.60 7.60 7.60 7.60 7.58 7.58 7.58 7.58 7.56 7.56 7.56 7.55 7.54 7.54 7.53 7.53 7.52 7.52 7.52 7.52 7.52 7.41 7.40 7.39 7.39 7.38 7.37 7.36 7.33 7.33 7.31 7.31 7.31 7.30 7.29 7.21 7.20 5.73 5.71 5.44 5.44 5.44 5.43 5.42 5.42 5.41 5.40 1.55 1.53 1.18
O
HN
N
Bz
3s (CDCl3)
0.99
2.02
4.07
2.05
1.02
0.95
1.01
3.34
9.00
f1 (ppm)

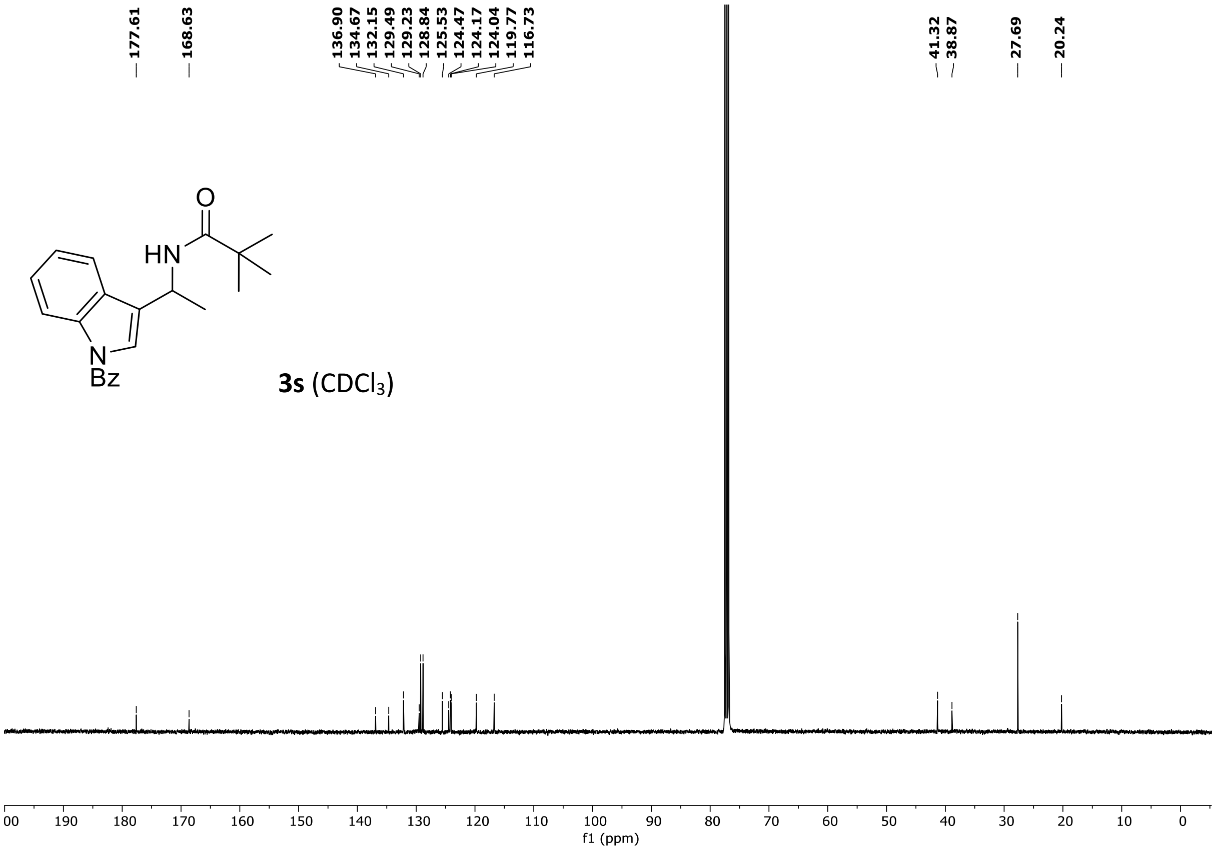

177.61
168.63
136.90
134.67
132.15
129.49
129.23
128.84
125.53
124.47
124.17
124.04
119.77
116.73
41.32
38.87
27.69
20.24
O
HN
N
Bz
3s (CDCl3)
f1 (ppm)

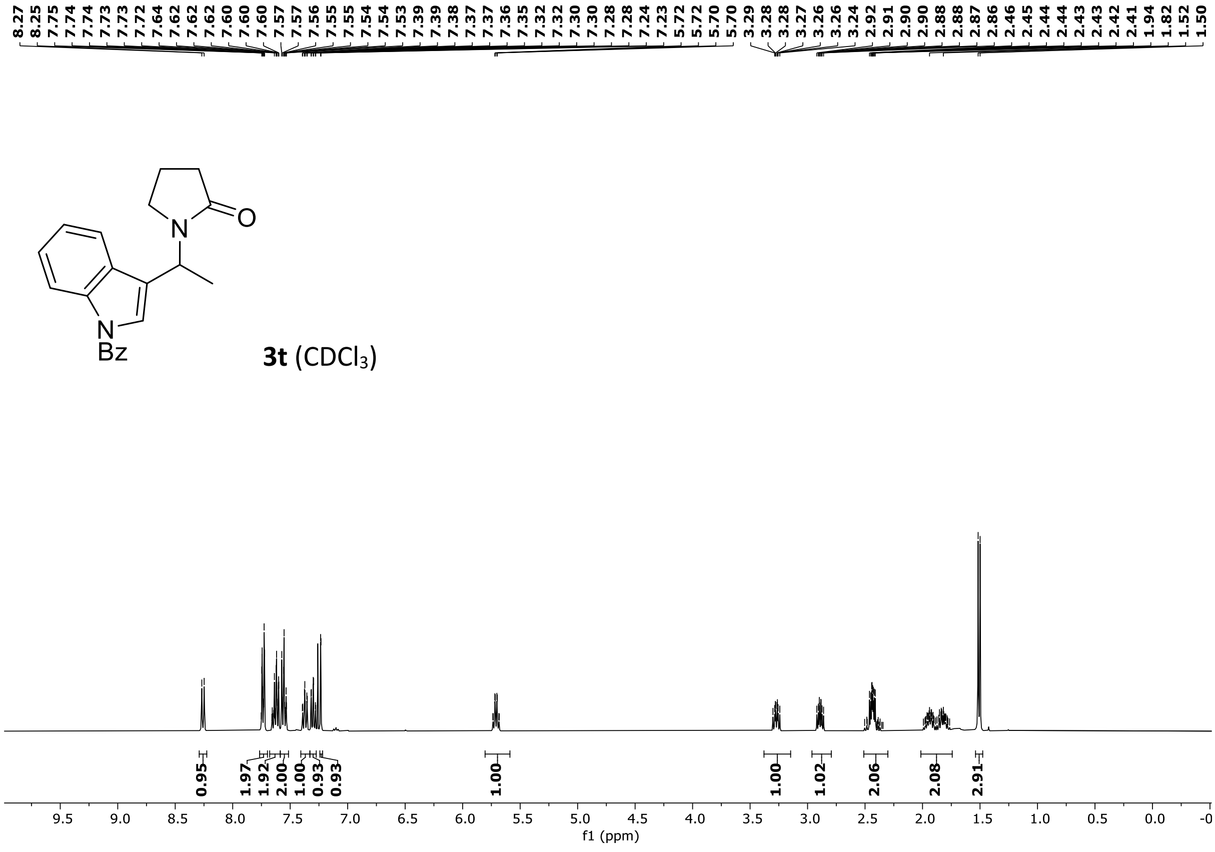

8.27 8.25 7.75 7.74 7.74 7.73 7.73 7.72 7.64 7.62 7.62 7.60 7.60 7.60 7.57 7.57 7.56 7.55 7.55 7.54 7.54 7.53 7.39 7.39 7.38 7.37 7.37 7.36 7.35 7.32 7.32 7.30 7.30 7.28 7.28 7.24 7.23 5.72 5.72 5.70 3.29 3.28 3.27 3.26 3.26 3.24 2.92 2.91 2.90 2.88 2.88 2.87 2.86 2.46 2.45 2.44 2.44 2.43 2.43 2.42 2.41 1.94 1.82 1.52 1.50
O
N
N
Bz
3t (CDCl3)
0.95
1.97
1.92
2.00
1.00
0.93
0.93
1.00
1.00
1.02
2.06
2.08
2.91
f1 (ppm)

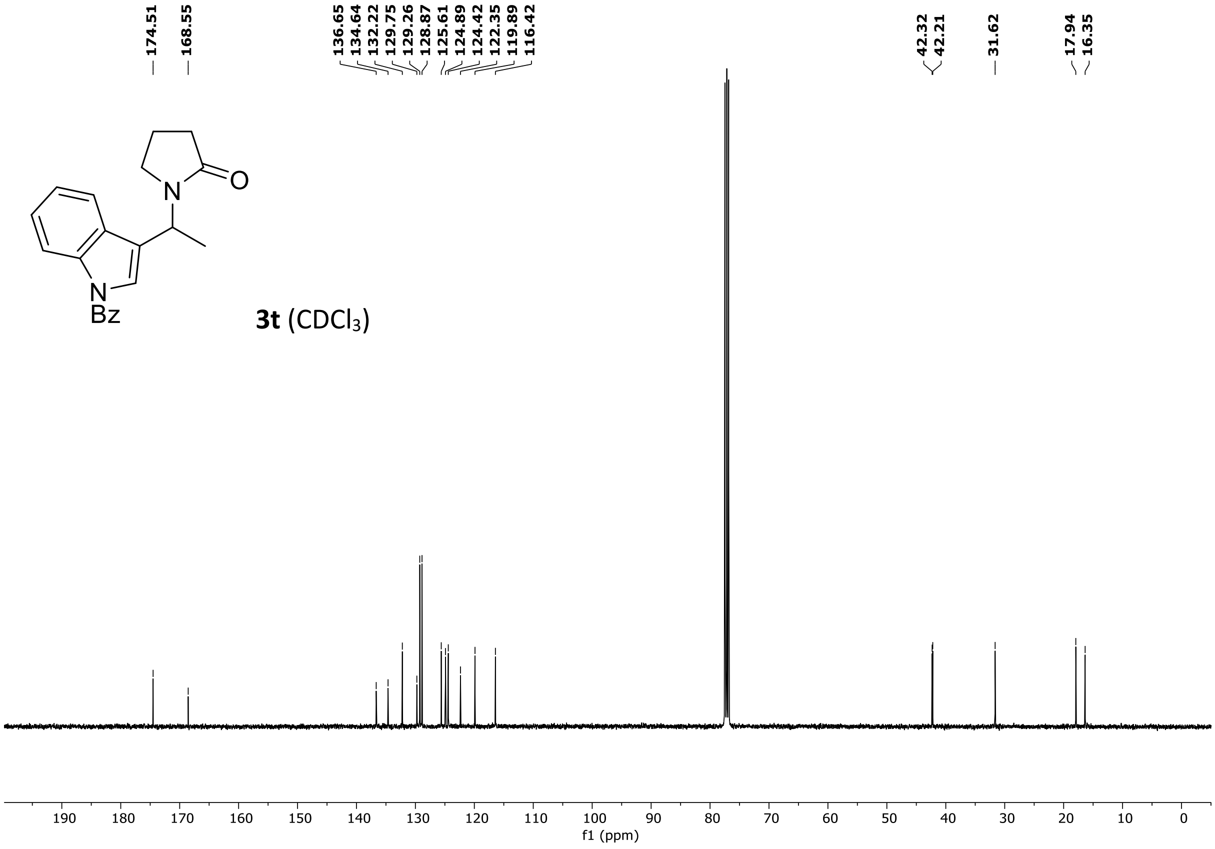

3t ($CDCl_3$)

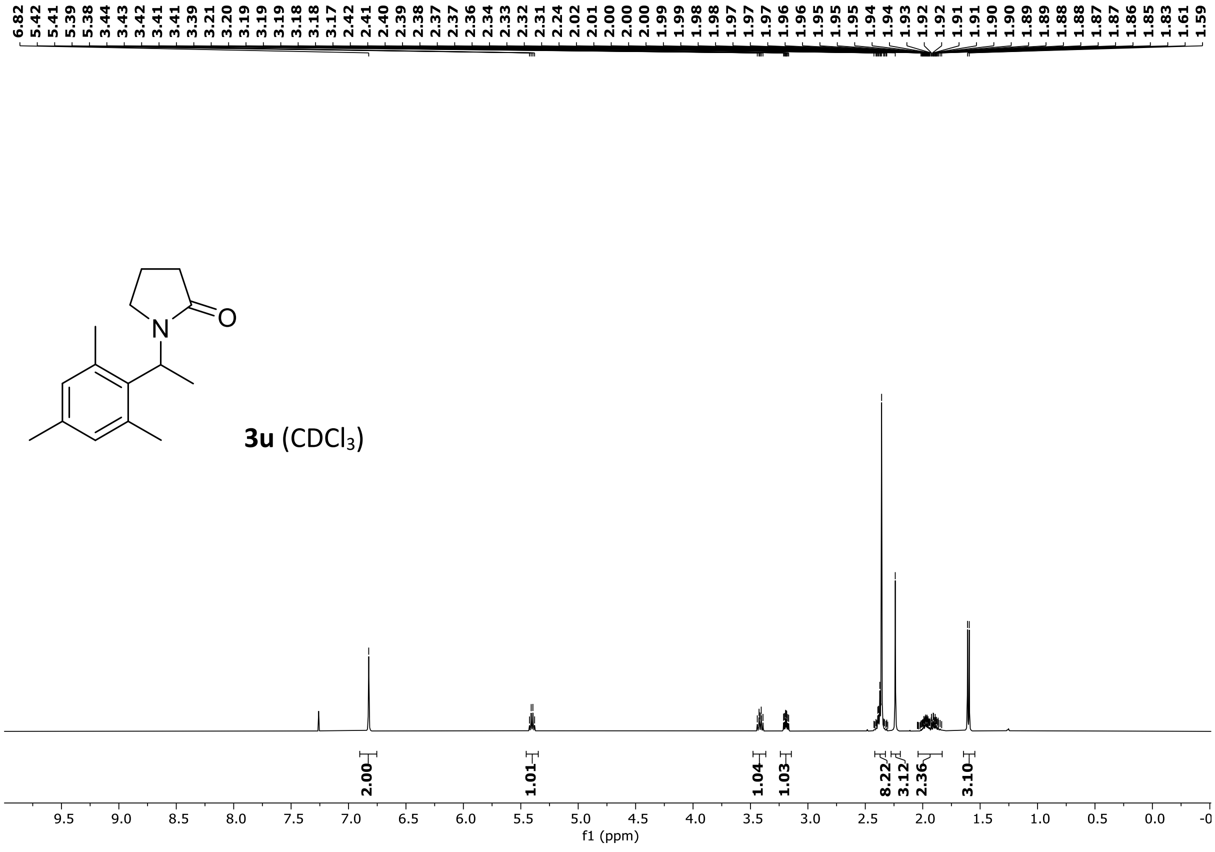

3u ($CDCl_3$)

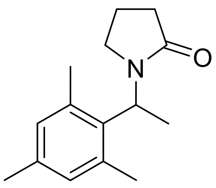

3u ($CDCl_3$)

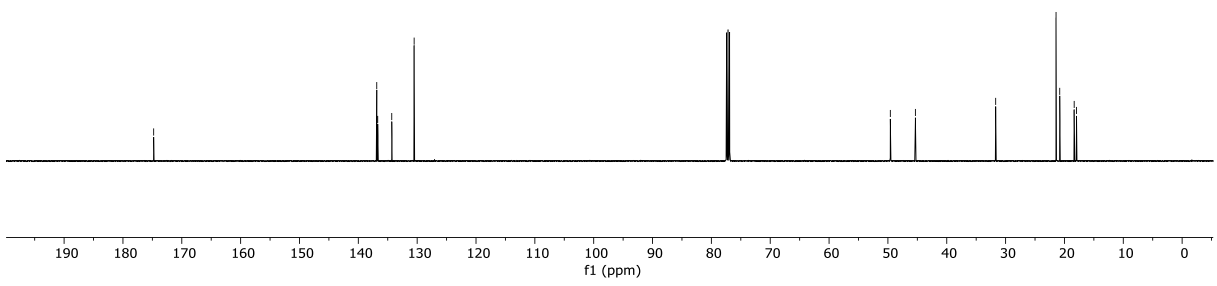

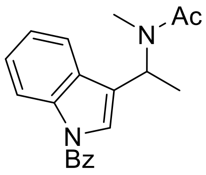

3v ($CDCl_3$)

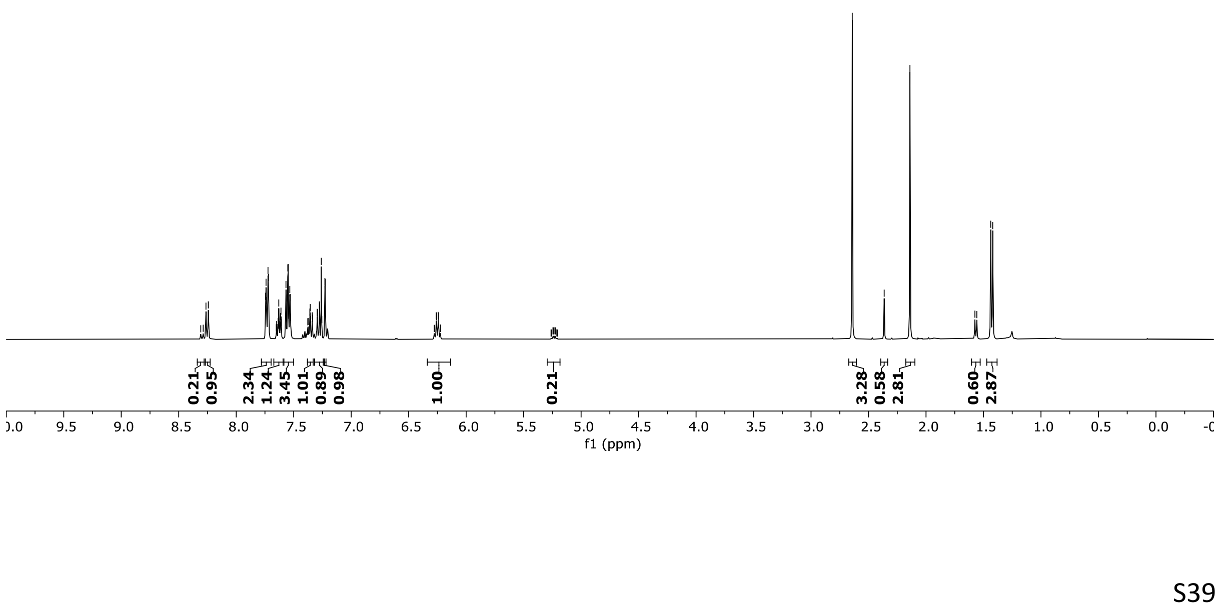

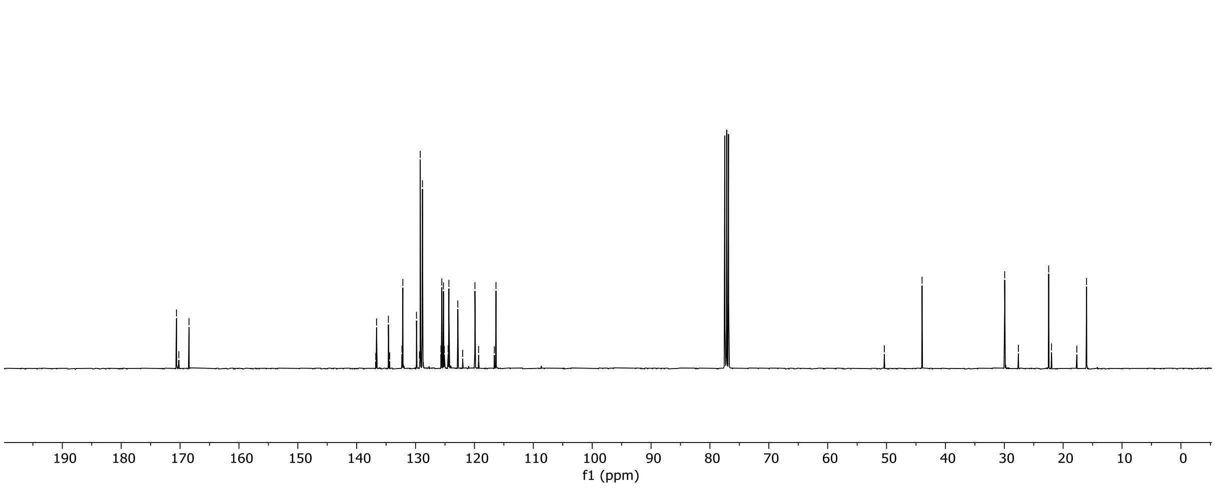

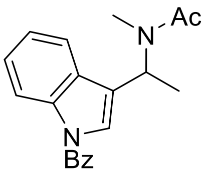

3v ($CDCl_3$)

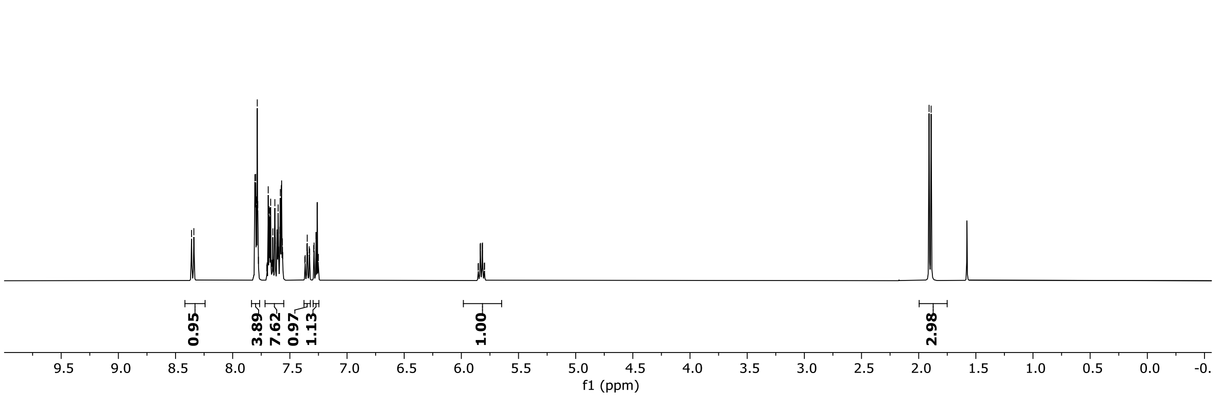

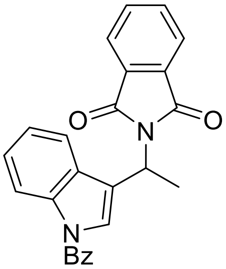

3w ($CDCl_3$)

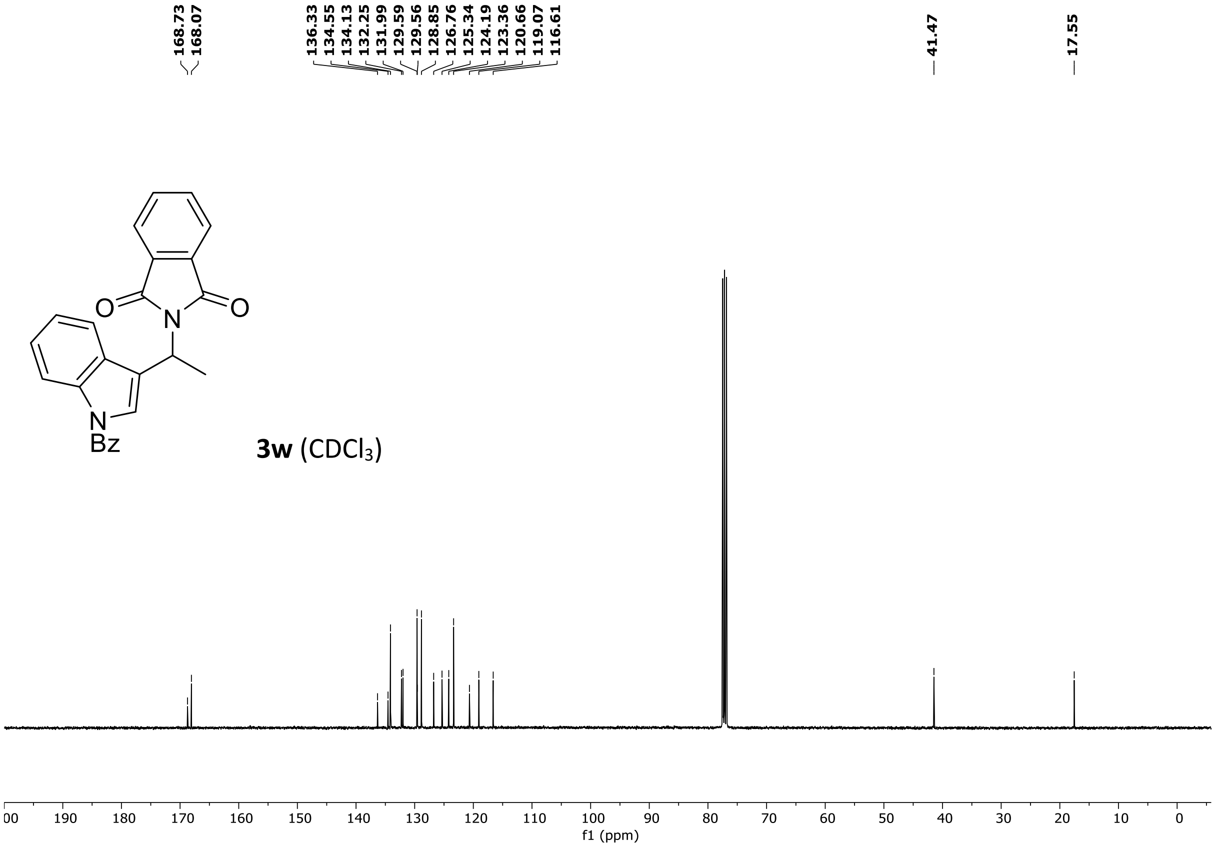

3w ($CDCl_3$)

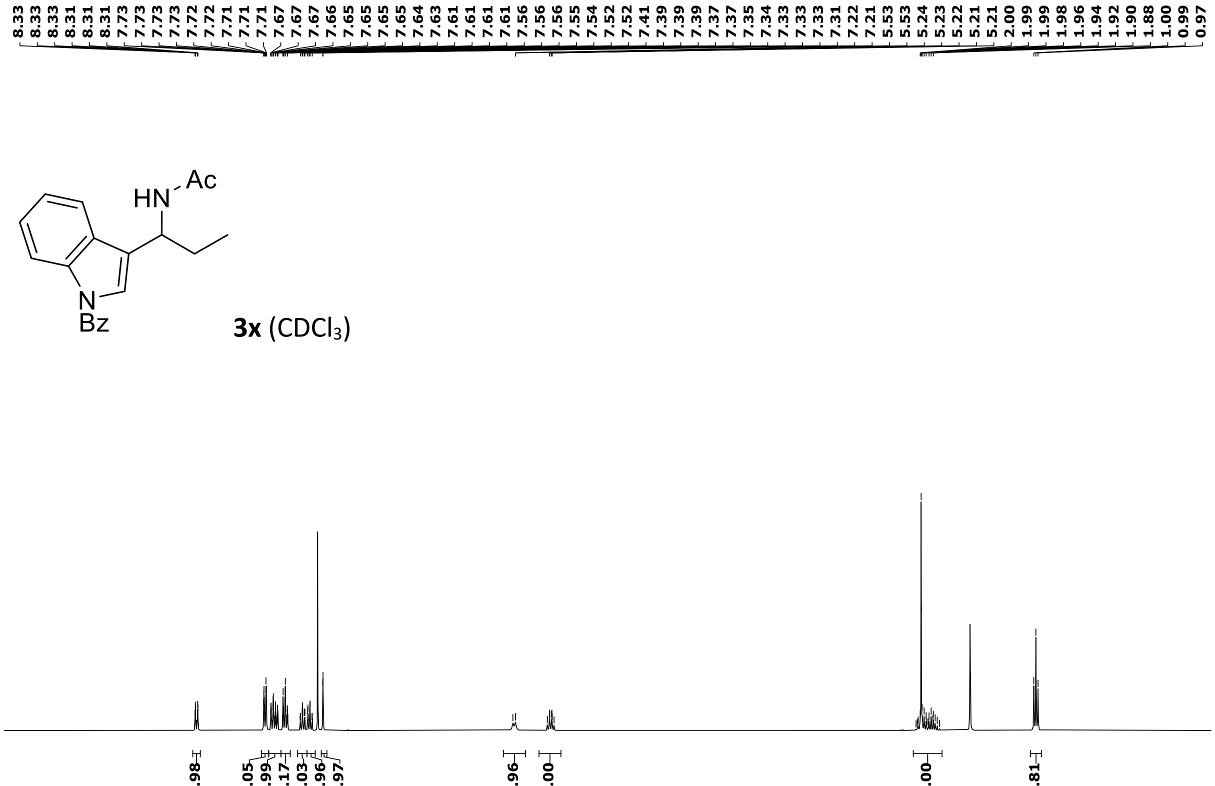

3x ($CDCl_3$)

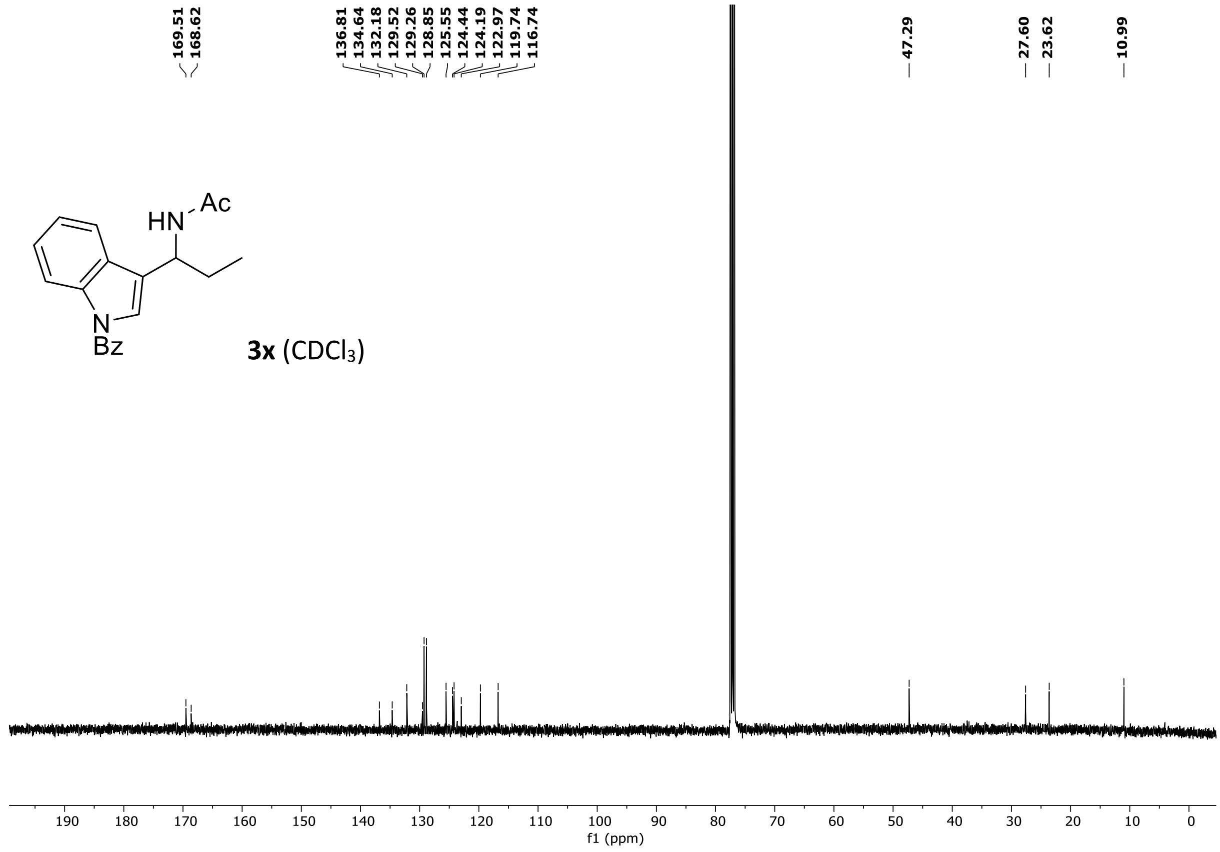

3x ($CDCl_3$)

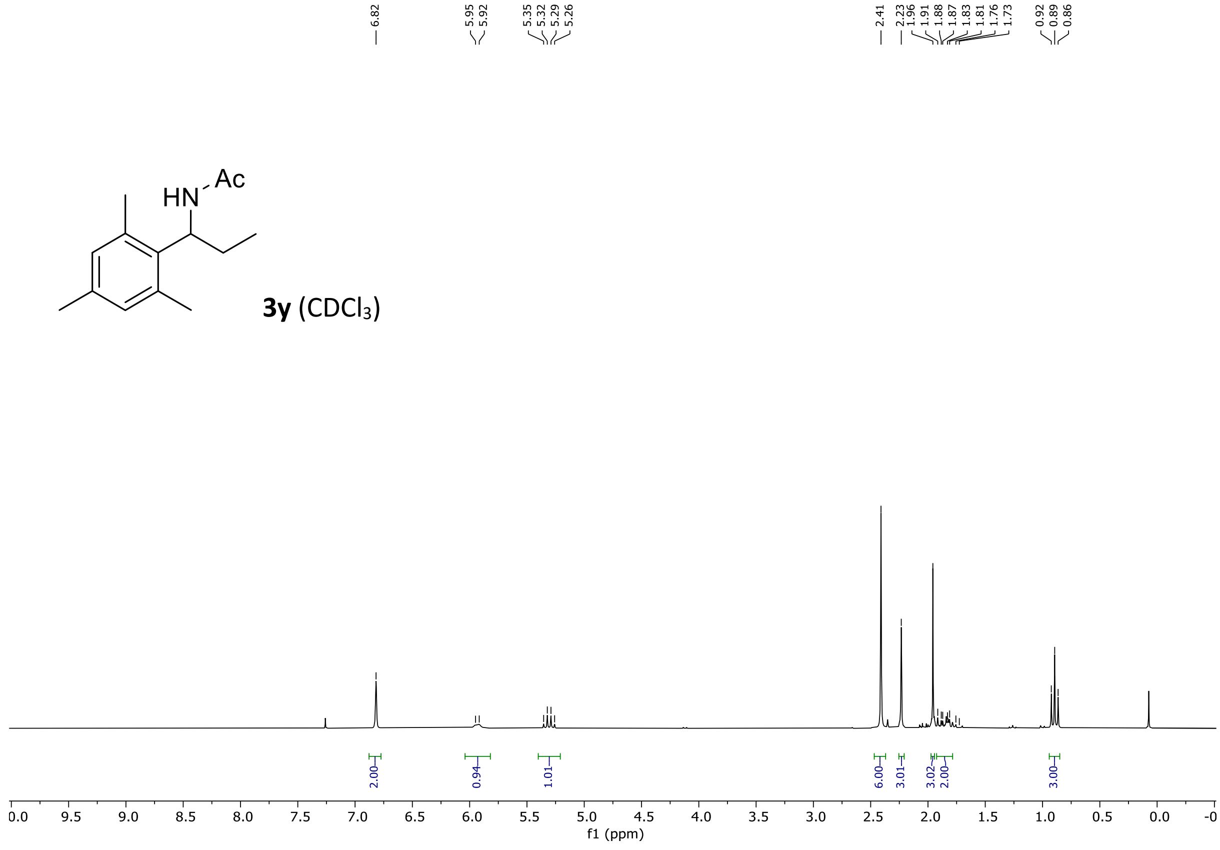

3y ($CDCl_3$)

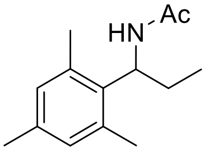

3y (CDCl$_3$)

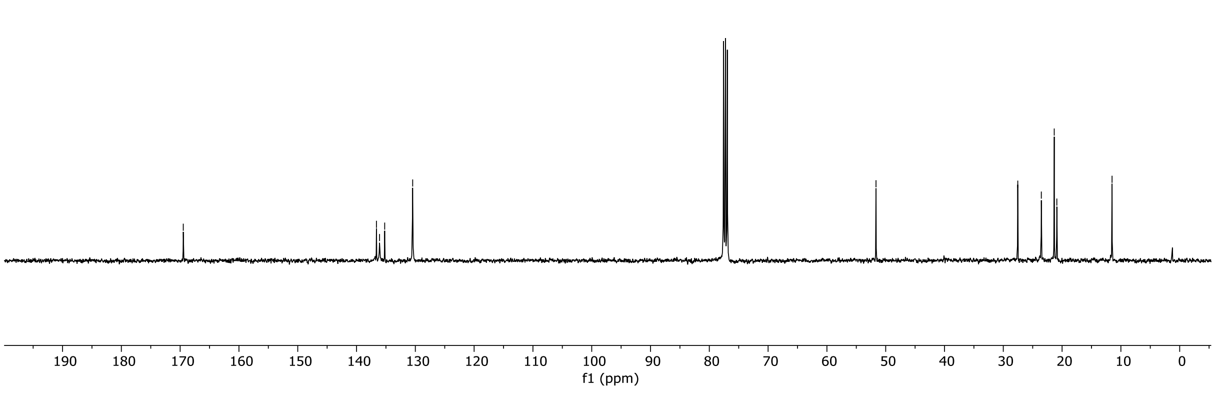

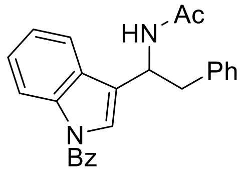

3z (CDCl$_3$)

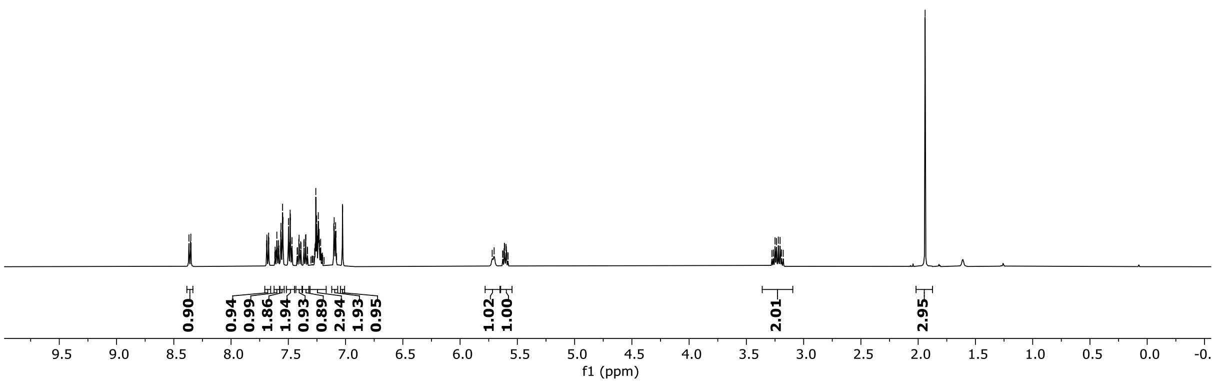

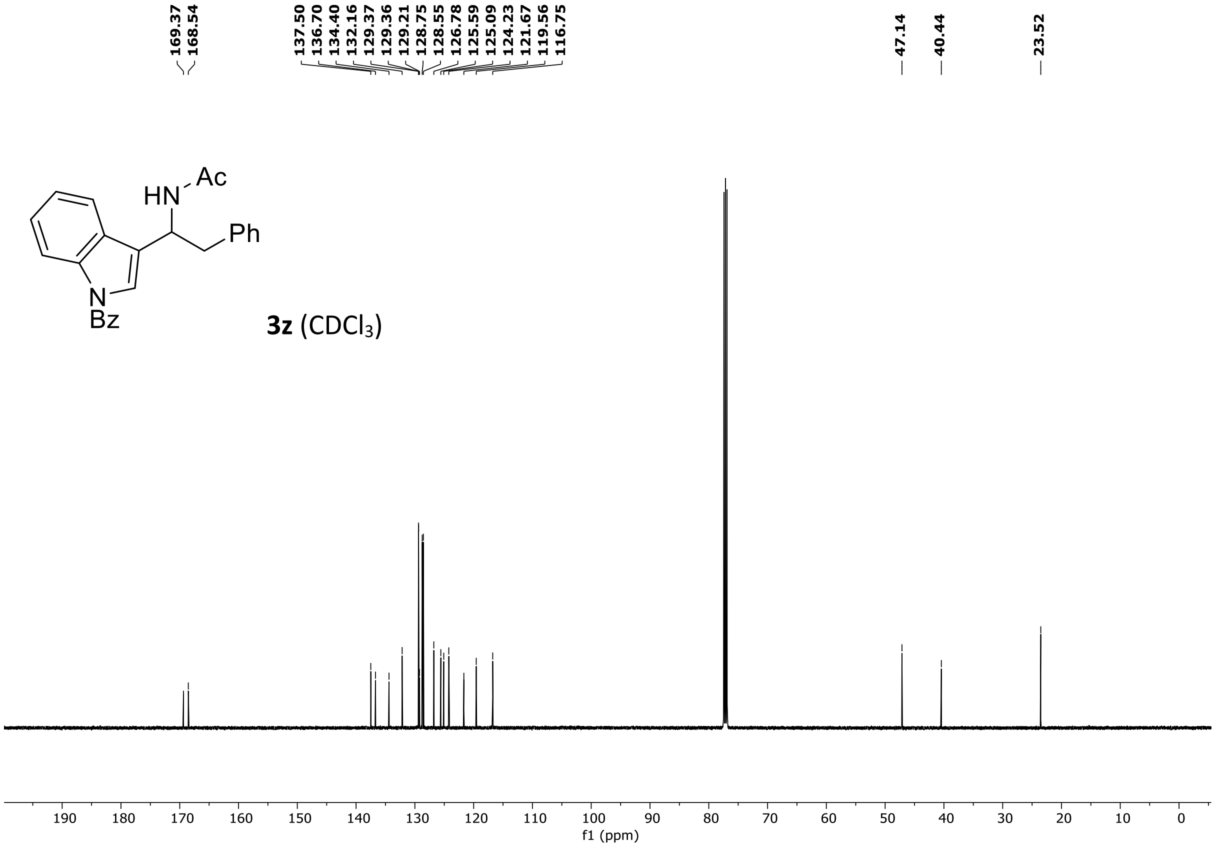

3z ($CDCl_3$)

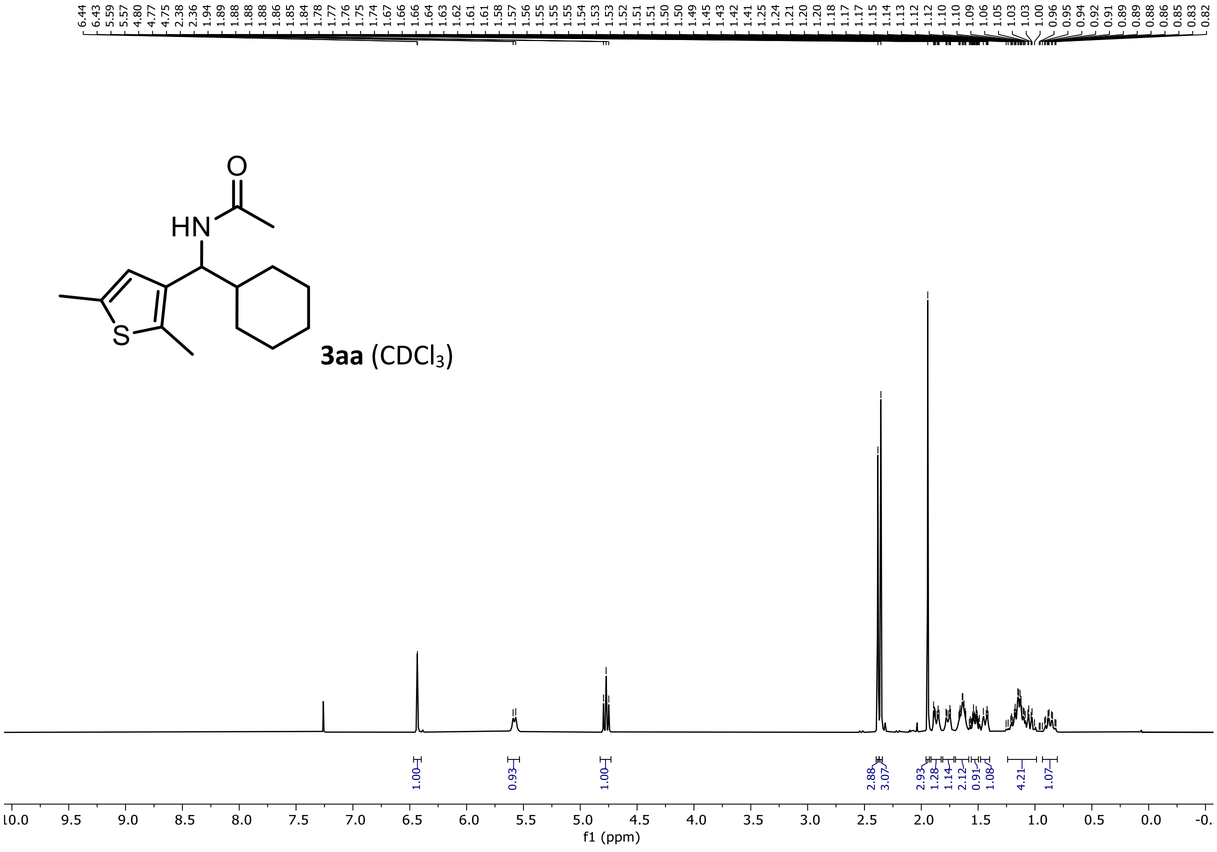

3aa ($CDCl_3$)

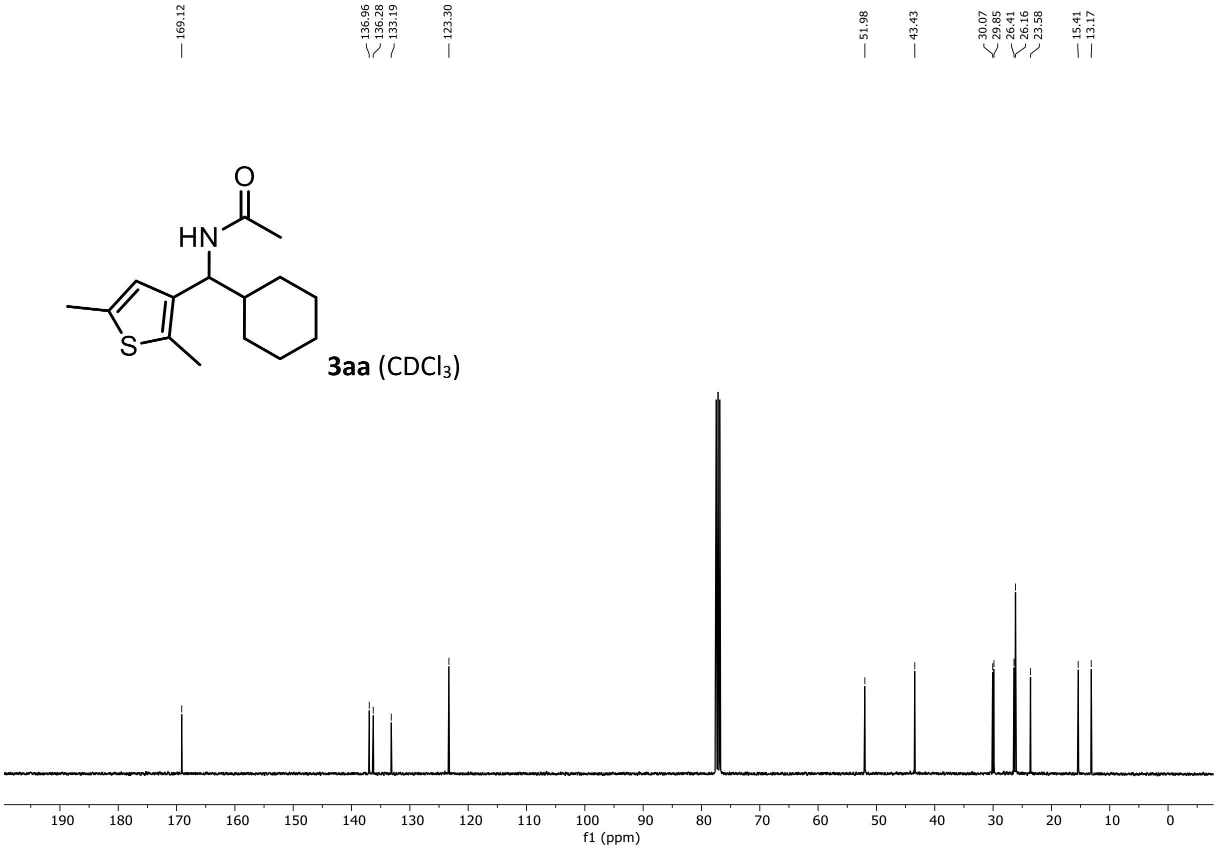

3aa ($CDCl_3$)

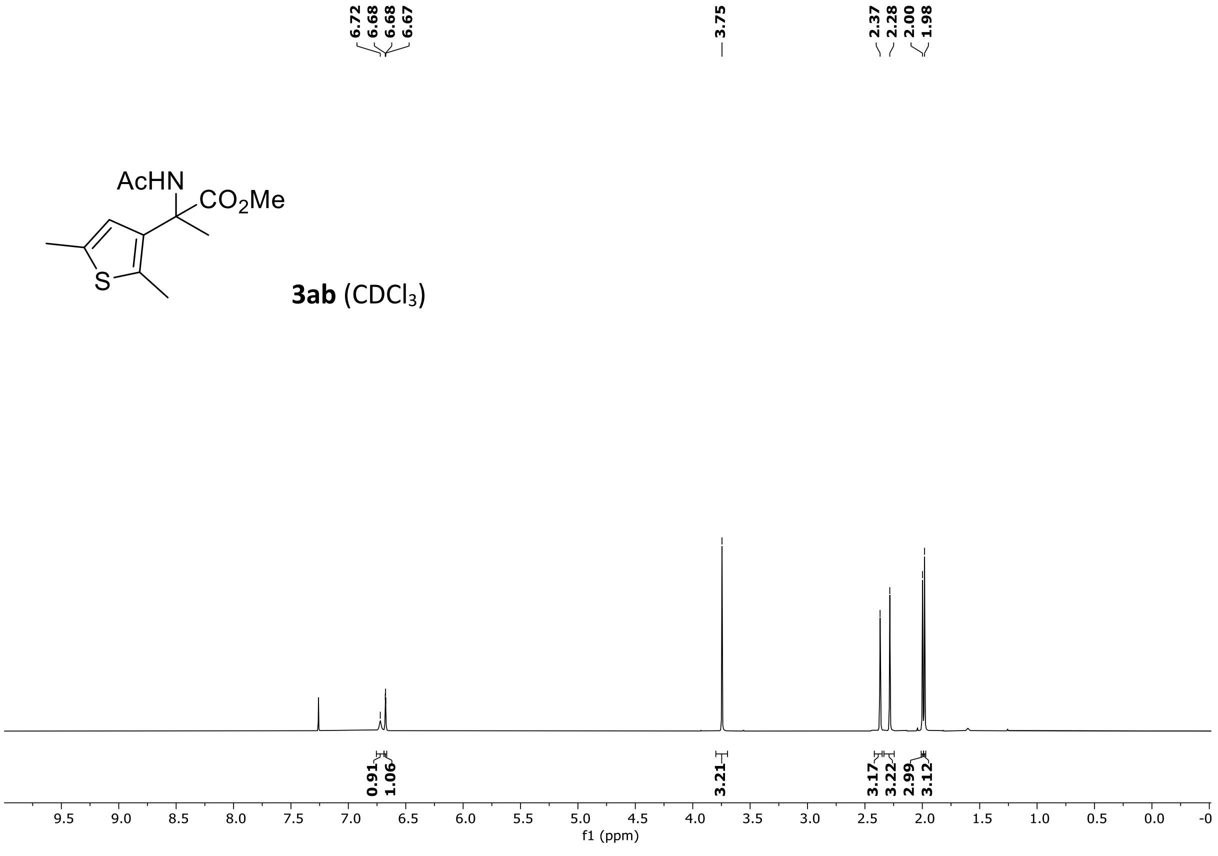

3ab ($CDCl_3$)

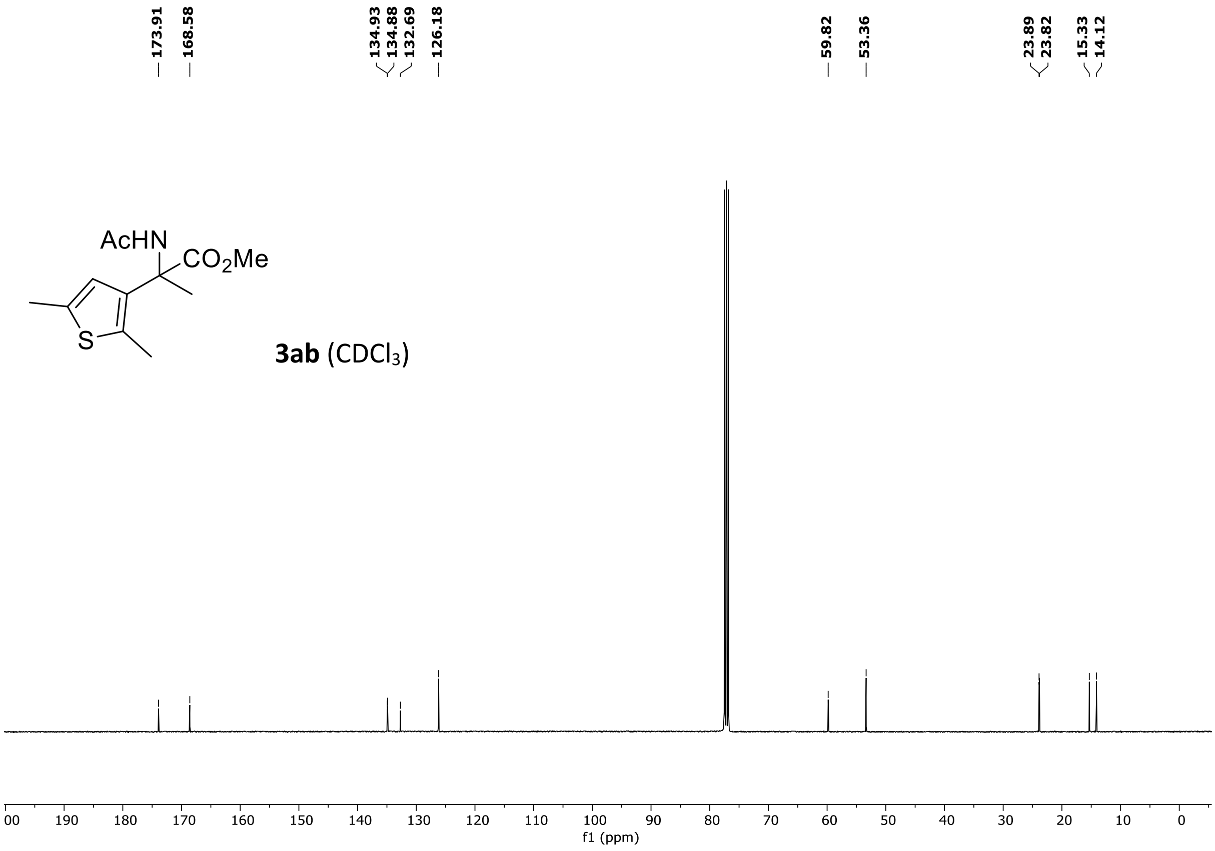

3ab (CDCl$_3$)

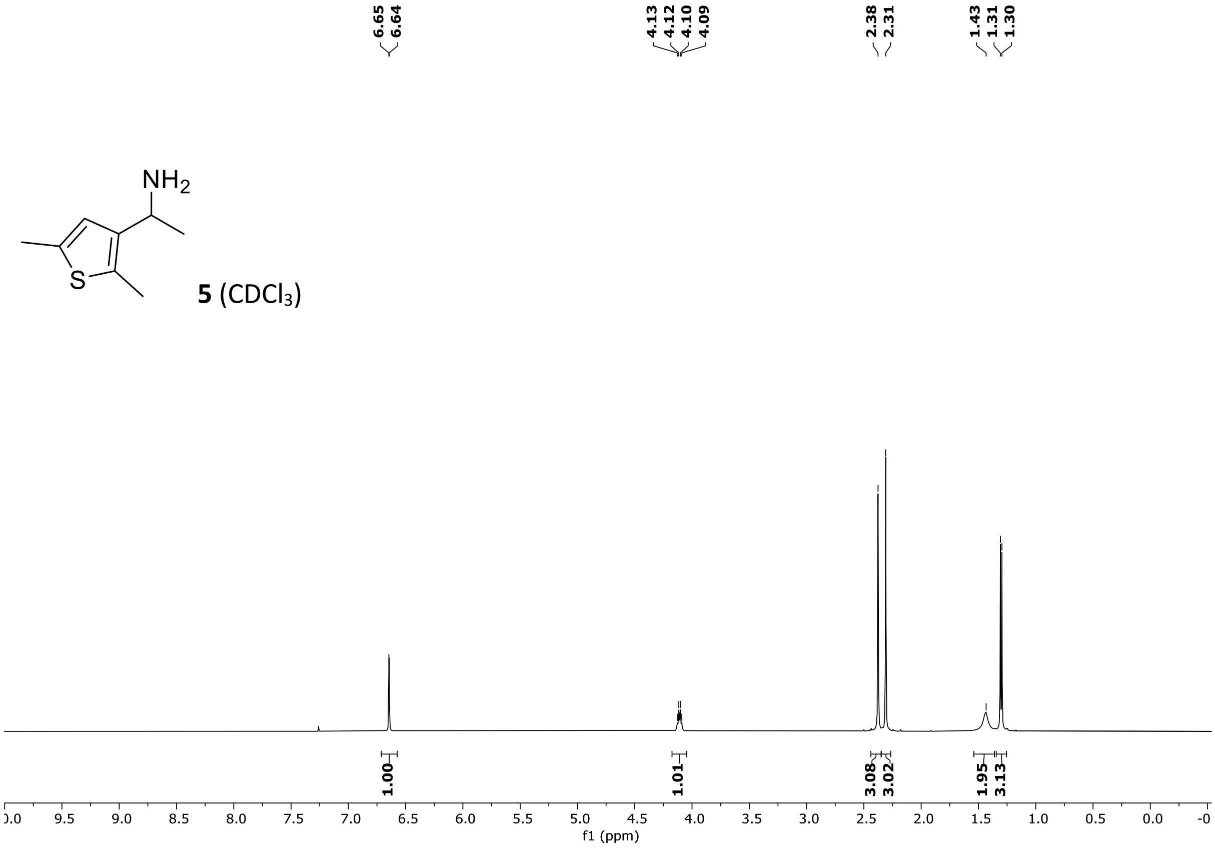

5 (CDCl$_3$)

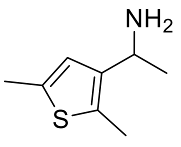

5 ($CDCl_3$)

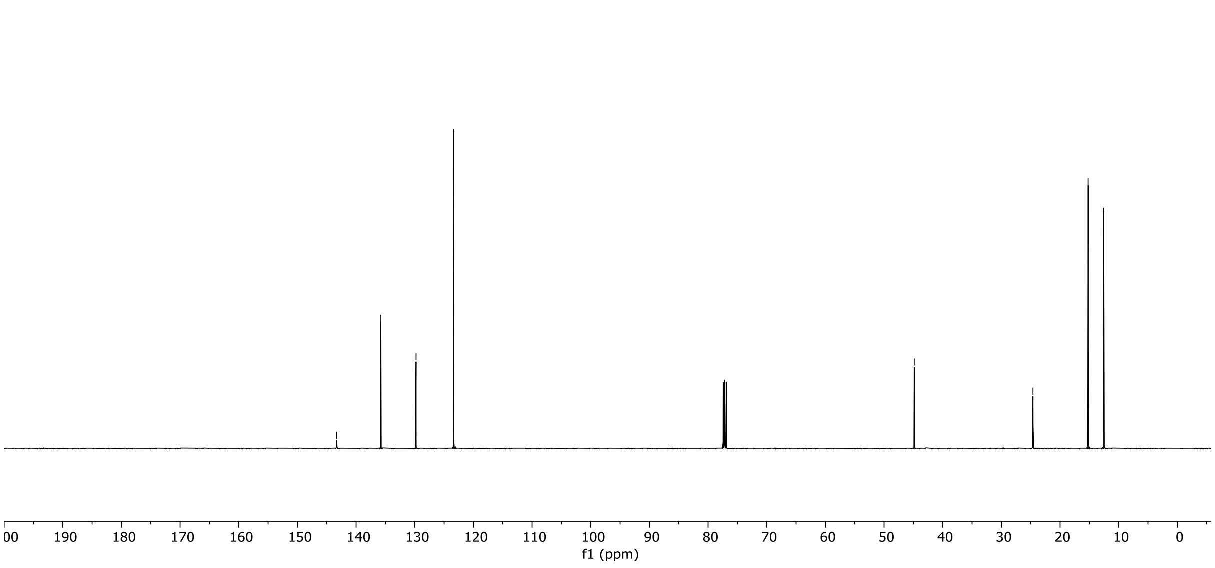

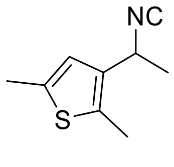

6 ($CDCl_3$)

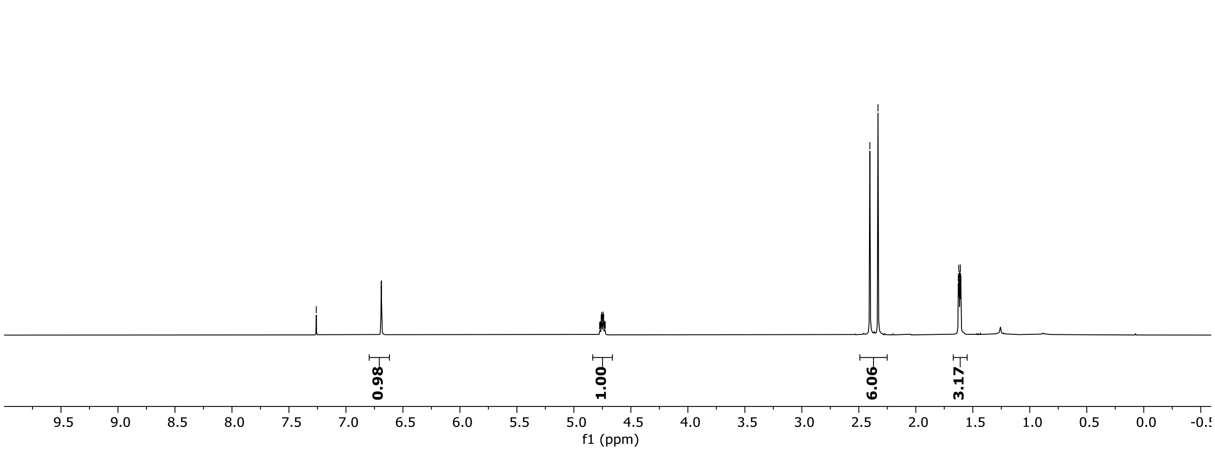

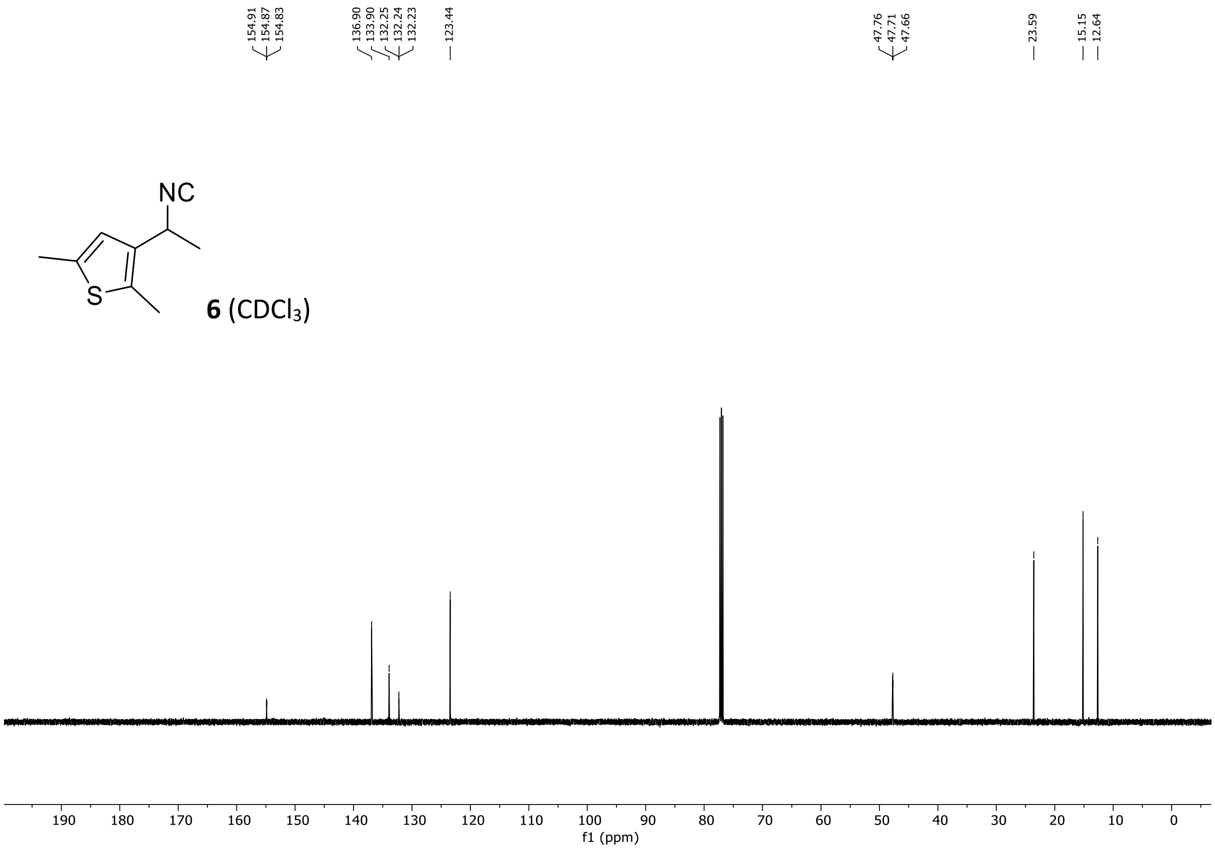

6 ($CDCl_3$)

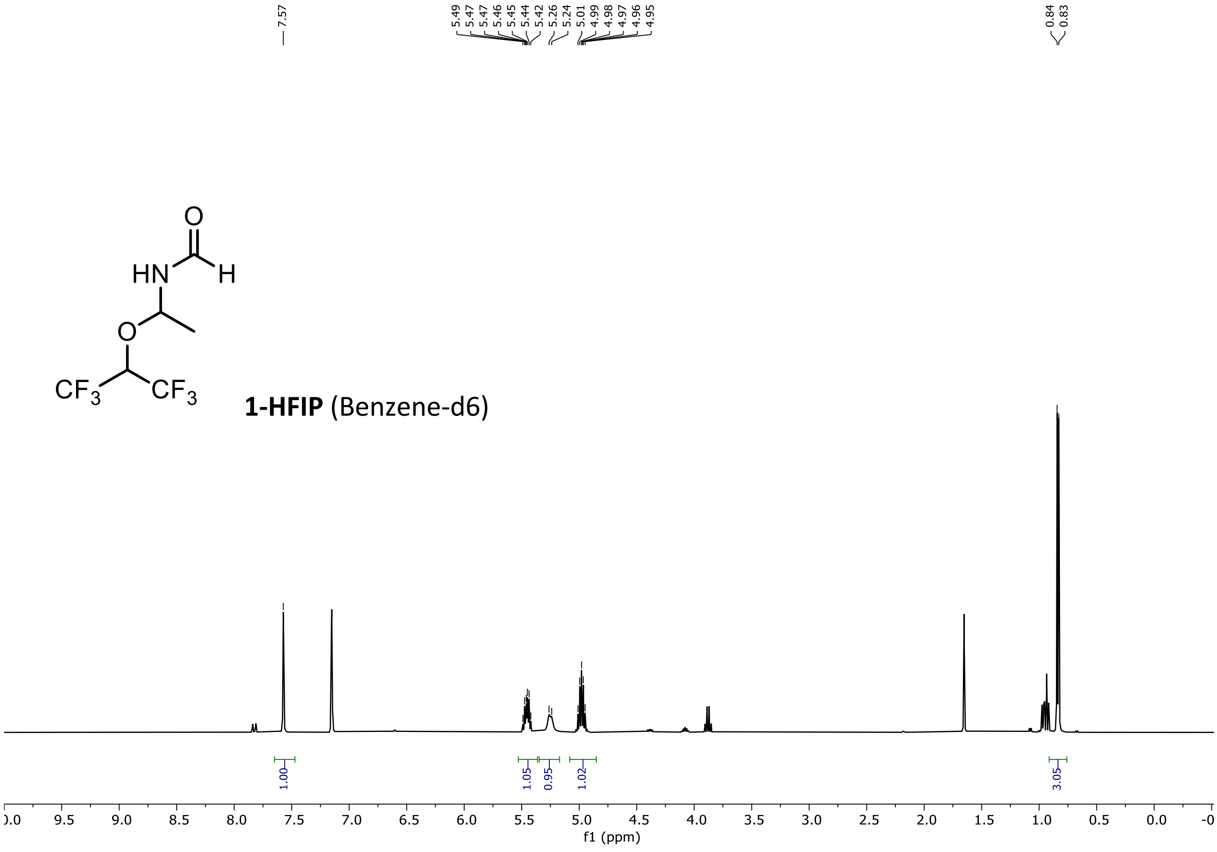

1-HFIP (Benzene-d6)

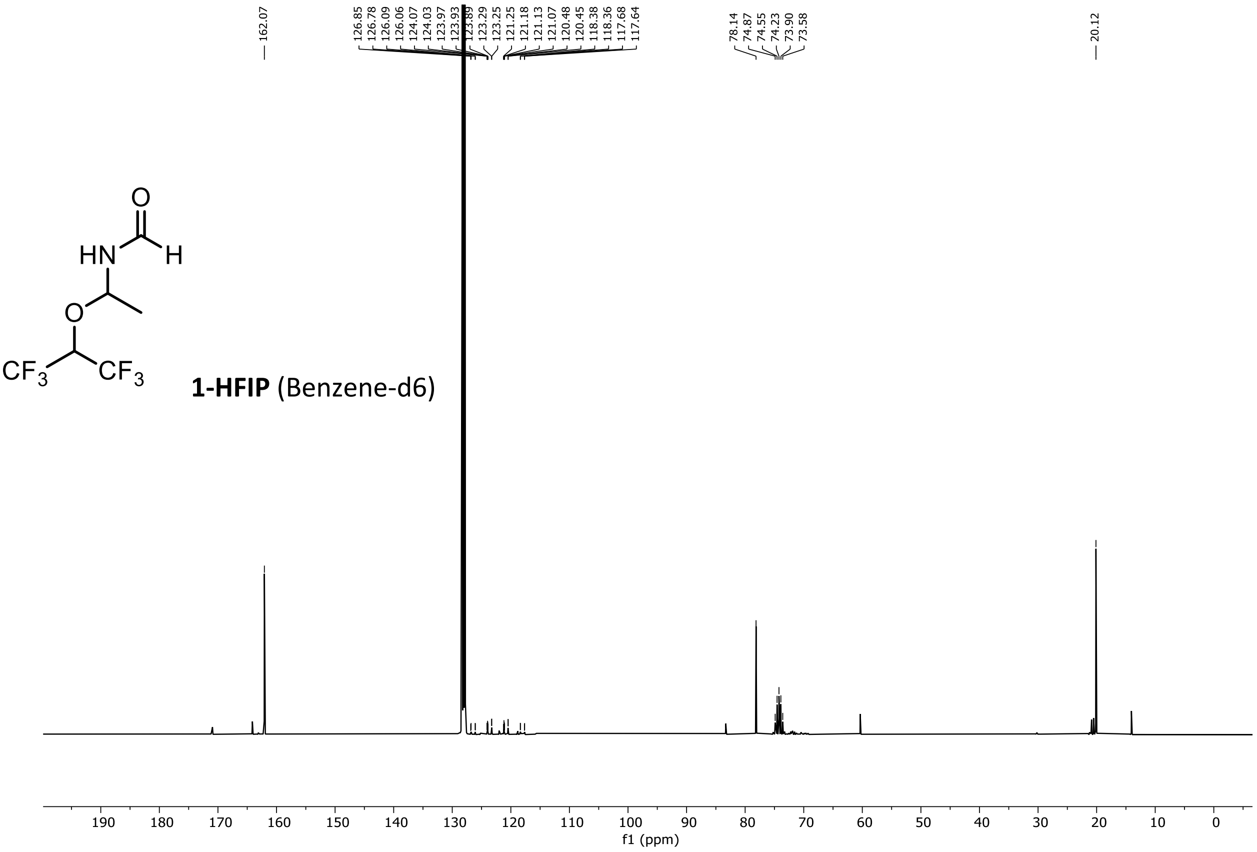
162.07
126.85
126.78
126.09
126.06
124.07
124.03
123.97
123.93
123.89
123.29
123.25
121.25
121.18
121.13
121.07
120.48
120.45
118.38
118.36
117.68
117.64
78.14
74.87
74.55
74.23
73.90
73.58
20.12
CF3
CF3
HN
H
O
O
1-HFIP (Benzene-d6)
f1 (ppm)

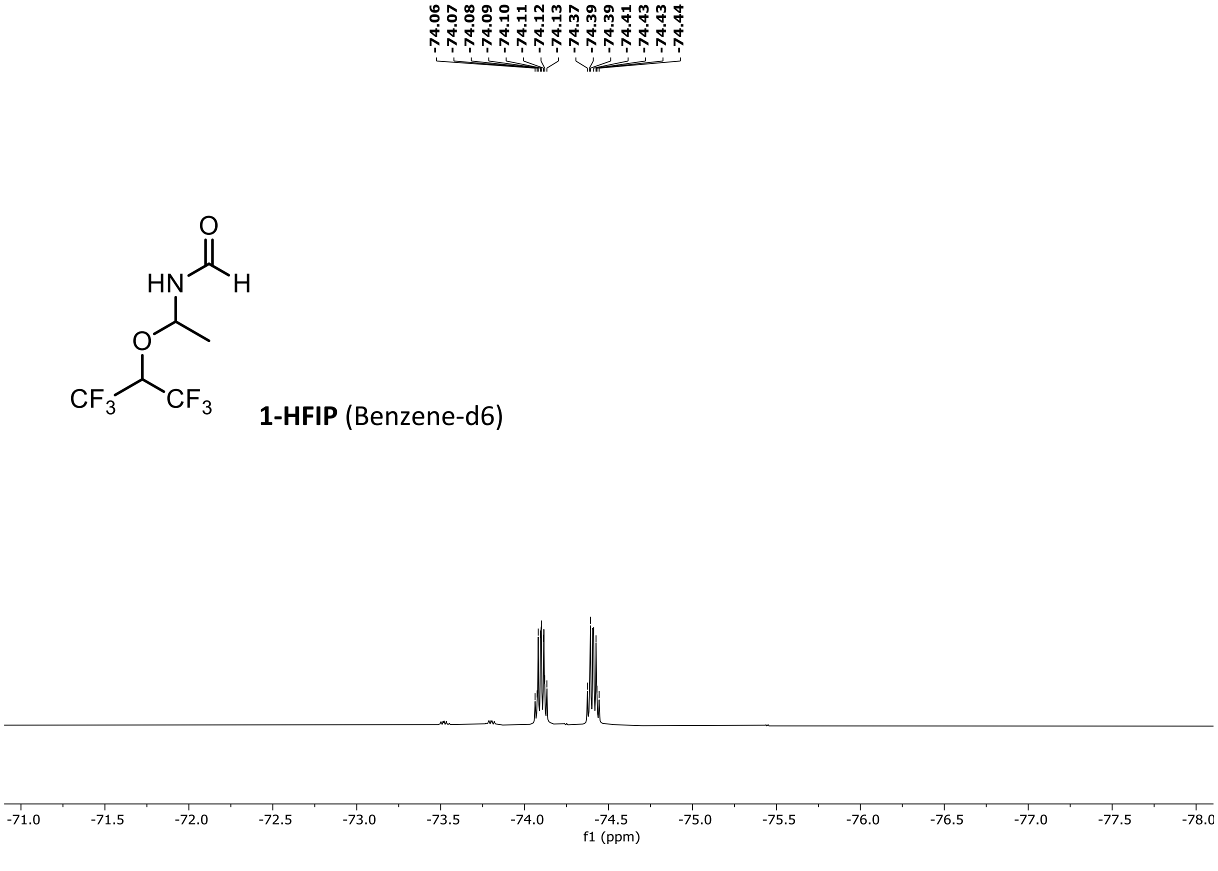
-74.06
-74.07
-74.08
-74.09
-74.10
-74.11
-74.12
-74.13
-74.37
-74.39
-74.39
-74.41
-74.43
-74.43
-74.44
CF3
CF3
HN
H
O
O
1-HFIP (Benzene-d6)
f1 (ppm)

IV. Mechanistic Studies

IV.1 Quantification of the Rotational Barrier of 1a (Figure 1A)

Determination of *cis/trans* isomerisation rates of *N*-vinylformamide (RM02-263)

A NMR tube was charged with *N*-vinylformamide (16.7 µL) in $CDCl_3$ (600 µL). NMR spectra were recorded with the *noesyphpp* pulse sequence with mixing times of 0 and 800 ms. Space integrals of the 2D spectra for both mixing times were determined with Mnova on the identical chemical shift ranges.

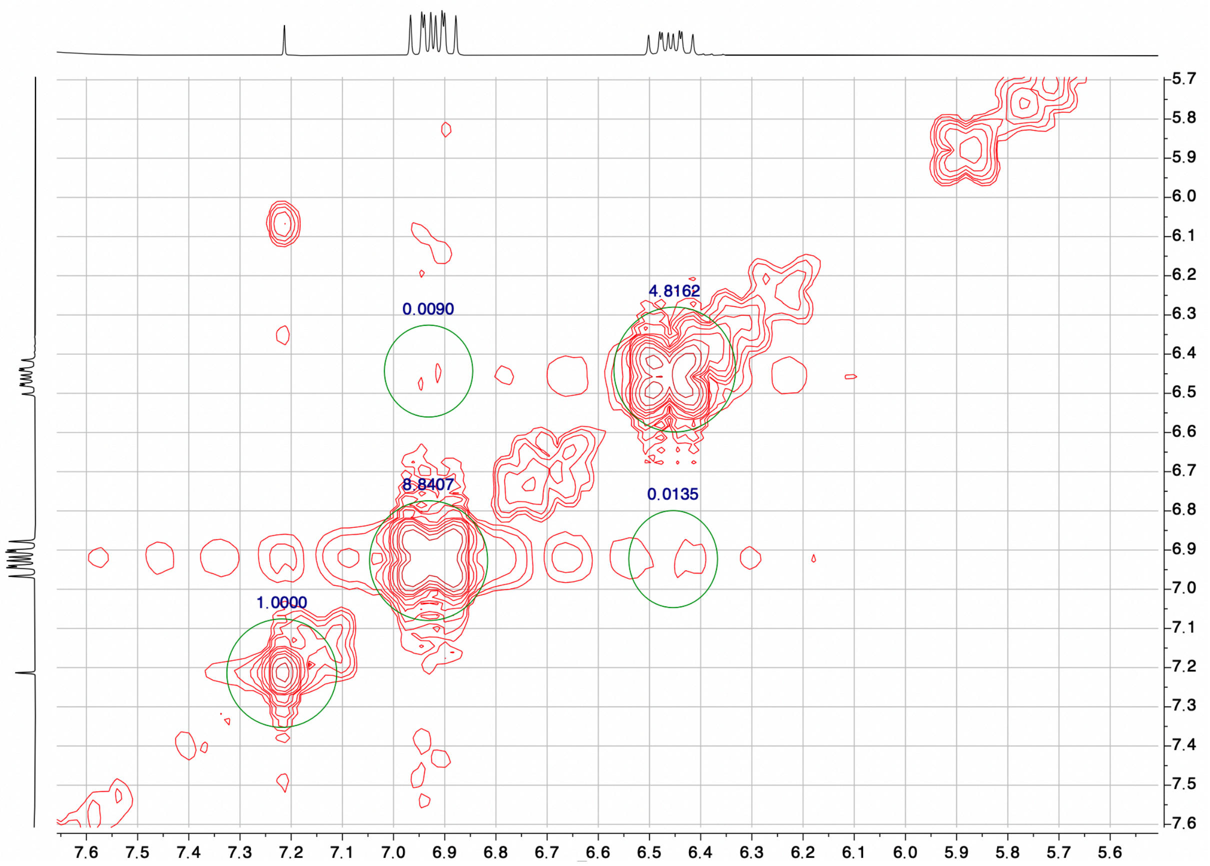

Figure S1. $^1H,^1H$ NOESY NMR (400 MHz, $CDCl_3$, mixing time: 0 ms) and volume integrals relative to the one of residual $CHCl_3$ (set as 1.000).

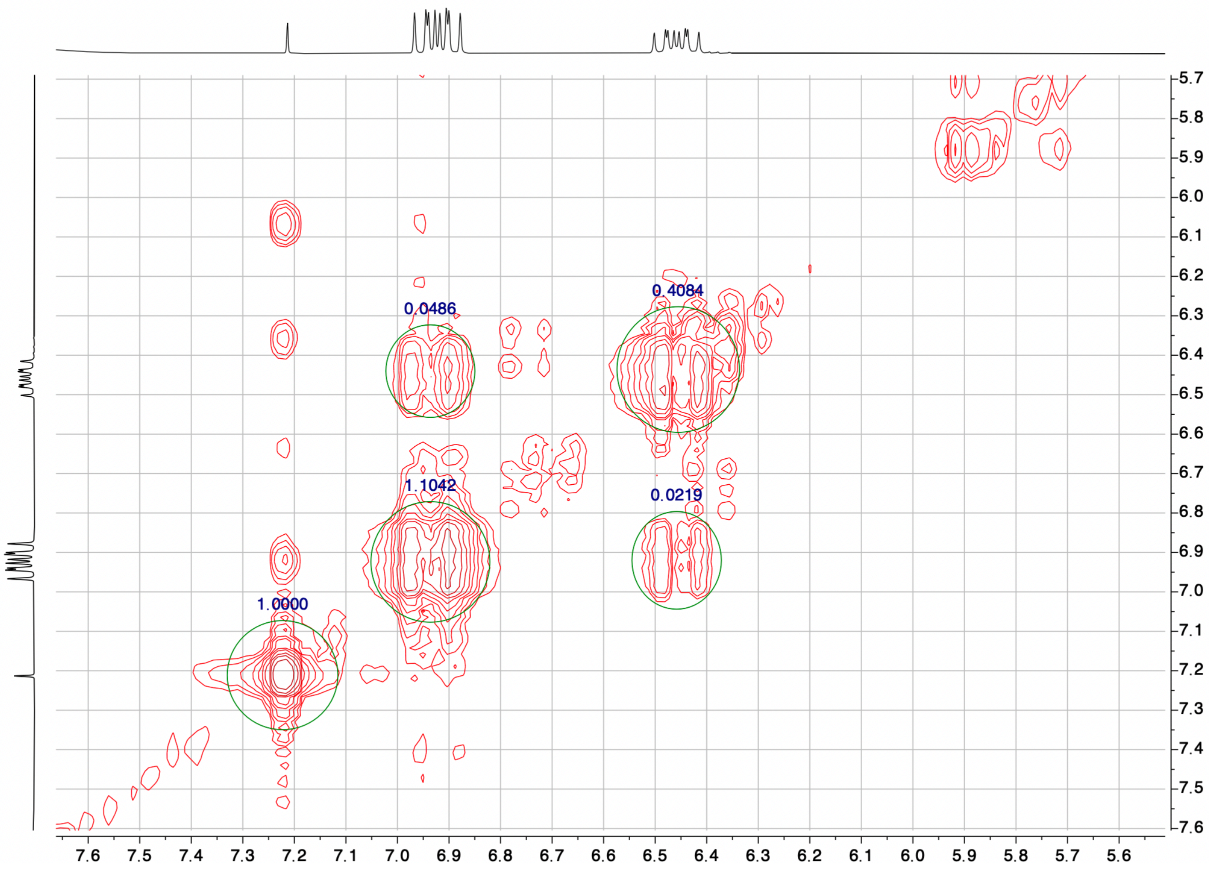

Figure S2. $^{1}H,^{1}H$ NOESY NMR (400 MHz, $CDCl_3$, mixing time: 800 ms) and volume integrals relative to the one of residual $CHCl_3$ (set as 1.000).

The space integrals were added into the EXSYCalc software which afforded k_1 = 0.122 s^{-1} and k_{-1} = 0.030 s^{-1} as rates for the exchange process.

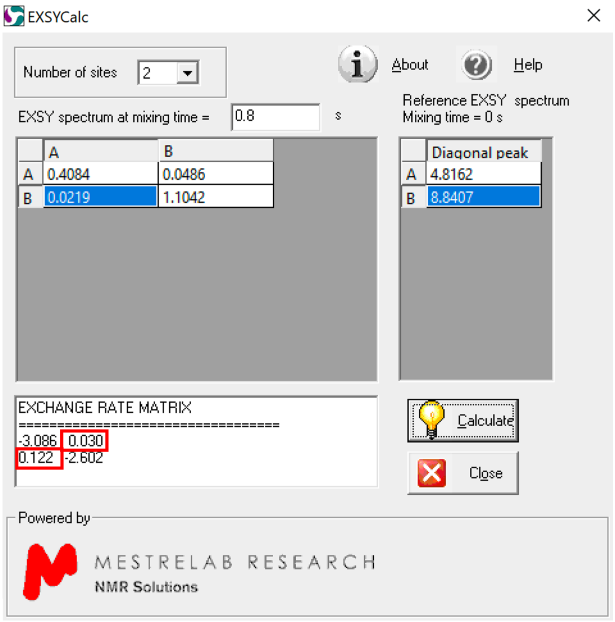

Figure S3. Output from the EXSYCalc software (values in red boxes) using the volume integrals from Figure S1 and S2.

IV.2 NMR Kinetics (Figure 1B)

^{1}H NMR kinetics for the reaction of *N*-vinylformamide (1a) with mesitylene (2a) - RM02-264-4

A NMR tube was charged with *N*-vinylformamide (16.7 µL, 0.24 mmol, 1 equiv; concentration in measurement: 0.400 M), mesitylene (134 µL, 0.96 mmol, 4 equiv; concentration in measurement: 1.60 M), C_6D_6 (50 µL), 1,1,2,2-tetrachloroethane (20 µL) and HFIP (378 µL). Triflic acid (2.12 µL, 0.024 mmol, 0.1 equiv; concentration in measurement: 0.0400 M) was added, the NMR tube was quickly sealed, mixed and a timer was started.

The NMR tube was added into the NMR spectrometer which was directly before shimmed on an NMR tube with similar composition. After insertion of the kinetics-sample, C_6D_6 was locked as solvent, the measurement of the kinetics was immediately started and the time for the acquisition of the first spectrum noted (104 s). Spectra with ns = 8 were continuously acquired for 6 h and the concentrations determined relative to those of tetrachloroethane.

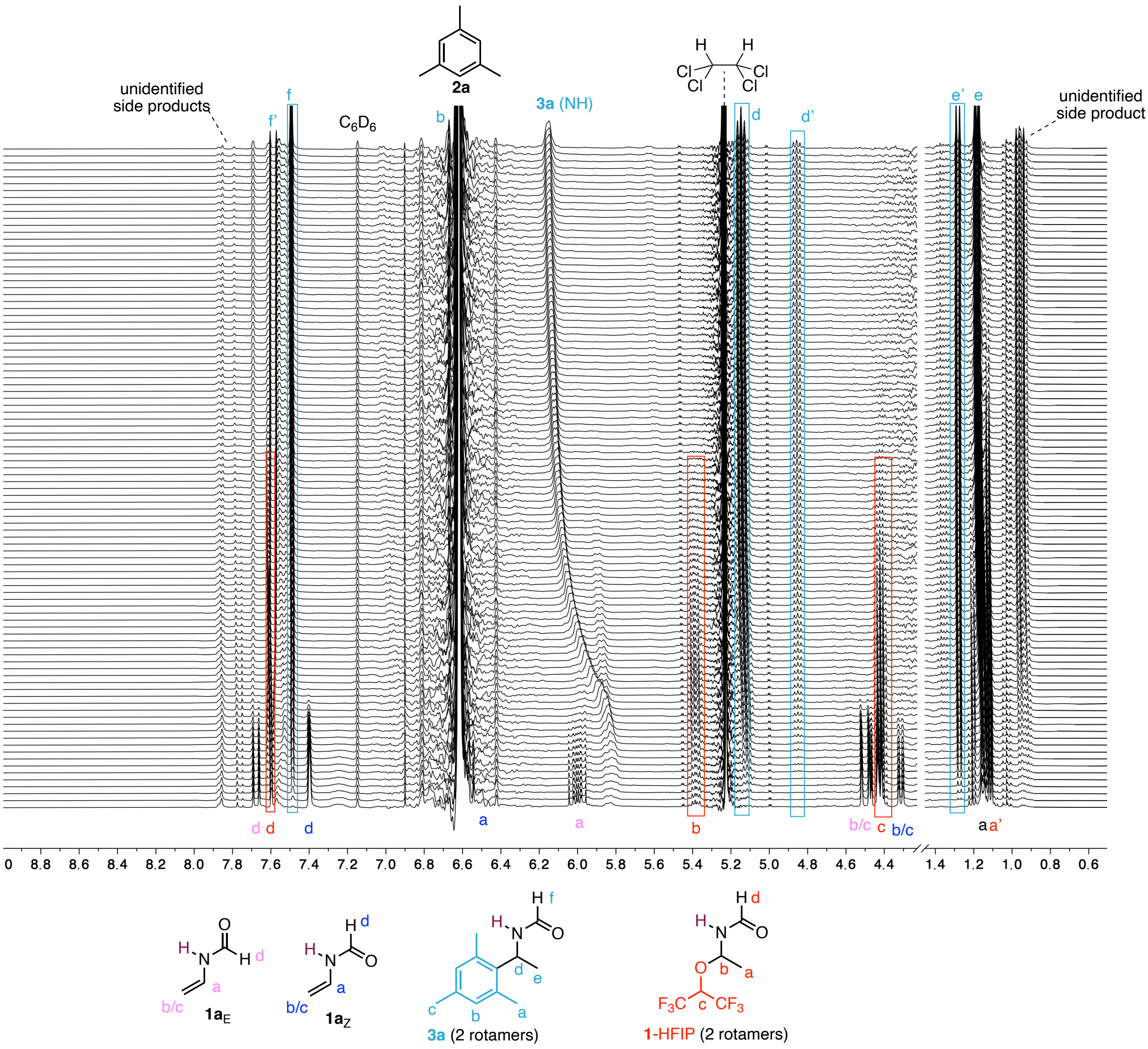

Figure S4. ^{1}H NMR kinetics (400 MHz) of the reaction of **1a** with **2a** in the presence of HOTf in HFIP containing C_6D_6 and tetrachloroethane as internal standards (the region between 1.5 and 4.3 ppm was removed for clarity).

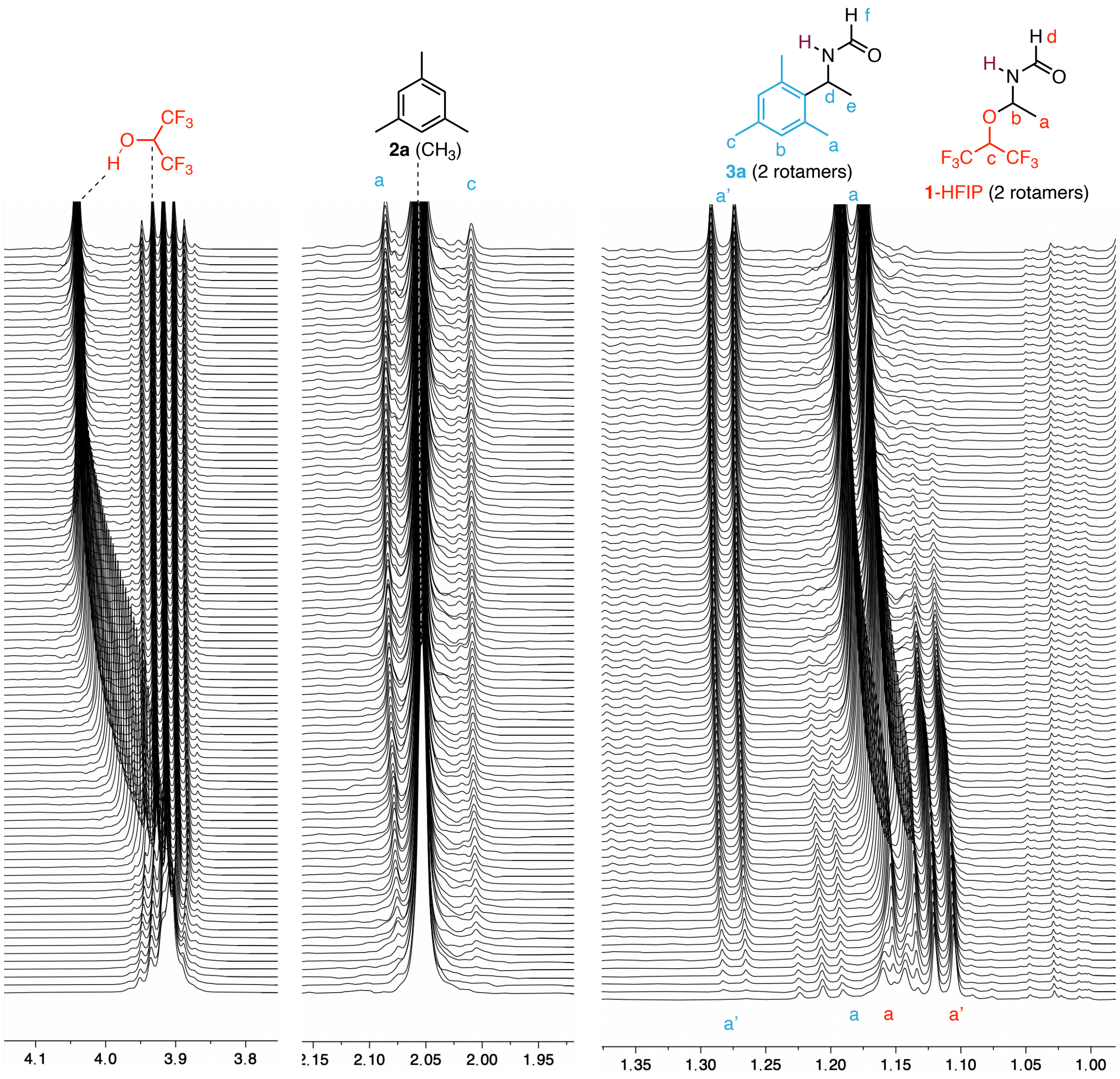

Figure S5. Specific regions in the ^{1}H NMR kinetics (400 MHz) of the reaction of **1a** with **2a** in the presence of HOTf in HFIP containing C_6D_6 and tetrachloroethane as internal standards.

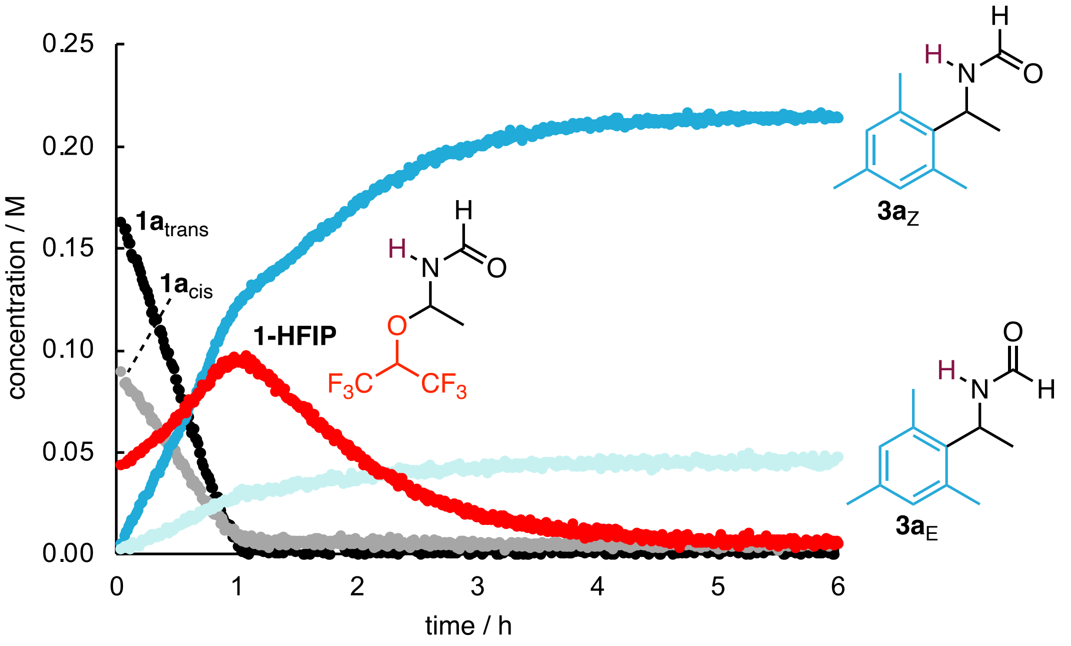

Figure S6. Concentration-time profiled from ^{1}H NMR kinetics (400 MHz) of the reaction of **1a** with **2a** in the presence of HOTf in HFIP containing C_6D_6 and tetrachloroethane as internal standards. Note: the measured concentrations differ from the weighed amounts as the spectrum was recorded with a relatively short relaxation time (0.1 s) and are therefore not quantitative.

IV.3 IR Kinetics (Figure 2)

IR kinetics were followed using a Mettler Toledo ReactIR 15 and a 6.3 mm AgX Fibre probe with 64 scans per spectrum. Data was acquired and processed with the Mettler-Toledo iC IR software with solvent subtraction of HFIP and a single-point baseline at 1754 cm^{-1}. First, the extinction coefficient of **1a** was determined under these conditions to allow quantification of the absolute concentration of **1a** in kinetic measurements.

Extinction coefficient of 1a

A flask was charged with 2000 µL HFIP and the solvent background was determined. **1a** was added stepwise and the absorbance increase at 1652 cm^{-1}, which corresponds to the C=O band of **1a**$_{trans}$, was recorded. As the ratio of **1a**$_{trans}$: **1a**$_{cis}$ stays constant during the reaction, monitoring of the band associated with **1a**$_{trans}$ can be used to determine the total concentration of **1a**.

Table S1. Concentration dependent absorbance of **1a**.

Step	V **1a** / µL	V_{tot} / µL	[**1a**]$_{total}$ / M	A at 1652 cm^{-1}
0	0	2000	0.00E+00	0.000
1	10	2010	7.14E-02	0.040
2	20	2020	1.42E-01	0.075
3	30	2030	2.12E-01	0.111
4	50	2050	3.50E-01	0.178
5	70	2070	4.85E-01	0.240
6	110	2110	7.48E-01	0.360

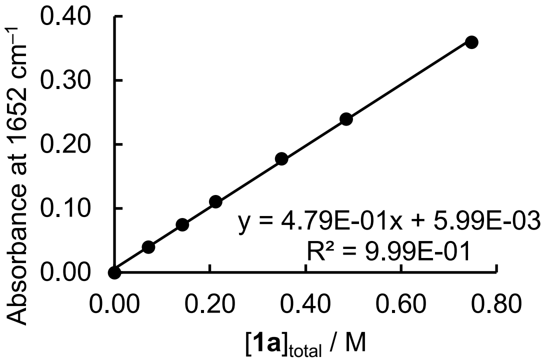

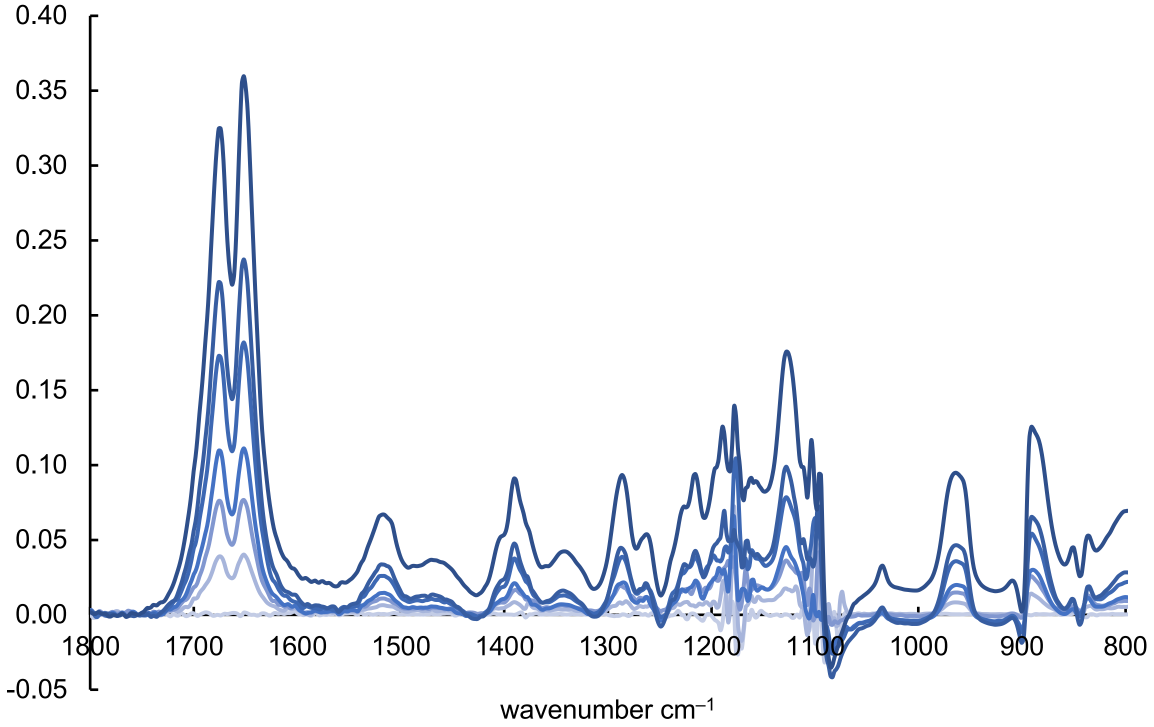

Figure S7. IR spectra of **1a** in HFIP at different concentrations after solvent subtraction.

IR Kinetics

A two-neck round-bottom flask connected to the probe of the ReactIR was successively charged with HFIP, *N*-vinyl formamide (**1a**), mesitylene (**2a**), and TfOH with all volumes calculated to reach a total volume of 2000 µL as indicated in Table S2. The decay of the absorbance at 1652 cm^{-1} was extracted and the concentration of **1a** determined from the absorbance by means of the calibration curve in Figure SX. Due to overlap of the absorbance of **1a** with the intermediate **1a**-H^+ and the reaction product

3a, not the full decay of the concentration of **1a** can be followed and only the initial linear part was evaluated by means of a linear fit.

Table S2. Concentrations and volumes of reagents used for IR kinetics.

Sample	[**1a**] / M	[TfOH] / M	[**2a**] / M	$V_{\mathbf{1a}}$ / µL	V_{TfOH} / µL	$V_{\mathbf{2a}}$ / µL	$V_{\mathbf{HFIP}}$ / µL	k_{init} / M s^{-1}
1	0.4	0.0400	0.4	55.7	7.08	111.3	1825.9	2.59 x 10^{-5}
2a	0.4	0.0400	0.8	55.7	7.08	222.6	1714.6	4.42 x 10^{-5}
2b	0.4	0.0400	0.8	55.7	7.08	222.6	1714.6	4.74 x 10^{-5}
3	0.4	0.0400	1.2	55.7	7.08	333.9	1603.3	5.50 x 10^{-5}
4	0.4	0.0400	1.6	55.7	7.08	445.1	1492.0	6.89 x 10^{-5}
5	0.4	0.0200	0.8	55.7	3.54	222.6	1718.1	2.38 x 10^{-5}
6	0.4	0.0600	0.8	55.7	10.62	222.6	1711.1	5.55 x 10^{-5}
7	0.4	0.0800	0.8	55.7	14.16	222.6	1707.5	7.02 x 10^{-5}
8	0.2	0.0400	0.8	27.9	7.08	222.6	1742.5	5.07 x 10^{-5}
9	0.6	0.0400	0.8	83.6	7.08	222.6	1686.7	3.90 x 10^{-5}
10	0.8	0.0400	0.8	111.5	7.08	222.6	1658.8	3.19 x 10^{-5}

Table S3. Kinetic curves (grey), data points used for the linear correlation (black) and linear regression (red).

RM02-265-1<br>[**1a**] = 0.4 M; [**2a**] = 0.4 M; [**TfOH**] = 0.04 M	RM02-265-2a<br>[**1a**] = 0.4 M; [**2a**] = 0.8 M; [**TfOH**] = 0.04 M
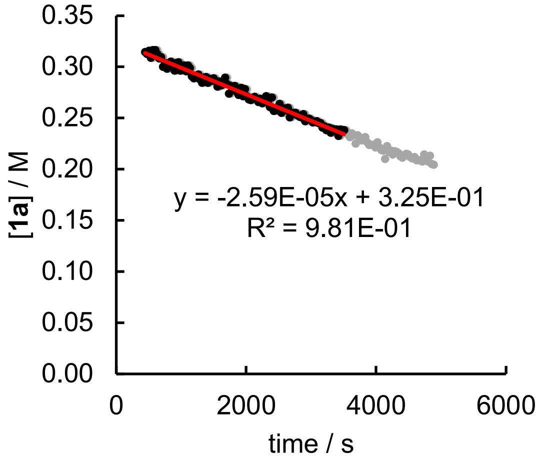	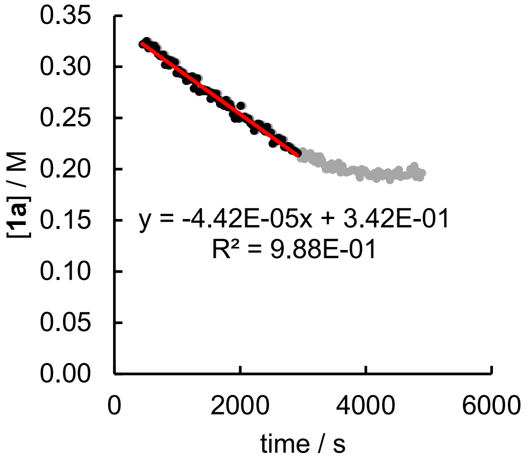

RM02-265-2b

[**1a**] = 0.4 M; [**2a**] = 0.8 M; [**TfOH**] = 0.04 M

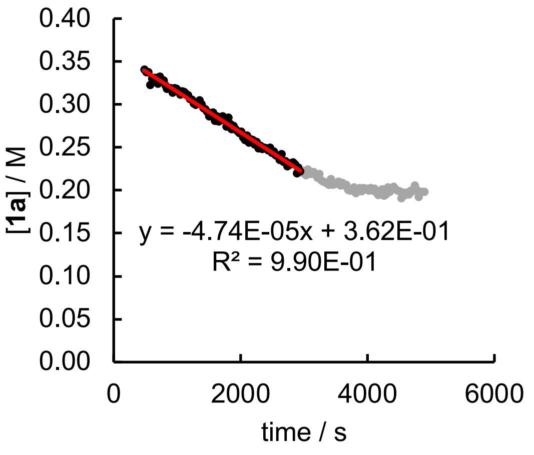

RM02-265-3

[**1a**] = 0.4 M; [**2a**] = 1.2 M; [**TfOH**] = 0.04 M

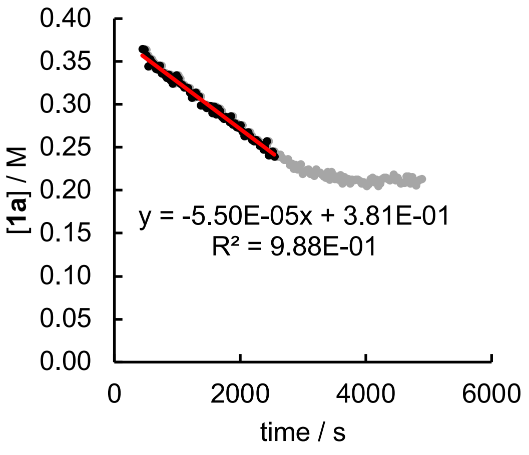

RM02-265-4

[**1a**] = 0.4 M; [**2a**] = 1.6 M; [**TfOH**] = 0.04 M

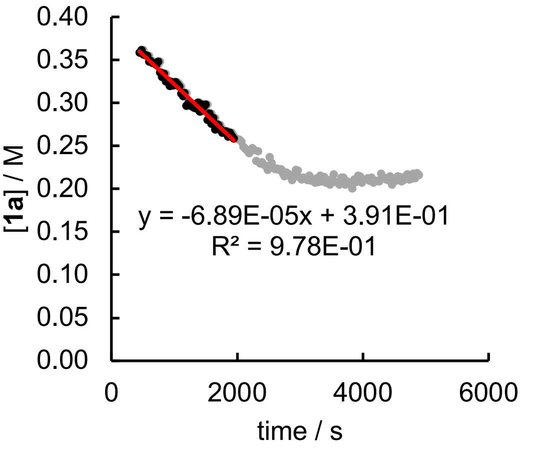

RM02-265-5

[**1a**] = 0.4 M; [**2a**] = 0.8 M; [**TfOH**] = 0.02 M

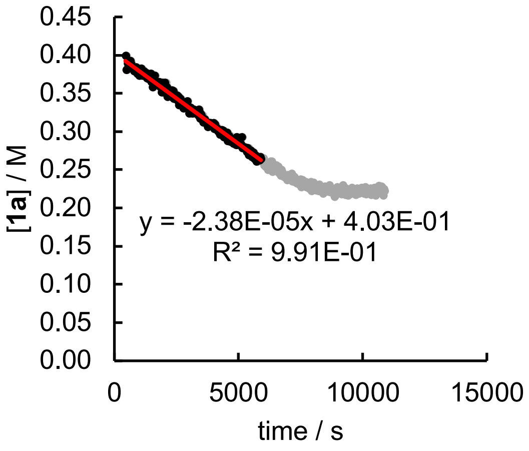

RM02-265-6

[**1a**] = 0.4 M; [**2a**] = 0.8 M; [**TfOH**] = 0.06 M

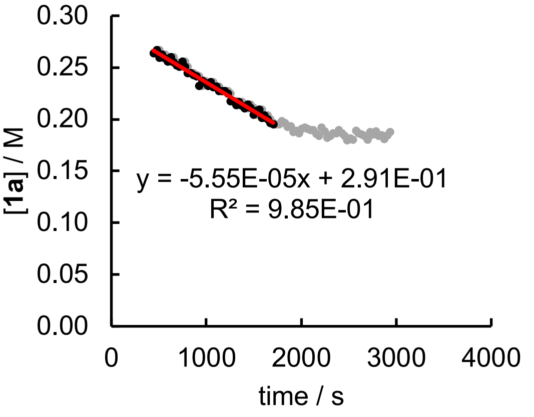

RM02-265-7

[**1a**] = 0.4 M; [**2a**] = 0.8 M; [**TfOH**] = 0.08 M

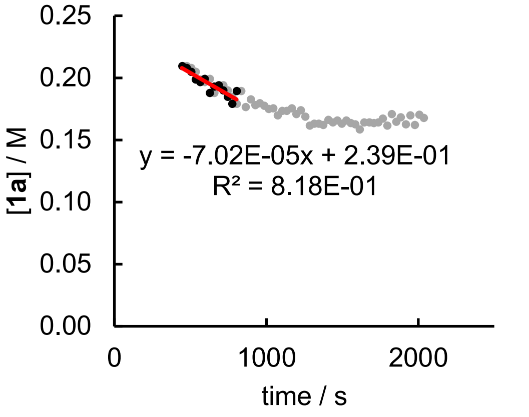

RM02-265-8
[**1a**] = 0.2 M; [**2a**] = 0.8 M; [**TfOH**] = 0.04 M

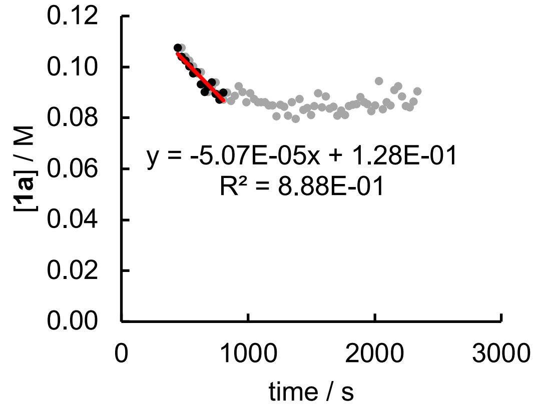

RM02-265-9
[**1a**] = 0.6 M; [**2a**] = 0.8 M; [**TfOH**] = 0.04 M

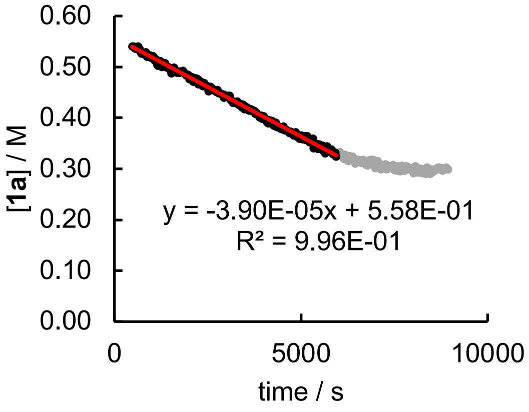

RM02-265-10
[**1a**] = 0.8 M; [**2a**] = 0.8 M; [**TfOH**] = 0.04 M

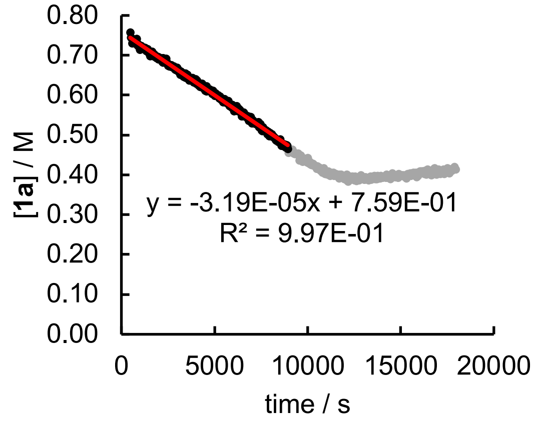

RM02-265-2-**deuterated**
[**1a**] = 0.4 M; [**2a**] = 0.8 M; [**TfOH**] = 0.04 M

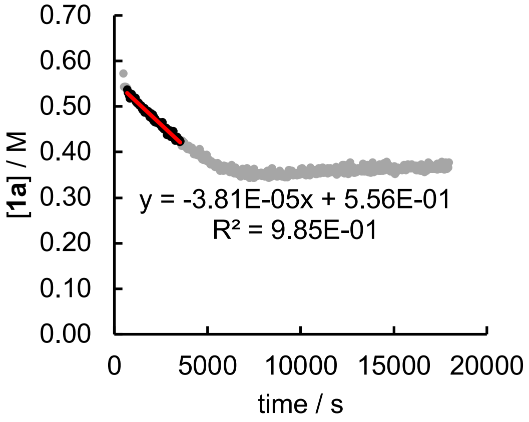

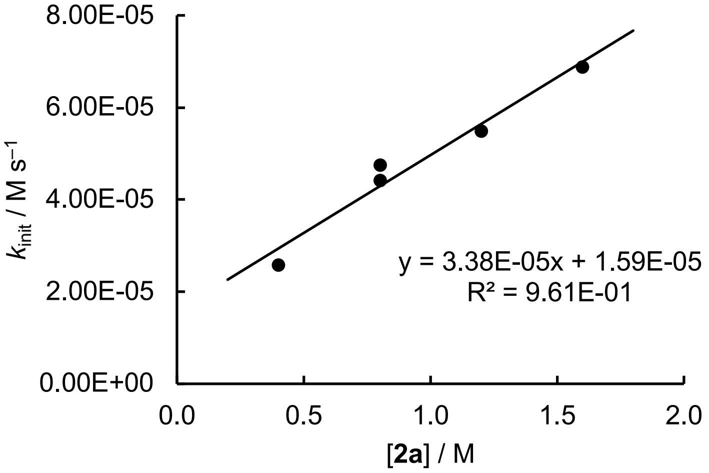

Figure S8. Correlation of k_{init} vs. [**2a**].

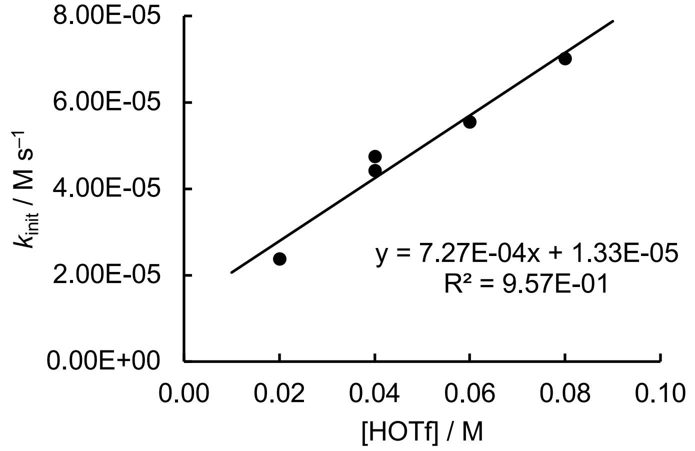

Figure S9. Correlation of k_{init} vs. [HOTf].

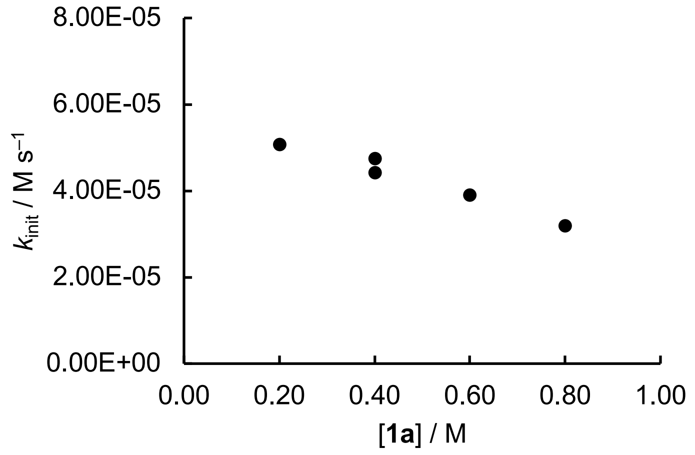

Figure S10. Correlation of k_{init} vs. [**1a**].

Product analysis of the reaction of 1a with 2a in OD-HFIP - RM02-265-2-deuterium (Figure 3)

After reaching full conversion according to IR monitoring, the reaction mixture was worked up as usual (General Procedure II.1) and the crude product characterized by ^{1}H and ^{2}H NMR as well as subjected to GC/MS analysis.

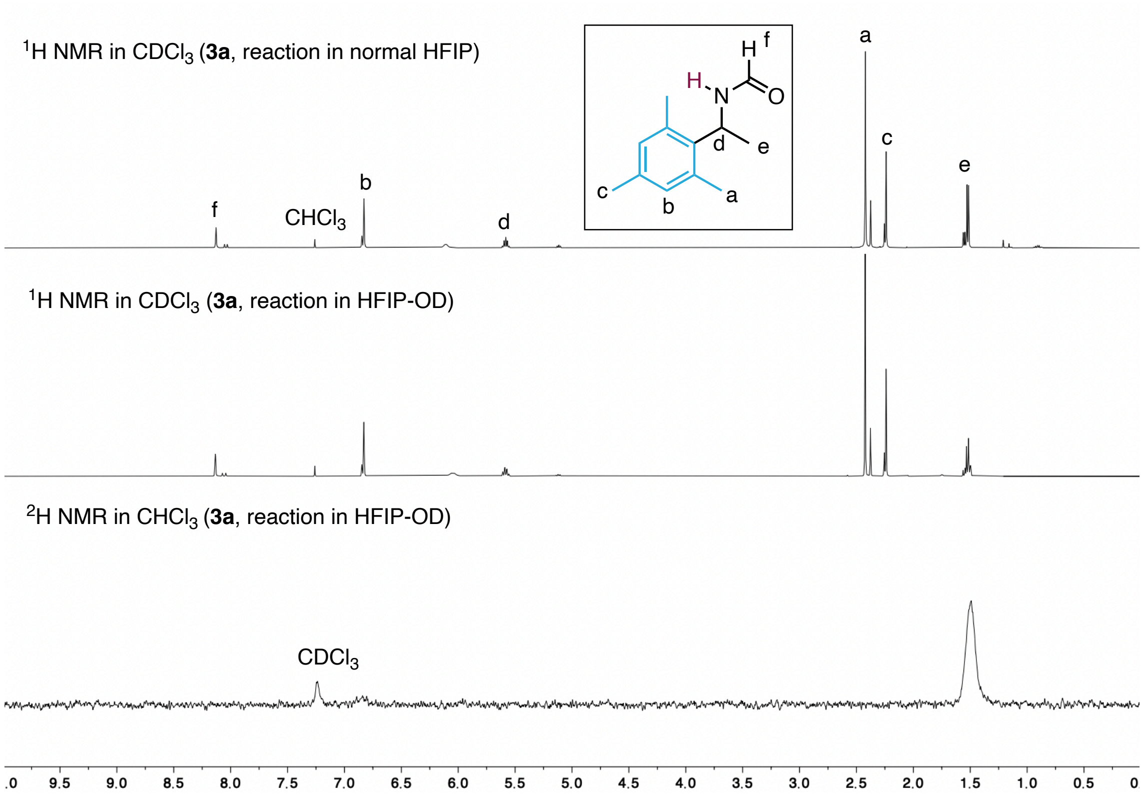

Figure S11. Comparison of ^{1}H and ^{2}H NMR spectra of **3a** obtained from the reaction in normal and deuterated HFIP.

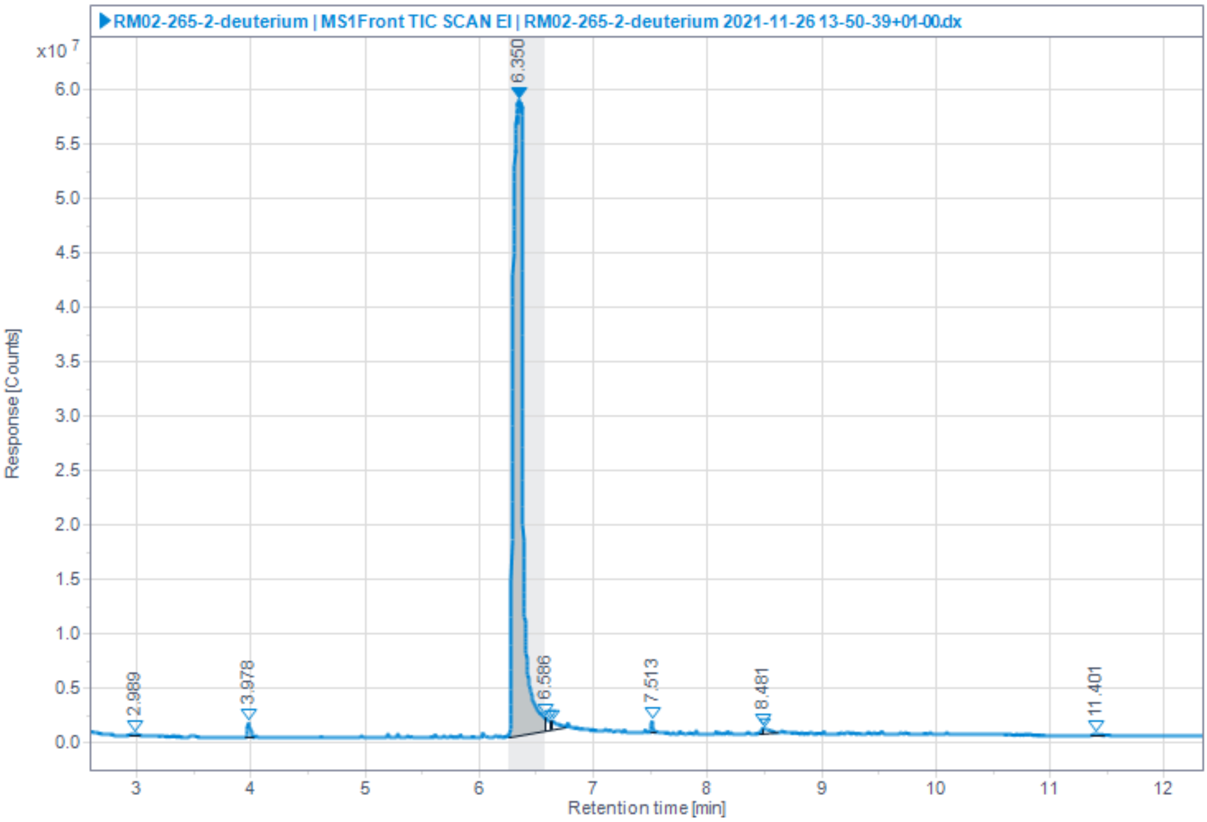

Figure S12. GC/MS chromatogram of **3a** obtained from the reaction in deuterated HFIP.

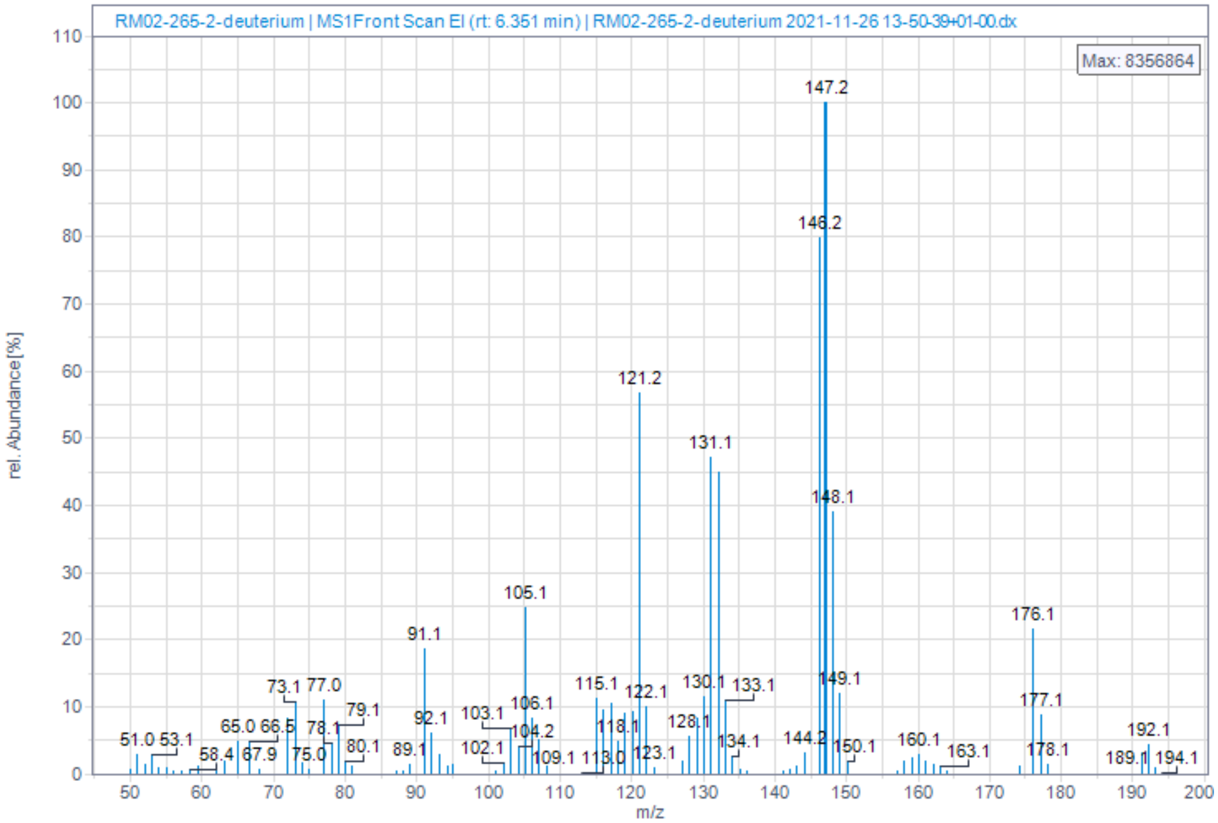

Figure S13. Mass spectrum from a GC/MS measurement of **3a** obtained from the reaction in deuterated HFIP.

Table S4. Abundance of mass fragments in the mass spectrum of **3a** obtained from the reaction in deuterated HFIP.

m/z	abundance
191.2	250304
192.1	355264
193.1	70216
194.1	9519
195	1035

The relative amounts of non-deuterated as well as mono-, di-, and tri-deuterated **3a** were estimated based on the isotopic distributions. E.g. For non-deuterated **3a**, the intensity of the signals at 191 : 192 : 193 is expected to be 100 : 13.6 : 1.

In this way, the ratio of 3 : 3.84 : 0.28 : 0.04 was calculated for the four possible isotopologues of the CH_3 group of **3a**.

IV.4 Computations (Figure 4)

Initially, all structures were subjected to a conformational search using the OPLS3[1] force field as implemented in Macromodel.[2] All obtained conformers were then optimized with the Gaussian 16 software package[3] at the MN15[4]/def2-TZVP[5] level of theory with the SMD[6] solvation model for HFIP, isopropanol or in gas phase (see the tables below for the respective method). The same level of theory was also used for the localization of the transition states. Frequency analysis level were performed to confirm that all structures correspond to minima or transition states. All transition states were additional verified by IRC calculations.

As HFIP is not by default parametrized within the SMD model implemented in Gaussian, the following parameters were used following a report by Paton and coworkers: eps = 16.7; epsinf = 1.625626; HBondAcidity = 1.96; hbondbasicity = 0.00; SurfaceTensionAtInterface = 23.23; ElectronegativeHalogenicity = 0.6; CarbonAromaticity = 0.00.[7]

Gibbs energies for unique conformers were evaluated considering Grimmes quasi-harmonic corrections for a concentration of 1 M using the Goodvibes package[8,9] (called qh-G) and weighted with a Boltzmann distribution. Structures were visualized with CYLView.[10]

Table S5. Results of DFT calculations for the reaction of **1a** with **2a** and **1a** (dimerization) at the SMD(HFIP)/MN15/def2-TZVP level of theory.

Structure	Filename (*.log)	E_{tot} (hartree)	qh-G (hartree)	weighting	ΔG (kJ mol^{-1})[a]
		Reactants			
HFIP	hfip_1	-789.496517	-789.464740	0.11422	
	hfip_2	-789.498129	-789.466672	0.88578	-5.1
		weighted:	-789.466451		
mesitylene (**2a**)	mesitylene	-349.862123	-349.711659		
**1a**-H$^+$ (*E*)	vf_iminium_e_1	-247.514114	-247.448268	0.19689	
	vf_iminium_e_2	-247.513812	-247.449594	0.80311	
		weighted:	-247.449333		
**1a**-H$^+$ (*Z*)	vf_iminium_z_1	-247.511608	-247.445688	0.87805	
	vf_iminium_z_2	-247.509853	-247.443826	0.12195	
		weighted:	-247.445461		

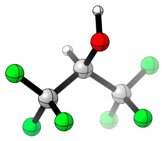

HFIP
hfip_2

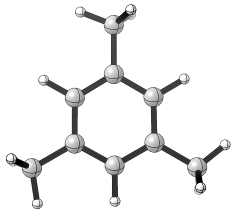

2a
mesitylene

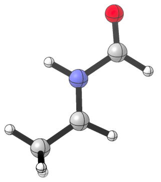

1a-H (*E*)
vf_iminium_e_2

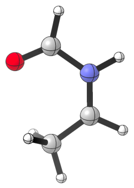

1a-H (*Z*)
vf_iminium_z_1

Structure	Filename (*.log)	E_{tot} (hartree)	qh-G (hartree)	weighting	ΔG (kJ mol^{-1})[a]
		**1a**-H$^+$ + **2a**			
TS (**1a**-H$^+$ + **2a**)	vf_mes_add_1_ts	-597.386821	-597.147194	0.00808	
	vf_mes_add_2_ts	-597.392048	-597.151713	0.97264	
	vf_mes_add_3_ts	-597.387790	-597.148015	0.01929	
	vf_mes_add_4_ts	-597.377110	-597.136898	0.00000	
		weighted:	-597.151605		24.6

	vf_mes_add_2_ts_z	-597.389967	-597.149655	1.00000	
		weighted:	-597.149655		29.8

TS with *trans*-(Z) amide conformation:

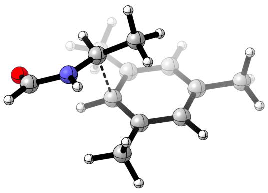
+11.9 kJ mol^{-1}
vf_mes_add_1_ts

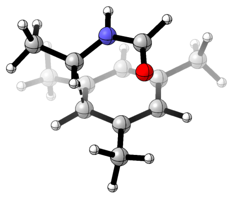
0.0 kJ mol^{-1}
vf_mes_add_2_ts

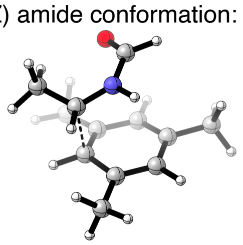
+9.7 kJ mol^{-1}
vf_mes_add_3_ts

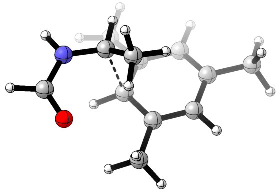
+38.9 kJ mol^{-1}
vf_mes_add_4_ts

TS with *cis*-(E) amide conformation:

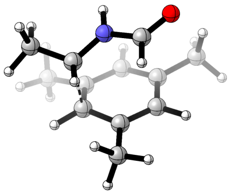
vf_mes_add_2_ts_z

Wheland-adduct	vf_mes_add_1	-597.399220	-597.157507	0.72016	
	vf_mes_add_2	-597.397895	-597.156345	0.21008	
	vf_mes_add_3	-597.395546	-597.155305	0.06975	
	vf_mes_add_4	-597.388748	-597.146996	0.00001	
		weighted:	-597.157109		10.2
		**1a**-H$^+$ + **1a**			
**1a**	vinylformamide_1	-247.089960	-247.035586	0.85558	
	vinylformamide_2	-247.088600	-247.033908	0.14442	
		weighted:	-247.035344		
TS (**1a**-H$^+$ + **1a**)	vf_dimer_ts_2	-494.614172	-494.474063	0.47710	
	vf_dimer_ts_7	-494.614220	-494.473542	0.27461	
	vf_dimer_ts_9	-494.614413	-494.473428	0.24335	
	vf_dimer_ts_13	-494.610801	-494.469754	0.00495	
		weighted:	-494.473744		28.7

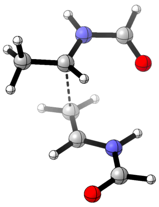
0.0 kJ mol^{-1}
vf_dimer_ts_2

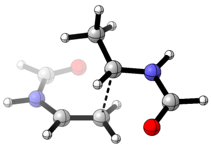
+1.4 kJ mol^{-1}
vf_dimer_ts_7

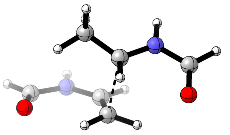
+1.7 kJ mol^{-1}
vf_dimer_ts_9

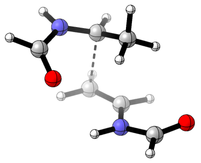
+11.3 kJ mol^{-1}
vf_dimer_ts_13

Dimer	vf_dimer_2	-494.650642	-494.507455	0.61465	
	vf_dimer_13	-494.646809	-494.503080	0.00595	
	vf_dimer_16	-494.646332	-494.502120	0.00215	
	vf_dimer_30	-494.645806	-494.502359	0.00277	
	vf_dimer_80	-494.650509	-494.506978	0.37067	

	vf_dimer_84	-494.647043	-494.501206	0.00082	
	vf_dimer_94	-494.646566	-494.502436	0.00300	
		weighted:	-494.507206		-59.2

[a] Gibbs free energy relative to the Boltzmann-weighted Gibbs energies of the reactants.

Table S6. Results of DFT calculations for the reaction of **1a** with **HFIP** and different bases at the SMD(HFIP)/MN15/def2-TZVP level of theory.

Structure	Filename (*.log)	E_{tot} (hartree)	qh-G (hartree)	weighting	ΔG (kJ mol^{-1})[a]
**1a**-H$^+$ (*E*) + HFIP with an additional molecule of HFIP acting as base					
Complex (**1a**-H$^+$ + 2 HFIP)	vf_hfip2_react_1	-1826.549456	-1826.380672	0.45785	
	vf_hfip2_react_2	-1826.549379	-1826.378448	0.04332	
	vf_hfip2_react_3	-1826.549511	-1826.380739	0.49155	
	vf_hfip2_react_4	-1826.547286	-1826.376766	0.00728	
		weighted:	-1826.380580		4.3
TS (**1a**-H$^+$ + 2 HFIP)	vf_hfip2_ts_1	-1826.54187	-1826.368877	0.62405	
	vf_hfip2_ts_2	-1826.540964	-1826.368399	0.37595	
	vf_hfip2_ts_3	-1826.530507	-1826.354970	0.00000	
		weighted:	-1826.368697		35.5

TS-conformers and their relative energies:

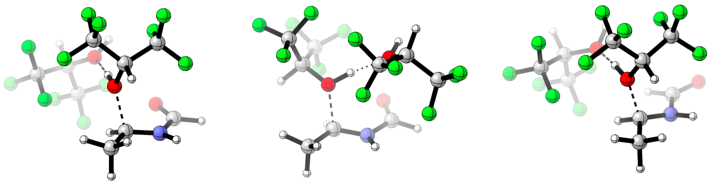

0.0 kJ mol^{-1} vf_hfip2_ts_1 | +1.3 kJ mol^{-1} vf_hfip2_ts_2 | +36.5 kJ mol^{-1} vf_hfip2_ts_3

Structure	Filename (*.log)	E_{tot} (hartree)	qh-G (hartree)	weighting	ΔG (kJ mol^{-1})[a]
Product complex (**1-HFIP** + HFIP-H$^+$)	vf_hfip2_prod_1	-1826.543933	-1826.369063	0.80145	
	vf_hfip2_prod_2	-1826.542412	-1826.367745	0.19816	
	vf_hfip2_prod_3	-1826.531001	-1826.356096	0.00000	
	vf_hfip2_prod_4	-1826.535760	-1826.361877	0.00039	
		weighted:	-1826.368799		35.3
**1a**-H$^+$ (*E*) + HFIP with an additional molecule of H_2O acting as base					
HFIP-H_2O	hfip_h2o_1	-865.894412	-865.842216	0.26442	
	hfip_h2o_2	-865.895156	-865.843181	0.73558	
		weighted:	-865.842926		
Complex (**1a**-H$^+$ + HFIP + H_2O)	vf_hfip_h2o_react_2	-1113.427734	-1113.291488	0.00090	
	vf_hfip_h2o_react_4	-1113.431687	-1113.294566	0.02341	
	vf_hfip_h2o_react_5	-1113.433656	-1113.298084	0.97569	
		weighted:	-1113.297996		-15.1
TS (**1a**-H$^+$ + HFIP + H_2O)	vf_hfip_h2o_ts_1	-1113.427613	-1113.288579	0.85807	
	vf_hfip_h2o_ts_2	-1113.424586	-1113.285337	0.02759	
	vf_hfip_h2o_ts_3	-1113.42602	-1113.286678	0.11434	
		weighted:	-1113.288272		10.5

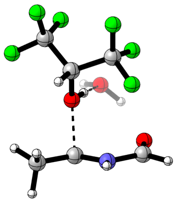

0.0 kJ mol^{-1}
vf_hfip_h2o_ts_1

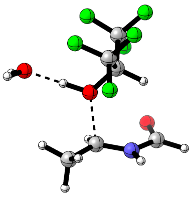

+8.5 kJ mol^{-1}
vf_hfip_h2o_ts_1

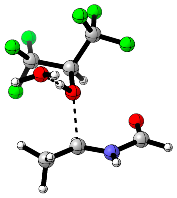

+5.0 kJ mol^{-1}
vf_hfip_h2o_ts_1

Product complex (**1a**-HFIP + H_3O^+)	vf_hfip_h2o_prod_2	-1113.444437	-1113.299764	0.59632	
	vf_hfip_h2o_prod_3	-1113.444491	-1113.299396	0.40368	
		weighted:	-1113.299615		-19.3
	vf_hfip_h2o_prod_1	-1113.455696	-1113.313232		-55.1

Note: The H_3O^+-coordinated to the reaction product can be either attached to the ether (corresponding to the initial product after addition) or to the carbonyl group. To illustrate the direct relationship of the activation energies with the thermodynamics of the reaction, we have shown in the manuscript the less favorable structure where coordiation to the ether occurs.

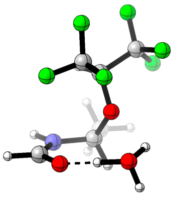

vf_hfip_h2o_prod_1

H_3O^+ coordinated to carbonyl

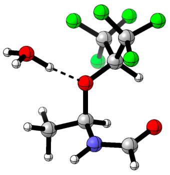

vf_hfip_h2o_prod_2

H_3O^+ coordinated to ether (direct reaction product)

**1a**-H$^+$ (*E*) + HFIP with an additional molecule of TfO$^-$ coordinated by HFIP acting as base					
TfO$^-$-HFIP	tfo_hfip_1	-1750.819039	-1750.768810	0.02196	
	tfo_hfip_2_2	-1750.817682	-1750.767554	0.00580	
	tfo_hfip_3	-1750.817810	-1750.767369	0.00476	
	tfo_hfip_5	-1750.817460	-1750.767054	0.00341	
	tfo_hfip_6	-1750.821843	-1750.771117	0.25340	
	tfo_hfip_8	-1750.821414	-1750.771095	0.24756	
	tfo_hfip_10	-1750.821309	-1750.771024	0.22961	
	tfo_hfip_11	-1750.821167	-1750.771002	0.22431	
	tfo_hfip_12	-1750.818546	-1750.767989	0.00919	
		weighted:	-1750.770933		
Complex (**1a**-H$^+$ + HFIP + TfO$^-$-HFIP)	vf_tfo_hfip_ts_1_react	-2787.884427	-2787.694324	0.00623	
	vf_tfo_hfip_ts_9_react	-2787.885708	-2787.696261	0.04861	
	vf_tfo_hfip_ts_10_react	-2787.887876	-2787.699060	0.94516	
		weighted:	-2787.698894		-32.0
TS (**1a**-H$^+$ + HFIP + TfO$^-$-HFIP)	vf_tfo_hfip_ts_1	-2787.878057	-2787.685494	0.00666	
	vf_tfo_hfip_ts_9	-2787.87664	-2787.685136	0.00455	
	vf_tfo_hfip_ts_10	-2787.879449	-2787.689079	0.29778	
	vf_tfo_hfip_ts_11	-2787.882206	-2787.689873	0.69101	
		weighted:	-2787.689586		-7.5

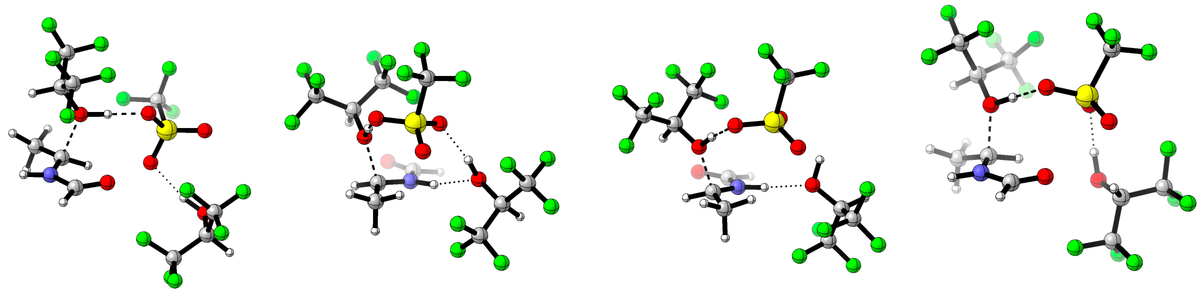

+11.5 kJ mol^{-1} vf_tfo_hfip_ts_1 | +12.4 kJ mol^{-1} vf_tfo_hfip_ts_9 | +2.1 kJ mol^{-1} vf_tfo_hfip_ts_10 | 0.0 kJ mol^{-1} vf_tfo_hfip_ts_11

Product complex (**1a**-HFIP + TfOH-HFIP)	vf_tfo_hfip_ts_1_prod	-2787.890891	-2787.696072	0.00769	
	vf_tfo_hfip_ts_9_prod	-2787.889394	-2787.693995	0.00085	
	vf_tfo_hfip_ts_10_prod	-2787.893348	-2787.699962	0.47551	
	vf_tfo_hfip_ts_11_prod	-2787.893538	-2787.700039	0.51595	
		weighted:	-2787.699967		-34.8
Overall thermochemistry for HFIP-Adduct formation					
HFIP	hfip_1	-789.496517	-789.464740	0.11422	
	hfip_2	-789.498129	-789.466672	0.88578	
		weighted:	-789.466451		
**1a**	vinylformamide_1	-247.089960	-247.035586	0.85558	
	vinylformamide_2	-247.088600	-247.033908	0.14442	
		weighted:	-247.035344		
**1a**-HFIP	vf_hfip_prod_1	-1036.628481	-1036.519842	0.99918	
	vf_hfip_prod_2	-1036.616090	-1036.507375	0.00000	
	vf_hfip_prod_4	-1036.619696	-1036.511603	0.00016	
	vf_hfip_prod_5	-1036.617037	-1036.508577	0.00001	
	vf_hfip_prod_6	-1036.615506	-1036.505737	0.00000	
	vf_hfip_prod_7	-1036.622303	-1036.512924	0.00065	
	vf_hfip_prod_8	-1036.609738	-1036.501217	0.00000	
		weighted:	-1036.519836		-47.4

Table S7. Results of DFT calculations for the isodesmic reaction of **1a-HFIP** with iPrOH to yield **4** and HFIP at the MN15/def2-TZVP level of theory in gas phase.

Structure	Filename (*.log)	E_{tot} (hartree)	qh-G (hartree)	weighting
**1a-HFIP**	gas_vf_hfip_prod_1	-1036.628352	-1036.519437	0.99853
	gas_vf_hfip_prod_2	-1036.619139	-1036.510334	0.00006
	gas_vf_hfip_prod_4	-1036.620219	-1036.512128	0.00043
	gas_vf_hfip_prod_5	-1036.618224	-1036.509957	0.00004
	gas_vf_hfip_prod_6	-1036.618261	-1036.508897	0.00001
	gas_vf_hfip_prod_7	-1036.622632	-1036.512842	0.00092
	gas_vf_hfip_prod_8	-1036.613677	-1036.504524	0.00000
		weighted:	-1036.519427	
*i*PrOH	gas_iproh_1	-194.196823	-194.113370	0.49046
	gas_iproh_2	-194.196971	-194.113406	0.50954
		weighted:	-194.113388	
**4**	gas_vf_iproh_prod_1	-441.329146	-441.169573	0.06867
	gas_vf_iproh_prod_1_2	-441.332097	-441.172005	0.90484

	gas_vf_iproh_prod_2	-441.328382	-441.167982	0.01271
	gas_vf_iproh_prod_4	-441.326490	-441.167674	0.00917
	gas_vf_iproh_prod_5	-441.326676	-441.166930	0.00417
	gas_vf_iproh_prod_6	-441.323860	-441.163864	0.00016
	gas_vf_iproh_prod_7	-441.324896	-441.164259	0.00025
	gas_vf_iproh_prod_8	-441.322487	-441.162293	0.00003
		weighted:	-441.171722	
HFIP	gas_hfip_1	-789.494909	-789.463524	0.91555
	gas_hfip_2	-789.492916	-789.461276	0.08445
		weighted:	-789.463334	

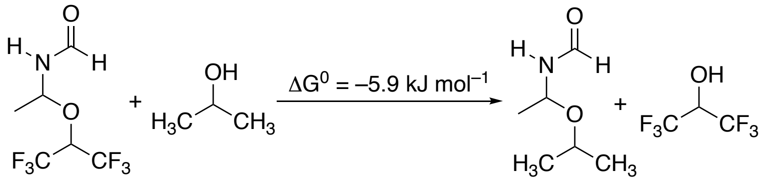

1a-HFIP **4**

Table S8. Results of DFT calculations for the reaction of **1a** with **iPrOH** with and without water acting as base at the SMD(iPrOH)/MN15/def2-TZVP level of theory.

Structure	Filename (*.log)	E_{tot} (hartree)	qh-G (hartree)	weighting	ΔG (kJ mol^{-1})[a]
		**1a**-H$^+$ (*E*) + iPrOH			
TS (**1a**-H$^+$ + *i*PrOH)	vf_iproh_4_ts1	-441.753862	-441.583960	0.15493	
	vf_iproh_5_ts1	-441.751575	-441.581179	0.00812	
	vf_iproh_6_ts1	-441.751531	-441.581718	0.01438	
	vf_iproh_7_ts1	-441.752759	-441.582878	0.04920	
	vf_iproh_8_ts1	-441.752139	-441.583007	0.05641	
	vf_iproh_9_ts1	-441.754200	-441.585405	0.71696	
		weighted:	-441.584834		32.9

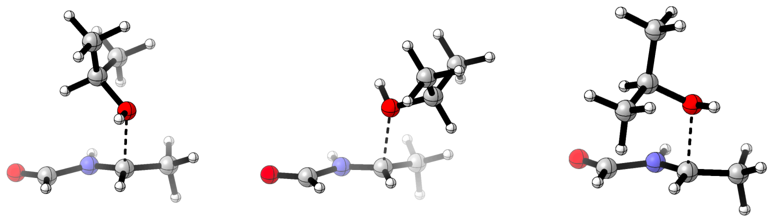

+3.8 kJ mol^{-1} vf_iproh_4_ts | +6.3 kJ mol^{-1} vf_iproh_8_ts | 0.0 kJ mol^{-1} vf_iproh_9_ts

**1a**-*i*PrOH-H$^+$	vf_iproh_add_1	-441.765845	-441.592691	0.69739	
	vf_iproh_add_2	-441.763438	-441.591836	0.28171	
	vf_iproh_add_3	-441.761200	-441.588995	0.01386	
	vf_iproh_add_4	-441.760865	-441.588357	0.00705	
		weighted:	-441.592368		13.9
		**1a**-H$^+$ (*E*) + iPrOH with an additional molecule of H_2O acting as base			
iPrOH-H_2O	iproh_h2o_1	-270.607431	-270.505371	0.44038	
	iproh_h2o_2	-270.607304	-270.505597	0.55962	
		weighted:	-270.505497		

TS (**1a**-H$^+$ + iPrOH)	vf_iproh_h2o_ts	-518.153879	-517.966941		33.4

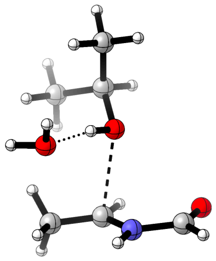

vf_iproh_h2o_ts

Product complex (**1a**-iPrOH-H$^+$)	vf_iproh_h2o_prod_1	-518.176524	-517.984350	0.03371	
	vf_iproh_h2o_prod_2	-518.179692	-517.987515	0.96629	
		weighted:	-517.987408		-20.3

[a] Gibbs free energy relative to the Boltzmann-weighted Gibbs energies of the reactants.

IV.5 Geometries in HFIP

Filename (cf. Tables S5/S6), cartesian coordinates (in Å), electronic energies (in Hartree), enthalpies (in Hartree), Gibbs free energies (in Hartree from Gaussian; for the value including qh corrections, see Table S5), and the number of the imaginary vibrational frequencies (NImag) for all stationary points at the SMD(HFIP)/MN15/def2-TZVP level of theory.

```
hfip_1.log
E =       -789.496517
H =       -789.423165
G =       -789.469080
NImag=0
O       -0.0496364       -1.2430986       -0.2092507
C       -0.5765459       -0.1719213       -0.9363964
H        0.8417976       -1.4926203       -0.5010939
C        0.2582627        1.0903951       -0.7126291
F        1.5142948        0.9496289       -1.1442156
F        0.3145526        1.3508906        0.5930499
F       -0.2658331        2.1523182       -1.3219107
C       -0.7440968       -0.5442943       -2.4112243
F       -1.3881089        0.4014008       -3.0929568
F       -1.4526896       -1.6692229       -2.4998894
F        0.4255223       -0.7605439       -3.0186654
H       -1.5761193        0.0376677       -0.5503176


hfip_2.log
E =       -789.498129
H =       -789.424987
G =       -789.470590
NImag=0
O        0.0304676       -1.3116323       -0.4168323
C       -0.6184233       -0.2155975       -0.9944709
H       -0.4780559       -1.6702073        0.3238036
C        0.2169884        1.0291121       -0.6967806
F        1.4631890        0.9151415       -1.1561767
F        0.2924916        1.1947632        0.6250900
F       -0.3145302        2.1346314       -1.2169010
C       -0.7521996       -0.5202527       -2.4850569
F       -1.2962561        0.4888114       -3.1636995
F       -1.5340823       -1.5894749       -2.6430742
F        0.4259493       -0.8032276       -3.0407568
H       -1.6224187       -0.0404652       -0.6018563


hfip_h2o_1.log
E =       -865.894412
H =       -865.794322
G =       -865.846765
NImag=0
O        0.1820044        0.8683664       -0.1060816
C       -0.9198301        0.2123330       -0.6523372
H        0.2374130        0.7656815        0.8819544
C       -0.8227505       -1.2945742       -0.4054658
C       -2.2180992        0.8367937       -0.1368255
H       -0.9153346        0.3504877       -1.7367488
O        0.3272991        0.5764013        2.5547378
F        0.3377809       -1.7414231       -0.8889068
F       -1.8016387       -1.9691375       -1.0094030
F       -0.8558363       -1.6031394        0.8931693
```

F	-3.3000904	0.2921339	-0.6942897
F	-2.2210065	2.1386581	-0.4290315
F	-2.3438029	0.7277952	1.1879509
H	1.0903014	0.0687191	2.8646449
H	0.3347554	1.4048413	3.0544617

hfip_h2o_2.log
E = -865.895156
H = -865.794923
G = -865.848098
NImag=0

O	0.2875120	1.2525633	0.9409886
C	0.0299103	-0.0782461	0.6097148
H	-0.5794739	1.7119961	1.0670162
C	0.0331608	-0.2143454	-0.9127853
C	1.1036408	-0.9128348	1.2993378
H	-0.9436078	-0.4316161	0.9645932
O	-2.2744542	1.9542151	1.0338405
F	-0.9290742	0.5634213	-1.4175180
F	-0.2012103	-1.4625230	-1.3188394
F	1.1872995	0.1825931	-1.4502532
F	1.0055563	-2.2105852	1.0100741
F	0.9820518	-0.7798356	2.6214584
F	2.3327040	-0.5120257	0.9709143
H	-2.6915902	2.1533245	1.8836369
H	-2.5744030	2.6372193	0.4178884

mesitylene.log
E = -349.862123
H = -349.668878
G = -349.716901
NImag=0

C	-0.9395084	-3.4956516	0.2679303
C	-2.1279235	-2.9155181	0.6894142
C	-2.0846805	-1.6405022	1.2628853
C	-0.8864655	-0.9564854	1.4144006
C	0.2938610	-1.5682102	0.9796776
C	0.2840910	-2.8323590	0.4066554
H	-0.9585493	-4.4861162	-0.1794311
C	-0.8438857	0.4122153	2.0303217
C	1.5532819	-3.4857950	-0.0587697
C	-3.4395425	-3.6307502	0.5387870
H	-3.0095709	-1.1769587	1.5963636
H	1.2378389	-1.0415066	1.0936323
H	-1.8409382	0.7486593	2.3174134
H	-0.2125106	0.4222024	2.9222336
H	-0.4265501	1.1435929	1.3337666
H	2.4171986	-2.8439186	0.1182836
H	1.7214041	-4.4322090	0.4611230
H	1.5105439	-3.7100785	-1.1274746
H	-4.1321041	-3.0549473	-0.0801439
H	-3.3060065	-4.6097210	0.0766782
H	-3.9212837	-3.7762420	1.5088528

tfo_hfip_1.log
E = -1750.819039
H = -1750.707972
G = -1750.776221

NImag=0

O	0.4668986	0.8160752	-0.9754017
C	0.6326958	2.9039943	0.5128831
F	-0.0850003	3.7738564	1.2195629
F	1.3154173	3.5761145	-0.4111470
F	1.5020067	2.3150871	1.3307375
O	-1.3339907	2.4578418	-1.0860685
S	-0.4516402	1.6662729	-0.2722459
O	-1.1007739	1.0467351	0.8678181
H	-2.8668381	1.1901548	0.7245380
O	-3.8196997	1.3287466	0.5118753
C	-4.4022293	0.2053892	-0.0723144
C	-3.8061245	-0.0713378	-1.4562050
C	-4.3125695	-0.9888302	0.8793354
H	-5.4646126	0.4047499	-0.2292060
F	-3.8882554	1.0306553	-2.2003586
F	-4.4676363	-1.0409060	-2.0927755
F	-2.5206295	-0.4286937	-1.4045994
F	-4.9033203	-2.0785915	0.3877432
F	-4.9201658	-0.6792145	2.0261642
F	-3.0494602	-1.3056084	1.1727525

tfo_hfip_10.log
E = -1750.821309
H = -1750.710126
G = -1750.778576
NImag=0

O	1.4284936	-1.3436043	0.8368253
C	2.7020533	0.8657535	0.4937101
F	1.6287231	1.5924444	0.8025717
F	3.4365569	1.5545942	-0.3754586
F	3.4183716	0.6863286	1.6005265
O	3.4639446	-1.3684133	-0.5120154
S	2.2086428	-0.7419399	-0.2127310
O	1.4245769	-0.3451233	-1.3664651
H	-0.2185345	-1.1427927	-1.3731036
O	-1.1306808	-1.3135715	-1.0556506
C	-1.4670529	-0.3540413	-0.0995254
C	-2.1841019	0.8000105	-0.7971205
C	-2.3129053	-1.0701409	0.9468542
H	-0.6024058	0.0686673	0.4210236
F	-3.2881861	0.3995995	-1.4301907
F	-1.3631704	1.3202662	-1.7138089
F	-2.5256133	1.7781968	0.0423955
F	-1.5686835	-1.9911343	1.5597400
F	-3.3558721	-1.6984998	0.4006990
F	-2.7804362	-0.2365796	1.8770038

tfo_hfip_11.log
E = -1750.821167
H = -1750.709918
G = -1750.778568
NImag=0

O	1.1703826	-1.1170631	0.5730794
C	3.3914465	0.1517305	0.2990718
F	4.1129684	-0.9489411	0.4968866
F	3.2366295	0.7678291	1.4686043
F	4.0702905	0.9583156	-0.5130524
O	2.1144593	-0.9122589	-1.6624851

S	1.7721015	-0.2707806	-0.4242669
O	1.1457621	1.0274991	-0.5605069
H	-0.6276966	1.5412348	-0.7471601
O	-1.5831156	1.4261859	-0.5661025
C	-1.7333165	0.3593270	0.3220443
C	-2.8949074	0.7220495	1.2399980
C	-1.9843400	-0.9368874	-0.4570647
H	-0.8537790	0.1939040	0.9521042
F	-2.5317182	1.7170346	2.0507540
F	-3.2727769	-0.3097095	1.9974862
F	-3.9618339	1.1395265	0.5579092
F	-3.2005608	-0.9671268	-1.0119832
F	-1.8674762	-2.0131242	0.3214935
F	-1.0979392	-1.0473549	-1.4469094

tfo_hfip_12.log
E = -1750.818546
H = -1750.707461
G = -1750.775016
NImag=0

O	2.4669013	-1.6703474	0.3252057
C	3.5293286	0.4118948	-0.7402884
F	4.0965782	-0.3032324	-1.7090195
F	4.4032407	0.5348860	0.2563669
F	3.2629190	1.6259275	-1.2160485
O	1.1720830	-0.4351983	-1.3315167
S	2.0035774	-0.4002373	-0.1595748
O	1.5393952	0.5079093	0.8720591
H	-0.1481173	1.0396430	0.5432920
O	-1.0494757	1.1142950	0.1583934
C	-1.9730127	0.3472433	0.8650527
C	-3.3339394	0.9709969	0.5669676
C	-1.8746696	-1.1182136	0.4232447
H	-1.8340801	0.3705880	1.9503684
F	-3.6151424	0.9498679	-0.7370531
F	-3.3298242	2.2472278	0.9540944
F	-4.3278388	0.3581331	1.2098310
F	-2.8526419	-1.8712039	0.9321088
F	-0.7155726	-1.6312107	0.8466901
F	-1.9080787	-1.2438590	-0.9021338

tfo_hfip_2_2.log
E = -1750.817682
H = -1750.706565
G = -1750.775111
NImag=0

O	3.1332727	-1.5306260	0.4250697
C	2.8825992	0.9981117	0.1058447
F	2.0144388	1.9707258	-0.1630077
F	3.8491991	1.0362774	-0.8096571
F	3.4261644	1.2419048	1.2970773
O	1.5373498	-0.7331733	-1.2353924
S	2.0619935	-0.6307819	0.0974166
O	1.0668924	-0.4822589	1.1380639
H	-0.6056436	-0.1267358	1.3927756
O	-1.5333177	0.0431967	1.6832022
C	-2.4437426	-0.0328704	0.6297037
C	-2.1626619	1.0707261	-0.3912847
C	-2.4517344	-1.4394785	0.0286288

```
H       -3.4487616        0.1464986        1.0189445
F       -0.9441549        0.9699253       -0.9219063
F       -2.2346435        2.2559414        0.2190166
F       -3.0460015        1.0755723       -1.3909940
F       -3.4000107       -1.5849969       -0.8989306
F       -2.6942078       -2.3241586        0.9976707
F       -1.2836900       -1.7650299       -0.5262014
```

```
tfo_hfip_3.log
E =     -1750.817810
H =     -1750.706797
G =     -1750.774434
NImag=0
O        1.4378816        1.4820026       -0.3239608
C        2.8288647       -0.6595021       -0.6352698
F        3.4091434       -1.6626361        0.0208866
F        3.7698501       -0.0033555       -1.3119748
F        1.9690523       -1.1759800       -1.5100250
O        3.0455984        0.8765855        1.4049007
S        1.9846190        0.4634229        0.5281907
O        1.0055233       -0.4142018        1.1355659
H       -0.6646995       -0.3544686        1.6269237
O       -1.6100769       -0.5690930        1.8020472
C       -2.4094876       -0.3701531        0.6781698
C       -2.6763596        1.1245674        0.4930754
C       -1.8337032       -1.0066491       -0.5973484
H       -3.3758936       -0.8476657        0.8520131
F       -3.4214476        1.5727686        1.5056925
F       -3.3342164        1.3755128       -0.6424610
F       -1.5516257        1.8384951        0.4831630
F       -0.9526317       -0.2254572       -1.2244964
F       -2.8110850       -1.2757718       -1.4691191
F       -1.2231461       -2.1523410       -0.3032733
```

```
tfo_hfip_5.log
E =     -1750.817460
H =     -1750.706350
G =     -1750.774326
NImag=0
O        1.3182617       -0.2488132       -1.5451043
C        3.4454421        0.0733071       -0.1371987
F        4.1408219        0.4974416       -1.1903218
F        3.6297094       -1.2392613       -0.0150380
F        3.9304507        0.6671416        0.9509852
O        1.6557094        1.8932517       -0.4371952
S        1.6758039        0.4590645       -0.3473327
O        1.1052965       -0.0807554        0.8698009
H       -0.5150280       -0.0818758        1.5100098
O       -1.4079923       -0.1124826        1.9288216
C       -2.4366615       -0.2188685        0.9933705
C       -2.4790585        1.0151567        0.0895533
C       -2.3254877       -1.5364083        0.2243928
H       -3.3906504       -0.2468488        1.5248738
F       -1.3918618        1.1301235       -0.6766975
F       -2.5474795        2.1090910        0.8494564
F       -3.5438711        1.0109010       -0.7137769
F       -1.1912954       -1.6274575       -0.4697264
F       -3.3402007       -1.7110967       -0.6245031
F       -2.3440387       -2.5496807        1.0929803
```

tfo_hfip_6.log
E = -1750.821843
H = -1750.710665
G = -1750.778257
NImag=0

O	2.8085997	-1.5003477	1.9150830
C	2.8903700	0.3070385	0.0864227
F	3.5528902	1.1597567	0.8641789
F	2.1348813	1.0080739	-0.7573263
F	3.7752077	-0.3890021	-0.6229944
O	1.1861395	-1.6047895	0.0927224
S	1.8573292	-0.8136113	1.0894829
O	0.9876756	0.1042742	1.8002152
H	-0.1890413	0.9725672	0.7669538
O	-0.8659181	1.0980885	0.0669825
C	-1.6375597	-0.0595356	-0.0330571
C	-2.9135614	0.1344028	0.7831355
C	-1.8842741	-0.2838952	-1.5209488
H	-1.1371571	-0.9528345	0.3549982
F	-2.5751754	0.3538708	2.0569270
F	-3.7111408	-0.9342071	0.7541954
F	-3.6238651	1.1865553	0.3729451
F	-2.6878923	-1.3223998	-1.7536385
F	-0.7230218	-0.5221711	-2.1314144
F	-2.4257659	0.7891157	-2.1019330

tfo_hfip_8.log
E = -1750.821414
H = -1750.710227
G = -1750.778373
NImag=0

O	3.0167421	-1.0908742	1.3820647
C	3.0736982	-0.0648109	-0.9640695
F	3.6585462	-1.1417431	-1.4847134
F	4.0248633	0.7928784	-0.5998051
F	2.3342218	0.5084270	-1.9093295
O	1.0985809	-1.4566385	-0.0778445
S	2.0541091	-0.5324962	0.4741614
O	1.4892670	0.7340137	0.8885190
H	-0.1784224	1.5014877	1.0202194
O	-1.1558811	1.5176686	0.9585374
C	-1.5956236	0.2321919	0.6382305
C	-2.9229714	0.0372873	1.3618930
C	-1.7333799	0.0808776	-0.8813299
H	-0.9174408	-0.5555547	0.9783024
F	-3.7485485	1.0683594	1.1815200
F	-2.7081631	-0.0710195	2.6739110
F	-3.5532114	-1.0669588	0.9551543
F	-1.8418822	-1.2002181	-1.2385989
F	-0.6632651	0.5885687	-1.4878228
F	-2.8031789	0.7261337	-1.3612293

vf_dimer_13.log
E = -494.646809
H = -494.458972
G = -494.506694
NImag=0

C	-1.5853097	0.8100437	1.4713122
C	-1.5810991	0.9576725	-0.0452886
N	-1.9899051	-0.2560514	-0.7594590
C	-1.6875440	-1.5045428	-0.4156862
H	-2.6762395	-0.1449753	-1.4963205
O	-0.8513066	-1.8088803	0.4479921
H	-2.2344112	-2.2743549	-0.9689764
C	-0.2966767	1.5932589	-0.5741148
C	1.0091422	0.9596437	-0.3853326
N	1.2419360	-0.2245507	0.0676995
C	2.5971867	-0.7081907	0.1694434
O	3.5235252	-0.0186066	-0.1311737
H	2.6564466	-1.7345135	0.5394037
H	0.4541606	-0.8801211	0.3438038
H	-1.6089586	1.8078090	1.9115022
H	-2.4772989	0.2719814	1.7946918
H	-0.7153954	0.2895585	1.8674311
H	-2.3592102	1.6791904	-0.2927071
H	-0.3575268	1.7782358	-1.6567126
H	-0.1922177	2.6006243	-0.1516622
H	1.8892511	1.5347520	-0.6696210

vf_dimer_16.log
E = -494.646332
H = -494.457451
G = -494.506049
NImag=0

C	-2.9515510	-0.7444765	-0.1895732
C	-1.5720250	-0.7857613	0.4412375
N	-1.2369177	0.4837490	1.0923809
C	-0.9821746	1.6096149	0.4093344
H	-1.4088846	0.5664115	2.0870876
O	-0.8502140	1.6522739	-0.8131967
H	-0.8704186	2.5003674	1.0400549
C	-0.5288537	-1.1743546	-0.6314347
C	0.8134234	-0.8758029	-0.1324006
N	1.5815626	0.0112259	-0.6688964
C	2.8923782	0.3044815	-0.1312554
O	3.3091545	-0.3030960	0.8044576
H	3.4048738	1.1059128	-0.6683916
H	1.2398250	0.5718967	-1.4564277
H	-2.9973740	0.0448306	-0.9430980
H	-3.1780563	-1.6987274	-0.6677417
H	-3.7076219	-0.5502553	0.5721229
H	-1.5411705	-1.5380812	1.2284652
H	-0.5929092	-2.2576584	-0.7725510
H	-0.7229097	-0.6669295	-1.5746605
H	1.2161212	-1.3925274	0.7375907

vf_dimer_2.log
E = -494.650642
H = -494.462981
G = -494.511231
NImag=0

C	2.8218901	-1.2584120	-0.4467855
C	1.5148205	-0.5057538	-0.2758398
N	1.7416713	0.6482739	0.5903449
C	1.2253692	1.8521122	0.3502852
H	2.4594106	0.5717138	1.3034368

O	0.3634346	2.0680862	-0.5144886
H	1.6129888	2.6562059	0.9826269
C	0.4532227	-1.4568095	0.2662122
C	-0.9512339	-1.0526441	0.2422253
N	-1.4042528	0.0743046	-0.1911187
C	-2.8226464	0.3384719	-0.1884195
O	-3.6032444	-0.4941209	0.1589135
H	-3.0683601	1.3466535	-0.5305985
H	-0.7572560	0.8493637	-0.5033396
H	3.5916897	-0.6020491	-0.8524706
H	3.1653330	-1.6500201	0.5141959
H	2.6877071	-2.0975398	-1.1295037
H	1.1908997	-0.1147554	-1.2439797
H	0.4716464	-2.4020531	-0.2949363
H	0.6725448	-1.7654528	1.2973306
H	-1.6979938	-1.7605942	0.5974652

vf_dimer_30.log
E = -494.645806
H = -494.457313
G = -494.506586
NImag=0

C	-3.0464491	-0.3730345	-0.3870125
C	-1.7830757	-0.5981386	0.4234399
N	-1.3031176	0.6438730	1.0348616
C	-0.7717112	1.6488205	0.3221340
H	-1.5727445	0.8361459	1.9919352
O	-0.4948242	1.5649469	-0.8733772
H	-0.5740252	2.5566211	0.9056921
C	-0.7091850	-1.2492418	-0.4767529
C	0.6034247	-1.1434722	0.1609919
N	1.5766695	-0.4660853	-0.3479353
C	2.8567603	-0.3524189	0.2886114
O	3.7122698	0.3067430	-0.2145763
H	2.9304880	-0.9125354	1.2263939
H	1.4445312	0.0672233	-1.2167678
H	-2.8575228	0.3482359	-1.1851874
H	-3.3811053	-1.3100721	-0.8348485
H	-3.8420743	0.0093644	0.2534453
H	-1.9836315	-1.2767239	1.2515909
H	-0.9479482	-2.3145747	-0.5541421
H	-0.6984910	-0.8021259	-1.4686310
H	0.7974361	-1.6395077	1.1107377

vf_dimer_80.log
E = -494.650509
H = -494.462841
G = -494.510682
NImag=0

C	3.0487855	-0.5716082	-0.5351718
C	1.5811521	-0.2623401	-0.3020271
N	1.4749031	0.8942220	0.5863444
C	0.6288216	1.9048379	0.3872439
H	2.2073169	1.0233273	1.2769298
O	-0.2930195	1.8776713	-0.4424226
H	0.7943361	2.7774269	1.0254103
C	0.8844584	-1.5024313	0.2499119
C	-0.5769181	-1.5038361	0.3183608
N	-1.3224381	-0.5316019	-0.0790618

C	-2.7440161	-0.5566653	0.0424170
O	-3.3708379	0.4136883	-0.2612569
H	-3.1530077	-1.4986687	0.4239269
H	-0.9184433	0.3883151	-0.4299099
H	3.1527253	-1.3994595	-1.2365037
H	3.5617064	0.2973001	-0.9473730
H	3.5339403	-0.8533262	0.4029104
H	1.1185728	0.0232003	-1.2504549
H	1.1414184	-2.3838947	-0.3541335
H	1.2474663	-1.7557801	1.2546541
H	-1.0803615	-2.3821749	0.7179775

vf_dimer_84.log
E = -494.647043
H = -494.457670
G = -494.504839
NImag=0

C	-2.7169108	-0.0526438	-0.9568412
C	-1.8069104	-0.4948575	0.1731458
N	-1.3695537	0.6416888	0.9910642
C	-0.5016250	1.5669232	0.5590982
H	-1.9054491	0.8563223	1.8235748
O	0.1389057	1.4667042	-0.4879701
H	-0.3818393	2.4210313	1.2363246
C	-0.6092609	-1.2826618	-0.4087335
C	0.4351284	-1.3537904	0.6172743
N	1.6219871	-0.8523493	0.5371475
C	2.1909434	-0.1214582	-0.5717955
O	3.1921415	0.4969485	-0.3729608
H	1.6924784	-0.2451034	-1.5317318
H	2.2429514	-0.9025624	1.3505634
H	-3.6096940	0.4294444	-0.5567968
H	-2.1935261	0.6540418	-1.6050532
H	-3.0242271	-0.9133063	-1.5531067
H	-2.3427790	-1.1553398	0.8536436
H	-0.9484469	-2.3073137	-0.5895972
H	-0.2749006	-0.8459101	-1.3437486
H	0.1999820	-1.8412596	1.5601320

vf_dimer_94.log
E = -494.646566
H = -494.458746
G = -494.506097
NImag=0

C	1.6388486	-0.4000147	1.5430302
C	1.7674354	-0.6240585	0.0408923
N	1.8098302	0.6198443	-0.7377900
C	1.1183168	1.7294509	-0.4910185
H	2.5454731	0.6975789	-1.4300761
O	0.1669886	1.8014391	0.3029661
H	1.4420703	2.6047152	-1.0618622
C	0.7917962	-1.6731378	-0.4942499
C	-0.6564008	-1.4667403	-0.4103931
N	-1.2401969	-0.3755815	-0.0563132
C	-2.6600149	-0.2415172	-0.0503612
O	-3.1462834	0.8096271	0.2433457
H	-3.2068873	-1.1499059	-0.3290231
H	-0.6923571	0.5066772	0.2090573
H	1.9453000	-1.3151446	2.0515500

H	2.3034217	0.4051377	1.8589043
H	0.6320811	-0.1545130	1.8764891
H	2.7500374	-1.0654247	-0.1246027
H	0.9794296	-1.8875312	-1.5562972
H	0.9856463	-2.6323340	0.0012036
H	-1.2987100	-2.2998851	-0.6906564

vf_dimer_ts_13.log
E = -494.610801
H = -494.425168
G = -494.473727
NImag=1

C	0.2899932	-0.4553238	1.5903070
C	1.2173081	-1.0345835	0.5939214
N	2.3349152	-0.3952233	0.2135559
C	2.3901895	0.9739258	-0.0645491
H	3.1224030	-0.9530539	-0.1119353
O	1.3912334	1.6400308	-0.1745523
H	3.4066060	1.3501107	-0.2136769
C	0.1907222	-1.0968074	-1.4819588
C	-0.9944578	-0.7814901	-0.9141425
N	-1.4531384	0.4946109	-0.7560908
C	-2.4944446	0.7773346	0.0965660
O	-3.0836579	-0.0785643	0.7257677
H	-2.7383253	1.8433985	0.1521966
H	-0.9043543	1.2717184	-1.1174327
H	-0.6175303	-1.0494568	1.6791533
H	0.8404848	-0.5443230	2.5369186
H	0.0565616	0.5952326	1.4426347
H	1.2981975	-2.1141270	0.5699595
H	0.7935645	-0.3313480	-1.9566764
H	0.4289507	-2.1328855	-1.6763370
H	-1.6478402	-1.5428986	-0.5032569

vf_dimer_ts_2.log
E = -494.614172
H = -494.428663
G = -494.478345
NImag=1

C	1.5738816	-2.1382520	-0.5620723
C	1.3328086	-0.6885883	-0.4548926
N	2.3455821	0.1346266	-0.1625158
C	2.1469027	1.5158251	-0.1405024
H	3.2289829	-0.2430689	0.1770684
O	1.0384256	1.9810330	-0.2493716
H	3.0589608	2.1012565	0.0017944
C	0.1311207	-0.5348982	1.4722727
C	-1.0449137	-0.5476312	0.8008629
N	-1.6573972	0.5714904	0.3196864
C	-2.8184803	0.5059007	-0.4172060
O	-3.3883568	-0.5368273	-0.6640660
H	-3.1767760	1.4846687	-0.7528449
H	-1.2179632	1.4797390	0.4525021
H	2.0209054	-2.3051516	-1.5498946
H	2.2700302	-2.4947995	0.1960779
H	0.6401082	-2.6937819	-0.5196540
H	0.5418213	-0.2275413	-1.0334296
H	0.5040428	-1.4526055	1.9054915
H	0.5586442	0.4035218	1.8054932

H -1.5556967 -1.4723342 0.5539112

vf_dimer_ts_7.log
E = -494.614220
H = -494.428322
G = -494.477884
NImag=1

C	-0.0486223	1.6139169	0.9110203
C	0.9356164	0.5558359	0.6192451
N	1.9443865	0.8286834	-0.2197481
C	3.0131397	-0.0532088	-0.3780634
H	1.8881065	1.6403670	-0.8319944
O	3.0420168	-1.1139554	0.1958923
H	3.7875189	0.3067614	-1.0611268
C	-0.1651298	-0.9397410	-0.6108274
C	-1.0819141	-1.3414561	0.3093201
N	-2.3472882	-0.8656100	0.5041893
C	-2.9583151	0.1440549	-0.2043538
O	-2.4062080	0.7849910	-1.0731863
H	-3.9960402	0.3143700	0.1036589
H	-2.8950874	-1.2915453	1.2448929
H	0.4473243	2.2983668	1.6107920
H	-0.3281570	2.1725077	0.0185634
H	-0.9289883	1.2232676	1.4148006
H	1.1650819	-0.1963242	1.3633099
H	-0.4067066	-0.2555809	-1.4101345
H	0.7210593	-1.5577360	-0.6970576
H	-0.8166732	-2.1220330	1.0131245

vf_dimer_ts_9.log
E = -494.614413
H = -494.428387
G = -494.477455
NImag=1

C	-0.0744322	1.8423666	-0.0183235
C	0.9255569	0.7979271	0.2662835
N	1.9162118	0.5695254	-0.6074366
C	2.9980557	-0.2459486	-0.2776371
H	1.8449231	0.9199535	-1.5604042
O	3.0633865	-0.8045319	0.7896767
H	3.7491236	-0.3263670	-1.0691296
C	-0.2034120	-1.1038653	0.5499824
C	-0.8251898	-1.0150081	-0.6543334
N	-2.0653236	-0.5131290	-0.9329684
C	-2.9760186	-0.0251674	-0.0245238
O	-2.7557015	0.0735554	1.1640161
H	-3.9239766	0.2699717	-0.4883994
H	-2.3569263	-0.5140673	-1.9050241
H	-0.3984979	1.8418161	-1.0588807
H	-0.9243074	1.7660181	0.6565137
H	0.4243165	2.7988762	0.1800965
H	1.1808849	0.5770296	1.2962384
H	0.7141136	-1.6788458	0.5938190
H	-0.6954177	-0.8578613	1.4788118
H	-0.3059991	-1.3548780	-1.5435252

vf_hfip2_prod_1.log
E = -1826.543933

H = -1826.294629
G = -1826.376447
NImag=0

C	-0.5455309	1.4811492	1.6614833
C	-1.0170280	0.9948583	2.9935540
N	-1.0925354	2.7078885	1.2592074
C	-0.6572755	3.2728751	0.0949692
H	-1.1481274	4.2163550	-0.1661062
O	0.2066918	2.7518342	-0.5878038
H	0.5370843	1.4912038	1.5684216
H	-0.6442371	-0.0108516	3.1808626
H	-0.6065160	1.6636616	3.7507646
H	-2.1044642	1.0132163	3.0780545
H	-1.9255731	3.0629270	1.7167752
O	-0.8615703	0.4171075	0.5930235
C	-2.1536752	-0.0913734	0.3352765
H	-0.0805030	0.2066413	-0.0753790
C	-2.7081115	0.4907163	-0.9789280
C	-2.0934062	-1.6321996	0.3149297
H	-2.8231032	0.2049471	1.1438418
O	0.9665313	-0.1939451	-1.0080877
F	-3.0871004	1.7519531	-0.8035987
F	-3.7587100	-0.2110748	-1.3831470
F	-1.7932931	0.4759402	-1.9460891
F	-3.3082559	-2.1369947	0.4826359
F	-1.3163505	-2.0652207	1.2980682
F	-1.6050459	-2.0946747	-0.8356019
C	2.3031519	-0.5441603	-0.7885606
H	0.7697368	-0.0885137	-1.9532031
C	3.1439095	0.7199760	-0.5707112
C	2.3280842	-1.5222378	0.3859193
H	2.7199121	-1.0681475	-1.6505277
F	2.9401877	1.5579145	-1.5849019
F	4.4402709	0.4286708	-0.5233079
F	2.8242398	1.3604884	0.5535311
F	3.5686215	-1.8905311	0.6811659
F	1.6295460	-2.6078213	0.0643947
F	1.7771492	-1.0022250	1.4852976

vf_hfip2_prod_2.log
E = -1826.542412
H = -1826.292847
G = -1826.375514
NImag=0

C	0.7249059	1.6895142	1.7542349
C	0.8405425	1.0944968	3.1176235
N	-0.5349616	2.2107831	1.4616042
C	-0.7460939	2.8681755	0.2789833
H	-1.7555719	3.2752628	0.1591369
O	0.1327098	2.9814710	-0.5536379
H	1.5167606	2.3848500	1.4835261
H	1.8098920	0.6162499	3.2521470
H	0.7604928	1.9043194	3.8442705
H	0.0431020	0.3712282	3.2900897
H	-1.3252954	1.9481715	2.0424636
O	0.9716362	0.5184275	0.7291133
C	2.2867120	0.1206011	0.3944398
H	0.2116237	0.3436806	0.0519971
C	2.3267834	-1.4158160	0.3059965
C	2.7701378	0.8130707	-0.8912416

H	2.9550245	0.4122073	1.2062451
O	-0.9193160	0.0987138	-0.9407141
F	1.6469827	-1.9349882	1.3200854
F	3.5794688	-1.8459458	0.3754051
F	1.7932765	-1.8636135	-0.8291737
F	3.8907744	0.2416024	-1.3178051
F	3.0152414	2.0964386	-0.6621056
F	1.8644352	0.7326892	-1.8623663
C	-2.1037968	-0.6443601	-0.8686023
H	-0.7337954	0.4017335	-1.8451668
C	-1.9380602	-1.6699564	0.2537549
C	-3.2942141	0.2978908	-0.6408436
H	-2.2943697	-1.1949848	-1.7922262
F	-1.0335699	-2.5781077	-0.0977227
F	-3.0824088	-2.2926172	0.5099710
F	-1.5156530	-1.0983377	1.3847059
F	-4.4492030	-0.3371797	-0.8059918
F	-3.2396570	1.2970040	-1.5196895
F	-3.2903217	0.8347660	0.5811423

vf_hfip2_prod_3.log
E = -1826.531001
H = -1826.281556
G = -1826.363695
NImag=0

C	-0.5429969	1.2883801	1.6724715
C	-0.9033554	0.6858787	2.9878397
N	-1.1751117	2.4863304	1.3638307
C	-0.7918358	3.1997699	0.2547989
H	0.1848264	2.8935105	-0.1521189
O	-1.4661926	4.0833163	-0.2258020
H	0.5271518	1.3429853	1.4848205
H	-0.4718784	-0.3092745	3.0804091
H	-0.4846774	1.3236164	3.7672163
H	-1.9841941	0.6410043	3.1300178
H	-2.1127148	2.6566514	1.7241549
O	-0.8926972	0.2180156	0.5591611
C	-2.1832515	-0.3029101	0.3177112
H	-0.1490191	0.1202426	-0.1567496
C	-2.8350156	0.3958645	-0.8943970
C	-2.0585184	-1.8252761	0.1346787
H	-2.8079312	-0.1266579	1.1956851
O	1.0206274	0.0808396	-1.0765201
F	-3.3121048	1.5860308	-0.5449457
F	-3.8355624	-0.3300907	-1.3722710
F	-1.9494940	0.5880052	-1.8707837
F	-3.2488731	-2.4037537	0.1980055
F	-1.2970759	-2.3286912	1.0974548
F	-1.5030374	-2.1291005	-1.0391036
C	2.3174357	-0.4122583	-0.8747682
H	0.8355470	0.2334987	-2.0177446
C	3.2186776	0.7474076	-0.4381896
C	2.2471654	-1.5567962	0.1382869
H	2.7362526	-0.8245062	-1.7942763
F	3.1431858	1.7158804	-1.3490224
F	4.4865647	0.3693042	-0.3330074
F	2.8418708	1.2697926	0.7304247
F	3.4516333	-2.0651014	0.3673838
F	1.4661723	-2.5203590	-0.3411714
F	1.7319552	-1.1697864	1.3077104

vf_hfip2_prod_4.log
E = -1826.535760
H = -1826.286527
G = -1826.369588
NImag=0

C	-0.8290726	-1.5198058	1.7677026
C	-0.9111917	-0.7754288	3.0550072
N	0.3853526	-2.1083313	1.4942936
C	0.5364033	-2.9937997	0.4494918
H	-0.4157947	-3.3385443	0.0201375
O	1.6187818	-3.3559822	0.0512662
H	-1.6685500	-2.1749165	1.5492069
H	-1.8417904	-0.2148494	3.1269432
H	-0.8935961	-1.5111191	3.8610406
H	-0.0617878	-0.1013992	3.1659143
H	1.2305684	-1.6914635	1.8823313
O	-1.0517129	-0.3923433	0.6054124
C	-2.3432514	0.0548472	0.2568306
H	-0.2698397	-0.2606412	-0.0450136
C	-2.3645088	1.5913005	0.2512745
C	-2.7984719	-0.5880918	-1.0599748
H	-3.0390770	-0.2733436	1.0338719
O	0.9704452	-0.1642257	-0.9816704
F	-1.8226339	2.0391063	1.3787243
F	-3.6138490	2.0315401	0.1767160
F	-1.6804548	2.1047956	-0.7662666
F	-4.0460117	-0.2372322	-1.3374787
F	-2.7440456	-1.9125434	-0.9377669
F	-2.0278585	-0.2457701	-2.0882660
C	2.1703426	0.5564563	-0.9158093
H	0.7717938	-0.4378501	-1.8918545
C	2.0252653	1.6086296	0.1847182
C	3.3394645	-0.4068391	-0.6681280
H	2.3776459	1.0870027	-1.8474050
F	1.1409410	2.5294471	-0.1870632
F	3.1825675	2.2130343	0.4275300
F	1.5890910	1.0765773	1.3300492
F	4.5087450	0.2049979	-0.8348460
F	3.2713048	-1.4109300	-1.5381119
F	3.3187170	-0.9239896	0.5608341

vf_hfip2_react_1.log
E = -1826.549456
H = -1826.301249
G = -1826.390423
NImag=0

C	-0.6299986	1.8816202	1.9734857
C	-1.0732432	1.4956281	3.3021810
N	-1.2408908	2.7527172	1.2354784
C	-0.8256681	3.0155218	-0.1180990
H	-1.3847930	3.8278615	-0.5891181
O	0.0408110	2.3703812	-0.6234031
H	0.2833049	1.4649497	1.5607592
H	-1.1658148	0.4041242	3.3092573
H	-0.2677285	1.7282202	4.0067484
H	-2.0041737	1.9708223	3.6009111
H	-2.0852488	3.2165592	1.5768803
O	-1.1661087	-0.2593526	0.5087775

C	-2.3053194	-0.8121355	-0.0566000
H	-0.3966399	-0.3026487	-0.1001173
C	-2.7683119	0.0468769	-1.2372196
C	-2.0790752	-2.2813323	-0.4337708
H	-3.1089214	-0.8002213	0.6827208
O	1.0515417	-0.3843336	-1.2463881
F	-3.1320090	1.2528040	-0.7867016
F	-3.8082742	-0.4801497	-1.8758071
F	-1.7906201	0.2416476	-2.1252910
F	-3.2237350	-2.8942739	-0.7286148
F	-1.5244398	-2.9182871	0.5961167
F	-1.2595747	-2.4208307	-1.4801964
C	2.3779281	-0.6350561	-0.9103829
H	0.9328404	-0.3330099	-2.2065903
C	3.0686471	0.6775617	-0.5221362
C	2.3578211	-1.6646166	0.2188113
H	2.9520200	-1.0670858	-1.7334611
F	2.8850091	1.5734952	-1.4904614
F	4.3794784	0.5085378	-0.3578662
F	2.5822375	1.2013926	0.6059498
F	3.5760511	-1.9031451	0.6965019
F	1.8562861	-2.8104080	-0.2389880
F	1.5854998	-1.2716377	1.2357305

vf_hfip2_react_2.log
E = -1826.549379
H = -1826.300390
G = -1826.386957
NImag=0

C	0.1272621	2.5367739	1.6391800
C	0.0503550	2.2756092	3.0674320
N	-0.9073324	2.5411555	0.8604413
C	-0.7788273	2.6849154	-0.5678767
H	-1.7388312	2.7854904	-1.0808528
O	0.2990030	2.6936166	-1.0774985
H	1.0785572	2.7685956	1.1700677
H	0.6709565	1.3929514	3.2625938
H	0.5302987	3.1019921	3.5992708
H	-0.9653260	2.1162729	3.4210758
H	-1.8380963	2.3680309	1.2459747
O	1.2296656	0.2179152	0.8117454
C	2.5118817	-0.1815268	0.4549304
H	0.5764668	0.0209262	0.1074390
C	2.5418337	-1.6960617	0.2283076
C	3.0047065	0.6261473	-0.7512147
H	3.1937709	0.0307856	1.2808821
O	-0.9346869	-0.1308282	-0.9654795
F	2.0916693	-2.3107517	1.3208960
F	3.7670511	-2.1511073	-0.0197639
F	1.7543236	-2.0578115	-0.7907115
F	4.2126794	0.2449146	-1.1596482
F	3.0776900	1.9153498	-0.4181107
F	2.1703569	0.5256153	-1.7904606
C	-2.0447581	-0.9429863	-0.7366149
H	-0.6386237	-0.1932989	-1.8878064
C	-1.9411516	-1.4446205	0.7051834
C	-3.3283001	-0.1426912	-0.9922316
H	-2.0741052	-1.8203125	-1.3874438
F	-0.9278427	-2.3010321	0.8104057
F	-3.0530029	-2.0645413	1.0890467

F	-1.7082584	-0.4467103	1.5635932
F	-4.4049419	-0.9217040	-0.9913799
F	-3.2469094	0.4499882	-2.1828733
F	-3.5177689	0.8181504	-0.0815084

vf_hfip2_react_3.log
E = -1826.549511
H = -1826.301075
G = -1826.390979
NImag=0

C	-0.4996675	2.1518005	2.1307923
C	-0.9232745	1.8113392	3.4784175
N	-1.1028555	3.0222196	1.3855774
C	-0.7138477	3.2388022	0.0166899
H	-1.2607101	4.0550310	-0.4619652
O	0.1203335	2.5553044	-0.4930233
H	0.3912130	1.6996653	1.7075759
H	-1.0475126	0.7236910	3.5141018
H	-0.0958333	2.0360384	4.1598296
H	-1.8335575	2.3198024	3.7863696
H	-1.9271621	3.5162691	1.7337266
O	-1.1234948	0.0030441	0.7250662
C	-2.3001325	-0.5331407	0.2250424
H	-0.3948486	-0.0492451	0.0681095
C	-2.7963329	0.3156731	-0.9496126
C	-2.1248069	-2.0131368	-0.1391948
H	-3.0675288	-0.4905907	1.0006177
O	0.9994758	-0.2428877	-1.1162020
F	-3.1266731	1.5330397	-0.5047138
F	-3.8652463	-0.2070980	-1.5425278
F	-1.8488015	0.4838616	-1.8755573
F	-3.2985957	-2.6111223	-0.3342838
F	-1.5034667	-2.6414954	0.8567707
F	-1.3913988	-2.1856262	-1.2429814
C	2.3286932	-0.5367252	-0.8276472
H	0.8441401	-0.2030017	-2.0714124
C	3.0803408	0.7565199	-0.4893594
C	2.3155888	-1.5478655	0.3184190
H	2.8525327	-1.0014767	-1.6661672
F	2.8959708	1.6400039	-1.4694002
F	4.3891004	0.5439298	-0.3662071
F	2.6532074	1.3201924	0.6434180
F	3.5431661	-1.8349836	0.7427461
F	1.7425077	-2.6763349	-0.0966079
F	1.6103651	-1.1070101	1.3635011

vf_hfip2_react_4.log
E = -1826.547286
H = -1826.298671
G = -1826.385184
NImag=0

C	0.1878164	2.5479596	1.6636493
C	-0.0331321	2.2232236	3.0639700
N	-0.7534332	2.5728259	0.7754940
C	-0.4829137	2.7782772	-0.6141863
H	0.5537231	3.0688522	-0.8180713
O	-1.3492899	2.6317548	-1.4200020
H	1.1829543	2.8131895	1.3182932
H	0.5861348	1.3490729	3.2953261

H	0.3663782	3.0414488	3.6707511
H	-1.0764127	2.0324141	3.3023647
H	-1.7164811	2.3295527	1.0267998
O	1.2086707	0.1773278	0.8490063
C	2.4837750	-0.1719821	0.4197829
H	0.5307171	-0.0432233	0.1745372
C	2.5400291	-1.6687582	0.1054495
C	2.8877656	0.7167880	-0.7602712
H	3.1979075	0.0148190	1.2244015
O	-0.9311128	-0.2813356	-0.9262302
F	2.1596748	-2.3575084	1.1797524
F	3.7619976	-2.0728721	-0.2289670
F	1.7106568	-1.9894677	-0.8938329
F	4.0736420	0.4015756	-1.2681096
F	2.9441643	1.9869358	-0.3477445
F	1.9863792	0.6639219	-1.7470341
C	-2.0778521	-1.0254980	-0.6594292
H	-0.6730655	-0.3576898	-1.8583509
C	-1.9820091	-1.4641874	0.8021704
C	-3.3295648	-0.1848090	-0.9471877
H	-2.1512553	-1.9308393	-1.2674010
F	-0.9892099	-2.3411362	0.9413169
F	-3.1061012	-2.0364270	1.2204430
F	-1.7170258	-0.4342396	1.6130326
F	-4.4350738	-0.9240446	-0.8963907
F	-3.2384773	0.3376359	-2.1691385
F	-3.4773710	0.8263057	-0.0869859

vf_hfip2_ts_1.log
E = -1826.541870
H = -1826.293921
G = -1826.376826
NImag=1

C	-0.6953828	1.7493503	1.6403666
C	-1.2598845	1.3118277	2.9325235
N	-1.2476489	2.8149117	1.0223668
C	-0.7444311	3.2494440	-0.2013049
H	-1.2686998	4.1153347	-0.6167330
O	0.1825924	2.6905048	-0.7359737
H	0.3746446	1.6506893	1.4913046
H	-0.8774315	0.3302891	3.2036305
H	-0.9223948	2.0319566	3.6834251
H	-2.3504679	1.3138046	2.9201284
H	-2.1631679	3.1477143	1.3167228
O	-0.9713238	0.3162628	0.5692734
C	-2.1965121	-0.2712387	0.2458770
H	-0.2187988	0.1656847	-0.0798325
C	-2.7191533	0.2728183	-1.0929371
C	-2.0515178	-1.8034393	0.2515379
H	-2.9256782	-0.0158978	1.0179417
O	1.0255722	-0.2093739	-1.0657564
F	-3.1602143	1.5200859	-0.9330165
F	-3.7196071	-0.4658731	-1.5563422
F	-1.7586688	0.3065802	-2.0168666
F	-3.2428888	-2.3871287	0.3177090
F	-1.3428118	-2.1780950	1.3104238
F	-1.4309631	-2.2522916	-0.8406133
C	2.3535432	-0.5189464	-0.7750815
H	0.8789579	-0.1100166	-2.0190346
C	3.1537032	0.7725703	-0.5605029

C	2.3314366	-1.4394294	0.4450856
H	2.8358153	-1.0692713	-1.5859291
F	2.9897157	1.5660348	-1.6184353
F	4.4536294	0.5210088	-0.4286636
F	2.7559441	1.4517410	0.5163305
F	3.5591820	-1.7546126	0.8432596
F	1.6888609	-2.5634224	0.1354972
F	1.6902738	-0.8855463	1.4776750

vf_hfip2_ts_2.log
E = -1826.540964
H = -1826.292669
G = -1826.377481
NImag=1

C	0.8635905	1.8741345	1.6185456
C	1.0231091	1.3528330	2.9907221
N	-0.3512382	2.3367139	1.2525165
C	-0.5510986	2.8722548	-0.0180393
H	-1.5528901	3.2814552	-0.1800171
O	0.3295567	2.8665771	-0.8435708
H	1.6944791	2.3998604	1.1600202
H	1.9524813	0.7944695	3.0886464
H	1.0789089	2.2186102	3.6569478
H	0.1731253	0.7338267	3.2769248
H	-1.1620302	2.1155928	1.8264480
O	1.0887124	0.3741984	0.6310959
C	2.3615372	-0.0942191	0.2801117
H	0.3523406	0.2331217	-0.0353763
C	2.3409956	-1.6308602	0.2838157
C	2.8367147	0.5000308	-1.0552782
H	3.0666979	0.2205562	1.0535271
O	-0.9910730	0.0850887	-1.0008866
F	1.7261175	-2.0584791	1.3811476
F	3.5734223	-2.1243175	0.2790738
F	1.6916560	-2.1209486	-0.7737213
F	3.9399039	-0.1102749	-1.4753357
F	3.1080675	1.7936831	-0.9135320
F	1.9092525	0.3804136	-2.0038257
C	-2.1909046	-0.6030618	-0.8066686
H	-0.8175512	0.2362294	-1.9441746
C	-2.0247872	-1.4553483	0.4507459
C	-3.3509125	0.3961118	-0.6866351
H	-2.4233959	-1.2780560	-1.6331750
F	-1.1495766	-2.4297919	0.2202763
F	-3.1785277	-2.0034054	0.8174070
F	-1.5617959	-0.7386410	1.4786885
F	-4.5271304	-0.2156284	-0.7787900
F	-3.2677358	1.2907921	-1.6694956
F	-3.3280270	1.0667231	0.4686089

vf_hfip2_ts_3.log
E = -1826.530507
H = -1826.281550
G = -1826.362241
NImag=1

C	-0.5416391	1.3988887	1.7291912
C	-1.1662160	0.9777666	3.0088097
N	-0.9872007	2.5667259	1.1714391
C	-0.4077011	3.0492637	0.0115821

H	0.6161521	2.6879433	-0.1625097
O	-0.9867931	3.8073157	-0.7293341
H	0.5356916	1.2751702	1.6659960
H	-0.9144079	-0.0567889	3.2343091
H	-0.7508909	1.6155854	3.7915464
H	-2.2480331	1.1179454	2.9931043
H	-1.9514610	2.8498988	1.3431535
O	-0.8780280	0.1486536	0.6465010
C	-2.1521198	-0.3704830	0.3512358
H	-0.1481501	0.0593888	-0.0581348
C	-2.7742838	0.3409219	-0.8646588
C	-2.0347029	-1.8923216	0.1470636
H	-2.8064076	-0.2121890	1.2106651
O	1.0668205	0.0479051	-1.0397244
F	-3.1943188	1.5574974	-0.5287524
F	-3.8103871	-0.3439432	-1.3298946
F	-1.8880332	0.4808231	-1.8495786
F	-3.2282019	-2.4658789	0.2271316
F	-1.2568556	-2.4099612	1.0887118
F	-1.5077747	-2.1925661	-1.0406279
C	2.3675265	-0.4412669	-0.8668644
H	0.8571835	0.1784450	-1.9787067
C	3.2769565	0.7231156	-0.4635573
C	2.3155533	-1.5693280	0.1648236
H	2.7687189	-0.8671354	-1.7880899
F	3.1998191	1.6732463	-1.3940099
F	4.5453617	0.3447858	-0.3578366
F	2.9083978	1.2707433	0.6963067
F	3.5223312	-2.0753609	0.3903782
F	1.5292309	-2.5410683	-0.2909271
F	1.8132698	-1.1625083	1.3329944

vf_hfip_h2o_prod_1.log
E = -1113.455696
H = -1113.255403
G = -1113.318611
NImag=0

C	1.7487792	1.3723827	-0.1520736
C	1.5686030	2.5793887	-1.0325236
N	2.6226621	0.4071926	-0.7864838
C	3.0505290	-0.6809041	-0.1598369
H	3.7102748	-1.3304776	-0.7366122
O	2.7539949	-0.9928687	1.0072891
H	2.1858817	1.6532219	0.8085097
H	0.9413364	3.3107487	-0.5275105
H	2.5425041	3.0305429	-1.2243897
H	1.1177911	2.3134492	-1.9909379
H	2.9027593	0.5499399	-1.7515633
O	0.5220704	0.7531960	0.2346983
C	-0.3354446	0.2345767	-0.7330609
H	0.6309242	0.5047634	2.4508311
C	-0.4753635	-1.2762065	-0.4817338
C	-1.6813330	0.9566002	-0.5945788
H	0.0100977	0.3703509	-1.7615205
O	1.2496284	-0.1979419	2.7226140
F	0.6072755	-1.9199676	-0.9233093
F	-1.5322094	-1.7873902	-1.1091001
F	-0.5956821	-1.5453774	0.8164533
F	-2.5163222	0.6480387	-1.5824805
F	-1.4908622	2.2736749	-0.6189707

F	-2.2768737	0.6565989	0.5616897
H	1.9373680	-0.4873209	1.9028843
H	1.7190842	0.0637653	3.5334650

vf_hfip_h2o_prod_2.log
E = -1113.444436
H = -1113.241407
G = -1113.304893
NImag=0

C	2.1362448	1.0159508	0.1376271
C	2.7584608	1.9843041	1.1048663
N	2.2205012	1.4964212	-1.2066224
C	1.9715719	0.6629125	-2.2384081
H	2.1003187	1.1113118	-3.2300407
O	1.6289890	-0.5009496	-2.0768154
H	2.5972586	0.0283353	0.1949802
H	2.6470866	1.6148196	2.1236292
H	3.8201092	2.0926408	0.8843763
H	2.2816512	2.9642179	1.0223378
H	2.4781209	2.4605195	-1.3807659
O	0.7419952	0.8486723	0.5130537
C	0.2083063	-0.4450760	0.5458524
H	0.0077278	2.0671500	1.1060727
C	0.1710995	-0.9113321	2.0037465
C	-1.1728242	-0.4201815	-0.1200908
H	0.8226907	-1.1493762	-0.0234094
O	-0.5529457	2.7986742	1.5536844
F	1.4230344	-1.0087237	2.4566175
F	-0.4132134	-2.0968815	2.1329445
F	-0.4658273	-0.0443483	2.7925094
F	-1.6328061	-1.6550685	-0.2956704
F	-1.0923780	0.1699003	-1.3084488
F	-2.0751691	0.2535927	0.5989609
H	-0.2099322	3.0514320	2.4321117
H	-0.6659784	3.5854857	0.9872457

vf_hfip_h2o_prod_3.log
E = -1113.444491
H = -1113.241135
G = -1113.304858
NImag=0

C	0.9397212	-0.5230838	2.0254811
C	0.2158844	-0.4939730	3.3428471
N	2.2598600	0.0143871	2.1386057
C	3.1462226	-0.1682726	1.1375306
H	4.1435561	0.2441627	1.3276897
O	2.8555746	-0.7490918	0.0998474
H	0.9989394	-1.5328754	1.6153055
H	-0.7919580	-0.8882996	3.2180641
H	0.7475417	-1.1107853	4.0670660
H	0.1564261	0.5284760	3.7238169
H	2.5270289	0.5236612	2.9725259
O	0.1720362	0.2655023	1.0749077
C	-0.0682323	-0.2670021	-0.1943883
H	-0.4529279	1.5726813	1.5767629
C	-1.5025696	-0.8019066	-0.2252770
C	0.1810121	0.8214057	-1.2471488
H	0.6126169	-1.0922822	-0.4198343
O	-0.8663671	2.4173889	1.9851361

F	-1.5923865	-1.8645774	0.5759682
F	-1.8685632	-1.1682990	-1.4491847
F	-2.3778258	0.1010133	0.2196014
F	0.2448314	0.2925212	-2.4646547
F	1.3269038	1.4467832	-1.0007399
F	-0.7841964	1.7476393	-1.2568363
H	-0.5799556	3.2338365	1.5331738
H	-1.8403637	2.3780829	2.0401475

vf_hfip_h2o_react_2.log
E = -1113.427735
H = -1113.227526
G = -1113.298800
NImag=0

C	2.8606655	1.1453290	0.0070178
C	3.3718815	1.9543691	1.1012460
N	2.6253815	1.6104050	-1.1791458
C	2.0256258	0.7851330	-2.1909400
H	1.9317989	1.2852512	-3.1577223
O	1.6684035	-0.3244364	-1.9368672
H	2.7128573	0.0775078	0.1360464
H	2.7119712	1.8087050	1.9622201
H	4.3409832	1.5382416	1.3969663
H	3.4634366	3.0080283	0.8502305
H	2.7835195	2.5972576	-1.3906003
O	0.3537267	0.8239309	0.5138943
C	-0.1882496	-0.4487502	0.6057000
H	-0.2098158	1.5259710	0.9460335
C	-0.3161703	-0.8761498	2.0697236
C	-1.5034678	-0.5208810	-0.1745149
H	0.4928410	-1.1630354	0.1326811
O	-1.1350916	2.6813983	1.6815151
F	0.8757391	-0.7495565	2.6607298
F	-0.6968600	-2.1462443	2.1925763
F	-1.1792763	-0.1210766	2.7519297
F	-2.0490968	-1.7353720	-0.1331788
F	-1.2710251	-0.2191574	-1.4534862
F	-2.4073035	0.3502706	0.2800683
H	-0.7644941	3.1437304	2.4460426
H	-1.5526980	3.3565400	1.1288111

vf_hfip_h2o_react_4.log
E = -1113.431687
H = -1113.231588
G = -1113.301138
NImag=0

C	1.3739679	-0.7136735	2.5316311
C	0.2633567	-0.8236699	3.4650668
N	2.2223652	0.2650368	2.5408784
C	3.2278680	0.3857305	1.5185069
H	3.8380120	1.2864647	1.6222602
O	3.3290737	-0.4374567	0.6618301
H	1.5590641	-1.4941279	1.7990217
H	-0.6586056	-0.8966065	2.8766645
H	0.3570416	-1.7821986	3.9860501
H	0.2084374	0.0038879	4.1679744
H	2.1027978	1.0414360	3.1950649
O	0.4376985	0.3510718	0.3376472
C	-0.2344296	-0.0541113	-0.8021408

```
H       -0.0314857        1.0665000        0.8383618
C        0.8053715       -0.7057684       -1.7128365
C       -1.3673583       -1.0026860       -0.4014311
H       -0.6847008        0.7692190       -1.3661654
O       -0.4659679        1.9770621        2.2120562
F        1.7781511        0.1688284       -1.9695930
F        0.2722437       -1.0818720       -2.8748825
F        1.3693483       -1.7789164       -1.1569047
F       -2.0618350       -1.4458382       -1.4461065
F       -2.2102127       -0.3424342        0.4021152
F       -0.9249590       -2.0600047        0.2810386
H       -0.2761122        2.9229872        2.1310004
H       -1.3792845        1.9188928        2.5276836
```

```
vf_hfip_h2o_react_5.log
E =     -1113.433656
H =     -1113.233817
G =     -1113.305103
NImag=0
C        2.7086574        1.1948672       -0.3933031
C        2.7435126        2.4554963       -1.1164218
N        2.9724361        0.0486050       -0.9367798
C        2.8253704       -1.1767888       -0.2006072
H        3.1421166       -2.0587774       -0.7623308
O        2.3827569       -1.1693626        0.9075871
H        2.5128309        1.1788566        0.6753221
H        1.7972838        2.9738105       -0.9304086
H        3.5172220        3.0787675       -0.6540936
H        2.9285703        2.3394660       -2.1809451
H        3.2068672       -0.0107372       -1.9297560
O        0.1645826        0.8620115        0.0239169
C       -0.9584316        0.3378458       -0.5960371
H        0.1558495        0.7075452        1.0067188
C       -1.1099620        1.1065488       -1.9066842
C       -0.7891453       -1.1716669       -0.7999251
H       -1.8826816        0.4806145       -0.0264071
O        0.3889973        0.2239112        2.5748669
F       -1.3498343        2.3905804       -1.6386120
F       -2.1130556        0.6467146       -2.6512739
F        0.0036075        1.0578053       -2.6434846
F       -1.8627668       -1.7340430       -1.3493756
F       -0.5813845       -1.7544777        0.3821172
F        0.2636128       -1.4616322       -1.5750180
H        1.1233234       -0.3719578        2.7807622
H        0.2451544        0.7836567        3.3497486
```

```
vf_hfip_h2o_ts_1.log
E =     -1113.427613
H =     -1113.228589
G =     -1113.294505
NImag=1
C        2.0297335        1.4162491       -0.3226591
C        1.5838502        2.6087965       -1.0560833
N        2.5967760        0.4050247       -0.9720021
C        2.9979858       -0.7469164       -0.2755944
H        3.4889097       -1.4976725       -0.9007097
O        2.7831999       -0.8657080        0.9023094
H        2.2870921        1.4942385        0.7279415
H        0.8887014        3.1891244       -0.4549122
```

H	2.4757163	3.2199921	-1.2325288
H	1.1480836	2.3508584	-2.0213498
H	2.5642554	0.3680887	-1.9894753
O	0.2943814	0.7305861	0.3205646
C	-0.7394234	0.2125722	-0.4469523
H	0.3776205	0.3667316	1.2798609
C	-0.5885291	-1.3075995	-0.6043667
C	-2.0835342	0.6139078	0.1760909
H	-0.7081301	0.6518736	-1.4482617
O	0.5655223	-0.1420133	2.6804466
F	0.4551390	-1.5738924	-1.3971315
F	-1.6664740	-1.8604106	-1.1513820
F	-0.3582150	-1.9057042	0.5620822
F	-3.0995870	0.3726044	-0.6463891
F	-2.0710155	1.9173252	0.4447707
F	-2.3141405	-0.0347788	1.3198806
H	1.4428643	-0.5229632	2.8387122
H	0.3778474	0.4659175	3.4098544

vf_hfip_h2o_ts_2.log
E = -1113.424586
H = -1113.225452
G = -1113.290968
NImag=1

C	-2.3617154	-0.5968327	0.2899936
C	-2.4449849	-2.0668348	0.3036906
N	-2.6472827	0.0768222	-0.8237103
C	-2.7272985	1.4789368	-0.8061431
H	-3.0481070	1.9170825	-1.7556782
O	-2.4437913	2.1048252	0.1811306
H	-2.5261024	-0.0427912	1.2065682
H	-1.9118176	-2.4707444	1.1625395
H	-3.5054129	-2.3156596	0.4180264
H	-2.0778119	-2.5017078	-0.6249171
H	-2.7234074	-0.4114674	-1.7142649
O	-0.4632038	-0.3619017	0.5465353
C	0.4176377	0.2460072	-0.3354119
H	-0.0474436	-0.9276128	1.3013962
C	1.2446850	1.2984986	0.4135695
C	1.2509934	-0.8221634	-1.0513937
H	-0.1367115	0.7796858	-1.1145572
O	0.5317090	-1.7385610	2.4096513
F	0.4213496	2.1560761	1.0113397
F	2.0288339	1.9838342	-0.4137781
F	2.0127232	0.7519411	1.3568059
F	2.1056554	-0.2925554	-1.9195073
F	0.4259105	-1.6308081	-1.7197235
F	1.9397042	-1.5757656	-0.1947098
H	0.2312794	-1.5552368	3.3112707
H	0.6458775	-2.6961362	2.3299157

vf_hfip_h2o_ts_3.log
E = -1113.426020
H = -1113.226598
G = -1113.292500
NImag=1

C	2.2998522	-0.6924928	-0.4857194
C	2.4475812	-2.0959648	-0.0740794
N	3.0769258	0.2444350	0.0394011

```
C        2.9229650        1.5863496       -0.3500007
H        3.6569138        2.2658811        0.0914163
O        2.0348389        1.9170460       -1.0899584
H        1.8730848       -0.4518699       -1.4534732
H        1.5639300       -2.6708387       -0.3410708
H        3.2955780       -2.4975106       -0.6400999
H        2.6645166       -2.1833116        0.9902630
H        3.6704366        0.0295903        0.8387151
O        0.5298004       -0.2544620        0.3262080
C       -0.5492059        0.2486933       -0.3951827
H        0.2978296       -0.8068478        1.1527309
C       -1.4625237       -0.8906330       -0.8540474
C       -1.2516963        1.3211868        0.4441715
H       -0.1784320        0.7473849       -1.2960069
O        0.0125542       -1.6380219        2.3997954
F       -0.7670012       -1.7106324       -1.6457743
F       -2.5097724       -0.4467260       -1.5409703
F       -1.9141940       -1.6157470        0.1701715
F       -2.2303958        1.9167323       -0.2306189
F       -0.3614561        2.2507936        0.7893259
F       -1.7691679        0.8215959        1.5684256
H       -0.0447812       -2.6030604        2.3621112
H        0.4415832       -1.4051339        3.2351060
```

```
vf_hfip_prod_1.log
E =     -1036.628481
H =     -1036.466262
G =     -1036.525389
NImag=0
C        1.1209451       -0.2825861        1.2719681
C        0.5232378       -0.8748193        2.5197920
N        1.9151018       -1.2615593        0.5758202
C        2.7764279       -0.8882627       -0.3872018
H        3.3304750       -1.7154756       -0.8467129
O        2.9392370        0.2793822       -0.7257664
O        0.0373677        0.1517337        0.4476905
C        0.1487592        1.3798794       -0.2020890
C       -0.3816019        1.1668509       -1.6194914
F        0.3858555        0.2835986       -2.2570760
F       -0.3823894        2.2957821       -2.3270191
F       -1.6252218        0.6833780       -1.6169874
C       -0.6449961        2.4149797        0.5973493
F       -0.6116208        3.6238765        0.0399806
F       -0.1134656        2.5149224        1.8201073
F       -1.9219552        2.0648805        0.7495768
H        1.7577147        0.5773728        1.4933605
H       -0.0664632       -0.1196608        3.0380763
H        1.3081955       -1.2319734        3.1864467
H       -0.1298982       -1.7090891        2.2543426
H        1.7837387       -2.2453387        0.7788968
H        1.1805562        1.7364280       -0.2858638
```

```
vf_hfip_prod_2.log
E =     -1036.616090
H =     -1036.453895
G =     -1036.512700
NImag=0
C        1.1824133       -0.0020468        1.0004380
C        1.4066541       -0.4036056        2.4368057
```

N	1.7962989	-0.9206477	0.0730615
C	2.8127873	-0.5715736	-0.7367773
H	3.1469977	-1.3837003	-1.3949025
O	3.3380216	0.5363011	-0.7505925
O	-0.2336210	-0.0284647	0.8033254
C	-0.9069065	0.9402063	0.0620101
C	-0.8612252	2.3228963	0.7297477
F	-1.1203267	2.1883111	2.0293151
F	-1.7830389	3.1312666	0.2082333
F	0.3185895	2.9378274	0.6179807
C	-0.5019332	0.9652299	-1.4161979
F	-0.5995310	-0.2646050	-1.9201298
F	0.7473454	1.3848649	-1.6146415
F	-1.3109112	1.7602419	-2.1175009
H	1.6047606	0.9817428	0.7958715
H	0.9392453	0.3218808	3.1031967
H	2.4750180	-0.4461356	2.6496027
H	0.9652625	-1.3864698	2.6161520
H	1.4546719	-1.8749346	0.0536675
H	-1.9616725	0.6531144	0.0696345

vf_hfip_prod_4.log
E = -1036.619696
H = -1036.457637
G = -1036.517328
NImag=0

C	1.5898197	0.2012356	0.6731507
C	1.6526418	0.4273805	2.1634797
N	1.9468118	-1.1485072	0.2961475
C	3.2313883	-1.5250492	0.2801736
H	3.3937951	-2.5751455	0.0103118
O	4.1576023	-0.7580335	0.5387230
O	0.2625095	0.3517865	0.1851467
C	-0.1739095	1.6549772	-0.0422329
C	-0.2252520	1.8719041	-1.5570682
F	-1.0457625	1.0092939	-2.1555682
F	0.9948953	1.6791282	-2.0621980
F	-0.6131062	3.1033363	-1.8804044
C	-1.5328992	1.7930512	0.6441643
F	-1.3767604	1.6612530	1.9615436
F	-2.3855093	0.8496732	0.2469936
F	-2.0912597	2.9796275	0.4133042
H	2.2624417	0.8878922	0.1495088
H	1.4031349	1.4621267	2.4056774
H	2.6666929	0.2281916	2.5147828
H	0.9539117	-0.2415046	2.6691377
H	1.2019844	-1.8196685	0.1484508
H	0.4859296	2.4195507	0.3810754

vf_hfip_prod_5.log
E = -1036.617037
H = -1036.454997
G = -1036.514103
NImag=0

C	1.5215471	0.1322610	0.7243328
C	0.9050546	-0.2365955	2.0517288
N	2.1904693	-0.9915889	0.1027244
C	3.3960381	-1.3831814	0.5323455
H	3.8053236	-2.2536876	0.0061817

O	4.0014129	-0.8154255	1.4404628
O	0.5727070	0.5121313	-0.2670257
C	-0.5148900	1.3113000	0.0673109
C	-0.1398870	2.7835355	-0.1190425
F	-1.1538473	3.6023265	0.1509466
F	0.2834536	3.0390519	-1.3551201
F	0.8589670	3.0820599	0.7172512
C	-1.6456284	0.8458631	-0.8498895
F	-1.3000432	0.9006599	-2.1351689
F	-2.7508182	1.5691793	-0.6874047
F	-1.9398292	-0.4237315	-0.5673844
H	2.2619676	0.9256241	0.8568540
H	0.5472113	0.6417072	2.5904127
H	1.6798223	-0.7038615	2.6618384
H	0.0857991	-0.9457126	1.9094425
H	1.6888223	-1.5142197	-0.6060384
H	-0.8676525	1.1896044	1.0953418

vf_hfip_prod_6.log
E = -1036.615507
H = -1036.452678
G = -1036.510843
NImag=0

C	1.8203853	0.4660951	0.6997800
C	1.4054310	0.2588408	2.1366049
N	1.9221619	-0.7905895	-0.0258990
C	3.0045700	-1.5661567	0.1397138
H	2.9687750	-2.5155286	-0.4080193
O	3.9635846	-1.2443887	0.8377551
O	1.0070644	1.3768877	-0.0220436
C	-0.3780673	1.2029072	-0.0828679
C	-1.0269371	2.4070655	0.6108250
F	-0.6889681	2.4202048	1.8994211
F	-2.3561594	2.3719432	0.5419686
F	-0.6141922	3.5569258	0.0793757
C	-0.7514931	1.1116865	-1.5673977
F	-2.0669600	1.0317842	-1.7516795
F	-0.2021595	0.0153115	-2.0973873
F	-0.2978862	2.1553388	-2.2570060
H	2.8095828	0.9233372	0.6724493
H	1.4359567	1.2009831	2.6819181
H	2.1220684	-0.4280147	2.5917730
H	0.4106198	-0.1835444	2.2208300
H	1.1155393	-1.1672641	-0.5094787
H	-0.7459162	0.2943752	0.4038643

vf_hfip_prod_7.log
E = -1036.622303
H = -1036.459809
G = -1036.518010
NImag=0

C	0.7359889	-0.2995042	1.6115217
C	0.6263109	-1.6357948	0.9083231
N	2.0947162	0.1681511	1.7889737
C	3.0295585	0.2126870	0.8225482
H	3.9989400	0.5916418	1.1721477
O	2.8498155	-0.1282029	-0.3412415
O	-0.1049825	0.7075777	1.0432591
C	-0.0212357	1.0201212	-0.3167304

C	-1.3966925	0.7454295	-0.9316472
F	-1.4177312	0.9773439	-2.2434166
F	-2.3517090	1.4869987	-0.3673108
F	-1.7238486	-0.5328809	-0.7425965
C	0.3814063	2.4948128	-0.4213987
F	1.6346927	2.6553380	0.0071194
F	-0.3951909	3.2752050	0.3302910
F	0.3229918	2.9436033	-1.6746748
H	0.3294224	-0.4003345	2.6158150
H	-0.3884118	-2.0134865	1.0277624
H	1.3128638	-2.3387068	1.3817817
H	0.8704653	-1.5891635	-0.1507314
H	2.3374173	0.5730195	2.6844825
H	0.7215124	0.4375446	-0.8696777

vf_hfip_prod_8.log
E = -1036.609738
H = -1036.447602
G = -1036.506433
NImag=0

C	1.3767686	0.1771509	0.5374397
C	1.5221806	1.1221426	1.7063570
N	1.8347606	-1.1576872	0.8682454
C	3.1439555	-1.4293576	0.9208453
H	3.3826463	-2.4594629	1.2106473
O	4.0127231	-0.5978041	0.6614774
O	0.0261314	-0.0619204	0.1567935
C	-0.7856059	0.8507014	-0.5102377
C	-1.6098158	1.7368535	0.4459050
F	-1.9789877	1.0191410	1.5031909
F	-2.7151387	2.1611466	-0.1703812
F	-0.9706754	2.8179728	0.8936005
C	-0.0722573	1.6564855	-1.5990961
F	0.8319978	2.5161478	-1.1266987
F	-0.9559499	2.3557959	-2.3093459
F	0.5591430	0.8220694	-2.4233803
H	1.9713453	0.5203111	-0.3131827
H	1.3190411	2.1524791	1.4199106
H	2.5536409	1.0722805	2.0609003
H	0.8508681	0.8239131	2.5144040
H	1.1444720	-1.8425447	1.1534970
H	-1.5335434	0.2552857	-1.0421913

vf_iminium_e_1.log
E = -247.514114
H = -247.415914
G = -247.451290
NImag=0

C	-0.7964089	-0.2889011	0.0097984
C	-2.2342116	-0.0942371	-0.0018828
N	0.0491294	0.5496097	-0.4922189
C	1.4719537	0.2978287	-0.4490709
H	2.0610169	1.0930227	-0.9114960
O	1.8940239	-0.6957031	0.0543810
H	-0.3626305	-1.1801123	0.4578643
H	-2.5841507	-0.1431876	1.0350289
H	-2.5431251	0.8327910	-0.4771109
H	-2.6825489	-0.9619000	-0.4980601
H	-0.2731844	1.4141350	-0.9331878

vf_iminium_e_2.log
E = -247.513812
H = -247.416298
G = -247.453049
NImag=0

C	1.0138596	0.3936729	0.0076799
C	2.3245837	-0.2309304	-0.0010693
N	-0.0411233	-0.1499264	-0.5045715
C	-1.3268781	0.4821680	-0.4863540
H	-1.3297062	1.4627839	-0.0002856
O	-2.2639617	-0.0687767	-0.9751828
H	0.8808907	1.3723892	0.4628535
H	2.6684560	-0.3019580	1.0362906
H	2.3318266	-1.2050561	-0.4828723
H	3.0200120	0.4571914	-0.4932836
H	0.0009445	-1.0711428	-0.9521103

vf_iminium_z_1.log
E = -247.511608
H = -247.413468
G = -247.449025
NImag=0

C	1.1513763	-0.0129939	-0.1535280
C	1.2764835	-1.4619681	-0.0471558
N	0.0483910	0.6646063	-0.0470464
C	-1.2531291	0.1085370	0.1955061
H	-2.0320868	0.8737220	0.2462433
O	-1.4134271	-1.0670514	0.3205873
H	2.0326306	0.5942946	-0.3377227
H	0.6351655	-1.9436991	-0.7929419
H	2.3118263	-1.7595053	-0.1760615
H	0.8911293	-1.7958028	0.9221629
H	0.0920405	1.6809610	-0.1412433

vf_iminium_z_2.log
E = -247.509853
H = -247.411013
G = -247.447376
NImag=0

C	-1.1313278	0.5792127	0.0148190
C	-1.9249480	-0.6387286	-0.0085801
N	0.0431378	0.7494926	0.5350765
C	0.8471464	-0.2222397	1.2150556
H	0.4201318	-1.2213306	1.3047840
O	1.9132454	0.1169887	1.6323404
H	-1.5500764	1.4651956	-0.4523582
H	-2.1556283	-0.8429201	-1.0600122
H	-1.4866537	-1.5101415	0.4637326
H	-2.8894896	-0.3937328	0.4497376
H	0.4835453	1.6724955	0.4745259

vf_mes_add_1.log
E = -597.399220
H = -597.104616
G = -597.162190
NImag=0

C	-2.2230145	-2.4879465	-0.5255542
C	-1.5509212	-3.7958612	-0.1729517
N	-3.6571765	-2.6273789	-0.5254104
C	-4.4391088	-1.5416706	-0.6436299
H	-5.5127931	-1.7520095	-0.7097258
O	-3.9845758	-0.3996918	-0.6697938
C	-1.6828281	-1.9041617	-1.9078545
C	-0.3694726	-1.2828340	-1.6486264
C	0.7496174	-1.7449630	-2.2787605
C	0.6634822	-2.7917617	-3.2133068
C	-0.5872542	-3.3407603	-3.5711386
C	-1.7389082	-2.9134243	-2.9833363
H	-2.3903409	-1.0894563	-2.1321536
C	1.8852804	-3.3136886	-3.8681243
C	-3.0573656	-3.4666636	-3.3900724
C	-0.3037055	-0.1772894	-0.6590655
H	-1.9799355	-1.7247816	0.2147239
H	-0.4665617	-3.6974947	-0.2580113
H	-1.7914644	-4.0754392	0.8532820
H	-1.8781552	-4.6012845	-0.8354310
H	-4.0700104	-3.5481610	-0.6197482
H	1.7160895	-1.2993142	-2.0731939
H	-0.6199230	-4.0974693	-4.3475201
H	2.7840943	-2.7931060	-3.5454644
H	1.9839934	-4.3813024	-3.6495553
H	1.7828270	-3.2331621	-4.9539548
H	-2.9913012	-3.9347949	-4.3717260
H	-3.3731448	-4.2376298	-2.6783678
H	-3.8298589	-2.6959687	-3.3991323
H	0.4874125	0.5255112	-0.9210471
H	-1.2584978	0.3453781	-0.5752070
H	-0.0565789	-0.5846195	0.3283581

vf_mes_add_1_ts.log
E = -597.386821
H = -597.093802
G = -597.152017
NImag=1

C	-1.1787518	-0.0147884	1.0521877
C	-0.2396703	-0.5696026	2.0502745
N	-2.2669765	-0.7687127	0.7380404
C	-3.2935186	-0.2520951	-0.0270214
H	-4.1087225	-0.9564695	-0.2182625
O	-3.2654113	0.8861287	-0.4424603
C	-0.1616861	0.2513801	-0.7426376
C	0.6121417	1.3999300	-0.4120386
C	1.9157971	1.2217709	-0.0109306
C	2.5014660	-0.0561992	-0.0032264
C	1.7588821	-1.1642160	-0.4158746
C	0.4411604	-1.0282423	-0.8160387
H	-1.0925232	0.4209689	-1.2792288
C	3.9100472	-0.2170609	0.4639389
C	-0.3691352	-2.2063444	-1.2405779
C	-0.0201422	2.7486477	-0.5067201
H	-1.3975162	1.0464918	1.0904655
H	0.7180580	-0.0520420	2.0293035
H	-0.6868682	-0.3912413	3.0339756
H	-0.0956352	-1.6430747	1.9224833
H	-2.2606977	-1.7668173	0.9351450
H	2.5130269	2.0783478	0.2850877

H	2.2271649	-2.1430071	-0.4291268
H	4.5451192	0.5784806	0.0705326
H	3.9466703	-0.1452885	1.5552138
H	4.3214231	-1.1834694	0.1750656
H	0.1927146	-2.8269109	-1.9409940
H	-0.5997416	-2.8397318	-0.3763857
H	-1.3055926	-1.9060081	-1.7124397
H	0.1300035	3.1500340	-1.5135933
H	-1.1008436	2.6966952	-0.3485366
H	0.4196588	3.4520485	0.2002784

vf_mes_add_2.log
E = -597.397895
H = -597.103444
G = -597.160768
NImag=0

C	-2.5008576	-2.7638303	-0.3181998
C	-2.7975590	-3.9715182	0.5466811
N	-3.6344020	-2.4663553	-1.1729152
C	-3.6160816	-1.3684182	-1.9409931
H	-4.4618078	-1.2659273	-2.6306760
O	-2.7165644	-0.5331353	-1.8655227
C	-1.1618095	-2.8879673	-1.1456249
C	-1.3081631	-3.5774218	-2.4540972
C	-0.7601363	-3.0353466	-3.5712897
C	0.0104312	-1.8456859	-3.5240992
C	0.2183225	-1.1823917	-2.3157267
C	-0.3802361	-1.6258905	-1.1639321
H	-0.5345111	-3.5631033	-0.5327897
C	0.6023507	-1.3349451	-4.7820887
C	-0.2072237	-0.8722032	0.1011225
C	-2.0007874	-4.8930917	-2.5187706
H	-2.3631835	-1.9041705	0.3379368
H	-1.9498451	-4.1731461	1.2023346
H	-3.6705770	-3.7683025	1.1688416
H	-3.0002596	-4.8669392	-0.0408339
H	-4.3341960	-3.1802168	-1.3394354
H	-0.8622148	-3.5443508	-4.5238055
H	0.8237322	-0.2837793	-2.2965817
H	-0.1984151	-1.1262019	-5.4979675
H	1.1940568	-0.4357221	-4.6267705
H	1.2248753	-2.1102413	-5.2376316
H	0.7091604	-0.2835961	0.0773974
H	-1.0463918	-0.1746158	0.2076327
H	-0.2057373	-1.5269900	0.9733380
H	-1.8819799	-5.3393442	-3.5050648
H	-1.5960179	-5.5759895	-1.7686247
H	-3.0712720	-4.7954623	-2.3182434

vf_mes_add_2_ts.log
E = -597.392048
H = -597.098888
G = -597.155962
NImag=1

C	-0.6912466	-1.4772850	0.5212311
C	-2.0995904	-1.7264934	0.9271198
N	0.1403175	-0.9547615	1.4565473
C	1.5115135	-1.1367564	1.4021397
H	2.0524740	-0.6364276	2.2124189

O	2.0411659	-1.8113494	0.5480248
C	-0.8518576	-0.1839830	-1.0787171
C	-1.4646802	0.9007569	-0.3904146
C	-0.6459995	1.8200760	0.2314324
C	0.7446536	1.7688882	0.0706763
C	1.3133815	0.8189183	-0.7907848
C	0.5379744	-0.1447746	-1.3938437
H	-1.4866792	-0.8435499	-1.6651489
C	1.6286615	2.7338197	0.7889390
C	1.1196169	-1.1737542	-2.3035849
C	-2.9508031	1.0331721	-0.3429978
H	-0.2180555	-2.2376961	-0.0908984
H	-2.7315246	-1.9439673	0.0694310
H	-2.0798137	-2.6157547	1.5657576
H	-2.5150369	-0.9009639	1.5049457
H	-0.2386941	-0.3644812	2.1931625
H	-1.0813560	2.6146012	0.8285570
H	2.3865009	0.8252282	-0.9526924
H	2.1557801	2.2191878	1.5982449
H	2.3904505	3.1354184	0.1184610
H	1.0602107	3.5554315	1.2229776
H	0.8622192	-0.9413513	-3.3409056
H	2.2047789	-1.2132245	-2.2129061
H	0.7139190	-2.1662758	-2.0907398
H	-3.2525877	1.8376012	-1.0206457
H	-3.4555497	0.1260144	-0.6720549
H	-3.3006293	1.3052581	0.6543262

vf_mes_add_2_ts_z.log
E = -597.389967
H = -597.096921
G = -597.153684
NImag=1

C	0.6870984	-1.8848659	-2.0170620
C	1.9608392	-1.3966258	-2.6173149
N	-0.0726370	-0.9622548	-1.3677453
C	-1.4307605	-1.1039121	-1.1732460
H	-1.8721225	-1.9466382	-1.7222718
O	-2.0717774	-0.3311753	-0.4943351
C	1.2547759	-3.3532031	-0.7572064
C	1.9468671	-2.5455216	0.1894686
C	1.2156152	-1.9644370	1.2064395
C	-0.1363157	-2.2753365	1.3821248
C	-0.7405855	-3.2624541	0.5783837
C	-0.0560309	-3.8386751	-0.4624757
H	1.8459228	-3.9071936	-1.4817942
C	-0.9589707	-1.5630219	2.3953905
C	-0.6714851	-4.8714369	-1.3423631
C	3.4144462	-2.3164123	0.0602455
H	0.1035011	-2.5587327	-2.6376671
H	2.6058232	-2.2194560	-2.9163347
H	1.6859054	-0.8359584	-3.5159620
H	2.4970029	-0.7237405	-1.9481208
H	0.3780918	-0.1833319	-0.8875294
H	1.6930008	-1.2687513	1.8885252
H	-1.7682238	-3.5481555	0.7785161
H	-1.5911045	-0.8300842	1.8764038
H	-1.6298765	-2.2507594	2.9126653
H	-0.3438991	-1.0311157	3.1200286
H	-0.1630409	-5.8294395	-1.2038048

H	-1.7296767	-5.0044854	-1.1204660
H	-0.5611001	-4.6131848	-2.3999012
H	3.9276044	-2.9142623	0.8200742
H	3.7949260	-2.6306557	-0.9106940
H	3.6788906	-1.2732476	0.2407712

vf_mes_add_3.log
E = -597.395546
H = -597.101168
G = -597.160276
NImag=0

C	-2.1833770	-2.3900560	-0.2118220
C	-3.3059098	-3.3382231	0.1666921
N	-2.6377052	-1.1771955	-0.8531522
C	-3.4464870	-1.1291647	-1.9296667
H	-3.6501634	-0.1063001	-2.2725023
O	-3.9248455	-2.1111154	-2.4840148
C	-0.9812699	-3.0858040	-1.0271489
C	-1.4559407	-3.5673032	-2.3369435
C	-1.1200229	-2.8731082	-3.4695166
C	-0.2190729	-1.8036087	-3.4149131
C	0.4330200	-1.4626912	-2.2031889
C	0.1133604	-2.0888184	-1.0432644
H	-0.7215898	-3.9359737	-0.3859935
C	0.1183018	-1.0419361	-4.6401014
C	0.7493907	-1.7148177	0.2478942
C	-2.2748140	-4.8038409	-2.4228602
H	-1.7033526	-2.0804801	0.7154105
H	-2.8921640	-4.2058315	0.6821271
H	-3.9664382	-2.8145360	0.8604161
H	-3.9015866	-3.6706677	-0.6781339
H	-2.2206776	-0.3029317	-0.5556666
H	-1.5240583	-3.1743090	-4.4296073
H	1.1953299	-0.6913833	-2.2212252
H	-0.4089842	-1.4124955	-5.5161717
H	-0.1267997	0.0135359	-4.4873816
H	1.1973989	-1.0825025	-4.8131848
H	1.7490871	-1.3142436	0.0810062
H	0.1649027	-0.9293772	0.7414407
H	0.8028990	-2.5623939	0.9320707
H	-1.8392749	-5.4644403	-3.1770507
H	-2.3245589	-5.3373722	-1.4761993
H	-3.2865975	-4.5614144	-2.7585481

vf_mes_add_3_ts.log
E = -597.387790
H = -597.094618
G = -597.152327
NImag=1

C	-1.1197048	0.9663450	-0.9092505
C	-2.5127932	1.1167516	-0.3994077
N	-0.7223192	-0.1452837	-1.5876739
C	-1.1647856	-1.4382721	-1.3672275
H	-0.6800715	-2.1668695	-2.0281283
O	-2.0024113	-1.7323741	-0.5457074
C	0.0652702	1.2038883	0.7713266
C	-0.2385877	-0.0335175	1.3949356
C	0.5389215	-1.1329977	1.0747921
C	1.6416023	-1.0110855	0.2301944

C	2.0350055	0.2607220	-0.2315510
C	1.2957088	1.3774718	0.0651846
H	-0.4484390	2.0949110	1.1224315
C	2.4225324	-2.2157647	-0.1780315
C	1.7048133	2.7455261	-0.3655450
C	-1.3798787	-0.1521768	2.3490312
H	-0.7174134	1.8690806	-1.3533144
H	-2.6101322	2.0630081	0.1292272
H	-3.1494006	1.1617088	-1.2907503
H	-2.8634676	0.2966121	0.2158325
H	0.0748981	-0.0517745	-2.2112960
H	0.2774517	-2.1083327	1.4722118
H	2.9396480	0.3495755	-0.8241498
H	2.1353072	-3.0948990	0.3972220
H	2.2505232	-2.4276816	-1.2376705
H	3.4938913	-2.0416178	-0.0599756
H	2.5530049	2.7126367	-1.0482706
H	0.8816621	3.2773715	-0.8510944
H	1.9857058	3.3402927	0.5078501
H	-0.9880133	-0.3347758	3.3537779
H	-1.9752122	0.7596348	2.3866268
H	-2.0218159	-0.9981414	2.0923068

vf_mes_add_4.log
E = -597.388748
H = -597.093715
G = -597.151870
NImag=0

C	-1.9998483	-2.5932238	-0.4615690
C	-2.5964718	-3.9927387	-0.4894703
N	-2.8386815	-1.6880744	0.2967033
C	-4.0374074	-1.2936807	-0.1642811
H	-4.5628860	-0.5857598	0.4881568
O	-4.5064534	-1.6829547	-1.2309881
C	-1.6329236	-1.9303651	-1.8873519
C	-0.3299551	-1.2577859	-1.6708893
C	0.7926103	-1.7158192	-2.2948536
C	0.7187553	-2.7770970	-3.2164070
C	-0.5286064	-3.3223853	-3.5905427
C	-1.6850327	-2.8885153	-3.0173843
H	-2.4112239	-1.1783590	-2.0582336
C	1.9468145	-3.2908189	-3.8629984
C	-3.0065592	-3.2861439	-3.5641401
C	-0.2675948	-0.1389433	-0.6968280
H	-1.0590028	-2.6678547	0.0814600
H	-2.0295857	-4.6553832	-1.1450795
H	-2.5363009	-4.3921421	0.5243392
H	-3.6436143	-3.9925906	-0.7850945
H	-2.4690323	-1.2453725	1.1285914
H	1.7549474	-1.2599078	-2.0933477
H	-0.5564961	-4.0523046	-4.3920876
H	2.8452790	-2.7980984	-3.4986218
H	2.0229695	-4.3693775	-3.6979972
H	1.8690775	-3.1543160	-4.9459588
H	-2.8935318	-3.9848832	-4.3917432
H	-3.6662621	-3.7166243	-2.8121022
H	-3.5170417	-2.3848511	-3.9204729
H	0.6682905	0.4091495	-0.7966739
H	-1.1107425	0.5417955	-0.8231223
H	-0.3116897	-0.5254740	0.3279885

vf_mes_add_4_ts.log
E = -597.377110
H = -597.083867
G = -597.141095
NImag=1

C	-1.0868734	0.6008145	1.0029723
C	-0.7378041	-0.5518694	1.8762475
N	-2.3856555	0.8501795	0.6333696
C	-3.2451464	-0.0996074	0.1306625
H	-4.2465705	0.2876255	-0.0865559
O	-2.9052899	-1.2445879	-0.0691813
C	-0.1370769	0.2090263	-0.8009417
C	0.7105809	1.3290352	-0.5588483
C	1.9676695	1.1110174	-0.0174567
C	2.4570339	-0.1828438	0.1423227
C	1.6776016	-1.2810255	-0.2699783
C	0.4083041	-1.1139256	-0.7659960
H	-1.0216933	0.3631865	-1.4109559
C	3.8097242	-0.4205352	0.7238361
C	-0.3789397	-2.2879626	-1.2495437
C	0.2292916	2.7041016	-0.8728051
H	-0.6030580	1.5213259	1.3057984
H	0.3428020	-0.6088888	2.0006468
H	-1.1648254	-0.3115462	2.8567544
H	-1.1400977	-1.5026953	1.5404805
H	-2.6748600	1.8204499	0.5487449
H	2.5945705	1.9573717	0.2414273
H	2.1045912	-2.2771543	-0.2093331
H	4.3900043	0.4992136	0.7845078
H	3.7099760	-0.8301546	1.7334418
H	4.3595884	-1.1576735	0.1356831
H	0.2813575	-3.0018695	-1.7442819
H	-0.8562549	-2.8058795	-0.4127979
H	-1.1693622	-1.9883252	-1.9378106
H	0.6873693	3.4498656	-0.2231511
H	0.5023614	2.9463248	-1.9042604
H	-0.8583777	2.7816136	-0.8104335

vf_tfo_hfip_ts_1.log
E = -2787.878057
H = -2787.592681
G = -2787.696429
NImag=1

C	1.1949667	-1.4172609	1.9595264
C	2.1236477	-1.1470317	3.0731154
N	0.9757856	-2.6883178	1.5866302
C	0.0611319	-2.9655314	0.5660828
H	-0.0587334	-4.0327948	0.3553334
O	-0.5157137	-2.0824826	-0.0140808
H	0.3515100	-0.7485514	1.8150959
H	2.3409850	-0.0840655	3.1415200
H	1.6105429	-1.4534361	3.9897932
H	3.0426450	-1.7280679	2.9849653
H	1.5939169	-3.4280653	1.9113357
O	2.0608591	-0.6019101	0.5322656
C	3.3079441	-0.9610314	0.0247796
H	1.4494452	-0.0797858	-0.0984335
C	3.1604310	-1.6298359	-1.3482472

C	4.1984924	0.2882879	0.0162910
H	3.7780390	-1.6922270	0.6894208
O	0.5400440	0.8223980	-0.9436253
F	2.3566954	-2.6887686	-1.2367131
F	4.3331404	-2.0502920	-1.8097454
F	2.6236182	-0.8131038	-2.2506569
F	5.4152217	0.0252100	-0.4483425
F	4.3194172	0.7433731	1.2621520
F	3.6704527	1.2640500	-0.7204033
C	0.6399436	2.9386831	0.5193870
F	-0.1048560	3.8152380	1.1813718
F	1.3435773	3.5827278	-0.4028245
F	1.4831636	2.3648771	1.3764623
O	-1.3014979	2.4006464	-1.1119345
S	-0.4212878	1.6710442	-0.2549251
O	-1.0365823	1.0261725	0.8836306
H	-2.8734005	1.1492326	0.7647136
O	-3.8118144	1.3106705	0.5271720
C	-4.4107438	0.2011521	-0.0693268
C	-3.8237930	-0.0636040	-1.4605909
C	-4.3405319	-1.0051563	0.8682494
H	-5.4703482	0.4200832	-0.2207858
F	-3.8954543	1.0528814	-2.1842273
F	-4.5037843	-1.0122289	-2.1081793
F	-2.5437454	-0.4397444	-1.4235919
F	-4.9460301	-2.0786390	0.3612656
F	-4.9457478	-0.6999755	2.0175704
F	-3.0830567	-1.3457270	1.1623022

vf_tfo_hfip_ts_10.log
E = -2787.879449
H = -2787.594681
G = -2787.701738
NImag=1

C	0.6629673	-2.5144163	0.2608272
C	-0.1058112	-2.4104531	-0.9996950
N	0.0821202	-2.0894886	1.3963026
C	0.6502818	-2.3602728	2.6355703
H	0.0447292	-2.0226050	3.4825011
O	1.7175417	-2.9155914	2.7432934
H	1.3429774	-3.3509850	0.3838425
H	-0.7102954	-1.5030689	-1.0425866
H	0.5535404	-2.4747606	-1.8625272
H	-0.7784386	-3.2733318	-1.0194276
H	-0.7277355	-1.4626274	1.3431209
O	2.0476975	-1.3596137	-0.1109810
C	3.3237813	-1.3728784	0.4554292
H	1.8394434	-0.5974424	-0.7588384
C	4.3501352	-1.5156322	-0.6734774
C	3.5233478	-0.1276931	1.3299115
H	3.4214796	-2.2461384	1.1053216
O	1.3317432	0.5190226	-1.6759350
F	5.5970549	-1.5052536	-0.2166717
F	4.2276297	-0.5444081	-1.5782416
F	4.1429762	-2.6735858	-1.2993563
F	4.6752084	-0.1859946	1.9911704
F	2.5369379	-0.0472819	2.2219766
F	3.5107475	0.9962700	0.6143593
C	0.8926169	3.0377319	-1.3902125
F	0.0492590	3.9190735	-0.8631113

F	0.9673812	3.2484142	-2.6991262
F	2.0935951	3.2268473	-0.8581054
O	-0.9759785	1.2866961	-1.7073599
S	0.2988811	1.3454414	-1.0577727
O	0.2903375	1.2437760	0.3791537
H	-1.7857240	0.7473290	1.0392203
O	-2.1311931	-0.1423941	0.8575871
C	-3.5175081	-0.2146428	0.9179097
C	-4.0961399	0.0957229	-0.4681980
C	-3.8406180	-1.6253380	1.4078532
H	-3.9655282	0.4862925	1.6271697
F	-3.8664588	1.3765700	-0.7602436
F	-5.4132383	-0.1014168	-0.5234002
F	-3.5288725	-0.6427740	-1.4214882
F	-5.1482015	-1.8369896	1.5206045
F	-3.2925401	-1.8088859	2.6108293
F	-3.3387497	-2.5581611	0.5947509

vf_tfo_hfip_ts_10_prod.log
E = -2787.893348
H = -2787.606121
G = -2787.711737
NImag=0

C	-0.5562909	-1.9838456	0.9153931
C	0.3170479	-1.3805971	1.9855732
N	0.2107725	-2.3039462	-0.2495007
C	-0.2155894	-3.2102131	-1.1515430
H	0.4722919	-3.3609588	-1.9924309
O	-1.2709966	-3.8246006	-1.0489711
H	-1.0615349	-2.8868336	1.2635885
H	0.9160181	-0.5539725	1.5901785
H	-0.2987401	-1.0159059	2.8075325
H	0.9968544	-2.1426665	2.3649196
H	1.0424573	-1.7509512	-0.4519858
O	-1.5911672	-1.0130162	0.6085025
C	-2.8261759	-1.4722642	0.1421047
H	-1.3398350	0.5237803	1.0061448
C	-3.8680792	-1.1404789	1.2139545
C	-3.1094087	-0.8178300	-1.2137253
H	-2.8328333	-2.5555269	-0.0118744
O	-0.9970770	1.4609138	1.1783721
F	-5.0982569	-1.4773579	0.8414266
F	-3.8698690	0.1574670	1.5246621
F	-3.5700203	-1.8132113	2.3265790
F	-4.2405882	-1.2758490	-1.7431266
F	-2.1176750	-1.0876828	-2.0601392
F	-3.2067629	0.5104065	-1.1213113
C	-0.9232780	3.5219329	-0.3355373
F	-0.2293207	4.1419297	-1.2757758
F	-0.9966463	4.2873472	0.7394309
F	-2.1393896	3.2648335	-0.7802321
O	1.1858144	2.3525113	0.6350912
S	-0.0598660	1.9660093	0.0824370
O	-0.1243661	1.1236007	-1.0612147
H	2.1992270	0.2042760	-1.5885581
O	2.4676446	-0.2243789	-0.7617968
C	3.8515112	-0.3089220	-0.6506944
C	4.3311255	0.7939123	0.2979394
C	4.1673521	-1.7188019	-0.1522468
H	4.3776123	-0.1757549	-1.5988916

```
F        4.0670279        1.9826915       -0.2469467
F        5.6440928        0.7325643        0.5198745
F        3.7113726        0.7442202        1.4767507
F        5.4735881       -1.9201567        0.0022762
F        3.7189695       -2.6076026       -1.0409363
F        3.5694927       -1.9817320        1.0112212
```

```
vf_tfo_hfip_ts_10_react.log
E =     -2787.887876
H =     -2787.602251
G =     -2787.712101
NImag=0
C       -0.1524716       -2.2523605        1.5871802
C        0.4963409       -1.3527399        2.5287197
N        0.2837282       -2.4744102        0.3883616
C       -0.4813113       -3.2614449       -0.5348751
H        0.0157199       -3.4005007       -1.4983616
O       -1.5568104       -3.6860783       -0.2350401
H       -1.0434424       -2.7999502        1.8800473
H        1.3974448       -0.8847514        2.1390085
H       -0.2427863       -0.5961630        2.8226757
H        0.7070197       -1.9169804        3.4431719
H        1.1260465       -1.9860710        0.0406463
O       -2.3622449       -0.9746162        0.7695673
C       -3.4823375       -1.1361627       -0.0325455
H       -2.1584882       -0.0242197        0.9367203
C       -4.6919115       -0.4959053        0.6521272
C       -3.2261249       -0.5905216       -1.4421101
H       -3.6961661       -2.2032218       -0.1400620
O       -1.4097269        1.5558341        1.1715510
F       -5.8060784       -0.5938697       -0.0699009
F       -4.4814851        0.7974817        0.9128770
F       -4.9047678       -1.1049001        1.8198410
F       -4.2319778       -0.8673728       -2.2716873
F       -2.1234102       -1.1532606       -1.9437201
F       -3.0405542        0.7305628       -1.4504556
C       -0.2573732        3.3174984       -0.3095296
F        0.8407994        3.5583278       -1.0191683
F       -0.3894946        4.2706564        0.6063713
F       -1.3057439        3.3618118       -1.1239542
O        1.0097721        1.7951211        1.3467020
S       -0.1374770        1.6844341        0.4932586
O        0.0139455        0.7729736       -0.6227393
H        1.6841092        0.0387687       -0.7745204
O        2.3150517       -0.5929062       -0.3656920
C        3.6381673       -0.3878765       -0.7299694
C        4.1988304        0.8077675        0.0493220
C        4.3566500       -1.7009586       -0.4280125
H        3.7749384       -0.1777852       -1.7949612
F        3.5664585        1.9154981       -0.3435732
F        5.5014653        0.9862235       -0.1677089
F        4.0123887        0.6818710        1.3624947
F        5.6495586       -1.6525309       -0.7340833
F        3.8029765       -2.6796725       -1.1471494
F        4.2473247       -2.0422996        0.8573314
```

```
vf_tfo_hfip_ts_11.log
E =     -2787.882206
H =     -2787.596555
```

G = -2787.701966
NImag=1

C	1.1033351	-2.6089653	0.9443152
C	1.8154565	-3.2807301	2.0509416
N	0.8124870	-3.3442931	-0.1499625
C	-0.1675930	-2.9460633	-1.0537856
H	-0.3020931	-3.6430400	-1.8867604
O	-0.8052381	-1.9343796	-0.9070920
H	0.3463182	-1.8704643	1.1932240
H	2.0720166	-2.5905779	2.8510645
H	1.1094872	-4.0080313	2.4614365
H	2.6952964	-3.8187446	1.6953647
H	1.3521335	-4.1826116	-0.3488782
O	2.3107774	-1.4109417	0.3306515
C	3.4282246	-0.9205596	1.0172325
H	1.9339853	-0.8023232	-0.4016553
C	4.5748713	-0.7502496	0.0151826
C	3.1077771	0.3552492	1.8120208
H	3.7614806	-1.6650756	1.7422650
O	1.3455094	0.0960217	-1.5379033
F	4.8906686	-1.9403078	-0.4909786
F	5.6523058	-0.2446538	0.6066907
F	4.2416630	0.0433930	-0.9990962
F	1.9667196	0.1852274	2.4767436
F	2.9777424	1.4286375	1.0392018
F	4.0716081	0.6009203	2.6960612
C	0.7882818	2.6104417	-1.7210549
F	-0.1412684	3.5111198	-1.4215565
F	0.9986024	2.6330647	-3.0328853
F	1.9156755	2.9399110	-1.1058463
O	-0.9563358	0.7623552	-2.0247837
S	0.2108969	0.9532848	-1.2192588
O	-0.0062842	1.0715099	0.2044494
H	-1.2927251	0.1863325	1.0748443
O	-2.0766466	-0.3124097	1.3918412
C	-3.0689896	-0.3170298	0.4099889
C	-3.9507723	-1.5224268	0.7214729
C	-3.8239389	1.0134782	0.4469777
H	-2.6851620	-0.4444275	-0.6082208
F	-4.9986567	-1.6059068	-0.0981912
F	-4.4119583	-1.4929385	1.9733977
F	-3.2409253	-2.6444475	0.5895133
F	-4.3801720	1.2470614	1.6372371
F	-4.7847269	1.0800833	-0.4747323
F	-2.9641824	2.0077763	0.2087532

vf_tfo_hfip_ts_11_prod.log
E = -2787.893538
H = -2787.606052
G = -2787.712293
NImag=0

C	0.9442367	-2.4983072	-0.3489751
C	1.5056050	-3.7597732	0.2491508
N	0.5999909	-2.6962045	-1.7211746
C	-0.3875260	-1.9850239	-2.3074078
H	-0.5903425	-2.2738229	-3.3452398
O	-1.0091235	-1.1001019	-1.7374976
H	0.0502673	-2.1690482	0.1824757
H	1.6557245	-3.6686248	1.3243892
H	0.7764976	-4.5538673	0.0905567

H	2.4435917	-4.0432490	-0.2346184
H	1.0950619	-3.3980436	-2.2593164
O	1.8851805	-1.3745966	-0.3175577
C	2.8733891	-1.3082089	0.6621858
H	1.4550511	0.0174661	-1.1902398
C	4.1010800	-0.6759580	0.0060558
C	2.3948579	-0.5291028	1.8963594
H	3.1916576	-2.2919516	1.0125405
O	1.1310610	0.8159777	-1.7106194
F	4.5907039	-1.5082438	-0.9107399
F	5.0514569	-0.4334363	0.9051030
F	3.8127756	0.4707025	-0.6101953
F	1.1576246	-0.9138913	2.2118320
F	2.3789048	0.7881748	1.7023526
F	3.1821691	-0.7742871	2.9425570
C	0.8761307	3.2160451	-0.8653583
F	0.0562040	4.0659583	-0.2715547
F	1.1844904	3.6638822	-2.0702341
F	1.9762236	3.0845354	-0.1473648
O	-1.0720689	1.8349474	-1.8814840
S	0.0239162	1.6059665	-1.0169735
O	-0.1771247	1.1292072	0.3105786
H	-1.3283253	-0.1554618	1.0060446
O	-2.0773262	-0.7515485	1.1911927
C	-3.1385229	-0.4587674	0.3322517
C	-3.9434222	-1.7492071	0.2166224
C	-3.9352932	0.7165614	0.9006316
H	-2.8267110	-0.1788881	-0.6798963
F	-5.0191031	-1.6059746	-0.5564826
F	-4.3505380	-2.1894652	1.4089920
F	-3.1819090	-2.7044142	-0.3196484
F	-4.3397409	0.4972540	2.1515076
F	-5.0079694	1.0119831	0.1671015
F	-3.1469785	1.7969937	0.9185970

vf_tfo_hfip_ts_1_prod.log
E = -2787.890892
H = -2787.603104
G = -2787.707236
NImag=0

C	1.6725831	-1.2037799	1.8293458
C	2.6953190	-1.1087634	2.9269006
N	1.2887275	-2.5543979	1.5672001
C	0.2033311	-2.7937873	0.7940321
H	-0.0180832	-3.8564999	0.6377473
O	-0.4726280	-1.8967387	0.3149464
H	0.7813624	-0.6249009	2.0768537
H	2.9593847	-0.0666324	3.0979738
H	2.2604467	-1.5108804	3.8422616
H	3.5978893	-1.6800009	2.7000166
H	1.8588578	-3.3212267	1.9034241
O	2.1173108	-0.5544585	0.6041776
C	3.2859895	-0.9925963	-0.0183737
H	0.9750300	0.2015609	-0.2577219
C	2.9882536	-1.5158253	-1.4368040
C	4.2556795	0.1959396	-0.0599280
H	3.7719779	-1.8096193	0.5219660
O	0.4372508	0.8193483	-0.8546811
F	1.9283708	-2.3184010	-1.4229887
F	4.0245801	-2.2088961	-1.9042665

F	2.7337951	-0.5310939	-2.3000657
F	5.3095768	-0.0594070	-0.8327535
F	4.6981245	0.4670965	1.1663218
F	3.6601480	1.2946320	-0.5212877
C	0.5963442	2.9826047	0.5756026
F	-0.1214929	3.9022218	1.1960766
F	1.3079906	3.5454499	-0.3832455
F	1.4062099	2.3937676	1.4435597
O	-1.3338935	2.4022648	-1.0986323
S	-0.5391822	1.7371035	-0.1360666
O	-1.1402136	1.0937827	0.9846537
H	-3.1528127	1.1348811	0.7615668
O	-4.0057055	1.3101062	0.3234842
C	-4.5143324	0.1737916	-0.3095086
C	-3.7596633	-0.1070077	-1.6139656
C	-4.5234504	-1.0052636	0.6631991
H	-5.5519915	0.3676931	-0.5894425
F	-3.7498916	0.9976695	-2.3585096
F	-4.3453992	-1.0714817	-2.3242703
F	-2.4894833	-0.4678576	-1.4100933
F	-4.9750170	-2.1254305	0.1036706
F	-5.3146401	-0.7145541	1.6971460
F	-3.3055897	-1.2515587	1.1532218

vf_tfo_hfip_ts_1_react.log
E = -2787.884427
H = -2787.597872
G = -2787.706422
NImag=0

C	1.2896875	-0.8031199	1.6165585
C	1.9862506	-0.4303191	2.8367283
N	1.1424109	-2.0258586	1.2177977
C	0.5403074	-2.3316169	-0.0565238
H	0.4076208	-3.4041350	-0.2202949
O	0.2471815	-1.4604993	-0.8139758
H	0.8250242	-0.0399706	0.9954881
H	2.8187404	0.2194941	2.5402169
H	1.3204874	0.1961417	3.4373698
H	2.3498222	-1.2829975	3.4050587
H	1.5106756	-2.8002998	1.7733286
O	3.0377080	-0.0778949	-0.3153077
C	4.0932167	-0.4924076	-1.1126528
H	2.3523347	0.3940704	-0.8487225
C	3.6101388	-1.5664791	-2.0922632
C	4.7547253	0.7028692	-1.8086670
H	4.8597519	-0.9521306	-0.4850339
O	1.1832756	1.3350898	-1.7167780
F	3.2143654	-2.6425079	-1.4011804
F	4.5649240	-1.9555583	-2.9349654
F	2.5663795	-1.1537177	-2.8110426
F	5.9112004	0.3629294	-2.3775439
F	5.0059602	1.6506498	-0.9051391
F	3.9797132	1.2438415	-2.7505296
C	1.3798163	3.3453410	-0.1340133
F	0.6852178	4.2881930	0.4919439
F	2.2235747	3.9247748	-0.9803670
F	2.0888512	2.6716163	0.7726767
O	-0.5328639	3.0563434	-1.8626757
S	0.2624236	2.2091644	-1.0257506
O	-0.4616978	1.5666714	0.0538531

H	-2.2545837	1.6868261	-0.2234949
O	-3.1802690	1.8377182	-0.5177182
C	-3.7083276	0.7434394	-1.2019497
C	-3.0055867	0.5351854	-2.5480489
C	-3.6943244	-0.4958718	-0.3059488
H	-4.7556243	0.9511707	-1.4331862
F	-3.0058659	1.6823977	-3.2245225
F	-3.6346378	-0.3762946	-3.2933331
F	-1.7358830	0.1430402	-2.4173556
F	-4.1968441	-1.5699050	-0.9137124
F	-4.4318672	-0.2576667	0.7801651
F	-2.4638337	-0.8044779	0.1136944

vf_tfo_hfip_ts_9.log
E = -2787.876640
H = -2787.591828
G = -2787.696995
NImag=1

C	0.6656659	-2.3719283	1.0977736
C	-0.0385212	-2.7193427	-0.1575704
N	-0.0395040	-1.7509801	2.0634990
C	0.4717055	-1.6118289	3.3470535
H	-0.2248675	-1.1462896	4.0510328
O	1.5863974	-1.9758474	3.6391129
H	1.4299184	-3.0491140	1.4649114
H	-0.7394832	-1.9390964	-0.4569092
H	0.6683053	-2.9296102	-0.9575748
H	-0.6070897	-3.6308237	0.0467585
H	-0.9179074	-1.2731524	1.8324004
O	1.8965103	-1.2006104	0.4481838
C	3.0558910	-0.7850947	1.1126339
H	1.6797006	-0.7211597	-0.4268531
C	4.2589697	-1.0309338	0.1954174
C	2.9015081	0.6709721	1.5687029
H	3.1950857	-1.3979431	2.0068487
O	1.2415961	-0.0211306	-1.7285318
F	5.3972692	-0.6622890	0.7720791
F	4.1465331	-0.3801968	-0.9615957
F	4.3304110	-2.3315049	-0.0811200
F	1.7915106	0.7909289	2.2976356
F	2.7960186	1.5096973	0.5397104
F	3.9312190	1.0528998	2.3158984
C	0.3597443	2.3306695	-2.3298100
F	1.3263287	2.9304391	-1.6487374
F	-0.7271155	3.0922779	-2.2942910
F	0.7463624	2.1909425	-3.5917406
O	-1.0811022	0.1928706	-2.4282633
S	-0.0207593	0.6974811	-1.6139004
O	-0.3599784	0.9879470	-0.2378038
H	-1.7881776	0.4302915	0.6317260
O	-2.3308959	-0.0113866	1.3248905
C	-3.6746235	-0.1484515	0.9840645
C	-4.1788639	1.1198528	0.2945982
C	-3.8786885	-1.4170384	0.1509642
H	-4.2647322	-0.2745121	1.8947679
F	-5.4606536	1.0295202	-0.0501582
F	-3.4738361	1.4005678	-0.8048910
F	-4.0462579	2.1535270	1.1259822
F	-5.1686716	-1.6761794	-0.0560805
F	-3.3568259	-2.4559817	0.8090560

F	-3.2753829	-1.3520468	-1.0353948

vf_tfo_hfip_ts_9_prod.log
E = -2787.889394
H = -2787.601115
G = -2787.705452
NImag=0

C	1.0883349	-2.2756940	-0.5613616
C	0.1029591	-2.2009008	-1.6954335
N	0.4313597	-2.5301636	0.6869795
C	1.1367458	-2.8837943	1.7789679
H	0.5274126	-3.0680466	2.6713492
O	2.3576294	-2.9946917	1.7799514
H	1.8416479	-3.0493706	-0.7275969
H	-0.5913066	-1.3710528	-1.5404492
H	0.6344710	-2.0456664	-2.6334151
H	-0.4648508	-3.1289780	-1.7555045
H	-0.5724775	-2.3907068	0.7598357
O	1.7956389	-1.0097824	-0.5270554
C	3.1534704	-1.0186841	-0.2057466
H	1.1312320	0.4170932	-0.8740407
C	3.9381844	-0.6052599	-1.4523219
C	3.4138748	-0.0786187	0.9852077
H	3.4956042	-2.0132555	0.0868983
O	0.7466719	1.3344162	-1.0768002
F	5.2216388	-0.3954199	-1.1666025
F	3.4491937	0.5001607	-2.0149581
F	3.8639753	-1.5762600	-2.3634858
F	2.3954271	-0.1046072	1.8384308
F	3.5794888	1.1910935	0.5995808
F	4.5146837	-0.4495354	1.6360778
C	0.2558560	3.0792855	0.7786568
F	1.1774987	2.6166986	1.6004207
F	-0.7250782	3.6274510	1.4749010
F	0.7866863	3.9807899	-0.0277850
O	-1.4558444	2.3224183	-1.0132127
S	-0.4519305	1.7207062	-0.2208191
O	-0.7619852	0.6634584	0.6860123
H	-2.0629997	-0.6688167	0.6564250
O	-2.5634910	-1.5023182	0.5505729
C	-3.8468108	-1.2893932	0.0482856
C	-4.5169060	-0.1233651	0.7767728
C	-3.7878347	-1.1028245	-1.4718230
H	-4.4531861	-2.1781273	0.2350124
F	-5.7809494	0.0480465	0.4007351
F	-3.8654624	1.0268992	0.5731588
F	-4.5058083	-0.3603515	2.0882385
F	-4.9944290	-0.9222772	-2.0051704
F	-3.2574607	-2.1959854	-2.0228869
F	-3.0240349	-0.0664026	-1.8260156

vf_tfo_hfip_ts_9_react.log
E = -2787.885708
H = -2787.599777
G = -2787.709190
NImag=0

C	0.9055817	-2.3171661	-1.1344993
C	0.1208381	-1.6566125	-2.1668505
N	0.4749858	-2.5324198	0.0678540

C	1.3528468	-3.0588293	1.0722454
H	0.8520753	-3.2606982	2.0225509
O	2.5142795	-3.2223893	0.8445562
H	1.8983565	-2.6945579	-1.3603003
H	-0.8314774	-1.2735419	-1.8101889
H	0.7339883	-0.8595951	-2.6024020
H	-0.0319228	-2.3780426	-2.9777442
H	-0.4661955	-2.2143266	0.3560228
O	2.6947512	-0.4858183	-0.5121126
C	3.7121651	-0.2699967	0.4070311
H	2.3044849	0.3592064	-0.8385683
C	4.8244755	0.5496704	-0.2515145
C	3.1631859	0.3746834	1.6842458
H	4.1448148	-1.2314147	0.6968960
O	1.3044771	1.7091958	-1.3894544
F	5.8269489	0.8206537	0.5815818
F	4.3640757	1.7076951	-0.7328723
F	5.3203888	-0.1371826	-1.2818268
F	2.1533168	-0.3614491	2.1570117
F	2.6953446	1.6047234	1.4677977
F	4.0879244	0.4505257	2.6406167
C	-0.2317246	3.3997095	-0.2038621
F	0.7104863	3.6866004	0.6857222
F	-1.4191225	3.5389988	0.3746936
F	-0.1449100	4.2640050	-1.2098176
O	-1.1002423	1.5497162	-1.7782672
S	-0.0314913	1.6987699	-0.8338709
O	-0.1525948	0.8697225	0.3493401
H	-1.5157275	-0.2324508	0.5192983
O	-1.9346354	-1.1055210	0.7114489
C	-3.3253741	-1.1020167	0.6734504
C	-3.8617658	0.1917827	1.2857394
C	-3.8175679	-1.3518231	-0.7562922
H	-3.7057738	-1.9280482	1.2790269
F	-5.1900614	0.2512494	1.2590350
F	-3.3855810	1.2667392	0.6518723
F	-3.4696079	0.2732740	2.5569745
F	-5.1391208	-1.5105046	-0.8047416
F	-3.2565123	-2.4745707	-1.2138439
F	-3.4897177	-0.3706608	-1.5960156

vinylformamide_1.log
E = -247.089960
H = -247.004105
G = -247.038688
NImag=0

C	0.5780505	-0.4192310	0.0000000
C	0.5884068	-1.7468967	0.0000000
N	-0.5873628	0.3526623	0.0000000
C	-0.5640194	1.7039169	0.0000000
H	-1.5608891	2.1601222	0.0000000
O	0.4633470	2.3720105	0.0000000
H	1.4853951	0.1720932	0.0000000
H	1.5291417	-2.2800791	0.0000000
H	-0.3272277	-2.3280567	0.0000000
H	-1.4890421	-0.1107415	0.0000000

vinylformamide_2.log
E = -247.088600

```
H =       -247.002471
G =       -247.037005
NImag=0
C        -0.1433525       -0.7920457       -0.0000199
C         1.1823307       -0.9166701        0.0000148
N        -0.9008178        0.3868383       -0.0000204
C        -0.4276710        1.6524415       -0.0000103
H        -1.2234473        2.4081749        0.0000198
O         0.7588447        1.9525263        0.0000421
H        -0.7731185       -1.6736107       -0.0000531
H         1.5942528       -1.9180002        0.0000080
H         1.8656729       -0.0813503        0.0000504
H        -1.9080940        0.2848959       -0.0000314
```

IV.5 Geometries in Gas Phase

Filename (cf. Tables S7), cartesian coordinates (in Å), electronic energies (in Hartree), enthalpies (in Hartree), Gibbs free energies (in Hartree from Gaussian; for the value including qh corrections, see Table S5), and the number of the imaginary vibrational frequencies (NImag) for all stationary points at the SMD(HFIP)/MN15/def2-TZVP level of theory.

```
gas_hfip_1.log
E =       -789.494909
H =       -789.421675
G =       -789.468126
NImag=0
O         0.0035212       -1.2409075       -0.2436701
C        -0.5800302       -0.1829599       -0.9247471
H         0.8887743       -1.4074488       -0.5990536
C         0.2382353        1.0945499       -0.7028157
F         1.4998562        0.9206041       -1.1095490
F         0.2683107        1.3756034        0.5965763
F        -0.2659770        2.1443079       -1.3407663
C        -0.7556413       -0.5485858       -2.4033876
F        -1.3016722        0.4318903       -3.1131687
F        -1.5418763       -1.6166416       -2.5003735
F         0.4223962       -0.8607576       -2.9529794
H        -1.5744957       -0.0090533       -0.5115653
```

```
gas_hfip_2.log
E =       -789.492916
H =       -789.419831
G =       -789.465230
NImag=0
O        -0.1383322       -1.3973007       -0.4327666
C        -0.6661384       -0.2612791       -1.0275229
H        -0.1278273       -1.2951741        0.5270241
C         0.1740449        0.9756372       -0.6861054
F         1.3738837        0.9594719       -1.2478005
F         0.3454005        1.0008284        0.6452278
F        -0.4440925        2.0991376       -1.0339235
C        -0.7261426       -0.5424166       -2.5298861
F        -1.0961688        0.5522335       -3.1946377
F        -1.6283151       -1.4928875       -2.7644466
F         0.4385320       -0.9511175       -3.0099266
H        -1.6917242       -0.0355321       -0.7119481
```

```
gas_iproh_1.log
E =      -194.196823
H =      -194.082420
G =      -194.116379
NImag=0
O        0.0056137        1.9104279       -0.1869178
C       -0.0001877        0.5388646       -0.5466321
H        0.0077996        1.9740878        0.7766610
C        1.2559432       -0.1528881       -0.0468705
C       -1.2576912       -0.1443848       -0.0387118
H       -0.0037392        0.5387362       -1.6393945
H       -1.2638678       -0.1424393        1.0562138
H       -2.1460513        0.3804796       -0.3894137
H       -1.3064255       -1.1828036       -0.3710948
H        1.2955798       -1.1915508       -0.3797114
H        1.2691956       -0.1511390        1.0479846
H        2.1455077        0.3660895       -0.4032279


gas_iproh_2.log
E =      -194.196971
H =      -194.082534
G =      -194.116415
NImag=0
O       -0.1920725        1.8824304       -0.1239434
C       -0.0707663        0.5396346       -0.5658230
H        0.5952404        2.3747501       -0.3830121
C        1.2232663       -0.0770603       -0.0649315
C       -1.2840961       -0.1960311       -0.0395179
H       -0.0827639        0.5110754       -1.6642820
H       -1.2769907       -0.1747214        1.0522462
H       -2.1995659        0.2817749       -0.3874388
H       -1.2794962       -1.2351397       -0.3710134
H        1.3181304       -1.1124835       -0.3967184
H        1.2397658       -0.0556219        1.0268840
H        2.0909406        0.4752255       -0.4345958


gas_vf_hfip_prod_1.log
E =     -1036.628352
H =     -1036.465631
G =     -1036.525324
NImag=0
C        1.0284430       -0.2699946        1.3449026
C        0.5313698       -1.0866998        2.5127224
N        1.8259540       -1.0760302        0.4559551
C        2.8651285       -0.5790115       -0.2680939
H        3.3582066       -1.3422257       -0.8886869
O        3.2342269        0.5759020       -0.2383215
O       -0.1234431        0.2410956        0.6890619
C        0.0980894        1.3121390       -0.1740017
C       -0.5035194        0.9555929       -1.5333404
F        0.1596467       -0.0898869       -2.0384323
F       -0.3953582        1.9630007       -2.3953646
F       -1.7837401        0.6161297       -1.4446267
C       -0.5048735        2.5695776        0.4574179
F       -0.2490852        3.6562028       -0.2653336
F        0.0460809        2.7399027        1.6638868
F       -1.8193874        2.4750969        0.6215349
H        1.6584601        0.5630478        1.6673766
H       -0.0660511       -0.4613339        3.1724516
```

H	1.3722685	-1.4998175	3.0674480
H	-0.0972641	-1.9021469	2.1500015
H	1.5310914	-2.0276845	0.2833366
H	1.1637571	1.5114435	-0.3346933

gas_vf_hfip_prod_2.log
E = -1036.619139
H = -1036.456612
G = -1036.516107
NImag=0

C	1.1355313	0.0032396	0.9707947
C	1.4264801	-0.3819117	2.4025103
N	1.7460450	-0.8976989	0.0211425
C	2.9133531	-0.5954212	-0.6046045
H	3.2309089	-1.3716130	-1.3184668
O	3.5476859	0.4171598	-0.4058081
O	-0.2770186	-0.0603082	0.7922276
C	-0.9268087	0.9214589	0.0648403
C	-0.8523183	2.3169218	0.7218166
F	-0.8802011	2.1835256	2.0458218
F	-1.9024108	3.0491511	0.3555821
F	0.2472681	2.9988303	0.4062065
C	-0.5326237	0.9548019	-1.4194844
F	-0.7219238	-0.2550352	-1.9478630
F	0.7338635	1.2958102	-1.6283103
F	-1.3062342	1.8202536	-2.0739987
H	1.5275019	0.9983868	0.7493032
H	0.9651635	0.3306921	3.0850749
H	2.5047741	-0.3880710	2.5594576
H	1.0278844	-1.3771987	2.6044040
H	1.2894600	-1.7820450	-0.1534425
H	-1.9874807	0.6607682	0.0710961

gas_vf_hfip_prod_4.log
E = -1036.620220
H = -1036.457895
G = -1036.518126
NImag=0

C	1.5846016	0.1897504	0.6654462
C	1.6560238	0.4809760	2.1485602
N	1.9479831	-1.1714875	0.3548865
C	3.2487751	-1.5410332	0.2901502
H	3.3892865	-2.5967733	0.0120926
O	4.1711396	-0.7857153	0.5162627
O	0.2557249	0.3146710	0.1727280
C	-0.1883384	1.6100151	-0.0395582
C	-0.2169792	1.8908636	-1.5475328
F	-1.0859991	1.1181387	-2.1860723
F	0.9965245	1.6474919	-2.0468219
F	-0.5165527	3.1623599	-1.7997514
C	-1.5538458	1.7531913	0.6390414
F	-1.3961569	1.6089665	1.9560821
F	-2.4133025	0.8317950	0.2267051
F	-2.0822495	2.9539252	0.4168527
H	2.2564360	0.8521737	0.1080670
H	1.3858571	1.5167086	2.3620341
H	2.6777577	0.3127866	2.4877001
H	0.9737606	-0.1795506	2.6838877
H	1.2029219	-1.8231469	0.1547038

H	0.4657327	2.3663942	0.4088381

gas_vf_hfip_prod_5.log
E = -1036.618224
H = -1036.455764
G = -1036.516181
NImag=0

C	1.5287576	0.1329444	0.7047595
C	0.9361380	-0.1382362	2.0712591
N	2.1577077	-1.0452852	0.1524710
C	3.3983809	-1.4194882	0.5433898
H	3.7745336	-2.3060952	0.0099171
O	4.0348730	-0.8411179	1.3997526
O	0.5676463	0.4735168	-0.2912431
C	-0.5213512	1.2627505	0.0310606
C	-0.1606091	2.7512949	-0.0559469
F	-1.1766351	3.5246910	0.3153470
F	0.2225361	3.1094636	-1.2729598
F	0.8563401	2.9862889	0.7825128
C	-1.6404041	0.8487955	-0.9294723
F	-1.2784549	0.9566165	-2.2002066
F	-2.7341448	1.5820414	-0.7386031
F	-1.9546316	-0.4266216	-0.6964602
H	2.2886907	0.9147067	0.7785211
H	0.5950797	0.7761625	2.5582804
H	1.7283759	-0.5643984	2.6857093
H	0.1143458	-0.8541079	1.9974761
H	1.6566100	-1.5478231	-0.5661438
H	-0.9077834	1.0911981	1.0406803

gas_vf_hfip_prod_6.log
E = -1036.618261
H = -1036.455716
G = -1036.514265
NImag=0

C	1.8766595	0.5259076	0.6942150
C	1.4787954	0.3579933	2.1435419
N	1.8614148	-0.7486009	-0.0162437
C	2.8022239	-1.6974883	0.2430258
H	2.7617583	-2.5438516	-0.4606517
O	3.6013245	-1.6360046	1.1518287
O	1.0646830	1.4392782	-0.0290677
C	-0.3015006	1.1995605	-0.0932055
C	-1.0819662	2.3022929	0.6452432
F	-0.5513203	2.5134998	1.8454366
F	-2.3510235	1.9341945	0.8194466
F	-1.0683148	3.4532079	-0.0173068
C	-0.6966085	1.1410802	-1.5767034
F	-2.0153598	1.0949491	-1.7293992
F	-0.1870903	0.0233863	-2.1227230
F	-0.2237382	2.1668402	-2.2632813
H	2.8782577	0.9565580	0.6384177
H	1.5413749	1.3126017	2.6614170
H	2.1667291	-0.3485519	2.6034210
H	0.4656709	-0.0372759	2.2371434
H	1.4312632	-0.7547748	-0.9319343
H	-0.5962319	0.2433969	0.3518798

gas_vf_hfip_prod_7.log
E = -1036.622633
H = -1036.459638
G = -1036.518395
NImag=0

C	0.7247727	-0.2857897	1.6265700
C	0.5762732	-1.6432660	0.9667752
N	2.0912514	0.1899777	1.6752389
C	3.0201660	0.1134516	0.6825969
H	3.9803343	0.5622452	0.9829191
O	2.8549738	-0.3847290	-0.4099467
O	-0.1635507	0.6926178	1.0955010
C	-0.0155865	1.0482662	-0.2429472
C	-1.3183711	0.7226003	-0.9854270
F	-1.1950180	0.9341755	-2.2939447
F	-2.3440921	1.4409061	-0.5411566
F	-1.6181134	-0.5651612	-0.8096892
C	0.3306728	2.5417908	-0.3014599
F	1.5346907	2.7389639	0.2481893
F	-0.5393071	3.2843492	0.3714404
F	0.3811995	2.9853873	-1.5542484
H	0.3986995	-0.3622698	2.6633192
H	-0.4359635	-2.0044425	1.1377533
H	1.2873727	-2.3379616	1.4133627
H	0.7688439	-1.6092462	-0.1016496
H	2.3330921	0.7802399	2.4588161
H	0.7939577	0.5092955	-0.7474126

gas_vf_hfip_prod_8.log
E = -1036.613677
H = -1036.451073
G = -1036.509717
NImag=0

C	1.3650251	0.1582871	0.5416558
C	1.4402324	1.1161354	1.7100399
N	1.8416959	-1.1573614	0.9004452
C	3.1655819	-1.4402386	0.9091374
H	3.3847010	-2.4804350	1.1966170
O	4.0286685	-0.6350260	0.6287587
O	0.0340859	-0.1045093	0.1004275
C	-0.7564381	0.8476359	-0.5147657
C	-1.5669443	1.7496101	0.4533029
F	-1.7688919	1.1159704	1.6024796
F	-2.7570047	2.0099923	-0.0912895
F	-0.9944361	2.9222102	0.7223833
C	-0.0456892	1.6708634	-1.5975223
F	0.9051212	2.4759788	-1.1267232
F	-0.9368936	2.4236538	-2.2392982
F	0.5221053	0.8458218	-2.4739401
H	1.9866029	0.5107140	-0.2852497
H	1.1490888	2.1250079	1.4250884
H	2.4760854	1.1437644	2.0489055
H	0.8018131	0.7649550	2.5213927
H	1.1575935	-1.8434238	1.1853465
H	-1.5244031	0.2914959	-1.0584926

gas_vf_iproh_prod_1.log
E = -441.329146
H = -441.125224

G =   -441.173846
NImag=0

C	1.7103725	-0.8517773	-0.1214005
C	1.8936879	-2.1380558	-0.8993755
N	2.9382835	-0.0877722	-0.1517651
C	3.1522348	0.9297878	0.7209265
H	4.0844286	1.4830464	0.5205584
O	2.4175346	1.2047052	1.6453978
O	0.6577050	-0.1087009	-0.6779200
C	-0.4226765	0.1849577	0.2052936
C	-1.2171489	1.2981980	-0.4403423
C	-1.2708612	-1.0557868	0.4310799
H	1.4955974	-1.0530474	0.9337545
H	0.9755901	-2.7227298	-0.8583092
H	2.7161710	-2.7290708	-0.4959077
H	2.0961989	-1.9037513	-1.9467744
H	3.5263557	-0.1545429	-0.9707447
H	-0.0012247	0.5315277	1.1561369
H	-1.5957870	0.9668975	-1.4096548
H	-2.0644427	1.5758795	0.1878908
H	-0.5888269	2.1751279	-0.5924899
H	-1.6646620	-1.4100540	-0.5246554
H	-2.1083749	-0.8342061	1.0946581
H	-0.6884431	-1.8604874	0.8858392

gas_vf_iproh_prod_1_2.log
E =   -441.332097
H =   -441.127969
G =   -441.175721
NImag=0

C	1.6774712	0.8817101	0.4447324
C	2.3497921	1.6440334	1.5645922
N	2.5813911	-0.1144482	-0.0976672
C	2.6720236	-0.3969320	-1.4226088
H	3.4104572	-1.1869075	-1.6385509
O	2.0303934	0.1476695	-2.2958205
O	0.5232939	0.2999955	0.9831455
C	-0.3520473	-0.3407303	0.0590882
C	-1.3539634	-1.1030819	0.9004117
C	-1.0340396	0.6619069	-0.8557446
H	1.4308971	1.5417226	-0.3913502
H	1.6647612	2.3876575	1.9670595
H	3.2506037	2.1388659	1.2036947
H	2.6144092	0.9544462	2.3691243
H	3.1280892	-0.6640439	0.5512898
H	0.2299361	-1.0465177	-0.5475507
H	-0.8450847	-1.7986042	1.5676676
H	-2.0411993	-1.6614328	0.2638920
H	-1.9317657	-0.4025230	1.5068071
H	-1.5424922	1.4192154	-0.2543969
H	-1.7773198	0.1517938	-1.4705837
H	-0.3265692	1.1427257	-1.5309521

gas_vf_iproh_prod_2.log
E =   -441.328382
H =   -441.124218
G =   -441.171903
NImag=0

C	1.2419525	-1.0424091	-0.3186263

C	1.7413312	-2.4554137	-0.5378590
N	2.3065751	-0.0790149	-0.5393931
C	2.7927552	0.7449035	0.4197721
H	3.5869515	1.4106285	0.0433917
O	2.4171436	0.7718833	1.5739493
O	0.1909210	-0.8208185	-1.2179675
C	-0.9012082	-0.0200729	-0.7672227
C	-1.7245752	-0.7650419	0.2713302
C	-0.4637579	1.3446629	-0.2632588
H	0.9308557	-0.8976504	0.7193715
H	0.9429153	-3.1670203	-0.3305309
H	2.5914749	-2.6644699	0.1107837
H	2.0463203	-2.5757847	-1.5792899
H	2.6823442	-0.0111285	-1.4753686
H	-1.5058300	0.1093957	-1.6676286
H	0.1431069	1.8593601	-1.0092759
H	0.1136984	1.2595624	0.6604686
H	-1.3439584	1.9539916	-0.0517760
H	-1.1722590	-0.8758249	1.2075144
H	-2.6376473	-0.2111305	0.4946388
H	-1.9974499	-1.7545787	-0.0963550

gas_vf_iproh_prod_4.log
E = -441.326490
H = -441.122754
G = -441.172404
NImag=0

C	-1.6029813	-0.4531789	-0.1935477
C	-2.0196296	-1.7199558	-0.9137861
N	-2.5353206	-0.0997230	0.8596990
C	-3.7189117	0.4914732	0.5908566
H	-4.3027381	0.7279788	1.4951058
O	-4.1114203	0.7449894	-0.5310198
O	-0.3614645	-0.6136439	0.4408856
C	0.7577301	-0.1938696	-0.3304841
C	0.9351931	1.3110526	-0.2265155
C	1.9625647	-0.9422845	0.1967182
H	-1.5783925	0.3835572	-0.9030084
H	-1.3006343	-1.9586844	-1.6996141
H	-3.0019720	-1.5801428	-1.3645861
H	-2.0548348	-2.5449487	-0.2014167
H	-2.2383676	-0.2703555	1.8095346
H	0.5846043	-0.4746015	-1.3790475
H	1.1427946	1.5806273	0.8109058
H	1.7681477	1.6443213	-0.8474594
H	0.0384356	1.8424950	-0.5487752
H	2.1072372	-0.7007964	1.2515904
H	2.8612132	-0.6558238	-0.3507282
H	1.8183937	-2.0183730	0.1047378

gas_vf_iproh_prod_5.log
E = -441.326676
H = -441.122767
G = -441.170808
NImag=0

C	1.6033929	-0.0437673	0.2108671
C	1.8457271	0.0361549	1.7070821
N	2.6378228	-0.8146607	-0.4480519
C	3.8539021	-0.2930941	-0.7146209

H	4.5150940	-0.9885268	-1.2566951
O	4.1987760	0.8269572	-0.3930876
O	0.4201856	-0.7193320	-0.1321375
C	-0.7886866	-0.2274464	0.4281001
C	-1.0158718	1.2361960	0.0886908
C	-1.8884356	-1.1051502	-0.1309227
H	1.6242021	0.9654724	-0.2185025
H	1.1357744	0.7076562	2.1923808
H	2.8502797	0.4242740	1.8727562
H	1.7603475	-0.9587713	2.1480512
H	2.3859448	-1.7433213	-0.7545810
H	-0.7520424	-0.3476097	1.5188987
H	-0.9610842	1.3757218	-0.9932835
H	-2.0050652	1.5451614	0.4288809
H	-0.2816032	1.8903983	0.5615618
H	-1.9459416	-0.9780024	-1.2136031
H	-2.8509135	-0.8357796	0.3050384
H	-1.6850899	-2.1537585	0.0847547

gas_vf_iproh_prod_6.log
E = -441.323860
H = -441.119953
G = -441.167782
NImag=0

C	1.6824318	-0.5459688	0.4303071
C	1.7094150	-1.8357855	-0.3680948
N	2.3279526	0.5448222	-0.3096066
C	3.6706420	0.5891855	-0.4837135
H	4.0175021	1.5358467	-0.9303022
O	4.4287342	-0.3129229	-0.1920122
O	0.4060630	-0.1182123	0.8055674
C	-0.5572668	0.0565090	-0.2332852
C	-1.5616498	-1.0807262	-0.1992300
C	-1.2278878	1.3977550	-0.0043325
H	2.2331302	-0.6947229	1.3625835
H	1.2405287	-2.6329680	0.2090997
H	2.7460002	-2.1014978	-0.5682616
H	1.1883011	-1.7234131	-1.3201200
H	1.8045322	1.4073521	-0.3786919
H	-0.0446447	0.0698130	-1.2031415
H	-2.0453084	-1.1039999	0.7791549
H	-2.3282138	-0.9403906	-0.9633190
H	-1.0762590	-2.0418694	-0.3659490
H	-1.6834791	1.4111749	0.9873208
H	-2.0050516	1.5750837	-0.7489211
H	-0.5041862	2.2135775	-0.0549473

gas_vf_iproh_prod_7.log
E = -441.324896
H = -441.120497
G = -441.168251
NImag=0

C	1.6497550	0.9617309	-0.9077440
C	1.7381977	2.1468535	0.0390854
N	2.5296334	-0.1431992	-0.5368038
C	2.9303742	-0.5313901	0.7028324
H	3.5851590	-1.4192599	0.6534469
O	2.6452567	-0.0051166	1.7564933
O	0.3370110	0.5228890	-1.1210661

```
C        -0.3444806       -0.0040966        0.0198104
C        -1.5356724        0.8841780        0.3233052
C        -0.7587997       -1.4278496       -0.2977904
H         1.9864756        1.2832729       -1.8953936
H         1.2069812        2.9813630       -0.4187133
H         2.7788613        2.4278311        0.2003703
H         1.3014205        1.9273885        1.0094297
H         2.7905507       -0.7621340       -1.2918812
H         0.3372730       -0.0099435        0.8766998
H        -2.0965413        0.5021757        1.1779001
H        -2.1985774        0.9156732       -0.5437929
H        -1.2107459        1.9016540        0.5449184
H        -1.3956347       -1.4354553       -1.1847540
H        -1.3126639       -1.8659753        0.5339654
H         0.1184866       -2.0453466       -0.4974051
```

```
gas_vf_iproh_prod_8.log
E =       -441.322487
H =       -441.118424
G =       -441.166160
NImag=0
C         1.4408945       -0.2122719       -0.0602272
C         1.2969148       -1.0505809        1.1981281
N         2.6457381       -0.5744454       -0.7831893
C         3.8656935       -0.1521692       -0.3911670
H         4.6747620       -0.4768808       -1.0655122
O         4.0672811        0.5170708        0.6033853
O         0.4258552       -0.4123498       -1.0079882
C        -0.8983969        0.0436615       -0.7448420
C        -1.7407151       -1.0334410       -0.0765452
C        -0.9452370        1.3583784        0.0137619
H         1.5328082        0.8450919        0.2141135
H         0.4862735       -0.6841213        1.8281201
H         2.2249564       -0.9863138        1.7662924
H         1.1008155       -2.0894405        0.9270251
H         2.5213446       -1.1026560       -1.6347729
H        -1.3078375        0.2146889       -1.7440725
H        -2.7950098       -0.7522987       -0.1113638
H        -1.4650182       -1.1736178        0.9689966
H        -1.6187801       -1.9822436       -0.5991942
H        -0.6080438        1.2408116        1.0462136
H        -0.3317300        2.1186878       -0.4710207
H        -1.9750002        1.7163678        0.0417374
```

IV.6 Geometries in *i*PrOH

Filename (cf. Tables S8), cartesian coordinates (in Å), electronic energies (in Hartree), enthalpies (in Hartree), Gibbs free energies (in Hartree from Gaussian; for the value including qh corrections, see Table S5), and the number of the imaginary vibrational frequencies (NImag) for all stationary points at the SMD(HFIP)/MN15/def2-TZVP level of theory.

```
iproh_h2o_1.log
E =       -270.607431
H =       -270.466029
G =       -270.508942
```

NImag=0

O	-0.0761289	0.9058977	1.5268112
C	0.0243601	-0.3385763	0.8424880
H	-0.1318164	1.6152590	0.8565670
C	-1.2145410	-0.5866314	0.0012968
C	1.2866143	-0.3846162	0.0007329
H	0.0858945	-1.0976931	1.6274273
O	-0.2349501	2.9008718	-0.5543856
H	-1.0488108	3.4225703	-0.5489762
H	0.4878414	3.5428489	-0.5535731
H	-1.2874292	0.1708249	-0.7854417
H	-2.1150352	-0.5359119	0.6163300
H	-1.1742896	-1.5688787	-0.4743348
H	1.4041903	-1.3606571	-0.4748473
H	1.2366690	0.3745636	-0.7861277
H	2.1676392	-0.1900609	0.6152797

iproh_h2o_2.log
E = -270.607304
H = -270.465980
G = -270.509821
NImag=0

O	-0.5305595	-1.3829129	0.5497047
C	0.0787060	-0.4303535	-0.3141659
H	-1.4819434	-1.4345866	0.3336565
C	-0.5570536	0.9360216	-0.1367924
C	1.5544672	-0.4069096	0.0178701
H	-0.0504640	-0.7525974	-1.3560515
O	-3.2845253	-1.4664128	-0.2282024
H	-3.6204803	-2.3508660	-0.4269705
H	-3.8963545	-1.0938758	0.4208361
H	-1.6270611	0.8982039	-0.3546347
H	-0.4237899	1.2753645	0.8943635
H	-0.0989551	1.6682917	-0.8053335
H	2.0854618	0.2906338	-0.6321179
H	1.6947451	-0.0893918	1.0549545
H	1.9950188	-1.3982189	-0.1035391

vf_iproh_4_ts.log
E = -441.753862
H = -441.539264
G = -441.587811
NImag=1

C	-1.0043101	1.0890095	-0.4860923
C	-0.7769581	2.4088639	0.1233439
N	-1.5202683	0.1015387	0.2281974
C	-1.9837498	-1.0895153	-0.3504322
H	-1.8900828	-1.1068449	-1.4435750
O	-2.4374464	-1.9789466	0.3152287
H	-1.1339671	1.0049512	-1.5588892
H	-0.0687437	2.9873674	-0.4621754
H	-1.7421485	2.9267597	0.1050462
H	-0.4479156	2.3200932	1.1577410
H	-1.5601210	0.1728325	1.2465622
O	0.9734519	0.5922738	-0.7595823
C	1.4793039	-0.4907683	0.0482716
H	1.0478094	0.3547076	-1.6991138
C	2.7618277	-1.0035746	-0.5677111
C	1.6825487	0.0511332	1.4432858

H	0.7315681	-1.2931180	0.0594755
H	3.5152566	-0.2130652	-0.5784591
H	2.5957833	-1.3495117	-1.5902285
H	3.1425020	-1.8415394	0.0174201
H	2.0750670	-0.7353377	2.0887069
H	2.3994832	0.8750066	1.4202723
H	0.7496388	0.4128334	1.8790623

vf_iproh_5_ts.log
E = -441.751575
H = -441.536854
G = -441.584965
NImag=1

C	-0.5763821	1.2603714	-0.4295085
C	0.0455011	2.4643644	0.1531695
N	-1.2740118	0.4416711	0.3554638
C	-2.1389978	-0.5292374	-0.1517612
H	-2.2286563	-0.5031522	-1.2453698
O	-2.7194575	-1.3005025	0.5645375
H	-0.8926396	1.2865033	-1.4659903
H	0.8530566	2.8307857	-0.4735221
H	-0.7426354	3.2248216	0.1735408
H	0.3853126	2.2910257	1.1723454
H	-1.1330947	0.4646452	1.3664900
O	1.0394261	0.3139469	-1.0233540
C	1.7998332	-0.6621470	-0.2599498
H	0.8320329	-0.0536348	-1.8988382
C	2.1158656	-0.0549913	1.0850935
C	1.0566434	-1.9762979	-0.1523010
H	2.7241571	-0.8073674	-0.8244612
H	1.2080739	0.0588264	1.6826666
H	2.6005881	0.9159071	0.9761520
H	2.7901815	-0.7194713	1.6261986
H	1.7292546	-2.7374246	0.2461346
H	0.2025373	-1.8990315	0.5231094
H	0.7097864	-2.3123578	-1.1319736

vf_iproh_6_ts.log
E = -441.751531
H = -441.536875
G = -441.585735
NImag=1

C	0.6353492	0.7494741	0.4445163
C	0.0093127	2.0587398	0.1924444
N	1.8026030	0.4594949	-0.1148739
C	2.5504611	-0.6615841	0.2581837
H	2.0992950	-1.2303437	1.0806160
O	3.5829341	-0.9444133	-0.2865559
H	0.4095305	0.2087945	1.3558867
H	-1.0293123	2.0598073	0.5088360
H	0.5553431	2.7854268	0.8036098
H	0.1011289	2.3486461	-0.8536070
H	2.1499738	1.0166143	-0.8962645
O	-0.5836176	-0.4813857	-0.5486712
C	-1.9600717	-0.8072810	-0.2243369
H	-0.4962236	-0.2845905	-1.4974276
C	-2.0862241	-0.7862363	1.2804170
C	-2.9161895	0.1417956	-0.9100071
H	-2.1203581	-1.8240213	-0.5926820

H	-1.9478106	0.2275471	1.6655467
H	-1.3551553	-1.4500709	1.7453607
H	-3.0832659	-1.1236496	1.5641694
H	-3.9418376	-0.1875742	-0.7371135
H	-2.8104807	1.1555886	-0.5194886
H	-2.7439472	0.1568556	-1.9884414

vf_iproh_7_ts.log
E = -441.752759
H = -441.537984
G = -441.586796
NImag=1

C	0.8140554	0.9103863	0.4802989
C	0.4568827	2.3290400	0.3371315
N	1.8695742	0.4153999	-0.1324805
C	2.3391198	-0.8849985	0.1146148
H	1.7718252	-1.4089199	0.8936450
O	3.2715216	-1.3407862	-0.4859405
H	0.4471216	0.3425117	1.3290693
H	-0.5905113	2.4926258	0.5798894
H	1.0585664	2.8729253	1.0739924
H	0.6977259	2.7064881	-0.6554091
H	2.3306640	0.9458074	-0.8739314
O	-0.7847917	0.0832155	-0.6181038
C	-1.9599219	-0.1584551	0.1868419
H	-0.9873286	0.7470139	-1.2986363
C	-3.1932158	-0.0750361	-0.6829697
C	-1.7856984	-1.5192947	0.8195597
H	-1.9995047	0.6165556	0.9627082
H	-3.1521969	-0.8362786	-1.4654828
H	-3.2772936	0.9092732	-1.1493123
H	-4.0862380	-0.2439979	-0.0800061
H	-2.6143318	-1.7241978	1.4982418
H	-1.7675950	-2.2898497	0.0455515
H	-0.8555282	-1.5753931	1.3893820

vf_iproh_8_ts.log
E = -441.752139
H = -441.537704
G = -441.586999
NImag=1

C	0.8565940	0.8270972	0.5345046
C	0.2945862	2.1627965	0.2938812
N	1.9154274	0.4120529	-0.1309369
C	2.5900129	-0.7751886	0.1985416
H	2.1737656	-1.2799538	1.0786181
O	3.5258264	-1.1642160	-0.4426514
H	0.6421945	0.3058259	1.4602866
H	-0.7187852	2.2361141	0.6793202
H	0.9186692	2.8628037	0.8615423
H	0.3401898	2.4341563	-0.7598434
H	2.2387907	0.9219697	-0.9548372
O	-0.6594883	-0.3670266	-0.2818787
C	-2.0054621	-0.2529283	0.2275996
H	-0.6596521	-0.2969508	-1.2511752
C	-2.8458287	0.5882698	-0.7066338
C	-2.5745073	-1.6404432	0.4288116
H	-1.8993038	0.2481378	1.1948762
H	-2.9267279	0.0933977	-1.6791231

H	-2.4100706	1.5782506	-0.8514814
H	-3.8518441	0.7087178	-0.3031832
H	-3.5655572	-1.5772729	0.8813548
H	-2.6658338	-2.1508494	-0.5331314
H	-1.9317868	-2.2325782	1.0814487

vf_iproh_9_ts.log
E = -441.754200
H = -441.539978
G = -441.589613
NImag=1

C	1.1512004	1.0072450	0.4009732
C	1.3723804	2.3958425	-0.0183202
N	1.6580314	0.0030212	-0.2780681
C	1.6686042	-1.3172592	0.2125559
H	1.2395814	-1.4090651	1.2178750
O	2.1212425	-2.2194564	-0.4333992
H	0.8403048	0.7840555	1.4151137
H	0.6014441	3.0509834	0.3785045
H	2.3274247	2.6974092	0.4277686
H	1.4488802	2.4830831	-1.1003512
H	2.0407135	0.1550016	-1.2135691
O	-0.9102521	0.9601381	-0.2168391
C	-1.5705258	-0.3148099	-0.1397608
H	-1.4221855	1.6191959	0.2818213
C	-1.8275675	-0.6907793	1.3030146
C	-2.8412762	-0.2721409	-0.9618449
H	-0.8689168	-1.0181090	-0.5985905
H	-2.5219589	0.0233604	1.7549701
H	-0.9029258	-0.6913384	1.8839574
H	-2.2727961	-1.6847535	1.3627396
H	-3.3179343	-1.2535761	-0.9668628
H	-3.5417009	0.4472694	-0.5294064
H	-2.6283337	0.0180424	-1.9912005

vf_iproh_add_1.log
E = -441.765845
H = -441.548918
G = -441.596154
NImag=0

C	-0.7119410	1.0230844	-0.3142563
C	-0.0978301	2.3206006	0.1105243
N	-1.5016097	0.4085394	0.6946751
C	-2.1486272	-0.7414520	0.4058453
H	-2.7911174	-1.1277954	1.2023867
O	-2.0030461	-1.3048667	-0.6741139
H	-1.2754852	1.1097778	-1.2418916
H	0.5328268	2.7112972	-0.6852349
H	-0.9069520	3.0271924	0.2949288
H	0.4833332	2.2115359	1.0264253
H	-1.5546141	0.8153694	1.6221254
O	0.3615772	0.0612748	-0.7211833
C	1.3840473	-0.3645255	0.2994872
H	-0.1140762	-0.7166729	-1.1081744
C	1.4005518	-1.8696090	0.3094920
C	2.6867301	0.2708631	-0.1015617
H	1.0109026	0.0265993	1.2461846
H	3.0120720	-0.1114395	-1.0701957
H	2.6038712	1.3562437	-0.1516741

```
H        3.4401328        0.0165751        0.6447558
H        1.6881355       -2.2555260       -0.6709701
H        0.4265962       -2.2740877        0.5905032
H        2.1381872       -2.2032505        1.0389733
```

```
vf_iproh_add_2.log
E =      -441.763438
H =      -441.546867
G =      -441.596038
NImag=0
C       -0.8324068       -1.4620476        1.6601480
C       -0.9530412       -0.9495288        3.0634183
N        0.4187347       -2.0461168        1.3958693
C        0.6109440       -2.9031872        0.3532645
H        1.6219951       -3.3238518        0.3166197
O       -0.2553156       -3.1689957       -0.4591585
H       -1.6407717       -2.1237346        1.3526897
H       -1.8880504       -0.4085635        3.2001246
H       -0.9438931       -1.8006825        3.7436346
H       -0.1150376       -0.2913895        3.2952010
H        1.1991687       -1.8277274        2.0066017
O       -0.9697123       -0.2511719        0.7458331
C       -2.3536460        0.1682707        0.3475475
H       -0.3826582       -0.3266886       -0.0362952
C       -2.2902390        1.6497998        0.0970302
C       -2.7860403       -0.6446325       -0.8428065
H       -2.9396768       -0.0625985        1.2360588
H       -2.1224086       -0.4556419       -1.6902773
H       -2.7988571       -1.7128161       -0.6256462
H       -3.7947584       -0.3394971       -1.1224083
H       -1.9327434        2.1831339        0.9770327
H       -1.6379703        1.8668696       -0.7510610
H       -3.2929958        2.0036173       -0.1440115
```

```
vf_iproh_add_3.log
E =      -441.761200
H =      -441.544355
G =      -441.592898
NImag=0
C       -0.4951524        1.2009004        1.5334307
C       -0.6333526        0.5446842        2.8754395
N       -1.3933374        2.2633507        1.3360869
C       -1.1820453        3.2456849        0.4159566
H       -0.2141430        3.1575718       -0.0985281
O       -1.9745785        4.1370327        0.1881731
H        0.5243909        1.4997967        1.2995529
H        0.0339849       -0.3112515        2.9466519
H       -0.3668018        1.2718653        3.6416137
H       -1.6627308        0.2230982        3.0457970
H       -2.2682413        2.2726392        1.8548763
O       -0.7251500        0.1073781        0.4954908
C       -2.1146374       -0.3859504        0.2198046
H       -0.2289905        0.2777371       -0.3307110
C       -2.4951066        0.0623051       -1.1661029
C       -2.0925445       -1.8765729        0.4191774
H       -2.7272076        0.1054025        0.9747859
H       -1.7600423       -2.1297138        1.4261946
H       -1.4338502       -2.3512199       -0.3099511
H       -3.1023822       -2.2632149        0.2778107
```

H	-1.8232557	-0.3787188	-1.9064370
H	-2.4700974	1.1494206	-1.2544738
H	-3.5078347	-0.2795369	-1.3794509

vf_iproh_add_4.log
E = -441.760865
H = -441.543791
G = -441.592119
NImag=0

C	0.7746806	1.4930741	1.6087639
C	0.9088489	0.9899733	3.0145662
N	-0.5039951	1.9736644	1.2980225
C	-0.7180634	2.9548573	0.3745351
H	0.2082957	3.3258633	-0.0883910
O	-1.8178952	3.3818664	0.0916289
H	1.5394108	2.2141936	1.3219682
H	1.8795343	0.5196671	3.1646161
H	0.8201913	1.8356778	3.6954976
H	0.1197111	0.2683948	3.2274817
H	-1.3088395	1.5842769	1.7836619
O	1.0456258	0.2855099	0.7081361
C	2.4651130	0.0398028	0.2993829
H	0.4357159	0.2739357	-0.0597354
C	2.6209993	-1.4472995	0.1423091
C	2.7501552	0.8354899	-0.9462075
H	3.0290376	0.4103234	1.1551383
H	2.3666348	-1.9680228	1.0648597
H	1.9912259	-1.8153834	-0.6697471
H	3.6607488	-1.6625061	-0.1060251
H	2.5687129	1.9005877	-0.7921161
H	2.1334164	0.4774815	-1.7738446
H	3.7980895	0.7030486	-1.2156600

vf_iproh_h2o_prod_1.log
E = -518.176524
H = -517.933445
G = -517.989273
NImag=0

C	0.5627786	0.1359075	0.3168636
C	0.7156215	1.5791327	0.7161109
N	1.6164414	-0.2856543	-0.5360960
C	1.8114520	-1.5955932	-0.8130882
H	2.6579734	-1.7838182	-1.4839604
O	1.1132976	-2.4882421	-0.3582122
H	0.4879950	-0.5313428	1.1750153
H	-0.1442363	1.9057874	1.2992937
H	1.6120914	1.6794976	1.3270286
H	0.8183827	2.2125407	-0.1662149
H	2.1821904	0.4120094	-1.0063186
O	-0.7139291	-0.0672493	-0.4001643
C	-1.7724075	-0.8598566	0.2672092
H	-1.0879184	0.7992165	-0.8700307
C	-2.3803244	-0.0498835	1.3849867
C	-2.7525701	-1.2464484	-0.8096895
H	-1.2401854	-1.7335983	0.6421864
O	-1.5644387	2.0026191	-1.5238991
H	-2.0304150	2.6285737	-0.9487882
H	-2.1302704	1.8508760	-2.2961900
H	-3.2390530	-0.3602254	-1.2239830

H	-3.5207322	-1.8850297	-0.3735146
H	-2.2560061	-1.7943321	-1.6105721
H	-3.1609860	-0.6405151	1.8654763
H	-2.8320438	0.8636685	0.9898134
H	-1.6387629	0.2120122	2.1409863

vf_iproh_h2o_prod_2.log
E = -518.179692
H = -517.937850
G = -517.991658
NImag=0

C	-0.4574052	0.4830611	-0.7840939
C	-0.6086240	1.9244758	-1.1862248
N	-1.3027705	0.1488036	0.3165547
C	-1.5996371	-1.1263290	0.6436752
H	-2.3071572	-1.2146654	1.4751643
O	-1.1341344	-2.1059040	0.0740524
H	-0.6465245	-0.1987472	-1.6132020
H	0.0692570	2.1514626	-2.0067262
H	-1.6334388	2.0955933	-1.5145748
H	-0.3966740	2.5893676	-0.3466100
H	-1.7211930	0.8977823	0.8580877
O	0.9464120	0.2167498	-0.4621437
C	1.5230332	0.7933882	0.7680772
H	1.2346242	-0.8068106	-0.7064526
C	1.9072794	-0.3344554	1.6942881
C	2.6766817	1.6707222	0.3546366
H	0.7216929	1.3867905	1.2086858
O	1.4240811	-2.1203157	-1.0034279
H	0.5416710	-2.5054407	-0.8260276
H	1.6589650	-2.3087862	-1.9249149
H	3.4506567	1.0736380	-0.1314737
H	3.1070846	2.1411836	1.2394914
H	2.3449586	2.4520698	-0.3303048
H	2.2660115	0.0880852	2.6331196
H	2.7089614	-0.9353610	1.2605338
H	1.0517582	-0.9785269	1.9075627

vf_iproh_h2o_ts.log
E = -518.153879
H = -517.913024
G = -517.972339
NImag=1

C	1.4245916	0.8559911	0.6454269
C	0.7091737	2.0210513	1.1573166
N	2.0482026	0.8711692	-0.4944730
C	2.7638888	-0.2628213	-0.9881069
H	3.1839363	-0.0964473	-1.9831017
O	2.8638244	-1.2655189	-0.3461651
H	1.5591777	-0.0293617	1.2549197
H	-0.2700766	1.7121070	1.5259121
H	1.2670529	2.3707029	2.0340550
H	0.6248867	2.8191754	0.4240899
H	1.9750655	1.6883497	-1.1033439
O	-0.3705474	-0.6097392	-0.2552140
C	-1.3678739	-1.3813690	0.4164575
H	-0.8043811	0.1673992	-0.6689028
C	-1.9157685	-0.6188269	1.6072904
C	-2.4654086	-1.7633365	-0.5576758

H	-0.8543026	-2.2814279	0.7629151
O	-1.3880837	1.7815462	-1.3486581
H	-1.9092341	2.3348006	-0.7502560
H	-1.9027452	1.7208554	-2.1655806
H	-2.9729359	-0.8618186	-0.9159411
H	-3.2062704	-2.4031165	-0.0748218
H	-2.0530925	-2.2952340	-1.4167188
H	-2.6656799	-1.2123356	2.1337862
H	-2.3908021	0.3083439	1.2693655
H	-1.1198676	-0.3686145	2.3117680

V. References

(1) Harder, E.; Damm, W.; Maple, J.; Wu, C.; Reboul, M.; Xiang, J. Y.; Wang, L.; Lupyan, D.; Dahlgren, M. K.; Knight, J. L.; Kaus, J. W.; Cerutti, D. S.; Krilov, G.; Jorgensen, W. L.; Abel, R.; Friesner, R. A. *J. Chem. Theory Comput.* **2016**, *12*, 281-296.

(2) Schrödinger Release 2019-4: MacroModel, Schrödinger, LLC, New York, NY (2019).

(3) Gaussian 16, Revision C.01, Frisch, M. J.; Trucks, G. W.; Schlegel, H. B.; Scuseria, G. E.; Robb, M. A.; Cheeseman, J. R.; Scalmani, G.; Barone, V.; Petersson, G. A.; Nakatsuji, H.; Li, X.; Caricato, M.; Marenich, A. V.; Bloino, J.; Janesko, B. G.; Gomperts, R.; Mennucci, B.; Hratchian, H. P.; Ortiz, J. V.; Izmaylov, A. F.; Sonnenberg, J. L.; Williams-Young, D.; Ding, F.; Lipparini, F.; Egidi, F.; Goings, J.; Peng, B.; Petrone, A.; Henderson, T.; Ranasinghe, D.; Zakrzewski, V. G.; Gao, J.; Rega, N.; Zheng, G.; Liang, W.; Hada, M.; Ehara, M.; Toyota, K.; Fukuda, R.; Hasegawa, J.; Ishida, M.; Nakajima, T.; Honda, Y.; Kitao, O.; Nakai, H.; Vreven, T.; Throssell, K.; Montgomery, J. A., Jr.; Peralta, J. E.; Ogliaro, F.; Bearpark, M. J.; Heyd, J. J.; Brothers, E. N.; Kudin, K. N.; Staroverov, V. N.; Keith, T. A.; Kobayashi, R.; Normand, J.; Raghavachari, K.; Rendell, A. P.; Burant, J. C.; Iyengar, S. S.; Tomasi, J.; Cossi, M.; Millam, J. M.; Klene, M.; Adamo, C.; Cammi, R.; Ochterski, J. W.; Martin, R. L.; Morokuma, K.; Farkas, O.; Foresman, J. B.; Fox, D. J. Gaussian, Inc., Wallingford CT, 2016.

(4) Yu, H. S.; He, X.; Li, S. L.; Truhlar, D. G. *Chem. Sci.* **2016**, *7*, 5032-5051.

(5) Weigend, F.; Ahlrichs, R. *Phys. Chem. Chem. Phys.* **2005**, *7*, 3297-3305.

(6) Marenich, A. V.; Cramer, C. J.; Truhlar, D. G. *J. Phys. Chem. B* **2009**, *113*, 6378–6396.

(7) Zhang, X.; Paton, R. S. *Chem. Sci.* **2020**,*11*, 9309-9324

(8) Luchini, G.; Alegre-Requena J. V.; Guan, Y.; Funes-Ardoiz, I.; Paton, R. S. (2019). GoodVibes: GoodVibes 3.0.1 http://doi.org/10.5281/zenodo.595246

(9) Grimme, S. *Chem. Eur. J.* **2012**, *18*, 9955-9964.

(10) Visualization was performed with CYLview20; Legault, C. Y., Université de Sherbrooke, 2020 (http://www.cylview.org).
